# Enhanced Mechanical Recycling of Polymer Mixtures by a Trifunctional Dynamic Crosslinker

**DOI:** 10.1002/adma.74752

**Published:** 2026-08-22

**Authors:** Xavier Westworth, Essa Shamsan, Yunpeng Gao, Nevaya Carr, Eugene Y.‐X. Chen

**Affiliations:** ^1^ Department of Chemistry Colorado State University Fort Collins Colorado USA

**Keywords:** dynamic exchange, plastics recycling, polymer compatibilization, universal dynamic crosslinking, upcycling

## Abstract

Mechanical recycling of mixed post‐consumer apolar/polar polymers, due to their mis‐matched polarity and inherent immiscibility, is typically a downcycling process yielding brittle materials. An emerging method that can enhance recycling of such mixtures into dynamically crosslinked, high‐performance thermosets is a dynamic crosslinker (DC) platform; however, current DCs typically require an external catalyst and, due to their insufficiently high peak activation temperature (*T*_a_ <190°C), are limited to a subset of applicable polymers and unsuitable for industrial melt‐extrusion processing of high melting temperature (*T*_m_) polymers. Herein, we report a trifunctional DC incorporating three sought‐after properties: thermally robust pyridotriazole cores as the high‐*T*_a_ (245°C) crosslinking sites compatible with reactive extrusion up to 270°C; dynamic siloxane linkages as the robust yet exchangeable bonds; and pyridine/ester functionalities as the internal catalysis sites devoid of external catalysts. Overall, this self‐catalyzed, high‐*T*_a_ DC can compatibilize waste plastic mixtures containing high *T*_m_ polymers and impart the recycled mixtures with superior thermoset properties such as enhanced creep resistance and thermomechanical stability while being melt‐(re)processable.

## Graphical abstract

A self‐catalyzed, trifunctional dynamic crosslinker that incorporates three desired properties into one system and is compatible with reactive extrusion can compatibilize waste plastic mixtures containing high *T*_m_ polymers and impart the recycled mixtures with superior thermoset properties such as enhanced creep resistance and thermomechanical stability while maintaining melt‐(re)processability.

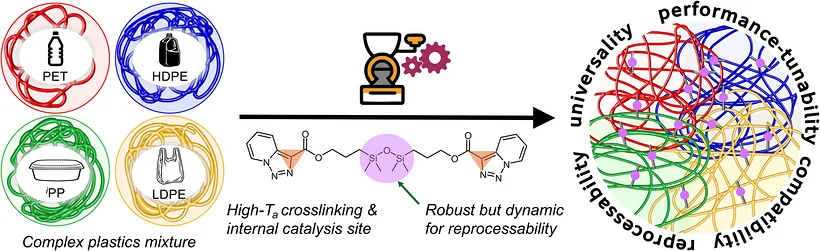

## 1 Introduction

Plastic waste accumulation in landfills continues to increase due to the lack of closed‐loop recycling and repurposing of waste plastics, which are causing shortages of landfills [1, 2, 3, 4]. These waste‐occupied landfills also contaminate the environment by leaching toxic chemicals and microplastics as a result of environmental weathering and photodegradation [5, 6, 7, 8]. To address this significant environmental issue, mechanical recycling has become a primary method to recycle or repurpose waste plastics, keeping them out of landfills [9, 10, 11, 12, 13]. However, a particularly challenging barrier in mechanical recycling is the processing of mixed plastic waste streams, which contain both polar polymers (e.g., polyesters, polyamides or nylons) and apolar polymers (e.g., polyolefins) [10, 11, 12, 13]. Even within apolar plastics, particularly common polyolefins, such as low‐density polyethylene (LDPE), high‐density polyethylene (HDPE), and isotactic polypropylene (*i*PP), are immiscible at room temperature, necessitating segregation of plastic types to prevent macrophase separation during melt processing [14, 15, 16, 17, 18]. These incompatibilities arise from chemical structure differences that lack enthalpic contributions to mixing and long polymer chains that result in the loss of entropic contributions to polymer mixing [13, 16, 19]. As a result, mechanically recycled mixed plastics often yield a droplet morphology with void spaces between different polymer phases and thus are frequently downcycled into brittle, lower‐value materials [10, 11, 12, 13, 14].

To mitigate phase separation during mechanical recycling, various compatibilization strategies have been developed, including (non)reactive compatibilizers, which improve the mechanical properties of mixed materials via entanglement, co‐crystallization, or supramolecular reactions between polymer domains [5, 16, 20, 21, 22, 23, 24, 25, 26, 27, 28, 29, 30, 31]. Block copolymer (BCP) compatibilizers represent one of the most widely studied non‐reactive strategies for improving interfacial adhesion in immiscible polymer blends [16, 17, 18]. By incorporating chemically distinct polymer blocks that preferentially partition into each polymer phase, BCPs localize at phase boundaries, reduce interfacial tension, and suppress macro‐phase separation during melt processing. However, these compatibilizers rely on multi‐BCPs that must be pre‐synthesized and specifically tailored to individual polymer pairs, limiting their applicability to complex and variable post‐consumer waste streams [2, 16, 20].

More recently, Chen and Guan groups independently introduced universal dynamic crosslinkers (UDCs) capable of compatibilizing immiscible polymer mixtures through in situ dynamic crosslinking [32, 33, 34]. These systems leverage facile C─H insertion chemistry, via thermally generated singlet carbenes or nitrenes to form dynamic crosslinked networks across chemically diverse polymer backbones, thereby developing a higher‐value thermoset material from mixed plastic waste. Despite these notable advances, the above UDC systems remain constrained by thermal processing limitations due to an insufficiently high peak‐activation temperature (*T*_a_) (Figure 1). Their *T*_a_ ranges (∼180°C for diazo‐based systems and 190°C for sulfonyl azide‐based systems) do not overlap with the melt‐processing windows of high melting temperature (*T*_m_) plastics and can trigger torque buildup during high‐temperature extrusion (vide infra) [32, 33, 34, 35].

**FIGURE 1:**
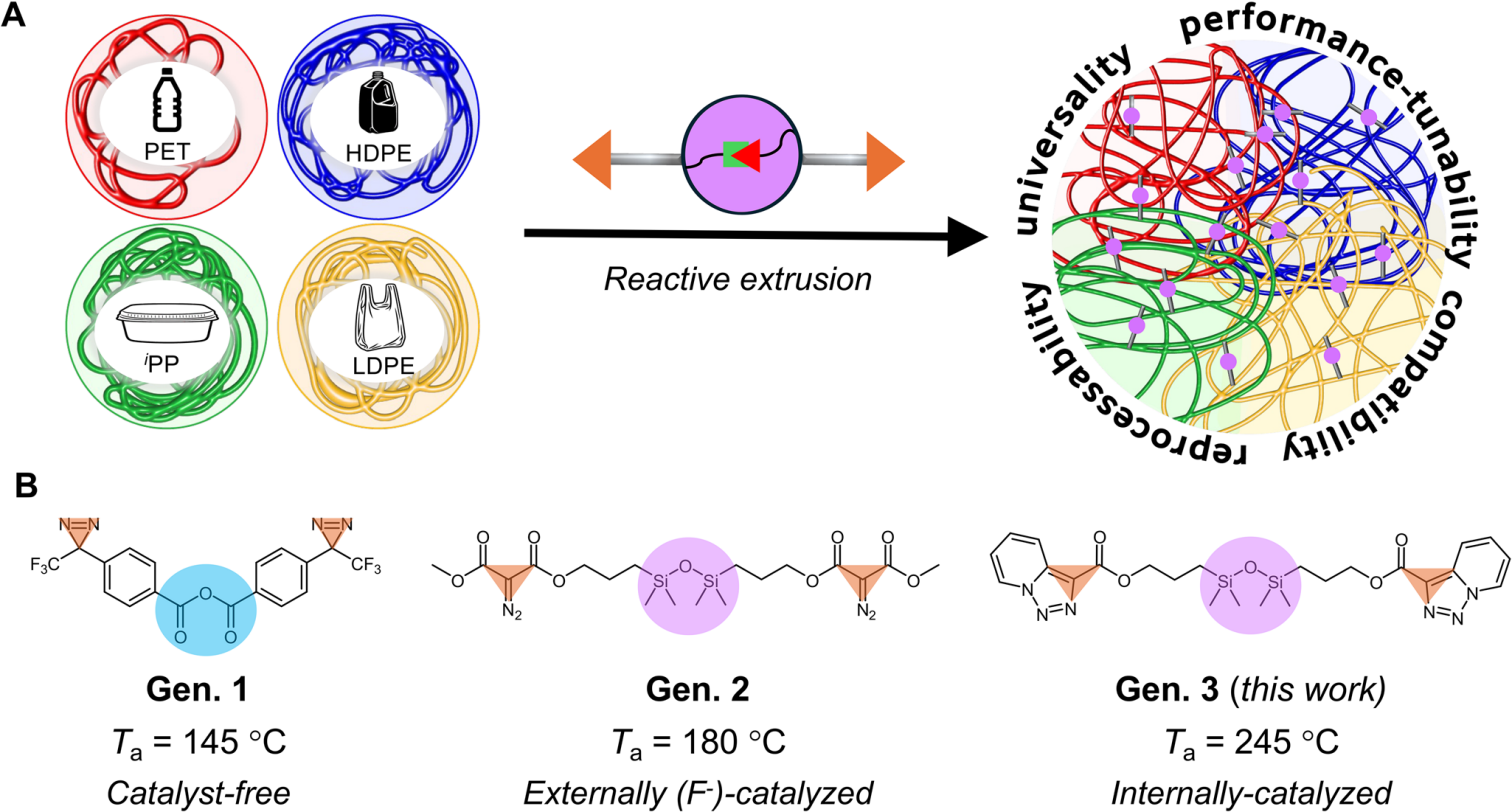
Design features of UDCs and advances in both carbene precursors (sites for crosslinking) and dynamic linkages (sites for melt‐processing). (A) Schematic representation of UDC compatibilization. (B) Comparison of the high‐*T*_a_ pyridotriazole carbene precursor (**Gen. 3**) to previously reported UDC systems (**Gen. 1** and **Gen. 2**).

In pursuit of a higher *T*_a_ dynamic crosslinker (DC) that could be compatible with reactive extrusion of thermally robust plastics or fibers with a high *T*_m_, such as poly(ethylene terephthalate) (PET) and Nylon 6 (*T*_m_ = 230°C–265°C), and overcome the thermal constraints of the previous DC systems, we arrived at a pyridotriazole‐based DC (PT‐DC) as a carbene precursor (Figure 1). Pyridotriazoles act as a “masked” carbene precursor in which two (closed and opened) skeletal isomers of the pyridotriazole can exist in equilibrium; the first is a triazole where the lone pair of the pyridine nitrogen donates into the pendant diazo nitrogen, furnishing a pyridotriazole (closed form), while the second isomer, formed under high temperatures, is a pyrido‐diazo (opened form) species capable of generating a reactive carbene for subsequent C─H insertion [36, 37, 38, 39]. Because the closed form is thermally robust, isomerization into the opened form occurs at ∼220°C with a peak *T*_a_ of ∼245°C [36]. Upon activation of the pyrido‐diazo, a carbene is generated, which is stabilized through donation into the empty *p*‐orbital of the carbene by the lone pairs of the oxygen (when there is an ester group substituted at the 3‐position of the triazole) and the pyridine [38]. The high peak *T*_a_ of the pyridotriazole moiety would allow this PT‐DC to be broadly applicable to 75% of the world's commodity polymers [1, 6, 10], allowing for high *T*_m_ polymers (e.g., PET and Nylon 6) to be compatible with reactive extrusion using this DC. Therefore, this high‐*T*_a_ DC approach would provide a broadly applicable route to polymer compatibilization and re/upcycling via reactive extrusion processing, irrespective of polymer type or waste stream complexity.

Furthermore, the dynamic exchange of the siloxane linkage provides a robust route toward dynamic networks with high thermal and oxidative stability, both of which are required during reactive extrusion [32, 33, 40, 41, 42, 43, 44]. Owing to the stability of the siloxane moiety, external catalysts such as Lewis acids or strong bases are commonly employed to enable efficient bond exchange for polymer network reconfiguration. However, a critical issue with an external catalyst to facilitate siloxane exchange is catalyst leaching, yielding a loss in performance over continued reprocessing [41]. To address the catalyst leaching issue, potassium fluoride embedded in a DB18:C6 ether was utilized in the previous siloxane exchange systems [32, 33, 40, 45]. These networks were shown not to leach such external catalysts and maintain performance over many reprocessing cycles. Nonetheless, a more advanced design would be based on the internally catalyzed siloxane exchange enabled by neighboring groups such as amide groups [43] or other accessible basic sites built into the DC framework.

Inspired by the above overviewed prior works, we hypothesized that assembling the three desired properties together into a trifunctional DC system, including the high‐*T*_a_ pyridotriazole, the thermally robust siloxane linkage, and the pyrido/ester internal catalysis site (Figure 1), could meet the need for a high‐*T*_a_ DC system that is compatible with reactive extrusion of high‐*T*_m_ polymers. Such a DC could be capable of compatibilizing a broad range of mixed plastic waste streams and enhancing the recycling of post‐consumer materials under industrially relevant processing conditions [46, 47]. In this design, a neighboring ester group to the siloxane is hypothesized to enhance the catalytic effect of the pyridine generated in situ upon activation of the DC (vide infra) to catalyze the siloxane exchange at elevated temperatures, much like the external or internal bases used in the previous systems [41, 43]. Together, the neighboring ester and adjacent pyridine sites could therefore sufficiently catalyze siloxane exchange during reactive extrusion without the need for an external catalyst, thus allowing for conventional reprocessing techniques, such as melt extrusion, for the generated network polymers. Overall, the herein designed high‐*T*_a_, PT‐based, and siloxane‐linked DC enables dynamic crosslinking of mixed plastics containing high *T*_m_ polymers such as PET without the need for external catalysts, effectively compatibilizing such mixtures and recycling them into dynamically crosslinked thermosets, while maintaining most features of the constituent polymers within these blends.

## 2 Results and Discussion

### 2.1 Synthesis of PT‐DC as Masked Carbene Precursor

PT‐DC was synthesized in four steps from commercial starting materials (Figures 2A and S1–S3). To determine the peak *T*_a_ of the PT moiety, the DC was subjected to thermal activation monitored by Differential Scanning Calorimetry (DSC), showing an onset *T*_a_ of 219°C and a peak *T*_a_ of 244°C (Figure S4). The importance of the pyridine at the 2‐position, with respect to the diazo moiety in the opened isomer form, can be highlighted by comparing previous literature. Pyridine groups placed at the 3‐ and 4‐positions exhibit a low onset *T*_a_ of 108°C and 133°C, respectively [26]. The 2‐position, on the other hand, enables a high onset *T*_a_ of 219°C because it is capable of donating into the diazo moiety, thereby forming a highly stable PT. Thus, the *T*_a_ observed for PT‐DC is reliant on overcoming the activation barrier of pyridotriazole isomerization to allow for nitrogen release and subsequent C─H insertion (Figure 2B). Additionally, the lone pair on the pyridine group can donate into the empty *p*‐orbital of the carbene, providing a stabilized carbene capable of undergoing C─H insertion.

**FIGURE 2:**
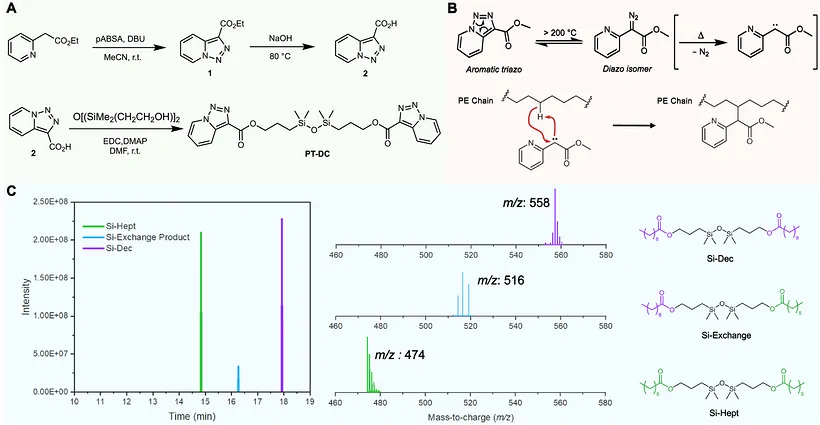
(A) Outlined synthetic pathway to PT‐DC. (B) Thermally induced isomerization of pyridotriazole to pyrido‐diazo adduct and subsequent thermal degradation to generate the reactive carbene for C─H insertion. (C) APGC elution peaks and their corresponding mass‐to‐charge ratio for Si‐Hept (14.8 min, 474 *m/z*), Si‐Exchange (16.2 min, 516 *m/z*), Si‐Dec (17.9 min, 558 *m/z*).

### 2.2 Evidence for Self‐Catalyzed Siloxane Exchange in PT‐DC

After displaying compatibilization in the diazo malonate‐based DC system without an external catalyst at low DC loadings (1.0 wt%), we hypothesized that neighboring malonates/esters could catalyze siloxane exchange [32]. Accordingly, a control experiment to probe the exchange dynamics of siloxane networks with neighboring ester groups in a dynamically crosslinked HDPE system was designed using a two‐armed diazo DC [32], which was reacted in the extruder at 180°C and 100 rpm for 8 min. This sample was then molded and directly used for stress relaxation experiments (210°C–250°C). Additionally, bipyridine was added to a sample of the two‐armed diazo DC during extrusion due to pyridine's thermal constraints (low boiling point). We hypothesized that, with the external bipyridine as the surrogate for the internal pyridine present in the current PT‐DC system, a decrease in the activation energy of the siloxane bond exchange would demonstrate the internal pyridine in the DC to be an effective catalyst. Indeed, the bipyridine‐catalyzed siloxane exchange showed a marked decrease in the activation energy of 28.4 kJ/mol, relative to a much higher energy barrier of 177.4 kJ/mol for the uncatalyzed siloxane exchange system (Figures S102 and S100). Overall, these results provided evidence that PT‐DC would not require an external catalyst to activate siloxane exchange, as the neighboring ester groups and catalysis of the exchange by adjacent pyridines can effectively catalyze the dynamic exchange of polymer networks.

To provide further evidence for siloxane exchange enabled by the neighboring ester and adjacent pyridine moieties, two small molecule models, (1,1,3,3‐tetramethyldisiloxane‐1,3‐diyl)bis(propane‐3,1‐diyl) didecanoate (Si‐Dec, Figures S8 and S9) and (1,1,3,3‐tetramethyldisiloxane‐1,3‐diyl)bis(propane‐3,1‐diyl) diheptanoate (Si‐Hept, Figures S7 and S10) were mixed in a 1:1 ratio, and 0.5 eq of pyridine was added as a catalyst. The mixture was heated for 2 h at 200°C to yield the final mixture containing three products: Si‐Dec (558 *m/z*; exact mass: 558.41), Si‐Hept (474 *m/z*; exact mass: 474.32), and the crossover (exchange) product (516 *m/z*; exact mass: 516.37), characterized by Atmospheric Pressure Gas Chromatography (APGC) (Figure 2C). The presence of the siloxane crossover product provides further evidence for the dynamic siloxane exchange. In contrast, treating Si‐Dec and Si‐Hept under identical conditions but without adding the pyridine catalyst resulted in no crossover product formation (Figures S5 and S6), highlighting the essential role of the pyridine site for internal or external catalysis with the ester neighboring group.

### 2.3 Dynamic Crosslinking of HDPE and LDPE

To evaluate the efficacy of PT‐DC for dynamic crosslinking by reactive extrusion, initial studies focused on dynamic crosslinking of polyethylene, specifically LDPE and HDPE. They were selected due to their thermal stability under high‐temperature extrusion conditions (>240°C), which are required to activate the DC crosslinker, and the absence of functional groups scattered throughout the polymers that could aid in catalyzing siloxane exchange. During reactive extrusion of H(L)DPE with PT‐DC, there was an increase in the pressure buildup within the extruder (Figure 3A). This pressure buildup is indicative of nitrogen release from the open‐form diazoisomer and the subsequent in situ crosslinking of the polymer [32, 33]. Owing to the fast reaction time of the DC, a residence time of 3 min and 200 rpm was utilized for all polymer samples to align our work with current industrial processing of plastic recycling [10, 11, 12, 13, 37].

**FIGURE 3:**
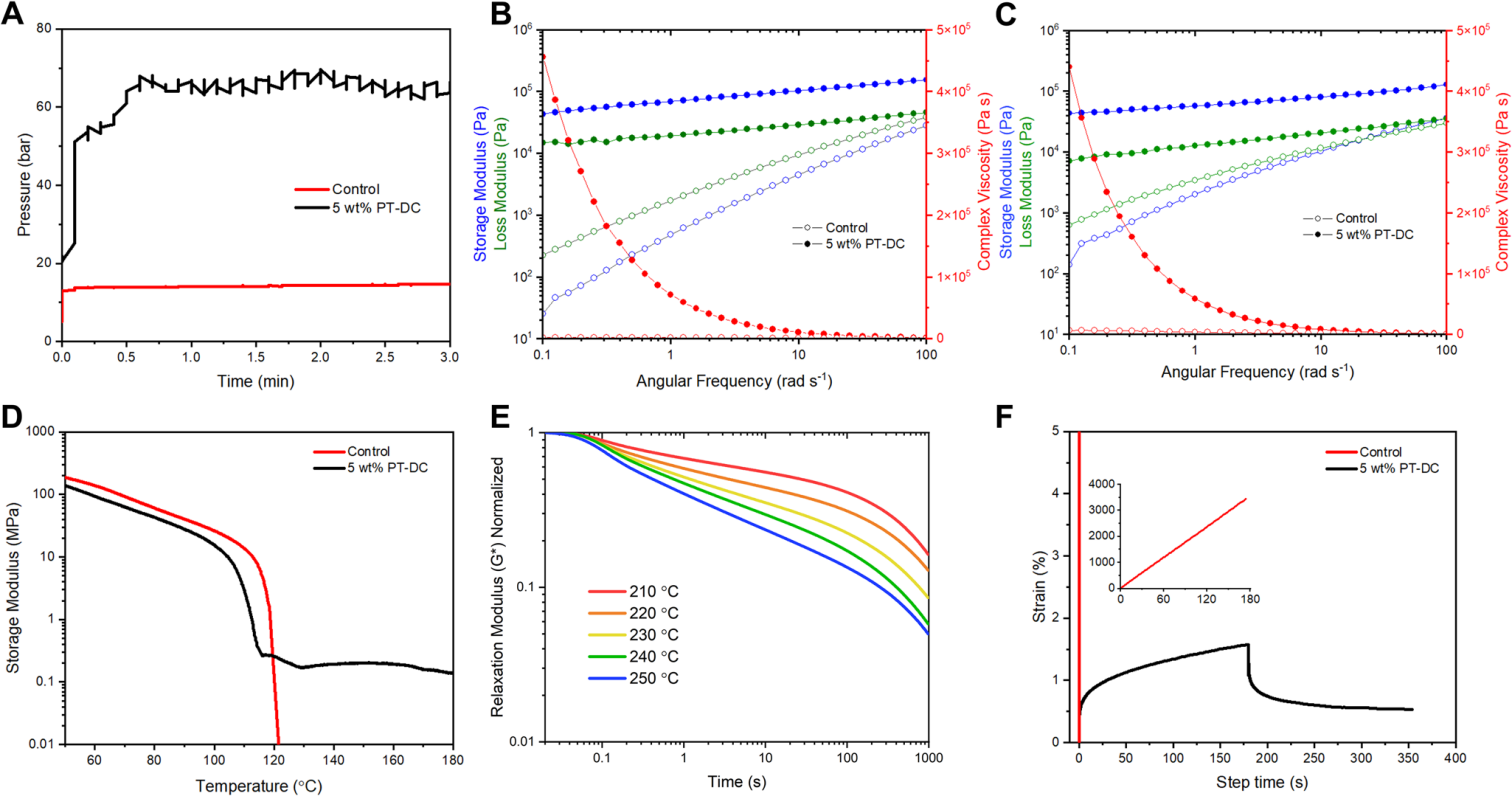
Evidence for dynamic crosslinking of HDPE and LDPE. (A) Extrusion profile of HDPE at 200 rpm and 250°C with PT‐DC (black) and without the DC (red). (B) Frequency sweep of HDPE treated with 5 wt% PT‐DC at 180°C. (C) Frequency sweep of LDPE treated with 5 wt% of PT‐DC at 180°C. (D) Temperature sweep of LDPE control (red) and crosslinked (black). (E) Stress relaxation of HDPE from 210°C to 250°C. (F) Creep recovery of HDPE at 180°C.

Frequency sweep experiments indicated that HDPE and LDPE dynamically crosslinked with 5 wt% PT‐DC, despite their ability to enter viscous flow at high temperatures and shearing frequencies, display a superb elastic response to the imparted oscillatory shear (Figure 3B–D). To quantify the exchange dynamics of PT‐DC, stress relaxation of the dynamically crosslinked HDPE above its *T*_m_ should elucidate the activation energy for siloxane exchange, given the Arrhenius dependence of these covalent adaptable networks. Therefore, stress relaxation experiments were carried out on the crosslinked HDPE from 210°C–250°C (Figure 3E) and plotted to yield an activation energy of 224 kJ/mol or 53.5 kcal/mol (Figures S98and S99). Quantification of crosslinking in the crosslinked HDPE and LDPE was determined via gel fraction measurements, performed on polymer samples crosslinked with 5 wt. % PT‐DC. For LDPE, gel fractions were obtained, corresponding to an estimated crosslinking extent of approximately 63.5%. By comparison, HDPE displayed a lower gel fraction of approximately 35.6% (Table S1). To further corroborate crosslinking in these samples, temperature sweeps were carried out in LDPE and HDPE, displaying rubbery plateaus at 0.2 and 0.7 MPa, respectively (Figures 3D and S65). Creep‐recovery experiments above the *T*_m_ of each respective polymer revealed that the crosslinked LDPE and HDPE exhibit pronounced creep resistance over 3 min, characterized by a low total strain and substantial elastic recovery. Specifically, the crosslinked HDPE sample displayed a total strain of about 1.7% recovered to 0.5% strain, while the LDPE sample exhibited a total strain of 1.9% and recovered to 0.4% strain (Figures 3F and S40). These results indicate that the formation of a crosslinked network enhanced creep resistance by 2400‐fold in PT‐DC‐treated HDPE polymers above its *T*_m_. DSC and thermogravimetric analysis (TGA) experiments were performed for all the resulting polymers (Figures S11–S17 and S26–S30).

Oscillatory shear frequency sweep measurements further supported the presence of a crosslinked network in the PT‐DC‐treated HDPE and LDPE samples (Figure 3B,C). In the crosslinked samples, a distinct first‐order decrease in complex viscosity with increasing frequency demonstrated the ability of these dynamic networks to exchange rapidly, allowing for viscosities comparable to those of a non‐crosslinked control at shearing rates of 60–100 rad s^−1^ (Figure 3B,C). Creep recovery, frequency sweeps and temperature sweep experiments were performed for all homopolymers (Figures S38–S40, S48–S52, and S65). Thus, the fast dynamic exchange allows for these networks to be processed via melt extrusion, similarly to conventional thermoplastics, highlighting the importance of dynamic siloxane exchange in these systems for their melt‐processability. Representative uniaxial tensile testing of homopolymers and their uniaxial tensile testing data after subsequent reprocessing cycles were collected, showing a decrease in elongation‐at‐break over multiple reprocessing cycles (Figures S74–S77).

## 3 Compatibilization and Reprocessing of Apolar/Polar Binary Plastic Blends

Binary blends of mismatched polarity were investigated to demonstrate effective crosslinking between apolar polyolefins (LDPE, HDPE, and *i*PP) and polar polyesters or polyamides (PET or Nylon 6) upon reactive extrusion with PT‐DC. These polymers were individually sourced from post‐consumer waste: HDPE (milk jug), LDPE (transparent bag), *i*PP (container), PET (soda bottle), and Nylon 6 (commercial powder). Three binary blends were tested for compatibilization by reactive extrusion with PT‐DC: HDPE‐*i*PP (70:30), LDPE‐PET (80:20), and HDPE‐PET (80:20). A representative image of the samples produced from PT‐DC functionalization is shown in Figure S104. DSC for all the corresponding blends were performed showing a decrease in the melt endotherm but displaying comparative TGAs for all blends (Figures S18–S22 and S31–S34). HDPE‐Nylon 6 compatibilization was performed with polymer pellets, as the majority of Nylon 6 waste contains elastomer or cottons that cannot be melt‐extruded, and thus these results were used as a proof of concept. These binary blends were then melt‐compounded at 250°C (blends without PET) or 270°C (blends with PET) with 5 wt% of PT‐DC for 3 min to ensure full melting of the constituent polymers and homogenous compatibilization during reactive extrusion [30, 31, 32]. Again, highlighting the robust nature of PT‐DC, its high peak *T*_a_ allows this DC to target these high‐*T*_m_ polymers (PET and Nylon 6) that were previously inaccessible by carbene‐mediated C─H insertion [23, 24, 25].

Frequency and temperature sweeps, as well as creep‐recovery experiments, were conducted to demonstrate crosslinking in these PT‐DC‐treated samples (Figures S41–S44, S53–S60, and S66–S68). Frequency sweep experiments conducted at 270°C indicated thermoset‐like behavior of the compatibilized HDPE–PET blend, as the crosslinked network exhibits a strong elastic response well above its *T*_m_, while maintaining pronounced shear thinning as a result of fast dynamic siloxane exchange (Figure 4A). The results from the creep–recovery experiments showed a significant improvement in deformation resistance for the DC‐compatibilized binary blends relative to the uncompatibilized controls. For example, the HDPE‐*i*PP (70:30) blend displayed a total strain of about 5% with a recoverable strain to 2.5%, while the HDPE‐Nylon 6 (80:20) and HDPE‐PET (80:20) blends exhibited total strains of 10.3% and 2.4%, with recoveries to 5.2% and 0.9%, respectively (Figures S44 and 4B). In contrast, the uncompatibilized blends showed a 150‐fold increase in irreversible deformation due to the lack of crosslinking in these systems (Figure 4B). As creep‐recovery experiments measure bulk resistance to deformation, this experiment is unable to provide insight into crosslinking between the two‐phase domains. Hence, temperature sweep experiments were additionally conducted to offer insight into the crosslinking between the two polymers via melting transitions. The control blend contains no secondary structures (i.e., crosslinking) to maintain the polymer structure above the *T*_m_ of the major phase, that is, LDPE in the LDPE‐PET blend. At this point, material failure occurs, and no secondary *T*_m_ can be elucidated as the bulk of the material enters viscous flow (Figure 4C). The DC‐compatibilized blend, on the other hand, maintained its thermomechanical stability past the first *T*_m_ and displayed a decreasing plateau until the second *T*_m_ is reached. Upon reaching the second *T*_m_, a decrease in the storage modulus results from polymer chain movement in the PET minor phase. The final rubbery plateau (Figure 4C) represents the secondary structures present within each polymer phase and between polymer domains because of the crosslinks “stitching” these two polymers together.

**FIGURE 4:**
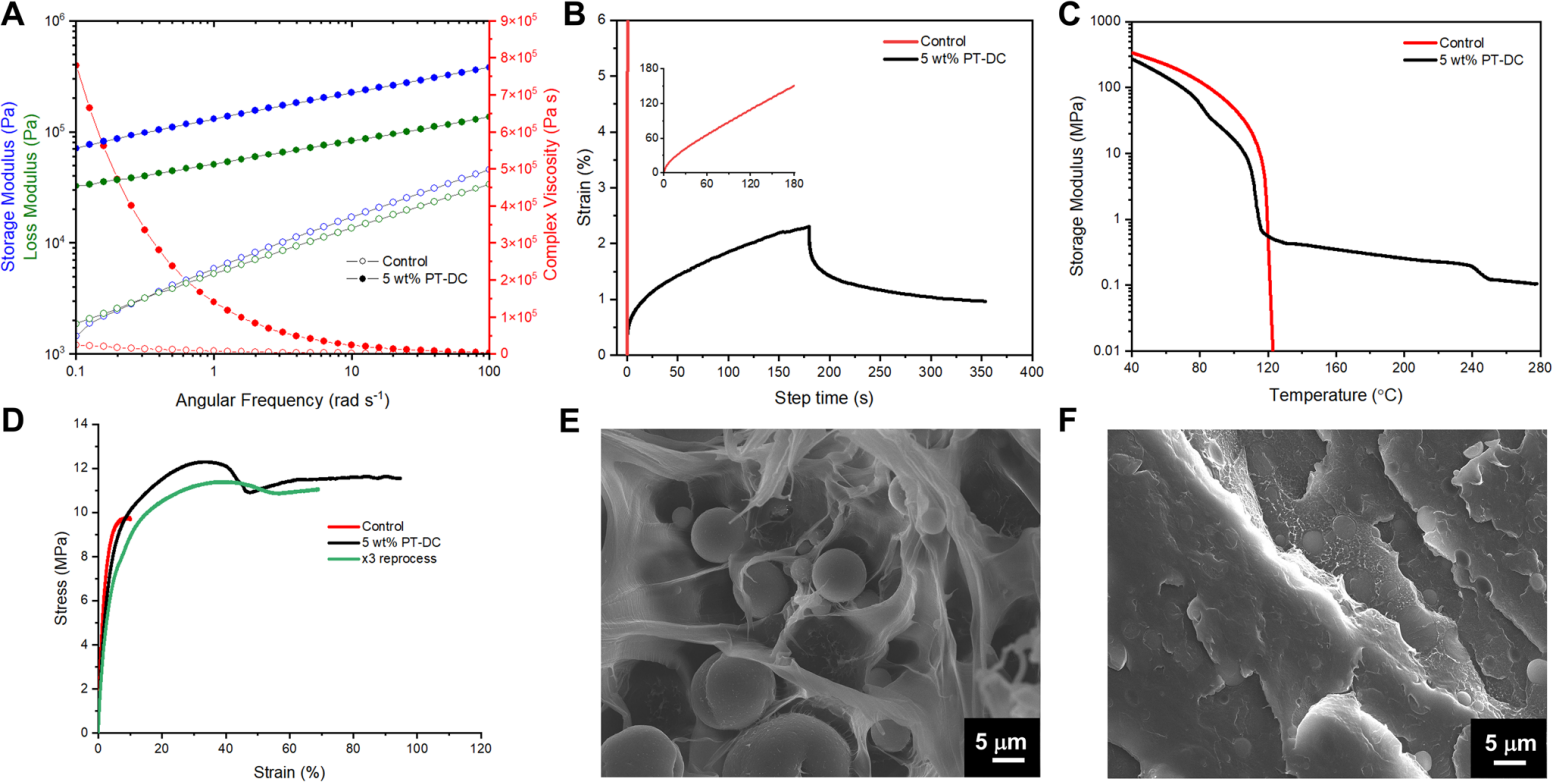
Evidence for dynamic crosslinking of high *T*_m_ PET/polyolefin binary blends. (A) Frequency sweep of the crosslinked HDPE‐PET (80:20) blend (filled spheres) and uncrosslinked HDPE‐PET (80:20) blend (unfilled spheres) at 270°C. (B) Creep‐recovery of the HDPE‐PET crosslinked blend (black) and the uncrosslinked HDPE‐PET blend (red). (C) Temperature sweep of the dynamically crosslinked LDPE‐PET (80:20) blend (red) and uncrosslinked LDPE‐PET blend (red). (D) Tensile test of the crosslinked LDPE‐PET (black) and its sample, after three reprocessing cycles (green), overlaid with the control LDPE‐PET blend (red). (E) SEM image of the LDPE‐PET blend control. (F) SEM image of compatibilized LDPE‐PET blend with 5 wt% pyridotriazole DC.

To further corroborate these results, scanning electron microscopy (SEM) imaging of the LDPE‐PET blend was conducted. A distinct phase domain morphology is present where the stringy major phase of LDPE contains small spheres of the PET minor phase within the control blend (Figures 4E and S96). The void spaces between LDPE and PET phases in the droplet morphology are a result of poor interfacial adhesion between these two immiscible polymers, thus creating brittle materials. However, upon compatibilization with 5 wt% of PT‐DC, the micro‐phase domain morphology is minimized (Figures 4F and S97), indicative of interfacial adhesion between the two domains as a result of dynamic crosslinking. This effect on material performance is further evidenced by tensile experiments in which the LDPE‐PET control broke at around 9%–10% elongation at break. Upon compatibilization with PT‐DC, the extensibility of the blend increased to 80%–95%, providing an eight‐ to nine‐ fold improvement in performance after the first extrusion (Figures 4D, S71, and S72). Upon further reprocessing in three consecutive cycles, a seven‐ to eight‐ fold improvement in the elongation‐at‐break compared to the control blend was observed, highlighting some degree of reprocessability (Figures 4D and S73). Compatibilization of apolar/polar blends was further extended to HDPE/Nylon‐6 (80:20), where a seven‐ to eight‐ fold improvement in tensile elongation‐at‐break compared to a control blend resulted upon addition of 5 wt% PT‐DC (Figures S78–S80). Overall, effective compatibilization of polymer blends between apolar polyolefins (HDPE and LDPE) and polar high‐*T*_m_ polymers (PET and Nylon 6) is shown here for the first time by the DC systems, highlighting a noteworthy advance in developing compatible DCs for dynamic thermosets from immiscible polymer mixtures.

## 4 Compatibilization of Complex Plastic Waste Mixtures

As demonstrated above, the higher *T*_a_ of PT‐DC in comparison to previous generations of DCs enabled reactive extrusion processing of high‐*T*_m_ polymers such as PET, thus presenting an opportunity for effective compatibilization of mixed plastic waste comprising multiple different plastics, including those high‐*T*_m_ plastics or fibers. The successful reactive processing of such complex polymer mixtures would further underscore the versatility of PT‐DC and its ability to compatibilize a broader range of plastic systems.

Owing to the difficulties associated with effective plastic waste segregation, including the prevalence of multimaterial packaging, post‐consumer plastic waste streams are inherently heterogeneous, often comprising complex systems with three or more polymer types [6, 10, 11, 12, 13]. To showcase the universality of PT‐DC and address this critical issue in plastic recycling, a simulated plastic waste stream was developed based on the polymers and compositions of which it is constituted [1, 22, 23]. Therefore, a ternary mixture of apolar polyolefins (LDPE: 35%, HDPE: 28.6%, and *i*PP: 36.4%) as well as a quaternary mixture including PET (LDPE: 30%, HDPE: 24.4%, *i*PP: 31.1%, and PET: 14.4%) were investigated. These plastics were sourced from post‐consumer plastic waste (vide supra), shredded, and then melt‐compounded at 250°C (ternary blend) or 270°C (quaternary blend) for 3 min to ensure full activation of PT‐DC.

To demonstrate that the crosslinking connections within the current blend system are dynamic (i.e., via siloxane linkages inherent in the PT‐DC system), we treated the control blend and DC‐blend using TBAF (tetrabutylammonium fluoride), which is known to be highly effective in cleaving siloxane linkages. As can be seen from the gel fraction analysis data presented in Figure S37, addition of TBAF to the crosslinked blend enabled complete dissolution of the blend, further confirming the dynamic chemical connections within the PT‐DC blend system. Thermomechanical and rheological properties of the DC‐compatibilized ternary and quaternary blends were characterized in comparison to the compatibilized controls (Figures S23–S25, S35, S36, S45–S47, S61–S64, and S69–S70). In creep recovery experiments, the ternary blend displayed a total strain of 3.3% with a recoverable strain to 0.97% (180°C), while the quaternary blend exhibited total strains of 11.6%, with recoveries to 4.5% (270°) (Figures S45 and S46). In comparison, the control blends (without PT‐DC) exhibited pronounced permanent deformation of 9865% strain without any recovery of the imparted deformation. These results highlight the high efficacy of PT‐DC to suppress creep even above the highest *T*_m_ in the compatibilized mixture of 255°C. The frequency sweep data shown in Figure 5A further corroborated the efficacy of the PT‐DC, as the storage modulus remained at 15 000 Pa above the loss modulus, indicating a thermoset‐like behavior. In contrast, the control sample exhibited a loss modulus exceeding the storage modulus, typical of viscous behavior. Temperature sweep experiments were taken of the two blends to provide further insight into the crosslinking between polymer domains. The LDPE/HDPE/*i*PP ternary blend exhibited high crosslinking and compatibilization of the domains via covalent crosslinking as each *T*_m_ in the resulting blend can be delineated and the presence of a rubbery plateau above the final *T*_m_ of *i*PP, around 165°C–170°C (Figure 5B). Likewise, the LDPE/HDPE/*i*PP/PET quaternary blend showed strong interfacial adhesion of the apolar polyolefins to the polar PET. The rubbery plateau of the compatibilized blend continued beyond the final *T*_m_ of PET (245°C–250°C), highlighting the dimensional stability offered by the siloxane crosslinks (Figure S70).

**FIGURE 5:**
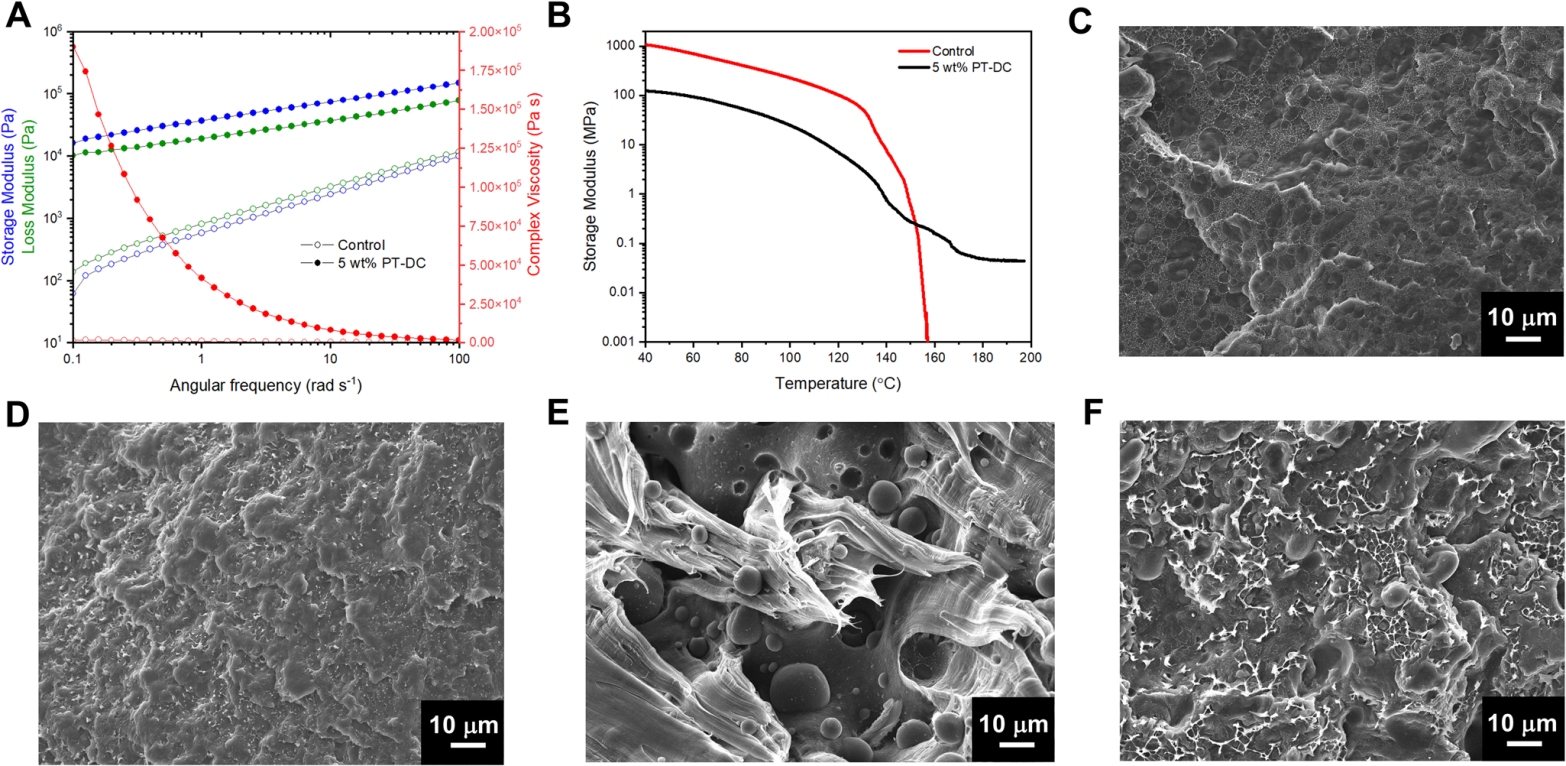
Evidence for dynamic crosslinking of simulated complex post‐consumer plastic waste. (A) Frequency sweep of the crosslinked quaternary blend (filled spheres) and uncrosslinked quaternary blend (unfilled spheres) at 270°C. (B) Temperature sweep of the crosslinked ternary (LDPE/HDPE/*i*PP) blend (black) and control blend (red). (C) SEM image of ternary blend control (LDPE/HDPE/*i*PP). (D) SEM image of ternary blend (LDPE/HDPE/*i*PP) with 5 wt% PT‐DC. (E) Representative SEM image of the quaternary blend control. (F) Representative image of the compatibilized quaternary blend with 5 wt% PT‐DC.

SEM images of the blends before and after DC‐compatibilization provide further evidence for compatibilization. The excellent compatibility was evident in the ternary blend in which a completely homogenous cross‐sectional area resulted from dynamic crosslinking (Figures 5D and S90) compared to that of the control blend (Figures 5C and S91). Such improvements in blend homogeneity were evidenced by uniaxial tensile testing in which a 40‐fold improvement in elongation at break resulted when compared to a control blend (Figures S81–S83). In addition, SEM images of the quaternary PET blend show that large polymer domains are present in the control blend with craters indicating incompatibility between the major phase polyolefins and the PET domains (Figures 5E and S92). Dynamic crosslinking of the blends resulted in the minimization of the droplet morphology within the samples and a continuous phase domain, suggesting the compatibilization of the apolar polyolefins with PET (Figures 5F, S93, and S94). Upon further reprocessing of the compatibilized quaternary blend, morphological stabilization of the phase domains resulted in improved blend homogeneity (Figure S95). However, despite the improved adhesion between these phases, the tensile properties of these blends showed only moderate improvements compared to the control blends (Figures S84–S89). The complexity of stress transfer between the domains of this complex blend of polymers, significantly different rigidities of the constituent polymers, and high‐crosslinking density would therefore develop brittle blends. Our ongoing study is investigating compatibilization and recycling of polyolefin (LDPE, HDPE, and *i*PP) feeds containing 1–5 wt% of polar polymers, including PET, Nylon 6, and polylactide, which represent a more realistic scenario of polar contamination in the reclaimed polyolefin materials.

## 5 Conclusion

Here, we introduced a trifunctional UDC, PT‐DC, that is thermally robust (with a high peak *T*_a_ of 245°C) and internally catalyzed (without externally added catalyst or additive), thereby allowing for compatibilization and enhanced recycling of high‐*T*_m_ containing plastic mixtures via reactive extrusion processing. This new trifunctional DC assembles the three desired properties together into a single DC: the pyridotriazole cores as the high‐*T*_a_ crosslinking sites, the siloxane linkage as the robust yet exchangeable dynamic bonds, and the pyridine/ester functionalities as the internal catalysis sites. Universal dynamic crosslinking enabled by PT‐DC has pushed the boundaries of carbene‐mediated C─H insertion, boasting a high peak *T*_a_ of >245°C. Coupled with dynamic exchange of the siloxane moieties in the presence of neighboring ester groups and adjacent pyridines, we have developed a system capable of effective reactive extrusion and compatibilization between high *T*_m_ polymers such as PET and Nylon 6 with apolar polyolefins (HDPE, LDPE, and *i*PP), notably expanding the scope of this DC technology to a wider range of polymer types. These compatibilized blends exhibit enhanced creep resistance and thermomechanical stability whilst demonstrating fast dynamic exchange under processing conditions, allowing for fast network rearrangement and equilibration of polymer blend networks. PT‐DC also renders blend homogeneity during post‐extrusion processing in binary and even quaternary blends at 270°C, therefore addressing a critical hurdle in DC compatibilization for enhanced mechanical recycling.

## Author Contributions

**Eugene Y.–X. Chen**: funding acquisition, Writing – review and editing, conceptualization, project administration, supervision, resources. **Essa Shamsan**: investigation, writing – original draft, conceptualization, methodology, validation, visualization, writing – review and editing, formal analysis, data curation. **Nevaya Carr**: investigation, data curation. **Xavier Westworth**: conceptualization, investigation, writing – original draft, methodology, validation, visualization, writing – review and editing, formal analysis, data curation. **Yunpeng Gao**: conceptualization, investigation, writing – original draft, validation, methodology, visualization, data curation.

## Conflicts of Interest

The authors declare no conflicts of interest.

## Supporting information




**Supporting File**: adma74752‐sup‐0001‐SuppMat.pdf.

## Data Availability

The data that support the findings of this study are available in the Supporting Information of this article.
