# Supplementary material for: Enhanced Mechanical Recycling of Polymer Mixtures by a Trifunctional Dynamic Crosslinker
Source: Adv Mater. 2026 Aug 22;38(58):e74752. doi: 10.1002/adma.74752 (PMC13674613; doi:10.1002/adma.74752)
Supplement: Supplementary file 1 [file ADMA-38-e74752-s001.pdf]

Supporting Information

Enhanced Recycling of Polymer Mixtures Enabled by A Trifunctional Dynamic Crosslinker

*Xavier Westworth, Essa Shamsan, Yunpeng Gao, Nevaya Carr, Eugene Y.-X. Chen**

Table of Contents

Materials and Methods......S1
Materials......S1
Polymer Crosslinking Procedures......S1
Instruments and General Characterization Procedures......S2
DC Preparation......S4
Synthesis and mechanistic pathways for internal siloxane catalysis......S4
Characterization of Crosslinker......S7
GC-MS and LC-MS Analysis......S9
Characterization of Model Substrates......S11
Figures and Tables......S15
DSC......S15
TGA......S24
Gel Fraction......S30
Creep-Recovery......S31
Frequency Sweep......S37
Temperature Sweep......S45
Tensile......S49
Scanning Electron Microscopy......S59
Stress Relaxation......S63
Representative polymer sample after PT-DC functionalization......S66
References......S67

Materials and Methods

Materials

All starting materials were obtained from commercial suppliers and used as received unless otherwise noted. Irganox 1010 and heptanoic acid were purchased from Sigma-Aldrich, 2,2-bipyridine and pyridine were purchased from Alladin, ethyl 2-(pyridin-2-yl)acetate was purchased from Ambeed, decanoic acid and 1,1,3,3-tetramethyldisiloxane was purchased from TCI Chemical Co., allylic alcohol was purchased from Alfa Aesar, 4-acetamidobenzenesulfonyl azide was purchased from Oakwood Chemical, Platinum(0)-1,3-divinyl-1,1,3,3-tetramethyldisiloxane complex solution in xylene, Pt ~2 % was purchased from Beantown Chemical, 1,8-diazabicycloundec-7-ene (DBU) was purchased from Fisher Scientific, EDC-HCl and Dicyclohexylcarbodiimide were purchased from Abeed Chemical, sodium hydroxide was purchased from Fisher Scientific, tetrabutylammonium fluoride hydrate solution (1.0 M in THF) was purchased from TCI Chemicals, and 4-(Dimethylamino)pyridine was purchased from Sigma Aldrich. High-performance liquid chromatography (HPLC) solvents (DCM, DMF, MeCN) were purchased from commercial suppliers and used as received. Activated 3Å mol sieves were added after opening HPLC-grade DCM, DMF, and MeCN bottles.

Commercial polymer specifications were as follows: HDPE (Goodfellow, M_n = 71.6 kDa), LDPE (Goodfellow, M_n = 80.5 kDa), *i*PP (Goodfellow, M_n = 337 kDa), Nylon-6 (Goodfellow, M_n = 23.5 kDa), and PET (Goodfellow, M_n = 35.2 kDa).

Synthesis of crosslinkers and small-molecule intermediates was performed in flame-dried glassware under nitrogen atmospheres on a dual-manifold Schlenk line (see specific procedures in the synthesis sections) unless otherwise specified. Reactions were stirred magnetically and monitored by analytical thin-layer chromatography (TLC) using Merck silica gel 60 F254 plates, visualized under 254 nm UV light, and by aqueous potassium permanganate staining followed by heating. Crude products were adsorbed onto silica and purified by flash column chromatography using SiliaFlash (SiliCycle Silica Gel P60, 230–400 mesh, 40–63 µm particle size) with analytical-grade hexane and ethyl acetate as eluents. Solvent removal was performed under reduced pressure by rotary evaporation at 40 °C unless otherwise noted. Reported yields refer to isolated, purified products.

The diazomalonate UDC (**UDC 2**) was prepared according to literature procedures, and its spectroscopic data matched reported values.[2]

Polymer Crosslinking Procedures

Polymer Crosslinking Procedure of the Bifunctional Diazomalonate UDC

HDPE powder (5.0 g) was added to a 50 mL centrifuge tube with 10 mg of Irganox 1010. The diazomalonate UDC was pulled up into a 1 mL syringe (~250 mg). The mixture was added to the micro-compounder at 180 °C and 100 rpm, and the UDC was added directly to the extruder over 1 min. The polymer sample was collected and directly used for rheological analysis.

Reactive extrusion in the presence of external base. HDPE powder (5.0 g) was added to a 50 mL centrifuge tube with 10 mg of Irganox 1010 and 160 mg of bipyridine (1 eq. with respect to the diazomalonate siloxane crosslinker). The diazomalonate UDC was pulled up into a 1 mL syringe (~250 mg). The mixture was added to the micro-compounder at 180 °C and 100 rpm, and the UDC was added directly to the extruder over 1 min. The polymer sample was collected and directly used for rheological analysis.

General Polymer Crosslinking (Melt Processing)

A polymer sample (5.0 g) was combined with Irganox 1010 (10 mg) and PT-DC (5 wt%, 250 mg) in a 50 mL centrifuge tube and vortexed to promote homogeneous dispersion. The mixture was charged into a preheated micro-compounder (250–270 °C specified for polymer blends) under nitrogen and melt-mixed for 3 min before extrusion of the polymer sample.

Reactive Extrusion of Polymer Blends

Polymer blends were prepared at fixed weight ratios of 70/30 (HDPE/iPP) and 80/20 (HDPE/Nylon-6, HDPE/PET, LDPE/PET). Each blend (5.0 g total mass) was combined with PT-DC (5 wt%) and Irganox 1010 (10 mg), vortexed to promote homogeneous dispersion. Then the mixture was processed in a micro-compounder at 250 °C (270 °C for PET-containing blends). Materials were melt-mixed at 200 rpm for less than 3 min and extruded at 60 rpm.

Instrumentation and General Characterization

Nuclear magnetic resonance (NMR) spectra were recorded on a Bruker 400 MHz spectrometer at 298 K. Chemical shifts (δ) are reported in parts per million (ppm) relative to residual solvent signals ($CDCl_3$: δ = 7.26 ppm for ^1H NMR and 77.16 ppm for ^{13}C NMR). Signal multiplicities are reported as s (singlet), d (doublet), t (triplet), q (quartet), m (multiplet or unresolved), or br (broad), with coupling constants (*J*) given in Hz.

High-resolution mass spectrometry (HRMS) data were collected on a Bruker MaXis Plus UPLC-MS equipped with ESI and APCI sources.

Melt Processing and Film Preparation

Melt-extrusion and reactive blending were conducted using a Thermo Fisher Scientific HAAKE Minilab 3 micro-compounder (twin-screw extruder) operated in manual mode under a nitrogen atmosphere. Processing temperatures were selected based on polymer melting points, and screw speeds were maintained at 60- 200 rpm to ensure torque remained below 5 Nm.

Film processing was carried out by melt-compression molding using a Carver Auto Series Plus laboratory press (model 3889.1PL1000, maximum force 15 t). Polymer samples were sandwiched between non-stick Teflon sheets in a stainless-steel mold and compressed at 190 °C under 5,000 psi for 8 min. Samples were cooled under pressure using air and water circulation prior to further testing.

Tensile Analysis

Tensile stress/strain tests were performed on ASTM D-638 Standard Type-V tensile bar (dog-bones; $w = 3.18$ mm; $t = 0.8$ mm) specimens on an Instron 5966 (TA Instruments) universal testing system equipped with a 10-kN load cell and operated at room temperature. Selected samples were tightly placed between textured grips with a grip separation of about 28.5 mm and pulled at a constant strain rate of 5 mm min^{-1} until break.

Rheological and Mechanical Characterization

Dynamic mechanical analysis was performed on a Q800 DMA Analyzer (TA Instruments). Samples for DMA were prepared under similar conditions (w = 7.00–8.20 mm, t = 0.4–1.0 mm), with length measured within the grips by Q-series measurement software (TA Instruments) before each trial. Grips were judiciously tightened by three—in accordance with the sample's modulus. Temperatures below ambient were probed by a liquid nitrogen GCA tank attachment (TA Instruments).

Oscillatory shear rheology studies were performed on a Discovery Series Hybrid 2 (DHR-2) Rheometer (TA Instruments) at 180 °C. Circular discs (25 mm) of polymer films were loaded between two 25-mm steel parallel-plate EHP geometries under N2 (30 psi) gas flow unless otherwise noted. Rheometer control and data analysis were performed with TA Instruments TRIOS software.

Creep-recovery experiments were conducted under a constant axial force of 1 N for 180 s followed by a 180 s recovery period at fixed temperatures appropriate to each polymer system.

Frequency sweeps were performed at 0.5% strain over an angular frequency range of 0.1–100 rad s^{-1}.

Temperature sweeps were conducted on a Q800 DMA at a heating rate of 3 °C min^{-1}, and a fixed frequency of 1 Hz.

Gel Fraction Analysis

Crosslinked samples (30–100 mg) were placed and swollen in appropriate solvents for 16 h (HDPE: xylene, 120 °C; LDPE: toluene, 85 °C; Ternary blend (LDPE/HDPE/*i*PP): xylenes, 150 °C. The swollen gels were dried under reduced pressure at 100 °C to constant mass. Gel fraction was calculated as the ratio of the final dried mass to the initial mass.

Scanning Electron Microscopy

SEM imaging was performed on a JEOL IT800HL microscope operating at 3 kV. Samples were sputter-coated with 15 nm chromium and imaged at multiple magnifications. Image analysis was conducted using ImageJ2.

Stress Relaxation

Stress relaxation experiments were performed on a DHR-2 rheometer using 25 mm parallel plates. Samples were equilibrated at the test temperature for 5 min, strained to 3%, and monitored for 10,000 s.

Differential Scanning Calorimetry

DSC was performed on dried samples (3–5 mg for polymers and ~1.0 mg for DCs) on an Auto Q20 in aluminum pans with hermetic lids against an identical, empty reference pan/lid (TA Instruments). The oven was constantly flushed with 40.00 ml/min of N_2. DC sample analysis (40–250 °C) was performed on the first heating scan, where single-event exothermic signals for carbene formation were recorded at a heating rate of 5 °C/min. On the other hand, DSC plots for polymer species show the second heating scan and first cooling scan (−40 °C or −50 °C to 200 °C). Initial (first) heating scans were operated at a ramp rate of 10 °C/min, followed by cooling at 10 °C/min and a second heating scan at 10 °C/min. Thermal transitions by DSC were characterized by Universal Analysis (TA Instruments). Explosivity and shock sensitivity values for DCs were obtained by DSC measurements and the correlation calculations according to literature procedures.[1]

Thermogravimetric Analysis

Thermogravimetric analysis was performed on DCs and dried polymer samples (5–10 mg) on a Q50 TGA Analyzer (TA Instruments). Polymer samples were placed in a weight-tared aluminum pan and heated from 25 °C to 700 °C at a rate of 10 °C/min. A constant flow of N_2 was flushed through the furnace at 40.00 ml/min and mass loss was quantified as a percentage of initial mass with increased temperature. Thermal stability and weight loss measurements were analyzed by Universal Analysis (TA Instruments).

PT-DC Preparation

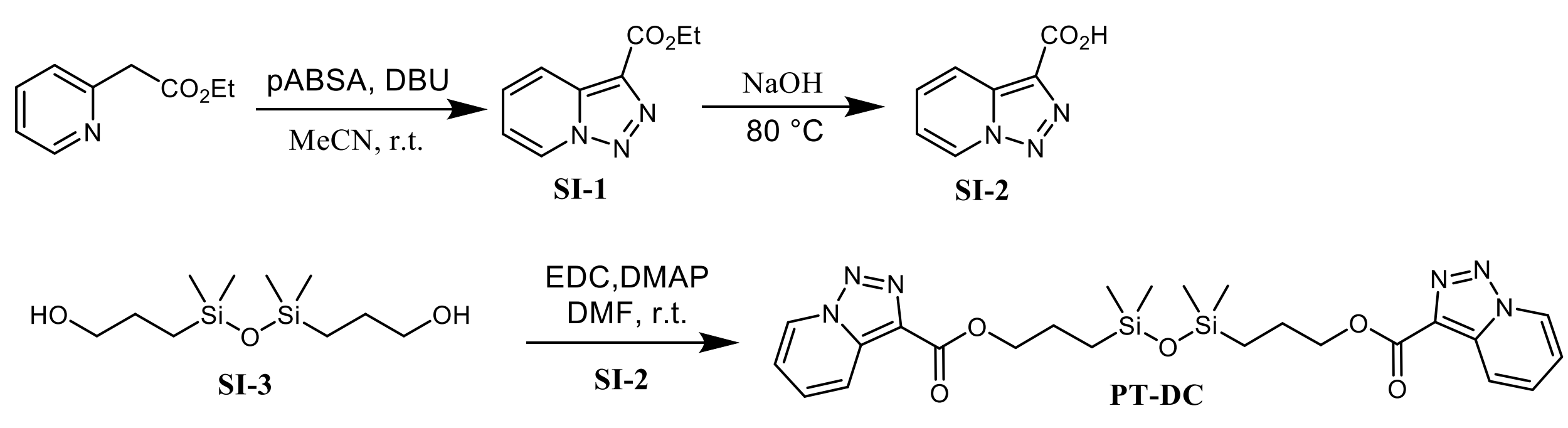

Scheme S1. Synthetic procedure of PT-DC.

Internal Pyridine Catalysis

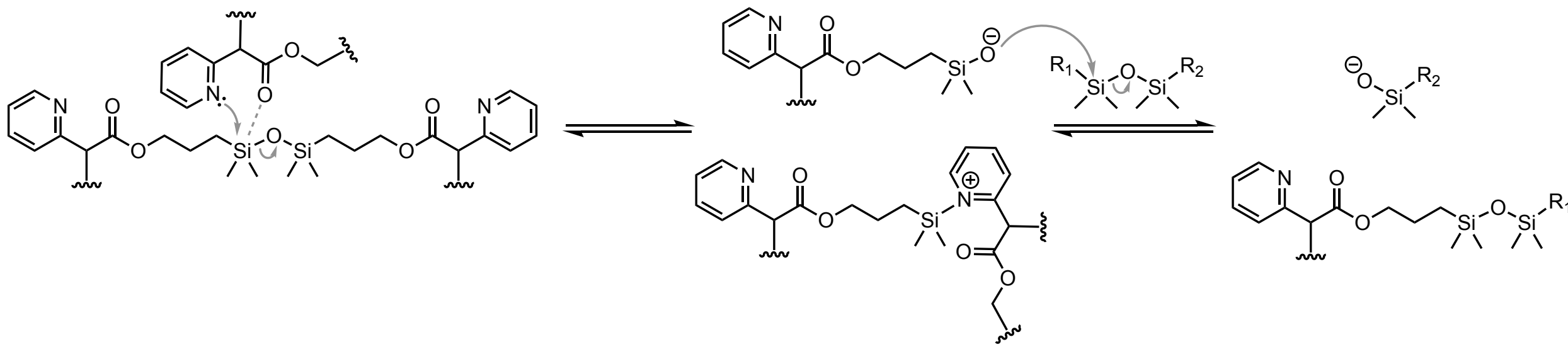

Scheme S2. Proposed intermolecular mechanism for internally catalyzed siloxane exchange in the PT-DC system. A possible intramolecular pathway involving nucleophilic attack of the electrophilic Si center by the pyridyl nitrogen on the same molecule is not shown.

Synthesis of PT-DC crosslinker

Ethyl 2-(pyridin-2-yl)acetate (1.0 equiv, 18.7 g, 113.2 mmol) and DBU (1.4 equiv, 23.6 mL, 158.5 mmol) were dissolved in anhydrous acetonitrile (300 mL) at room temperature and placed under a nitrogen atmosphere. Next, pABSA (1.2 equiv, 32.6 g, 135.8 mmol) was slowly added to the solution. The mixture was stirred for 16 h. After the reaction was completed, the mixture was concentrated in a rotary evaporator. The residue was dissolved in ethyl acetate and washed with saturated aqueous NH_4Cl solution. The organic layer was further washed with brine and then dried on anhydrous Na_2SO_4. The solution was concentrated in vacuo and subjected to flash chromatography (SiO_2, hexane/EtOAc = 1:1). Pure **SI-1** was isolated in 96% overall yield (20.8 g).

SI-1 (1.0 equiv, 3.8 g, 20 mmol) was dissolved in 10% NaOH aqueous solution (20 mL) and heated at 80 °C to obtain a clear solution. The mixture was then cooled to room temperature. 2 M HCl was added to the mixture to tune the pH to 2. The precipitate was collected by filtration and washed with deionized water. The obtained white solid was dried in a vacuum oven at 60 °C for three days to obtain **SI-2** in quantitative yield.

A round-bottomed flask was charged with allyl alcohol (2.0 equiv, 7.8 mL, 113.6 mmol), Karstedt's catalyst (80 ppm), and 40 mL of toluene and stirred at ambient temperature for 5 min after which time 1,1,3,3-tetramethyldisiloxane (TMDS, 10.0 mL, 56.6 mmol) was added and the reaction was refluxed for 17 h. After the reaction was completed, the mixture was passed through a silica pad and eluted with hexane. The solvent was removed in vacuo to yield **SI-3** as a clear colorless liquid, which was directly used in the next step.

The crude diol **SI-3** (1.0 equiv, 1.61 g, 7.5 mmol), **SI-2** (2.0 equiv, 2.45 g, 15 mmol), and DMAP (2 equiv, 1.83 g, 15 mmol) were dissolved in anhydrous DMF (100 mL). EDC (3 equiv, 4.31 g, 22.5 mmol) was slowly added to the solution. The mixture was stirred at room temperature. A clear solution was obtained after ~ 2 h. Then the solution was stirred for 16 h. After the reaction was completed, water was added, and the mixture was extracted with ethyl acetate three times. The organic layer was collected and washed with 1 M HCl, water, saturated Na_2CO_3 aqueous

solution, and brine. The organic layer was dried on Na_2SO_4, filtered, and then concentrated in a rotary evaporator. The mixture was subjected to flash chromatography (SiO_2, hexane/EtOAc = 1:1 – 1:3). Pure **PT-DC** was isolated in 59% yield (2.23 g).

PT-DC: ^{1}H NMR (400 MHz, $CDCl_3$) δ 8.82 (dt, *J* = 7.0, 1.1 Hz, 2H), 8.26 (dt, *J* = 8.9, 1.2 Hz, 2H), 7.54 (ddd, *J* = 8.9, 6.8, 1.0 Hz, 2H), 7.14 (td, *J* = 6.9, 1.3 Hz, 2H), 4.41 (t, *J* = 8.0 Hz, 4H), 1.94 – 1.80 (m, 4H), 0.67 – 0.61 (m, 4H), 0.10 (s, 12H). ^{13}C NMR (100 MHz, $CDCl_3$) δ 161.6 (*C*O), 135.3 (*C*), 130.0 (*C*), 129.2 (*C*), 126.0 (*C*), 119.5 (*C*), 116.5 (*C*), 67.7 (CH_2), 23.0 (CH_2), 14.3 (CH_2), 0.4 (CH_3). HRMS-ESI (*m*/*z*): $[M+Na]^+$ calcd for $C_{24}H_{32}N_6O_5Si_2Na$, 563.1870; found, 563.1833.

Synthesis of model compounds Si-Dec and Si-Hept

Crude **SI-3** (1 equiv, 1 g, 4 mmol), heptanoic acid (2 equiv, 1.04 g, 8 mmol), and DMAP (0.1 equiv, 48.9 mg, 0.4 mmol) were added to a two-necked flask, dissolved in anhydrous DCM (80 mL), and the reaction flask was added to an ice bath. DCC (2 equiv, 1.65 g, 8 mmol) was dissolved in 10 mL of anhydrous DCM in a scint vial and added dropwise. After the addition of the DCC, a white precipitate formed in the solution, and the reaction was continued for 16 h. After the reaction was completed, the solution was placed in a dry ice/acetone bath to precipitate the residual DCU byproduct and filtered through a silica pad. The resulting filtrate was collected and concentrated in a rotary evaporator. The residual oil was dissolved in hexane (100 mL) and cooled in a dry ice/acetone bath. The solution was again filtered over a silica pad to retrieve the corresponding siloxane diester. Pure **Si-Hept** was isolated in 53% yield (1.01 g).

The same procedure was used to synthesize **Si-Dec**.

(Si-Hept): ^{1}H NMR (400 MHz, $CDCl_3$) δ 4.02 (t, *J* = 7.0 Hz, 3H), 2.30 (t, *J* = 7.5 Hz, 3H), 1.62 (dq, *J* = 14.5, 7.2 Hz, 6H), 1.43 – 1.18 (m, 9H), 0.93 – 0.77 (m, 4H), 0.60 – 0.46 (m, 2H), 0.07 (d, *J* = 7.9 Hz, 9H). ^{13}C NMR (101 MHz, $CDCl_3$) δ 174.40(C), 77.68 (CH_2), 67.16 (CH_2), 34.87(CH_2), 31.93(CH_2), 29.30(CH_2), 25.46(CH_2), 23.04(CH_2) (d, *J* = 17.9 Hz), 14.56 (CH_3) (d, *J* = 15.1 Hz), 0.63 (CH_3) (d, *J* = 14.5 Hz).

(Si-Dec): ^{1}H NMR (400 MHz, $CDCl_3$) δ 4.02 (t, *J* = 7.0 Hz, 1H), 2.29 (t, *J* = 7.5 Hz, 1H), 1.68 – 1.51 (m, 2H), 1.27 (d, *J* = 9.6 Hz, 7H), 0.87 (t, *J* = 6.6 Hz, 2H), 0.71 – 0.35 (m, 1H), 0.06 (s, 4H). ^{13}C NMR (101 MHz, $CDCl_3$) δ 174.41(*C*), 77.80(CH_2), 77.48(CH_2), 77.16(CH_2), 67.16(CH_2), 34.87(CH_2), 32.33(CH_2), 30.15 – 28.11 (CH_2) (m), 25.51(CH_2), 23.13(CH_2), 14.60 (CH_3) (d, *J* = 6.9 Hz), 0.71(CH_3).

Characterization

NMR

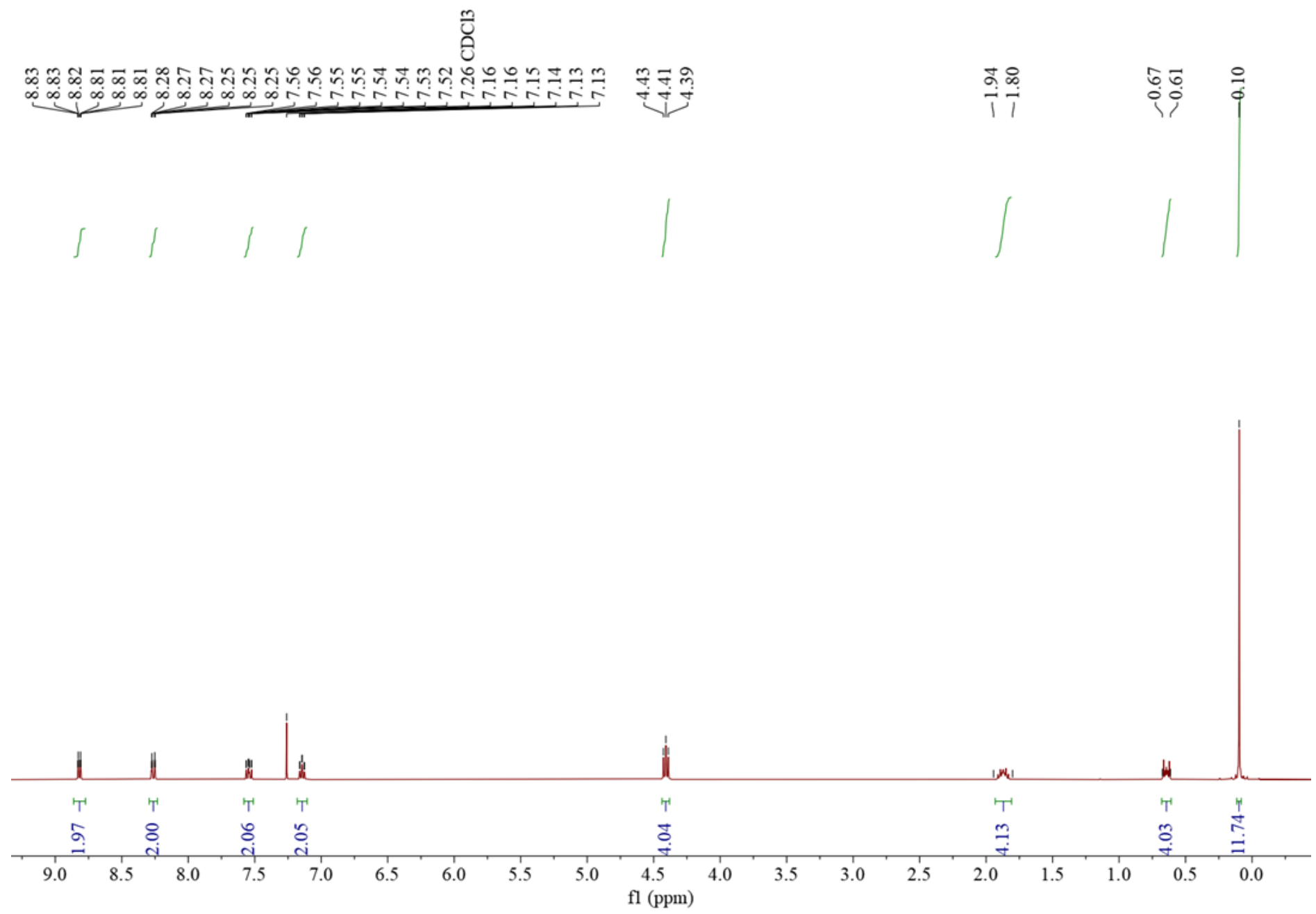

Figure S1. ^{1}H NMR ($CDCl_3$, 23 °C) spectra of PT-DC.

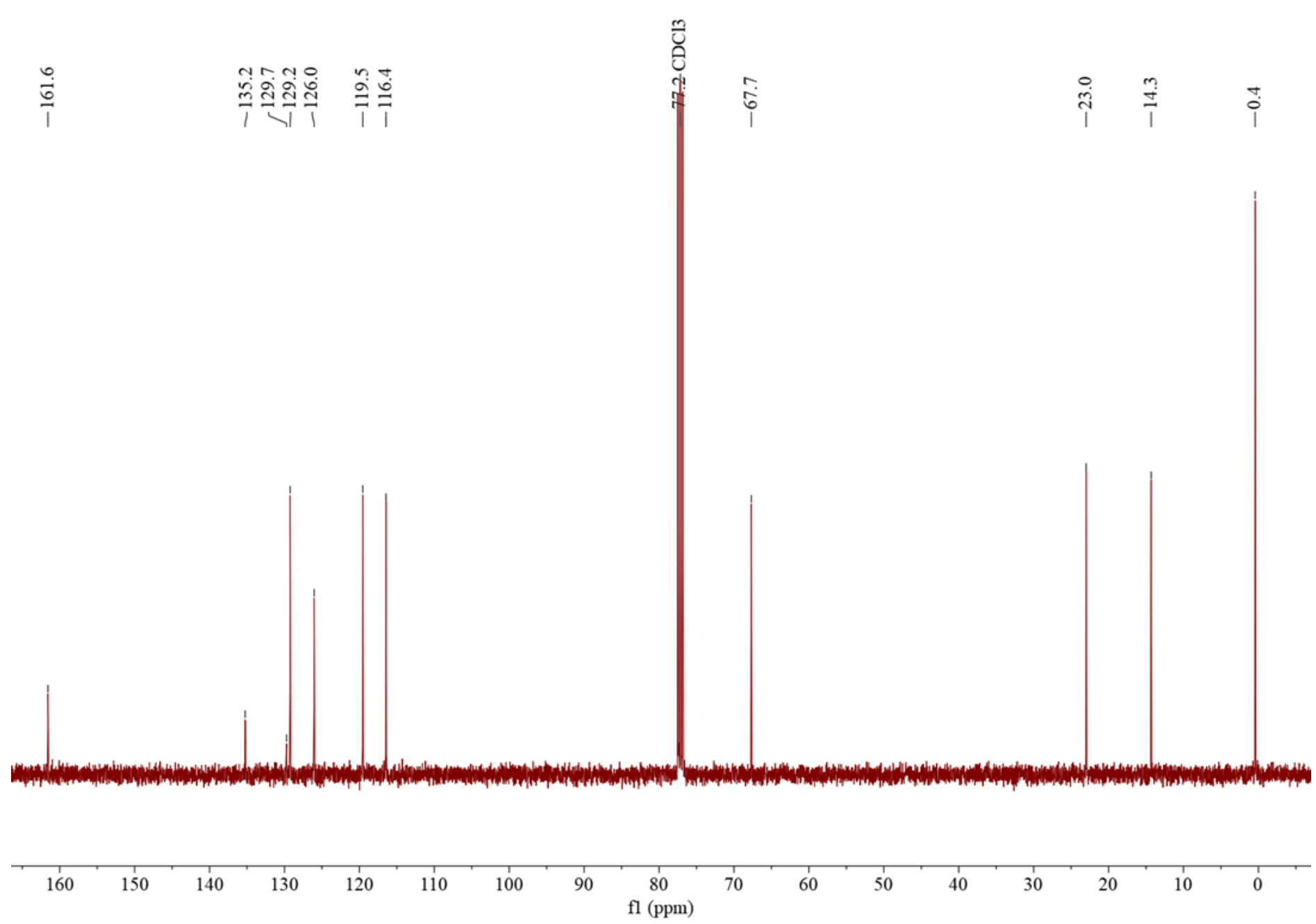

Figure S2. ^{13}C NMR ($CDCl_3$, 23 °C) spectra of the PT-DC.

High-Resolution Mass Spectrometry

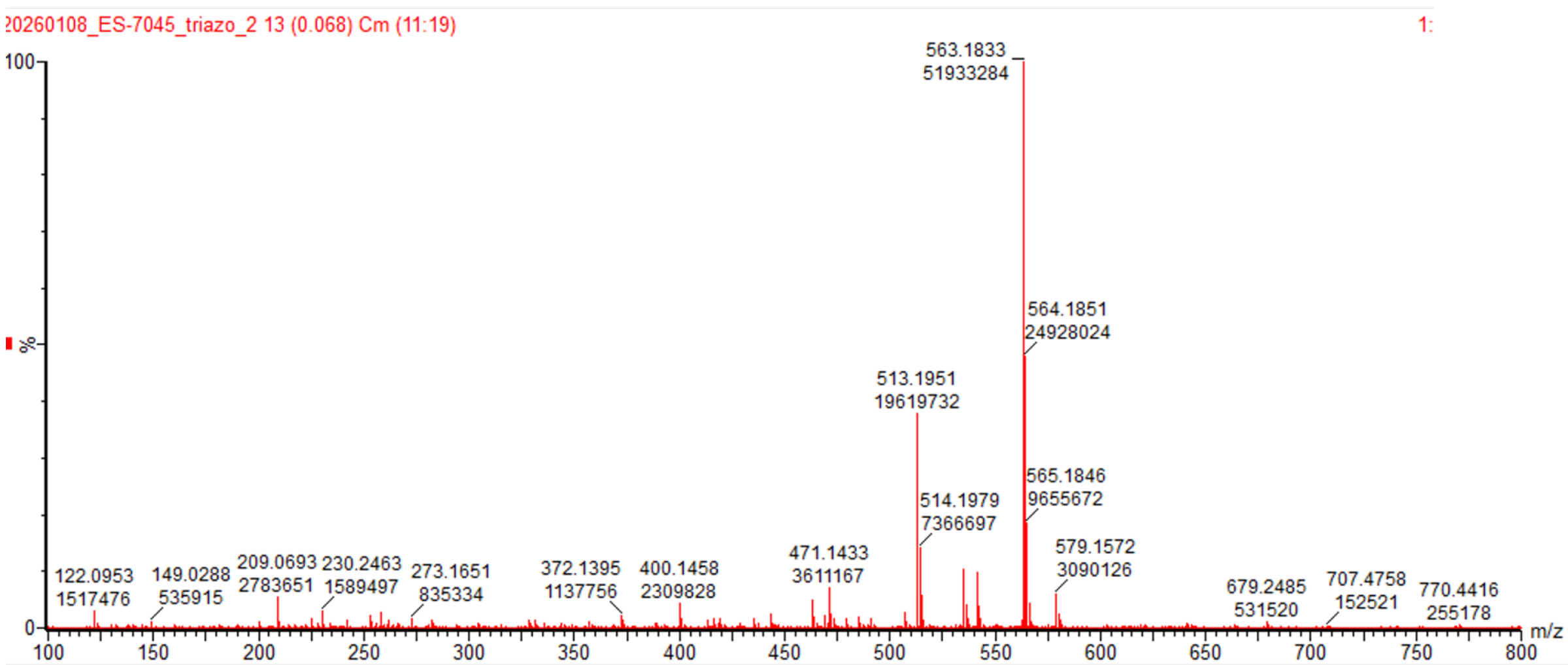

Figure S3. HRMS spectra of PT-DC. [M + Na+] in positive mode:

Theoretical - 563.1870 (*m/z*)

Observed - 563.1833 (*m/z*)

Crosslinker Characterization (Yoshida Correlation)

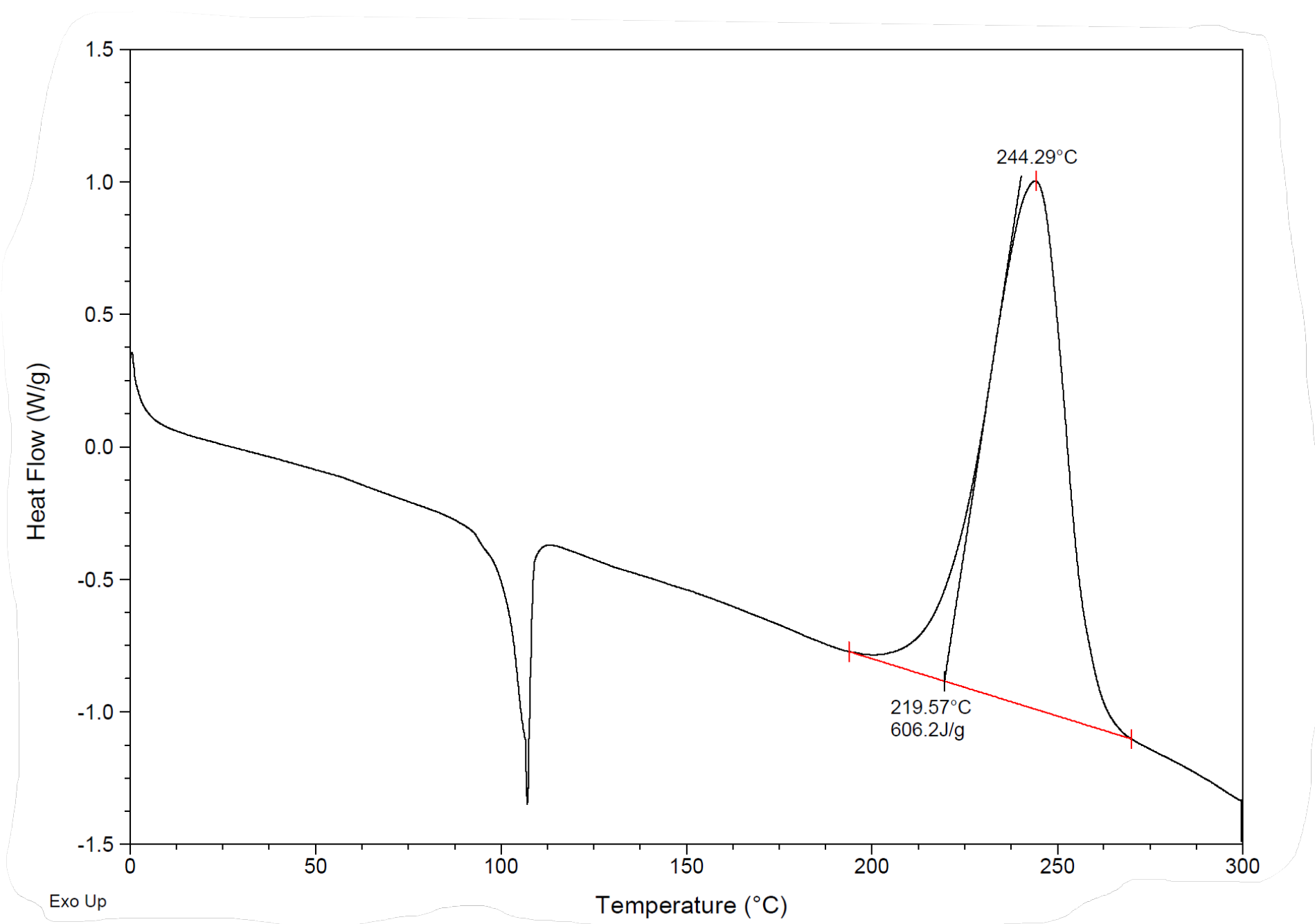

Figure S4. DSC trace of PT-DC. Sample Size 0.6 mg: 0 ˚C – 300 ˚C with 10 ˚C min^{-1} increase, Tzero pan, hermetic lid (pinholed).

$$\text{Shock Sensitivity (SS)} = \log(Q_{DSC}) - 0.72 \times \log(T_{onset} - 25\ °C) - 0.98$$

$$\text{Explosive propagation (EP)} = \log(Q_{DSC}) - 0.38 \times \log(T_{onset} - 25\ °C) - 1.67$$

$$SS_{(1)} = -0.47$$

$$EP_{(1)} = -0.38$$

SS < 0 indicates that the PT-DC is not shock sensitive. EP < 0 indicates no explosion propagation expected for this compound.

LC-MS Analysis

Procedure Agilent 6545 Q-TOF Mass Spectrometer:

The samples were dissolved in ACN prior to analysis. The instrument was equipped with an electrospray ionization (ESI) source, and samples were run in positive mode. The LC method employed a binary mobile phase consisting of A: H_2O/MeOH (9:1) containing 10 mM NH_4OAc and 0.2 mM NH_4F, and B: ACN/MeOH/IPA (2:3:5) containing 10 mM NH_4OAc and 0.2 mM NH_4F.

GC-MS Analysis

Data was acquired using an Agilent 7890B coupled to a Waters Xevo G2-XS QTOF with an APGC source. Corona current was set to 2.0uA, cone voltage set to 15v, source offset set to 30v, source temperature set to 150 °C. Cone gas set to 0L/h and aux gas set to 300L/h using nitrogen. The MSMS method included an MSe scan from 50-1200 m/z with a 0.2 second scan rate. High energy channel used a collision ramp of 6-30v. For lock mass, column bleed at 355.0705 m/z was used.

APGC was run in dry mode to produce M+ ions. Injections (1 uL) were made using pulsed splitless mode onto a DB-5MS column (30m x 0.25mm x 0.25umm). The GC inlet was held at 300 °C. The transfer line was set to 320 °C. Nitrogen was used as carrier gas and makeup gas with flow rates of 1.2mL/min and 200mL/min, respectively. The oven program was as follows:

Initial Temp (°C)	80	Initial Hold (min)	1
Rate (°C/min)	Final Temp (°C)	Hold Time (min)	Total Time (min)
20	230	1.5	10
15	320	3	19

Data Processing

GC-MS data was processed using Waters MassLynx software.

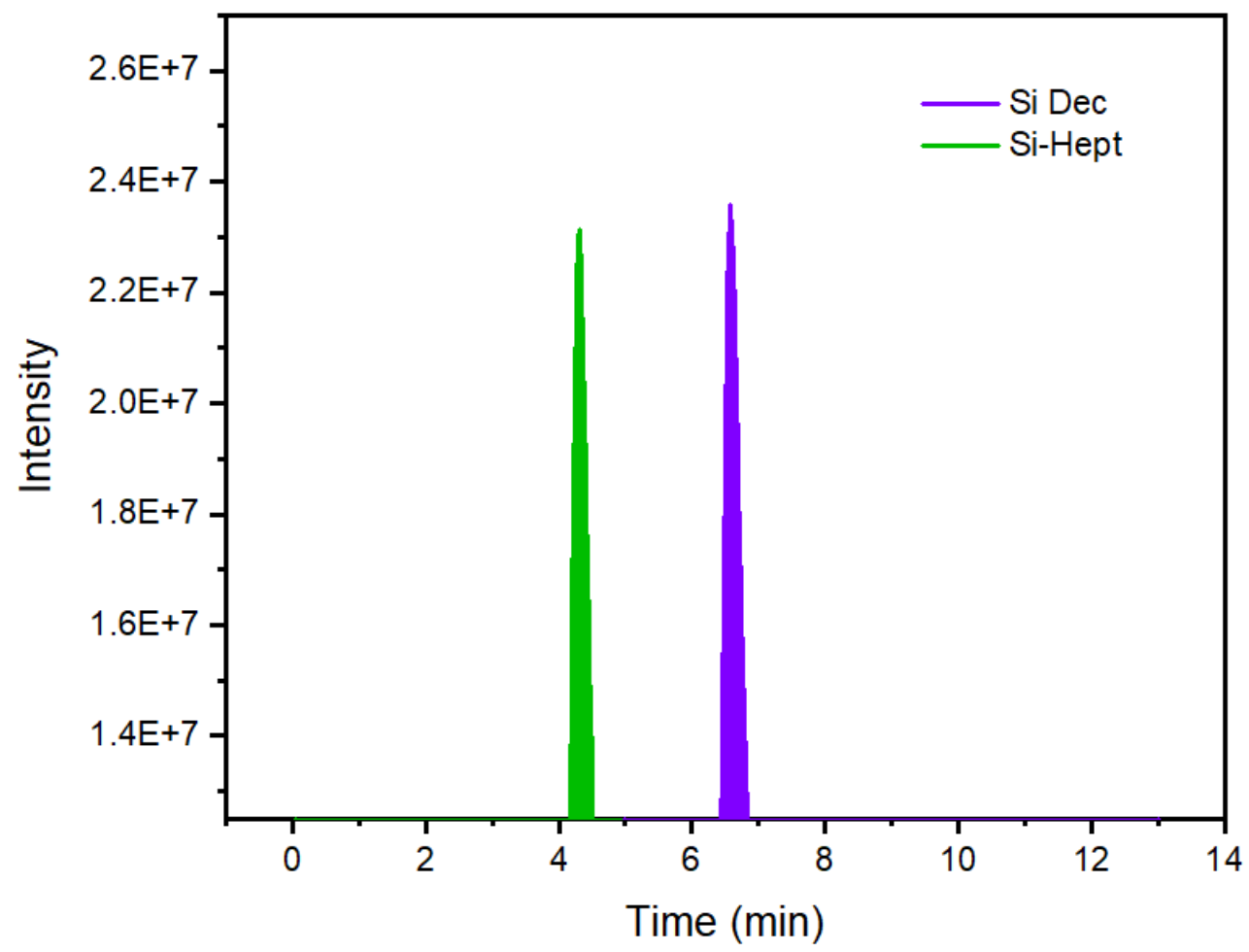

Figure S5. Elution peaks of the exchange experiment of Si-Dec and Si-Hept in the absence of pyridine catalyst.

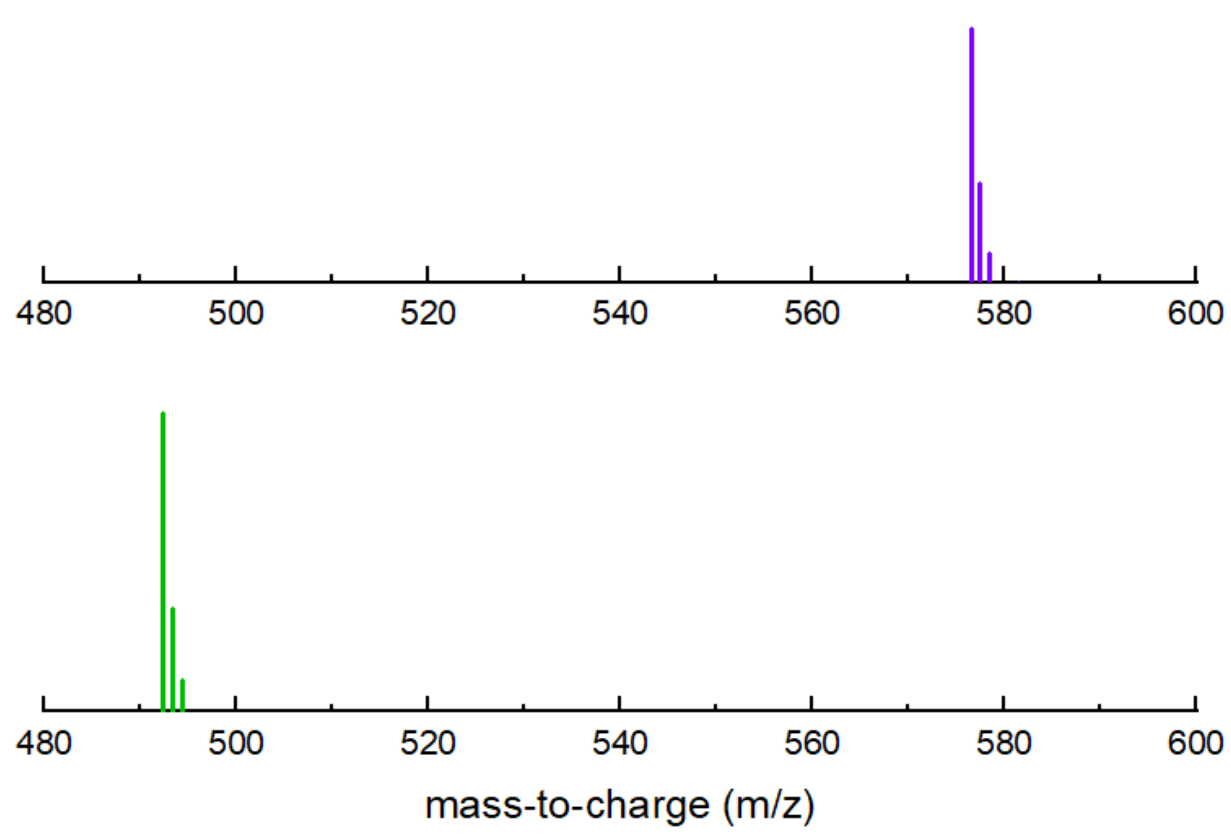

Figure S6. Corresponding mass-to-charge ratio from respective peaks for Si-Dec and Si-Hept [$M+NH_4$].

Characterization of Model Substrates

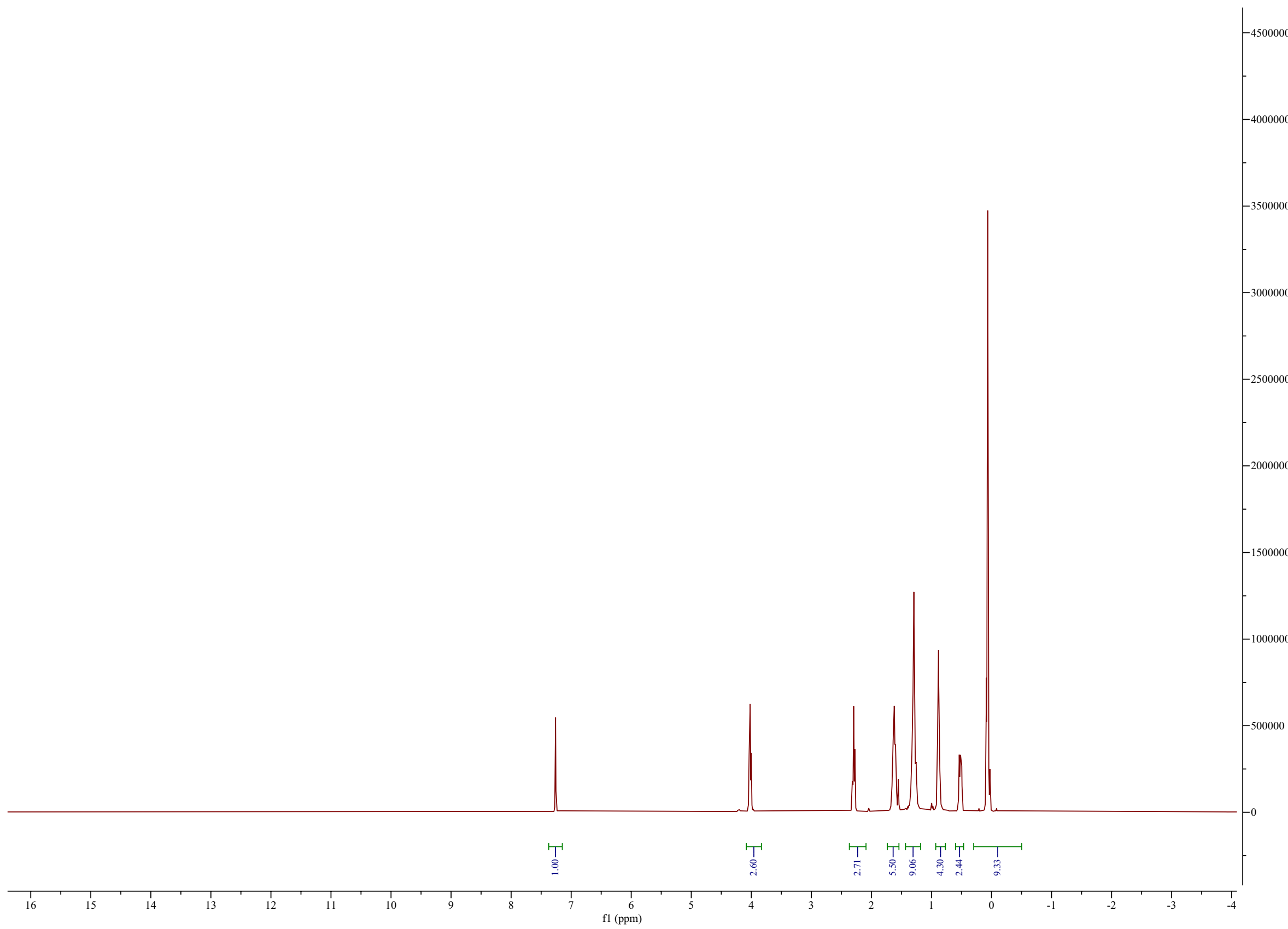

Figure S7. ^{1}H-NMR ($CDCl_3$, 23 °C) spectra of Si-Hept.

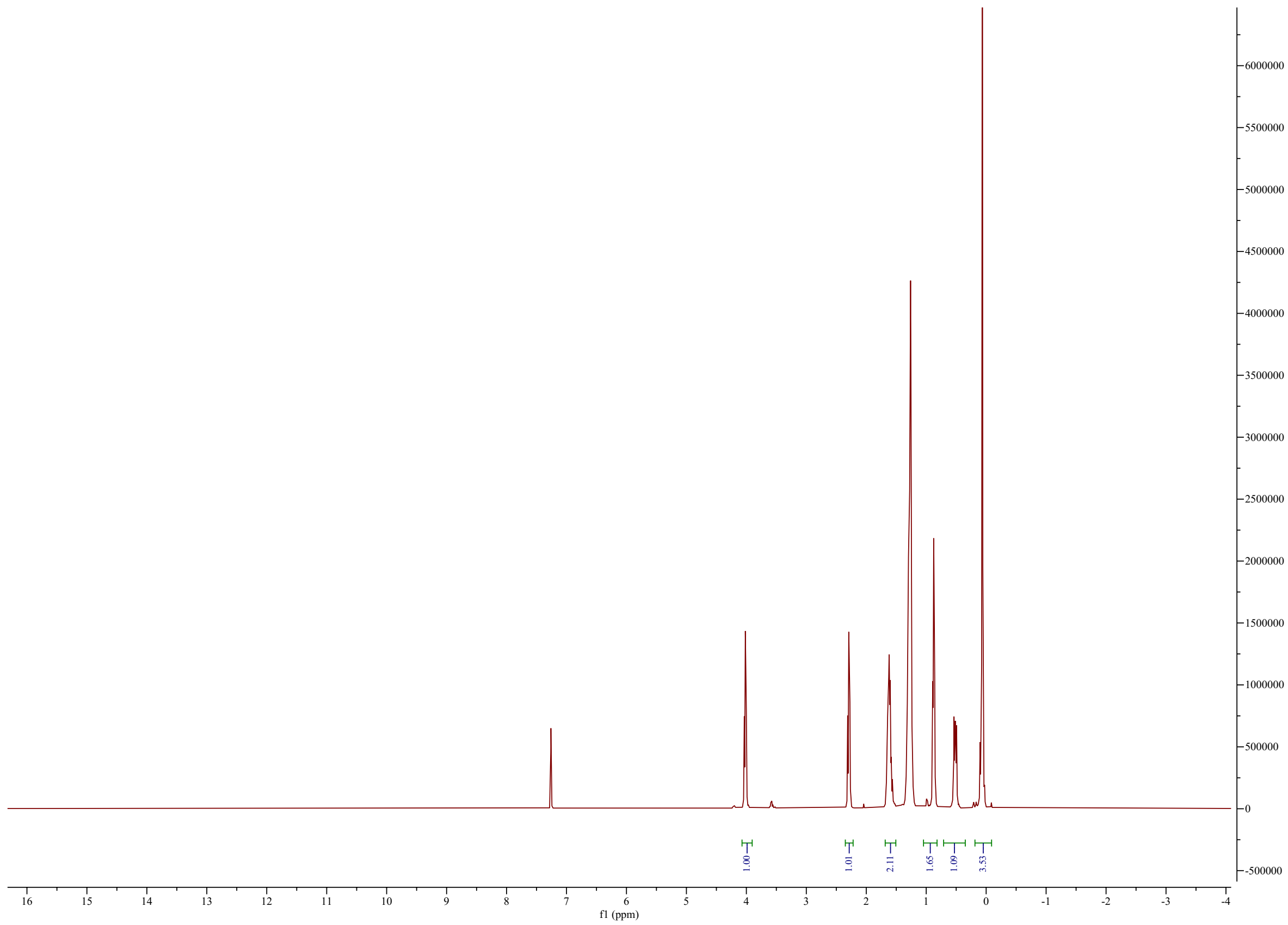

Figure S8. ^{1}H-NMR ($CDCl_3$, 23 °C) spectra of Si-Dec.

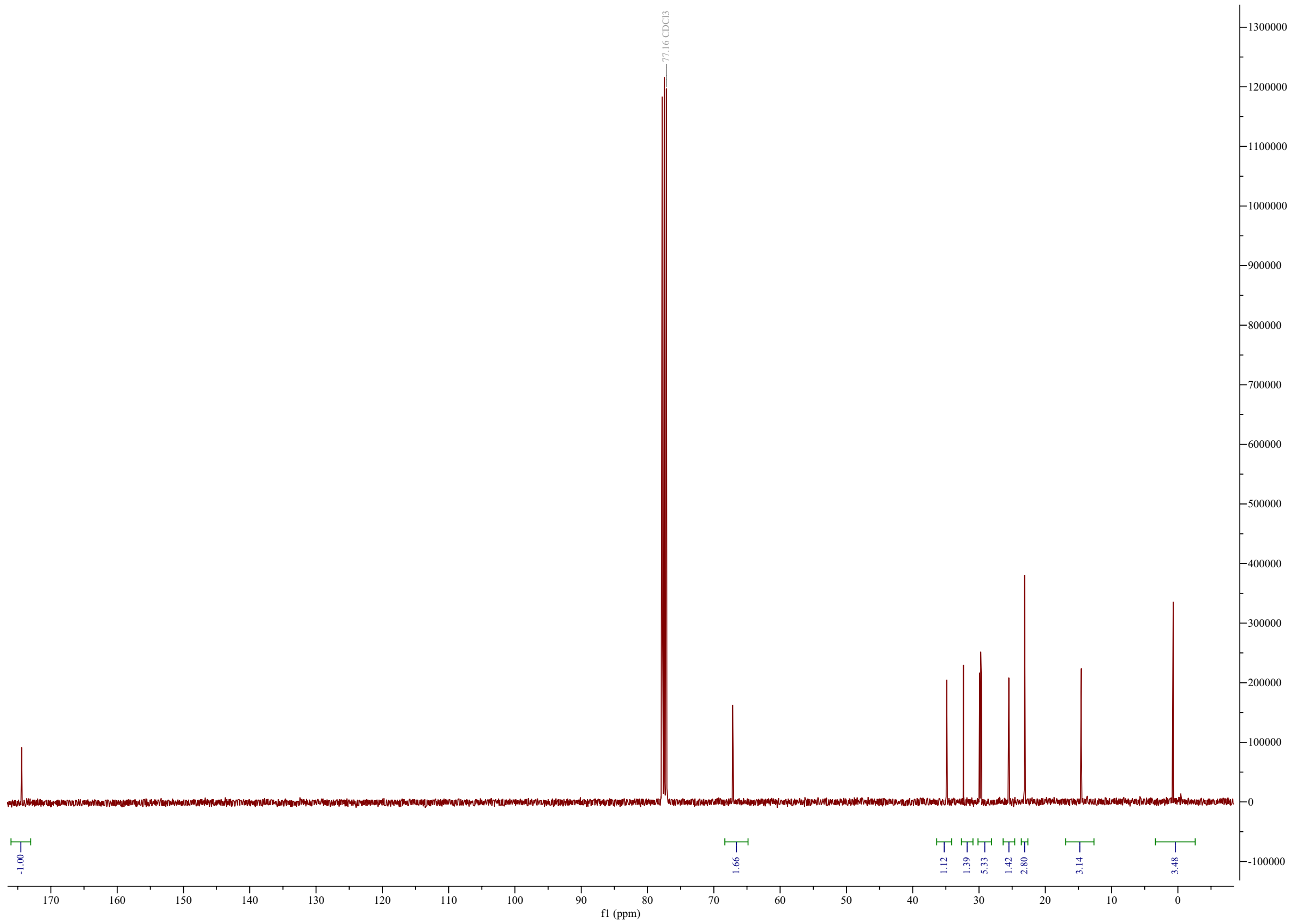

Figure S9. Si-Dec ^{13}C NMR ($CDCl_3$, 23 °C) spectra of Si-Dec.

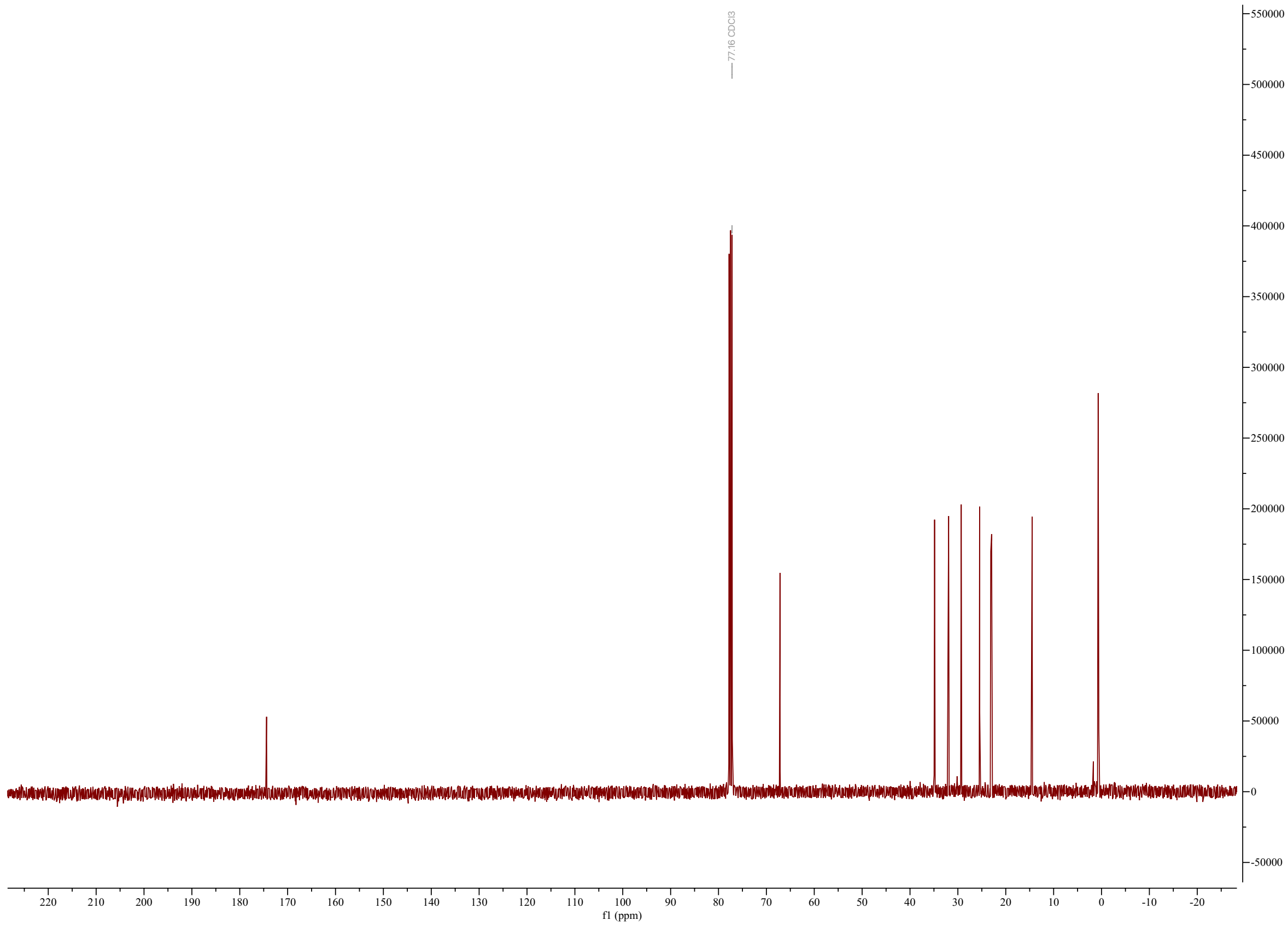

Figure S10. ^{13}C NMR ($CDCl_3$, 23 °C) spectra of Si-Hept.

Differential Scanning Calorimetry

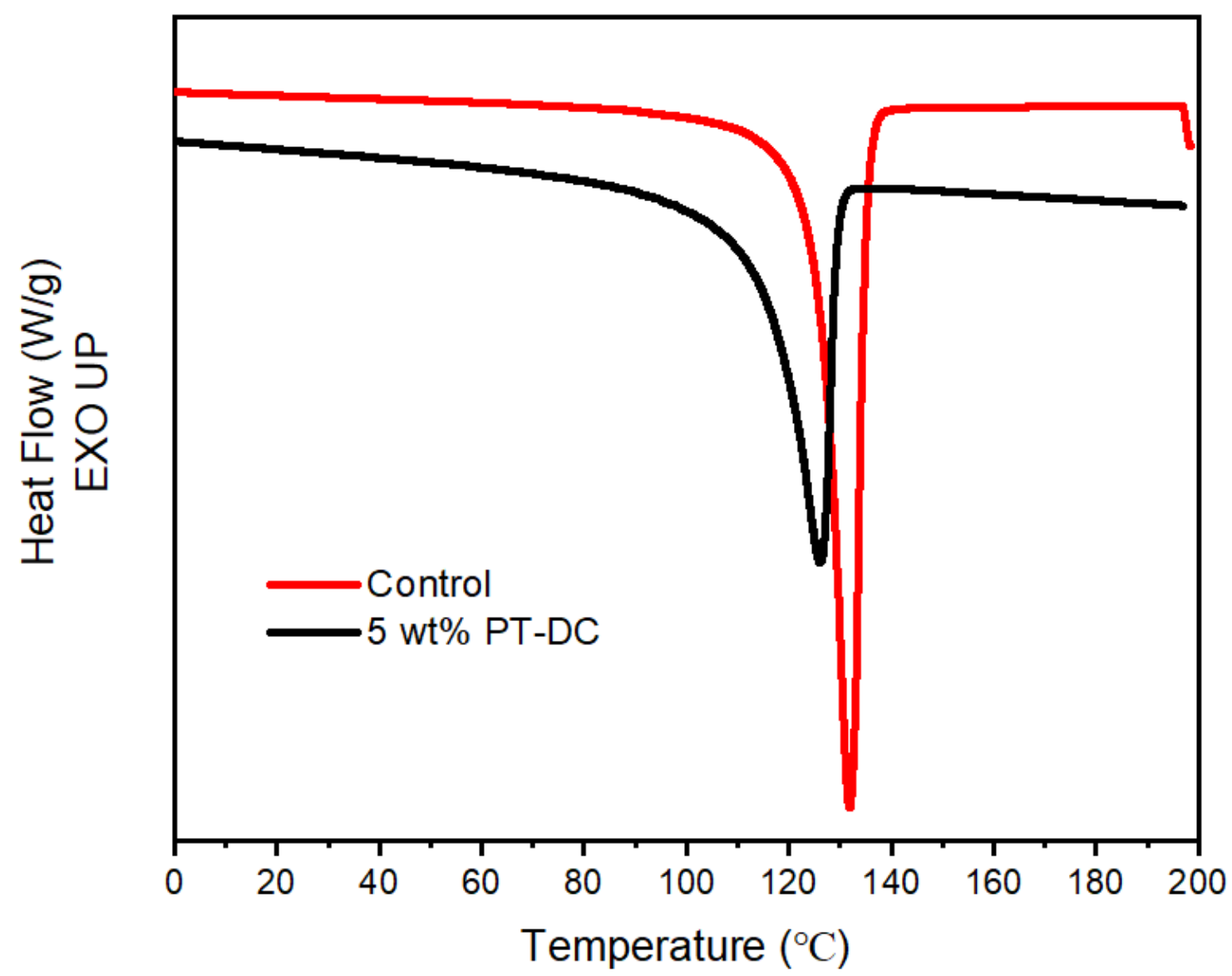

Figure S11. Differential scanning calorimetry of HDPE control (red) and HDPE with 5 wt% PT-DC (black).

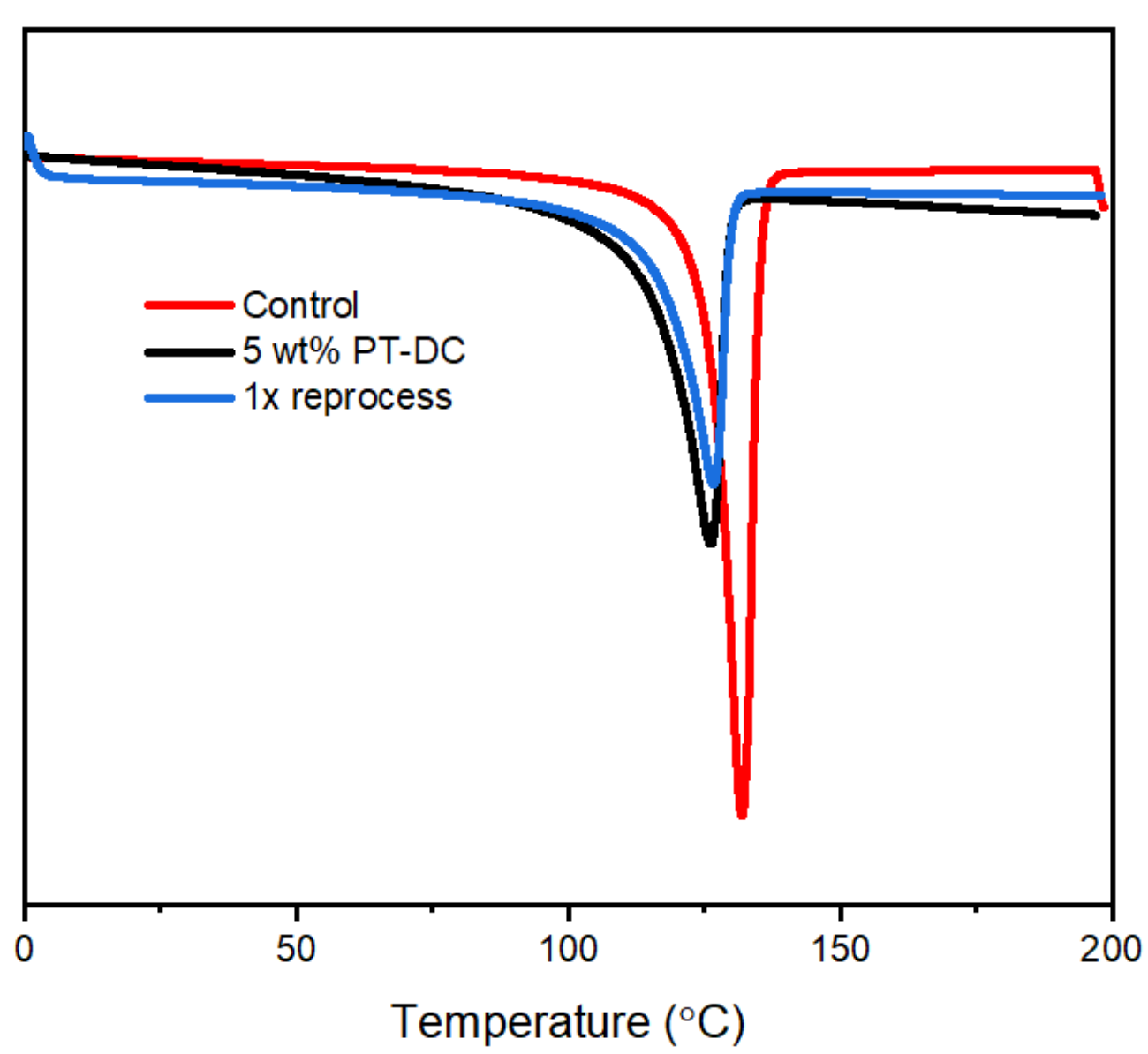

Figure S12. Overlay plot of DSC curves of HDPE control (red), HDPE treated with 5 wt% PT-DC (black), and HDPE with PT-DC after one reprocessing (blue).

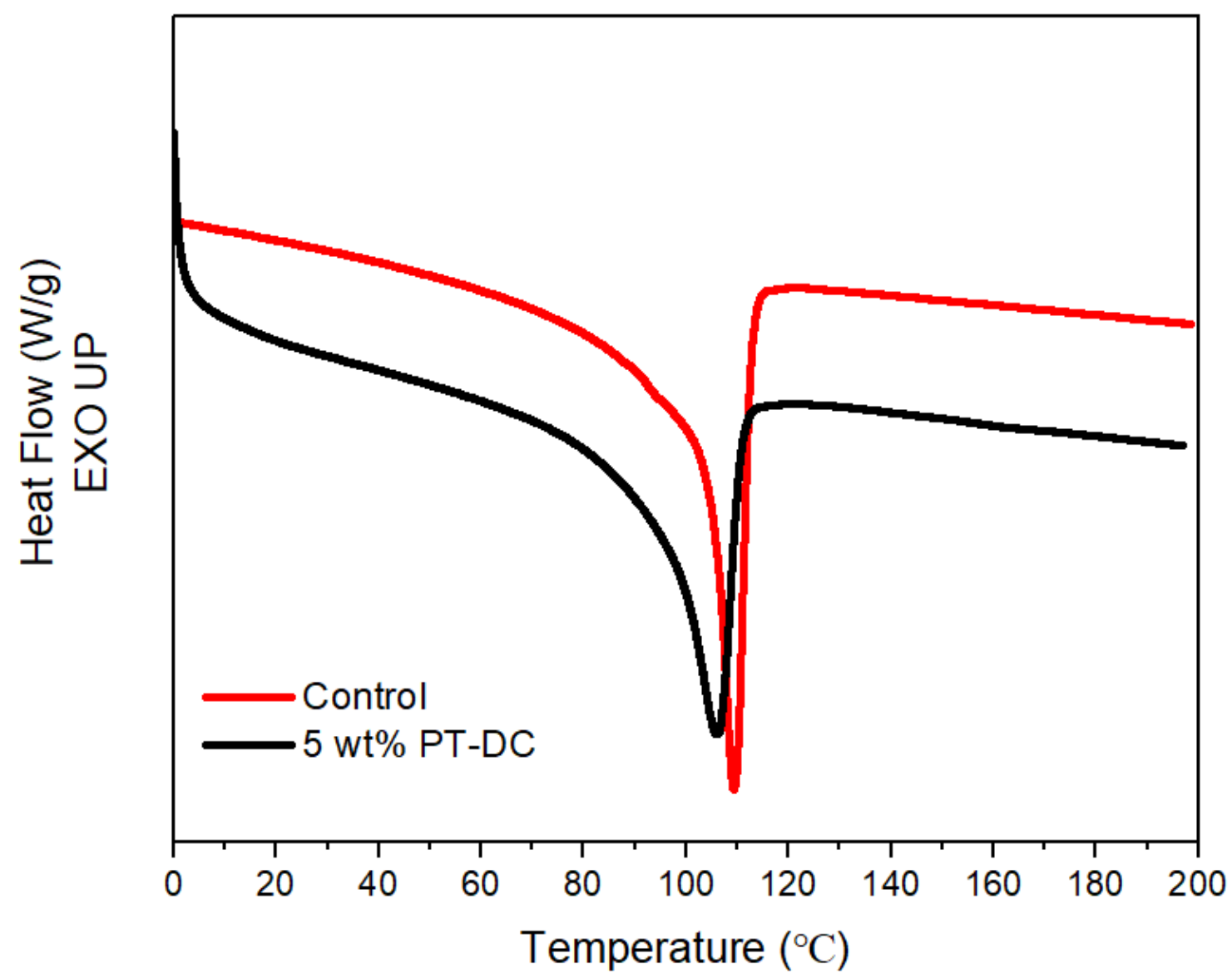

Figure S13. Differential scanning calorimetry of LDPE control (red) and LDPE with 5 wt% PT-DC (black).

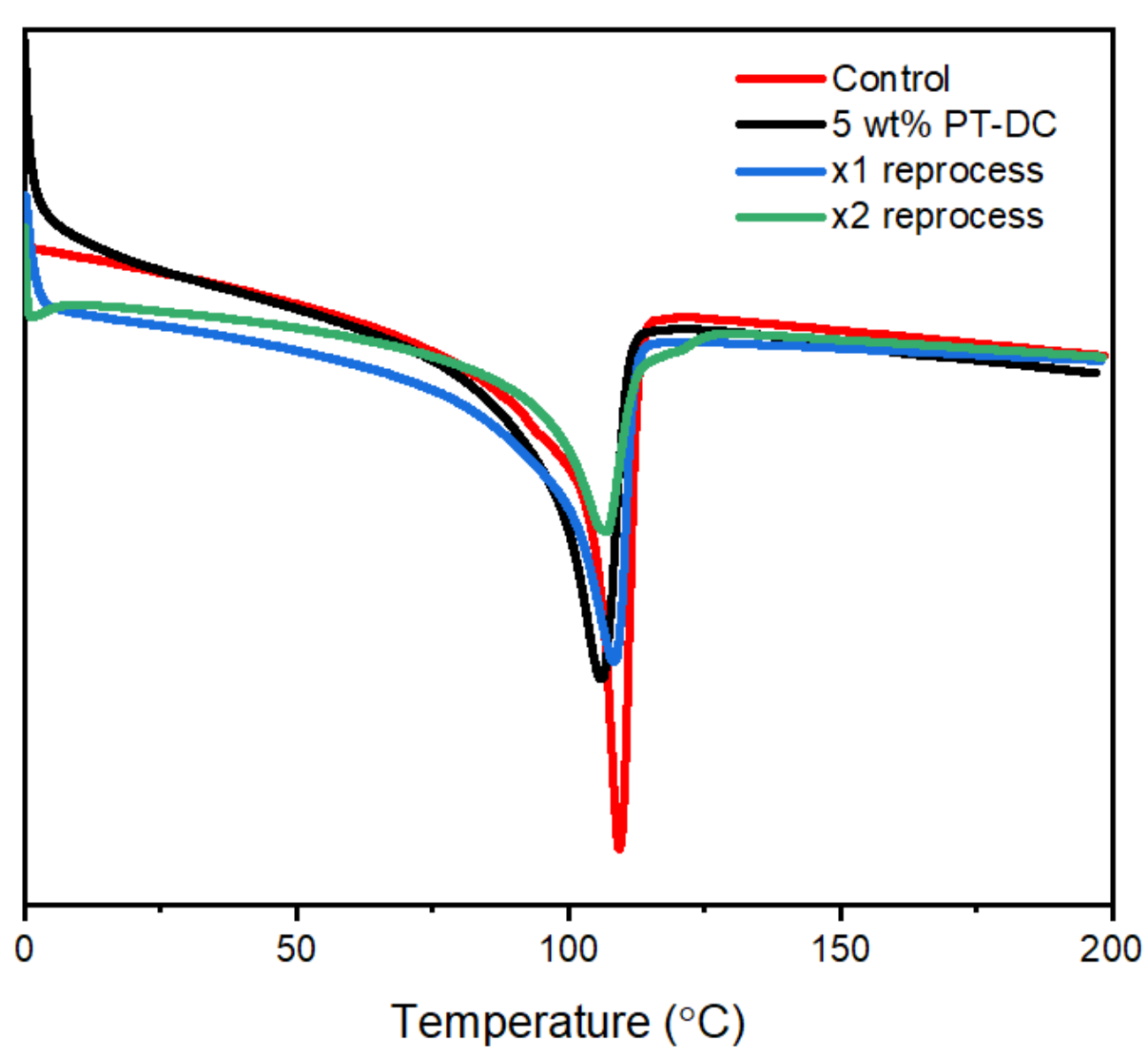

Figure S14. Overlay plot of DSC curves of LDPE control (red), LDPE treated with 5 wt% PT-DC (black), LDPE-DC after one reprocessing (blue), and LDPE-DC after two reprocessing cycles (green).

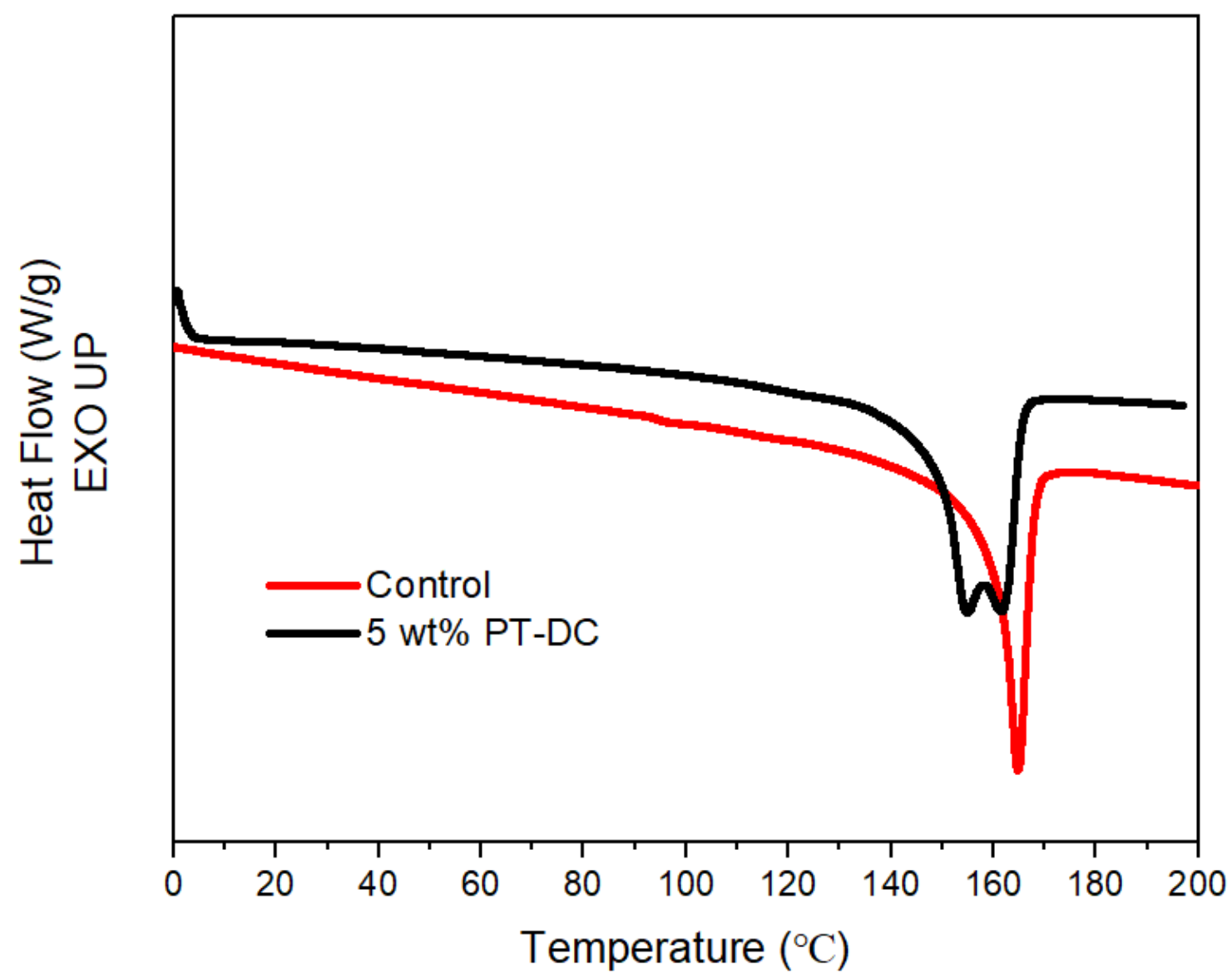

Figure S15. Differential scanning calorimetry of *i*PP control (red) and *i*PP with 5 wt% PT-DC (black).

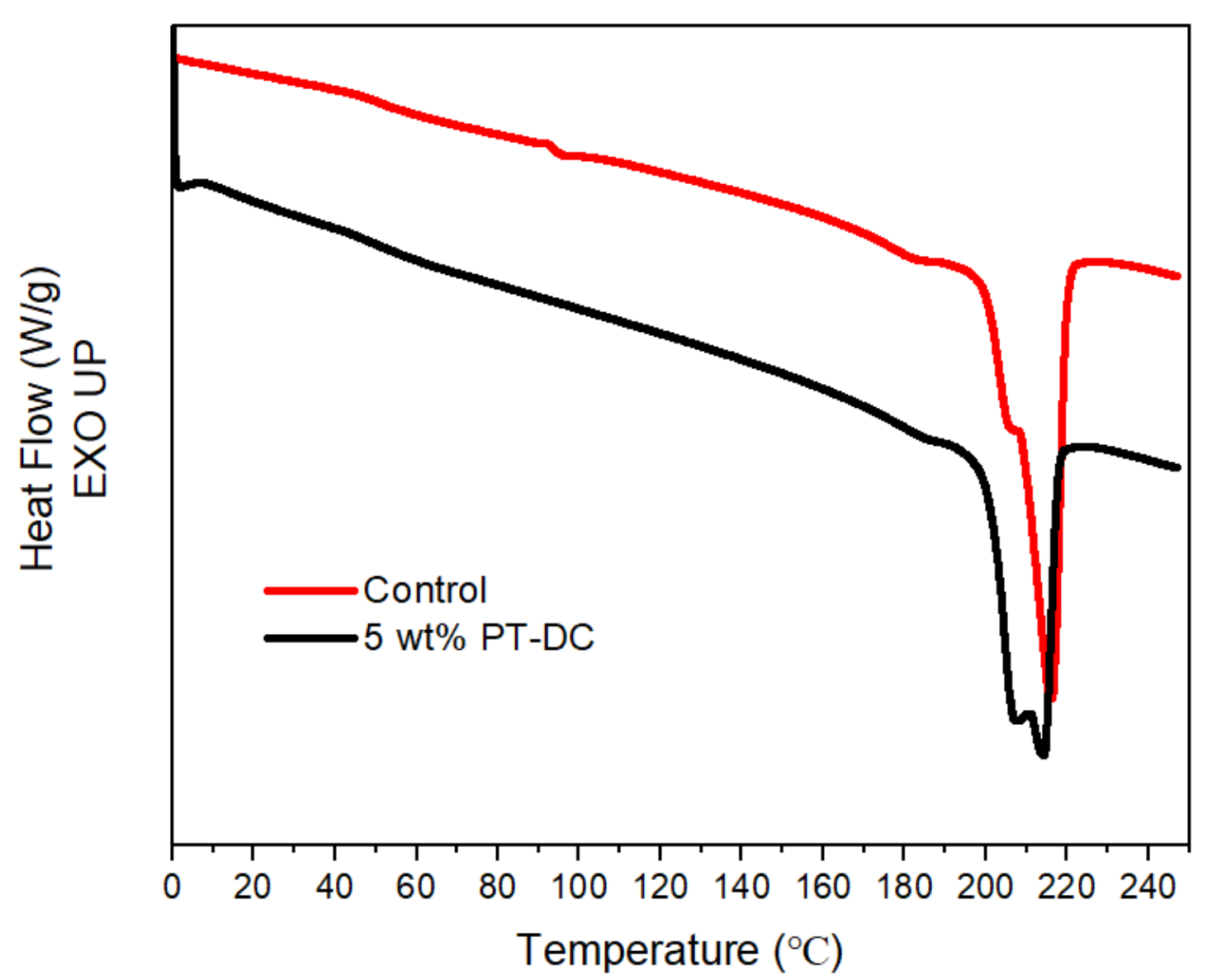

Figure S16. Differential scanning calorimetry of Nylon-6 control (red) and Nylon-6 with 5 wt% PT-DC (black).

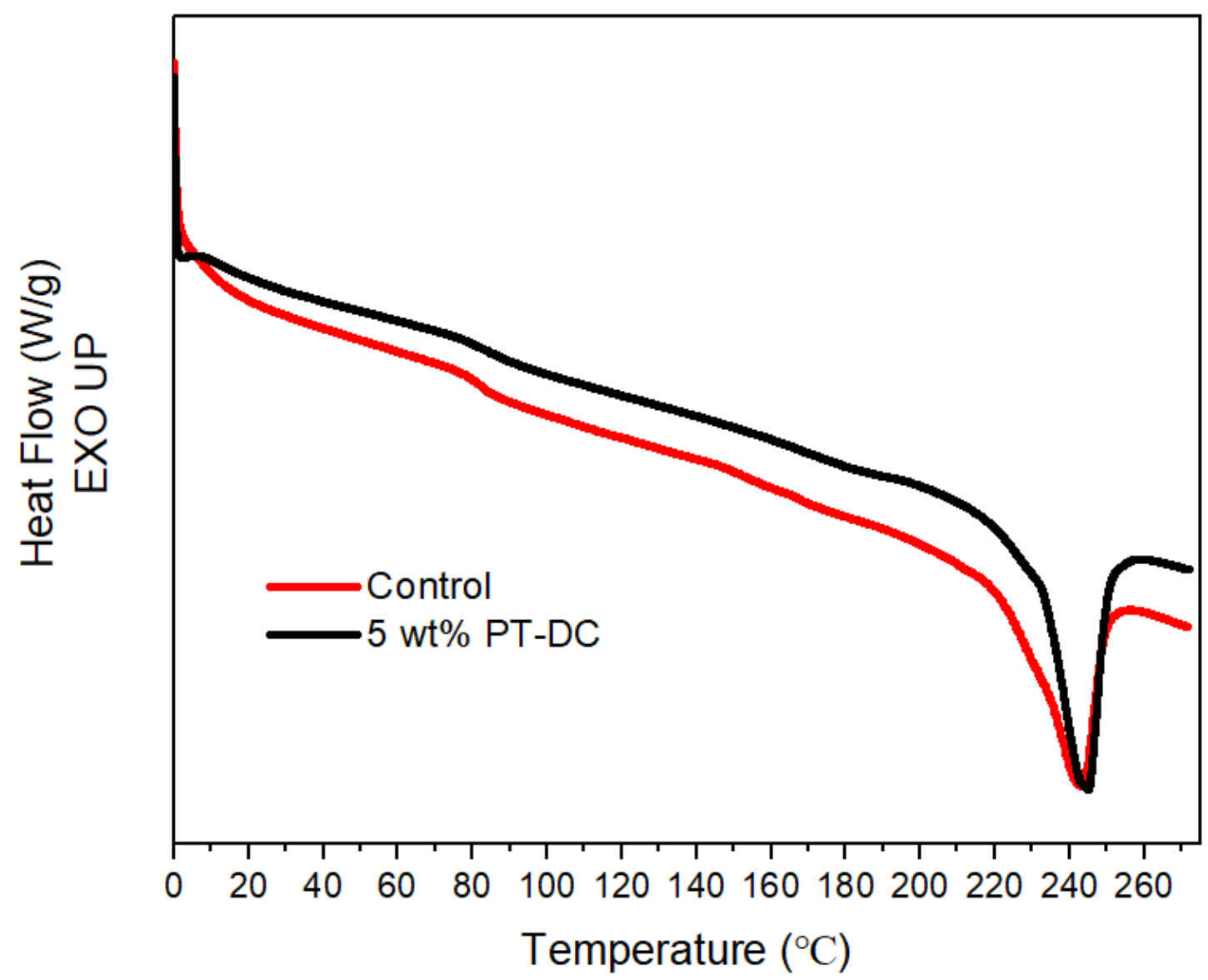

Figure S17. Differential scanning calorimetry of PET control (red) and PET with 5 wt% PT-DC (black).

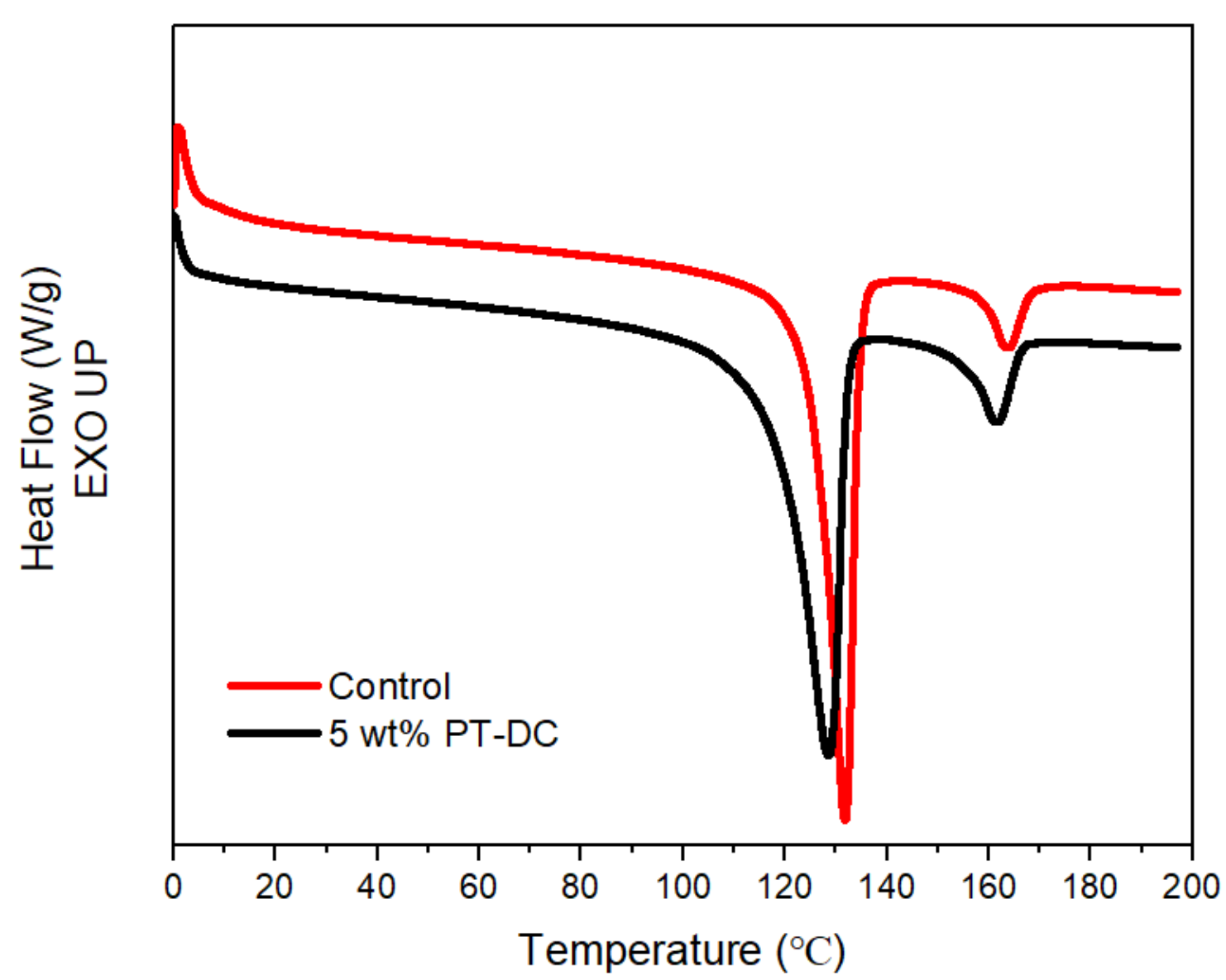

Figure S18. Differential scanning calorimetry of HDPE-*i*PP (70:30) control (red) and HDPE-*i*PP (70:30) with 5 wt% PT-DC (black).

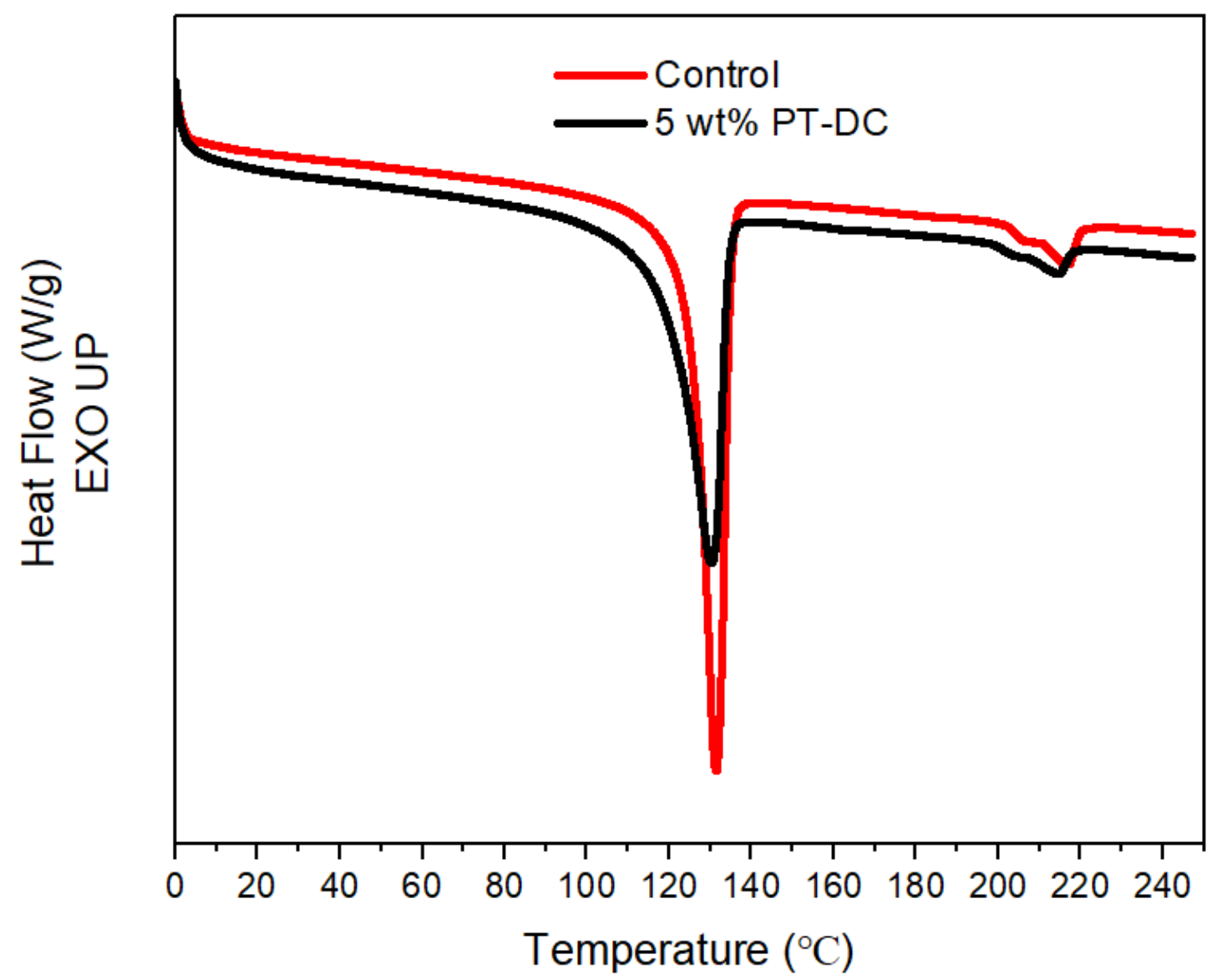

Figure S19. Differential scanning calorimetry of HDPE-Nylon6 (80:20) control (red) and HDPE-Nylon 6 (80:20) with 5 wt% PT-DC (black).

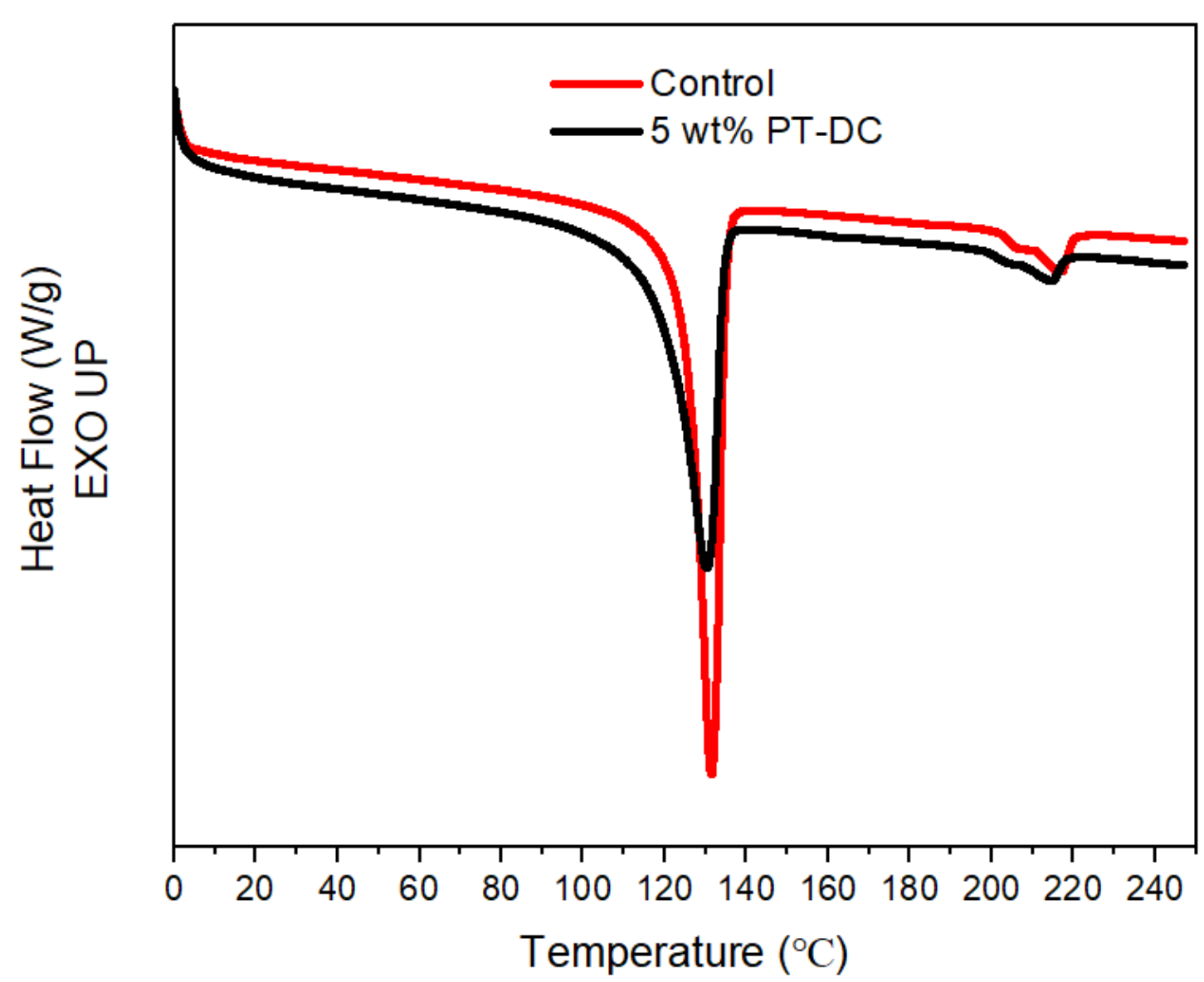

Figure S20. Differential scanning calorimetry of HDPE-PET (80:20) control (red) and LDPE-PET (80:20) with 5 wt% PT-DC (black).

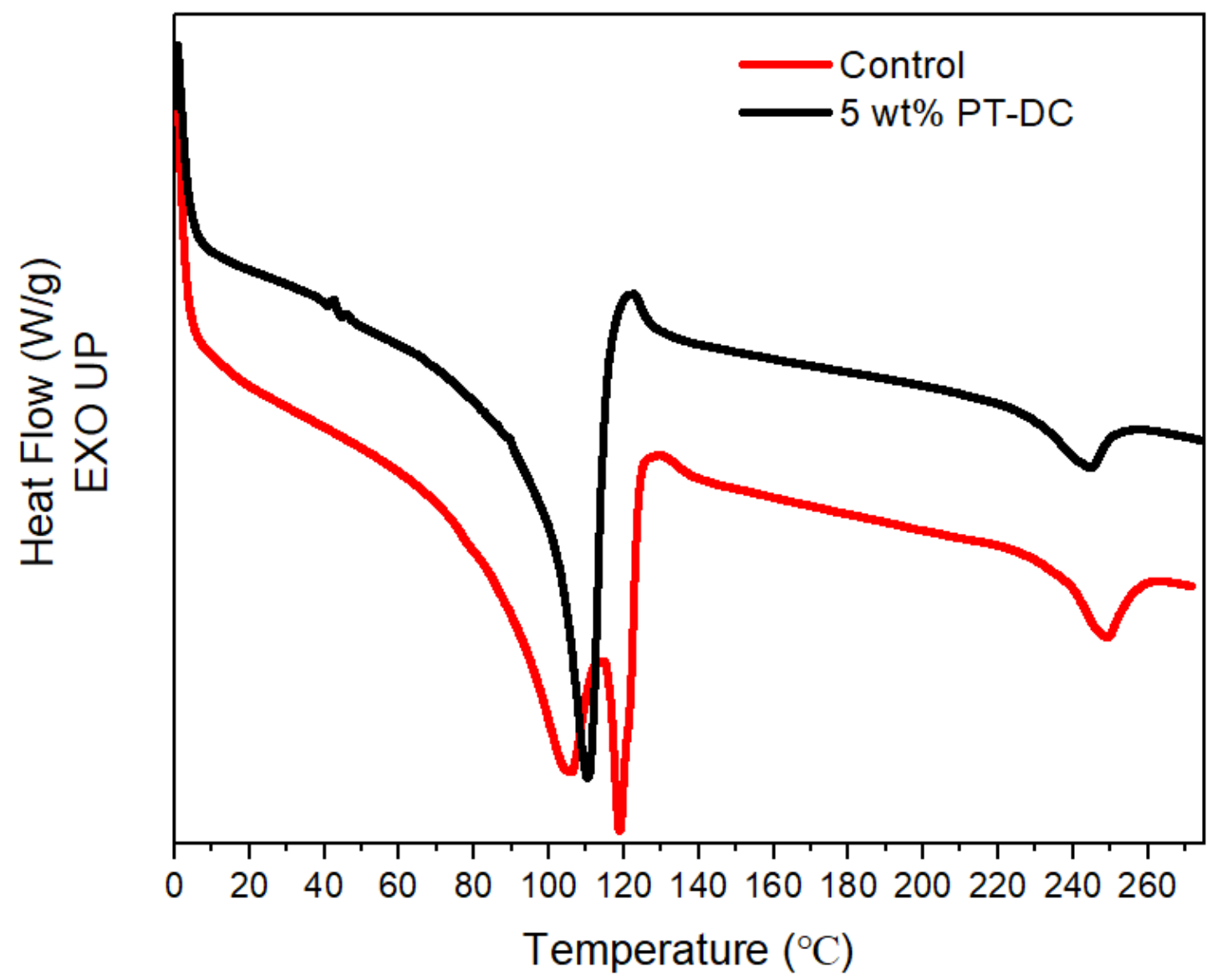

Figure S21. Differential scanning calorimetry of LDPE-PET (80:20) control (red) and LDPE-PET (80:20) with 5 wt% PT-DC (black).

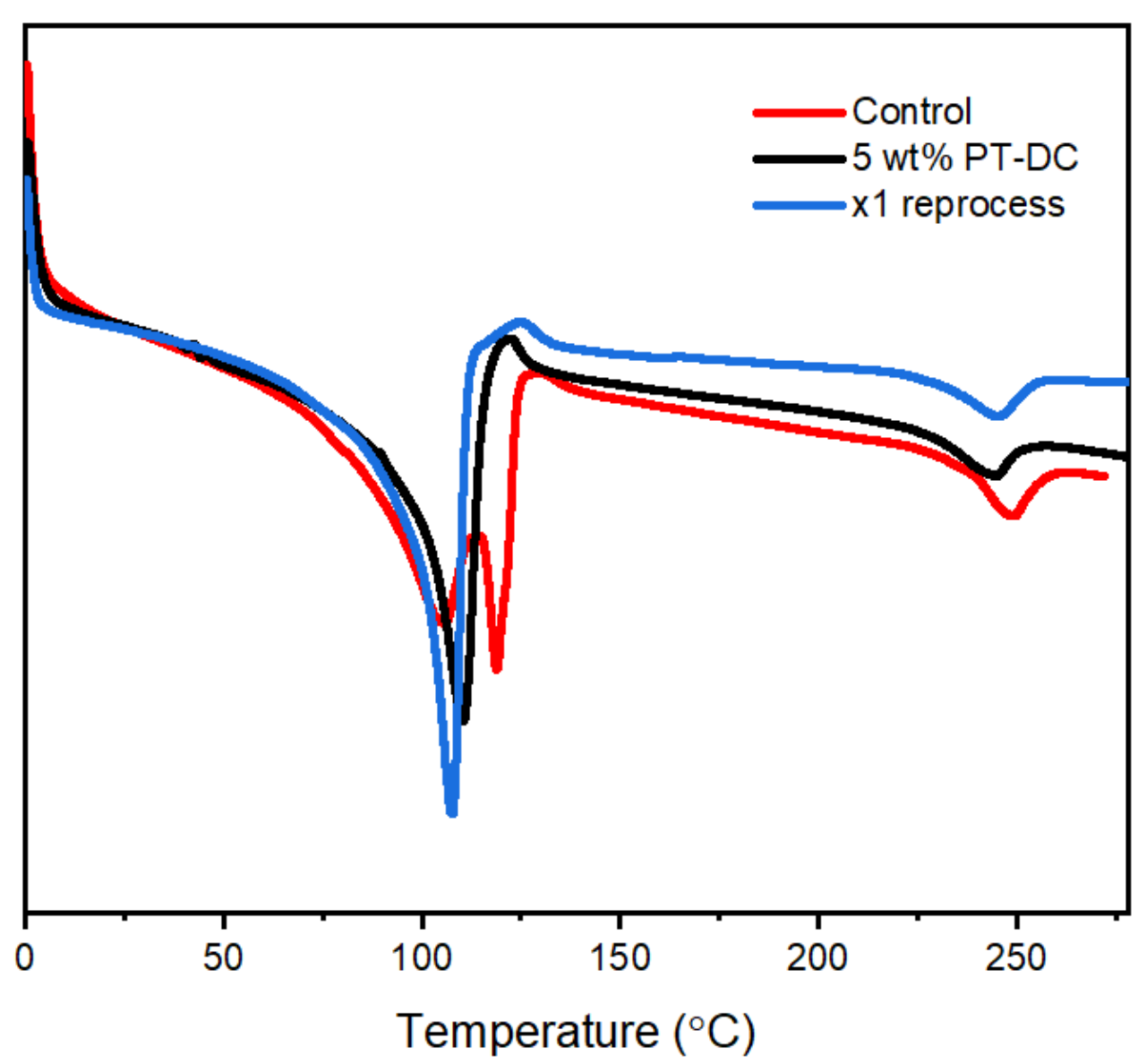

Figure S22. Overlay plot of DSC curves of LDPE-PET control (red), LDPE-PET blend compatibilized with 5 wt% PT-DC (black), and the compatibilized blend after one reprocessing (blue).

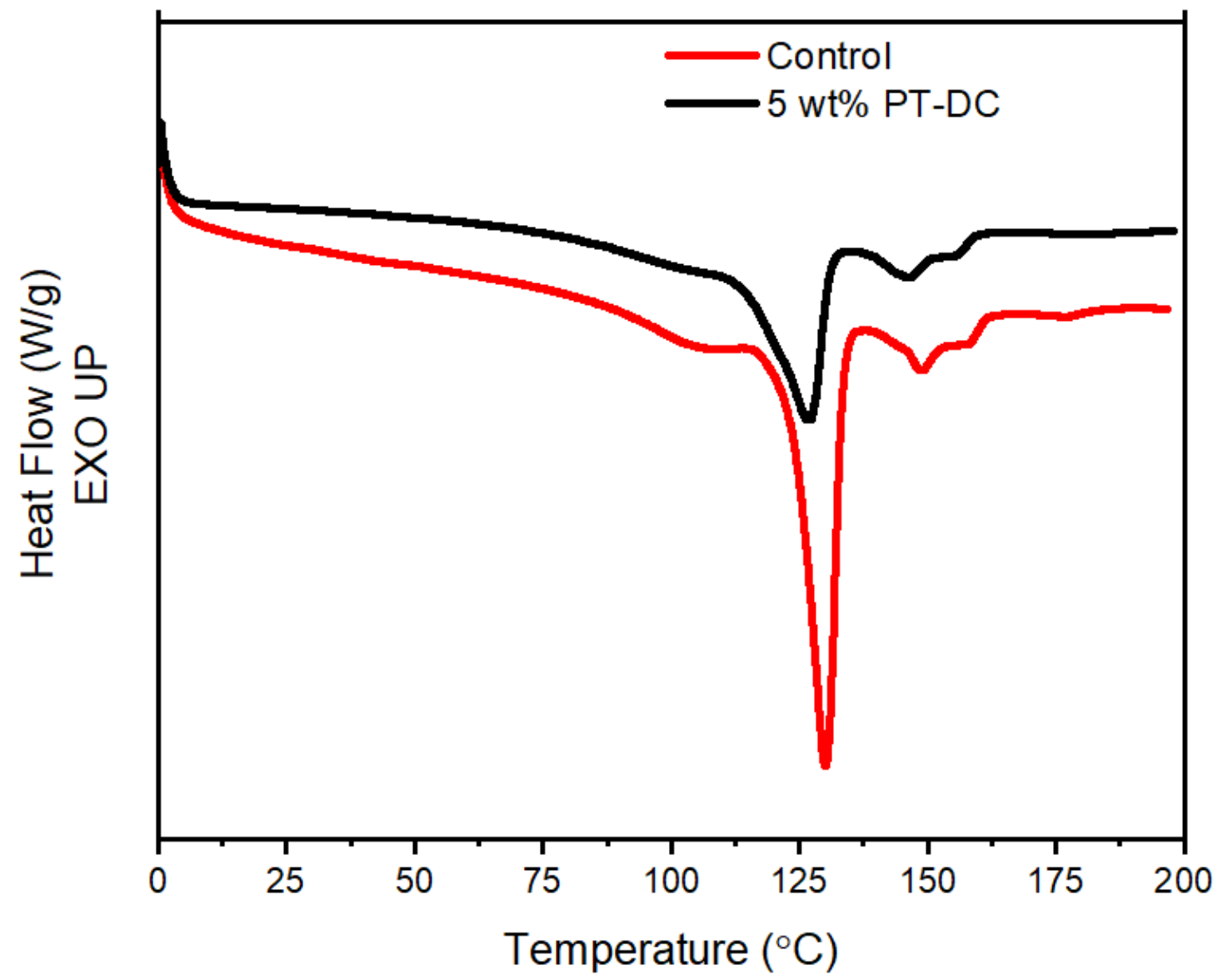

Figure S23. Differential scanning calorimetry of ternary waste blend (36.4% *i*PP, 28.6% HDPE, 35% LDPE) control (red) and ternary waste blend with 5 wt% PT-DC (black).

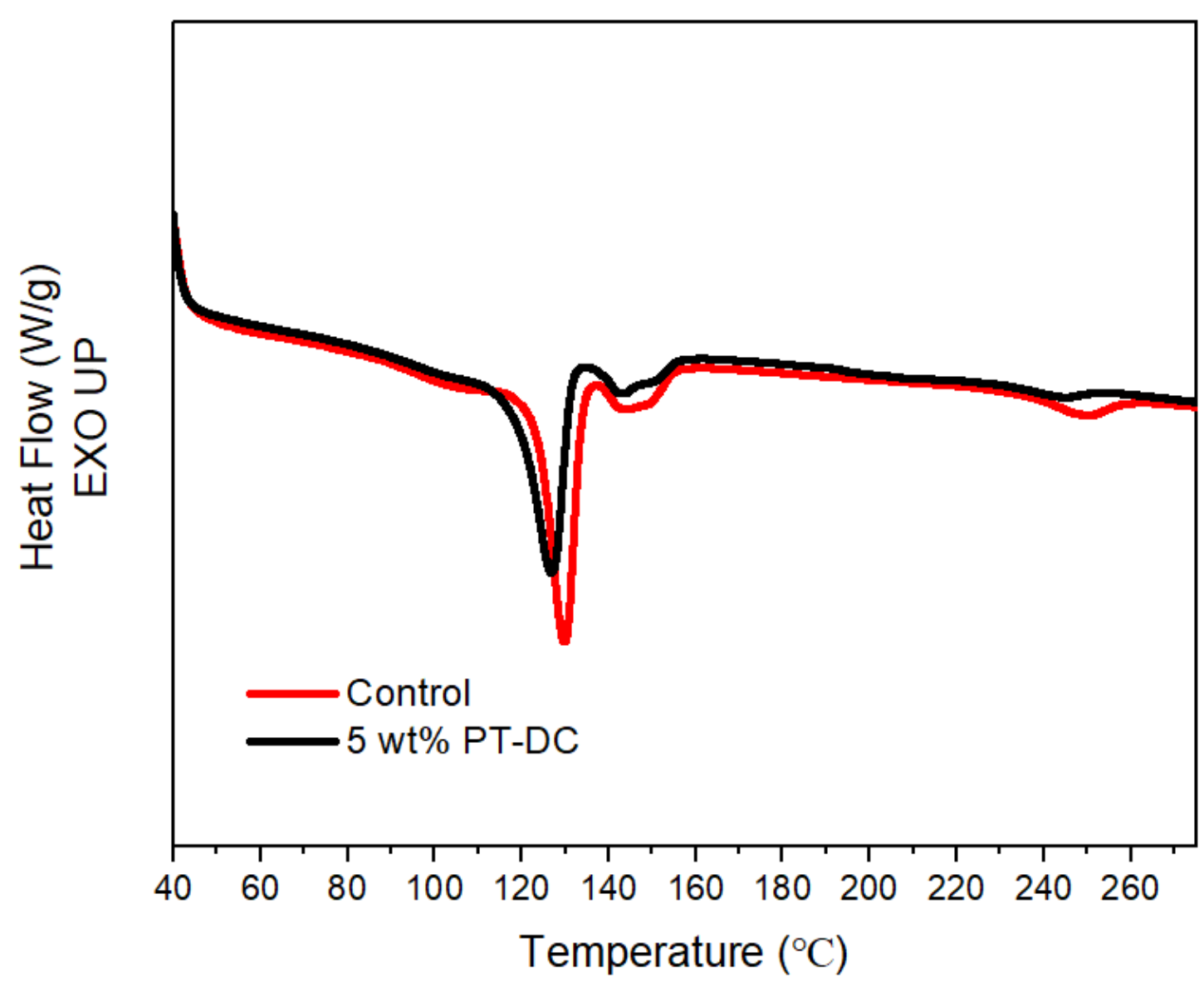

Figure S24. Differential scanning calorimetry of quaternary waste blend (14.4% PET, 30% LDPE, 24.4% HDPE, 31.1% *i*PP) control (red) and quaternary waste blend with 5 wt% PT-DC (black).

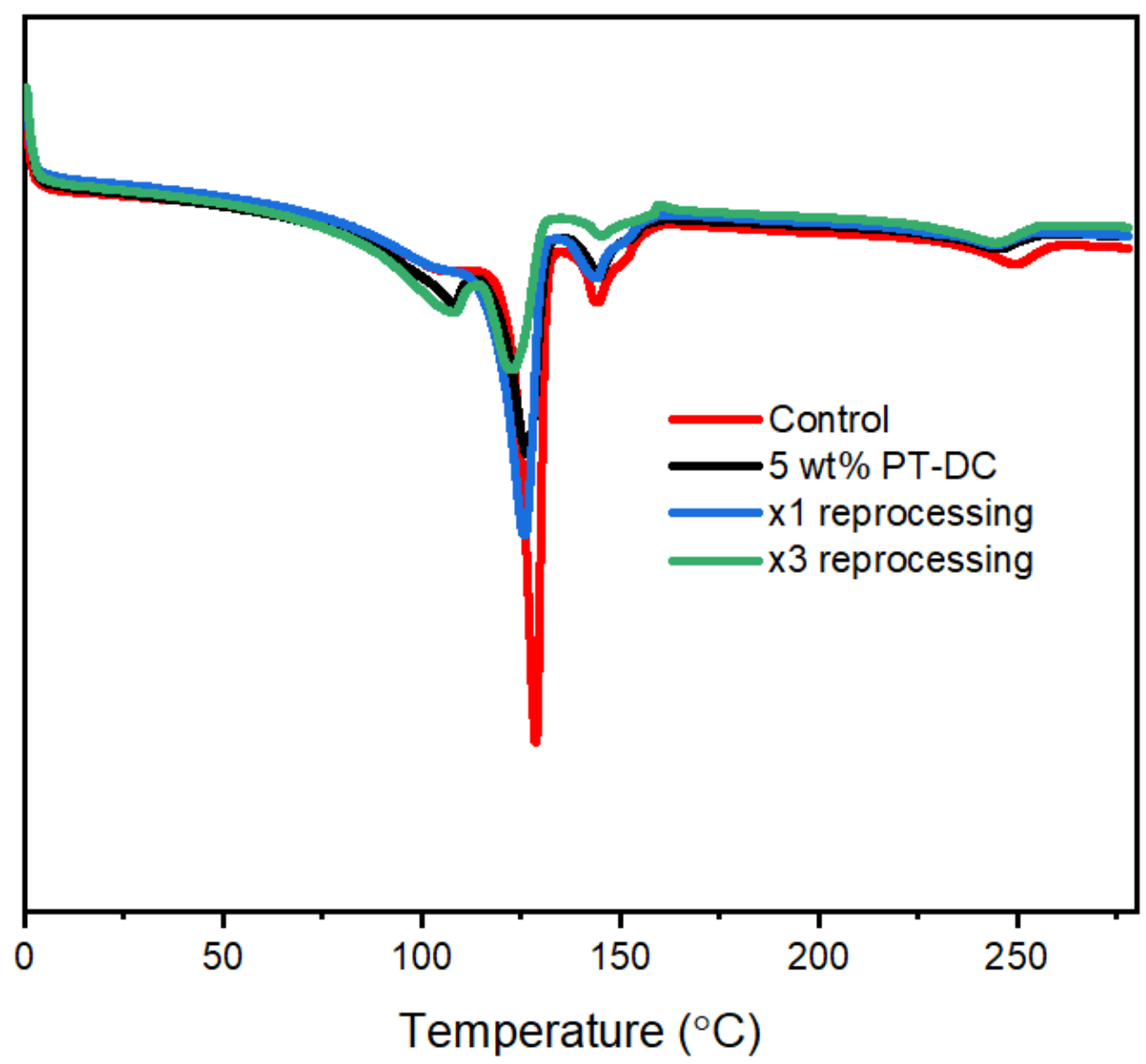

Figure S25. Differential scanning calorimetry of quaternary waste blend (14.4% PET, 30% LDPE, 24.4% HDPE, 31.1% *i*PP) control (red), quaternary waste blend with 5 wt% PT-DC (black), after one reprocessing cycle (blue), and after three reprocessing cycles (green).

Thermogravimetric Analysis

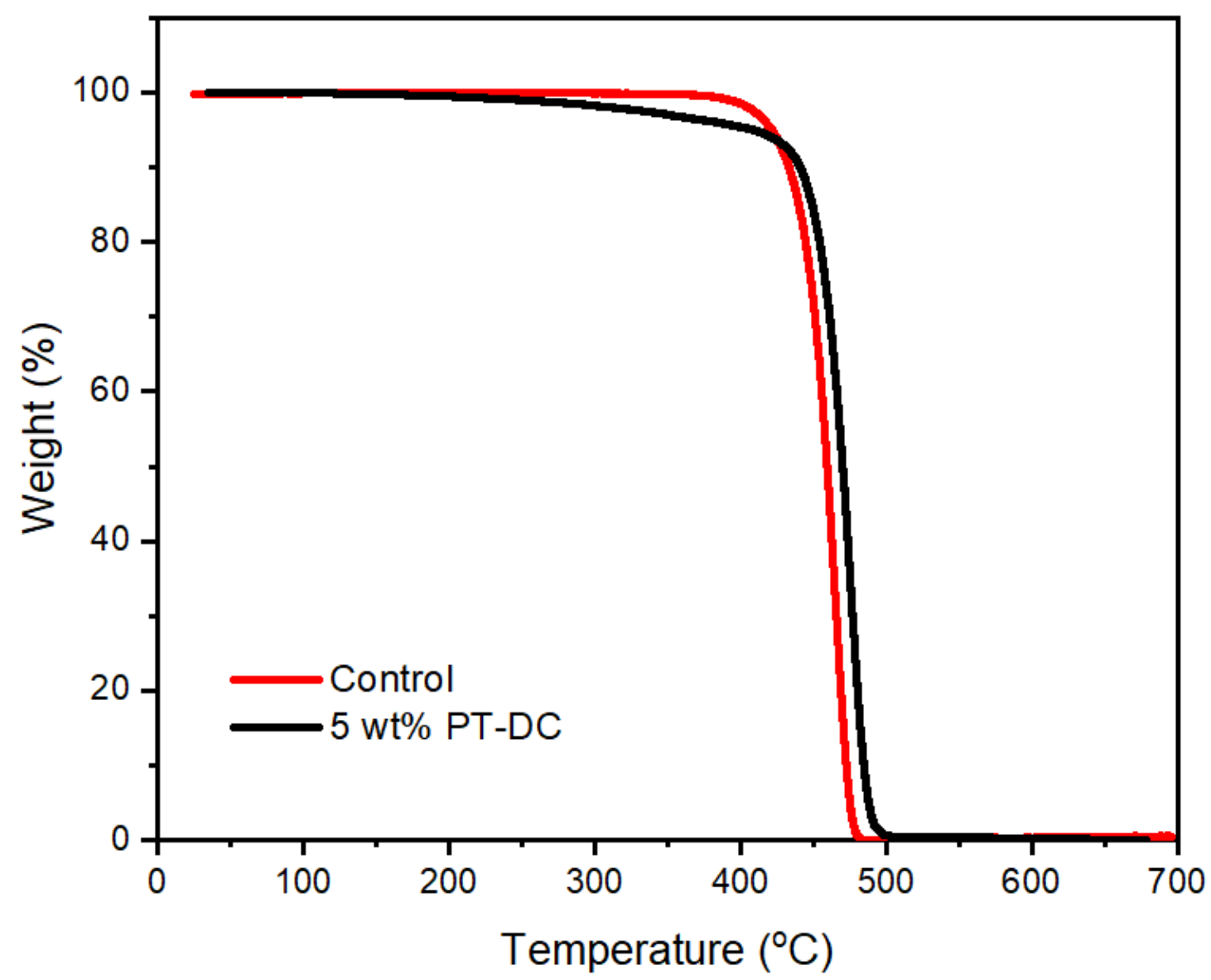

Figure S26. TGA of HDPE control (red) and HDPE with 5 wt% PT-DC (black).

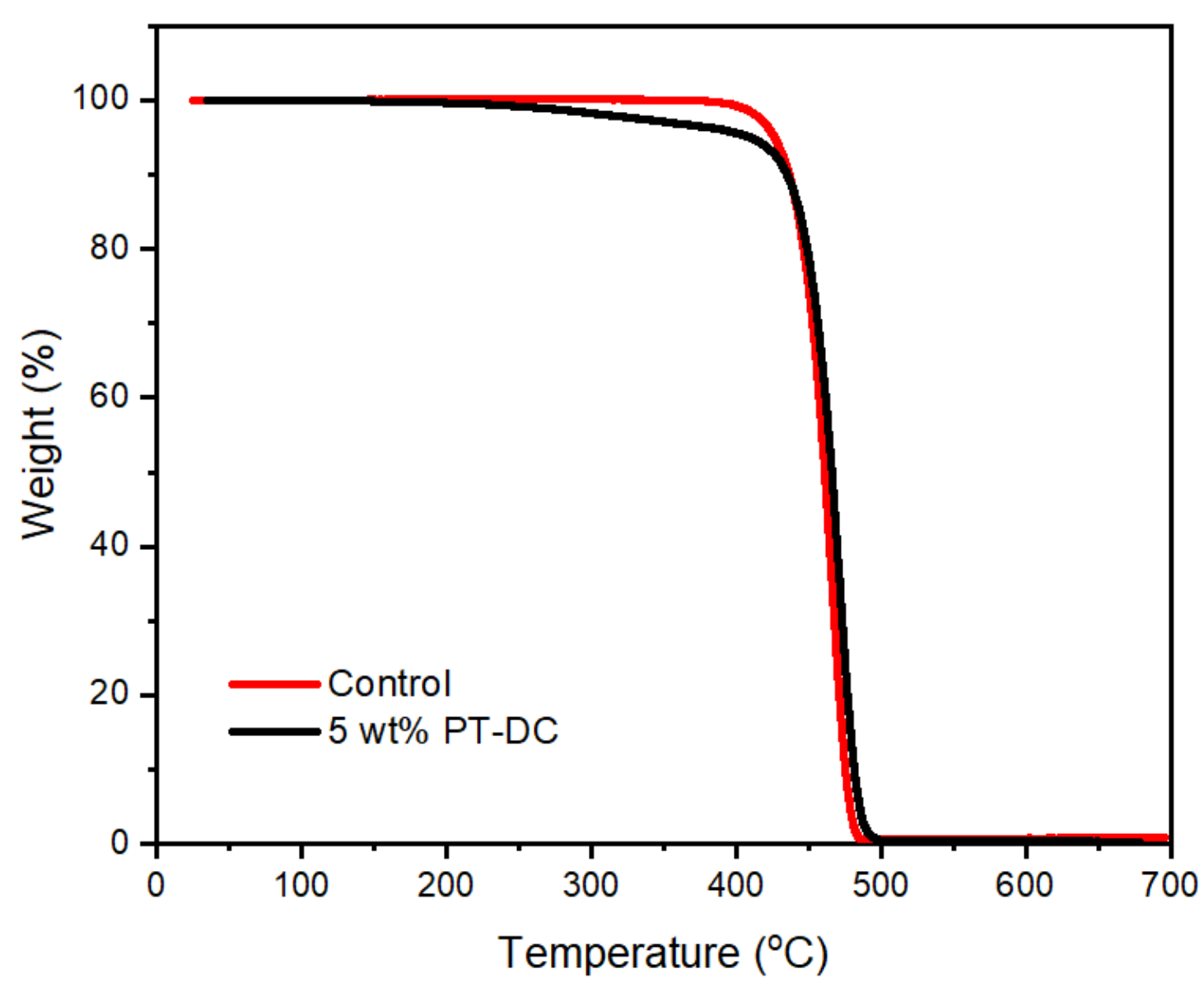

Figure S27. TGA of LDPE control (red) and LDPE with 5 wt% PT-DC (black).

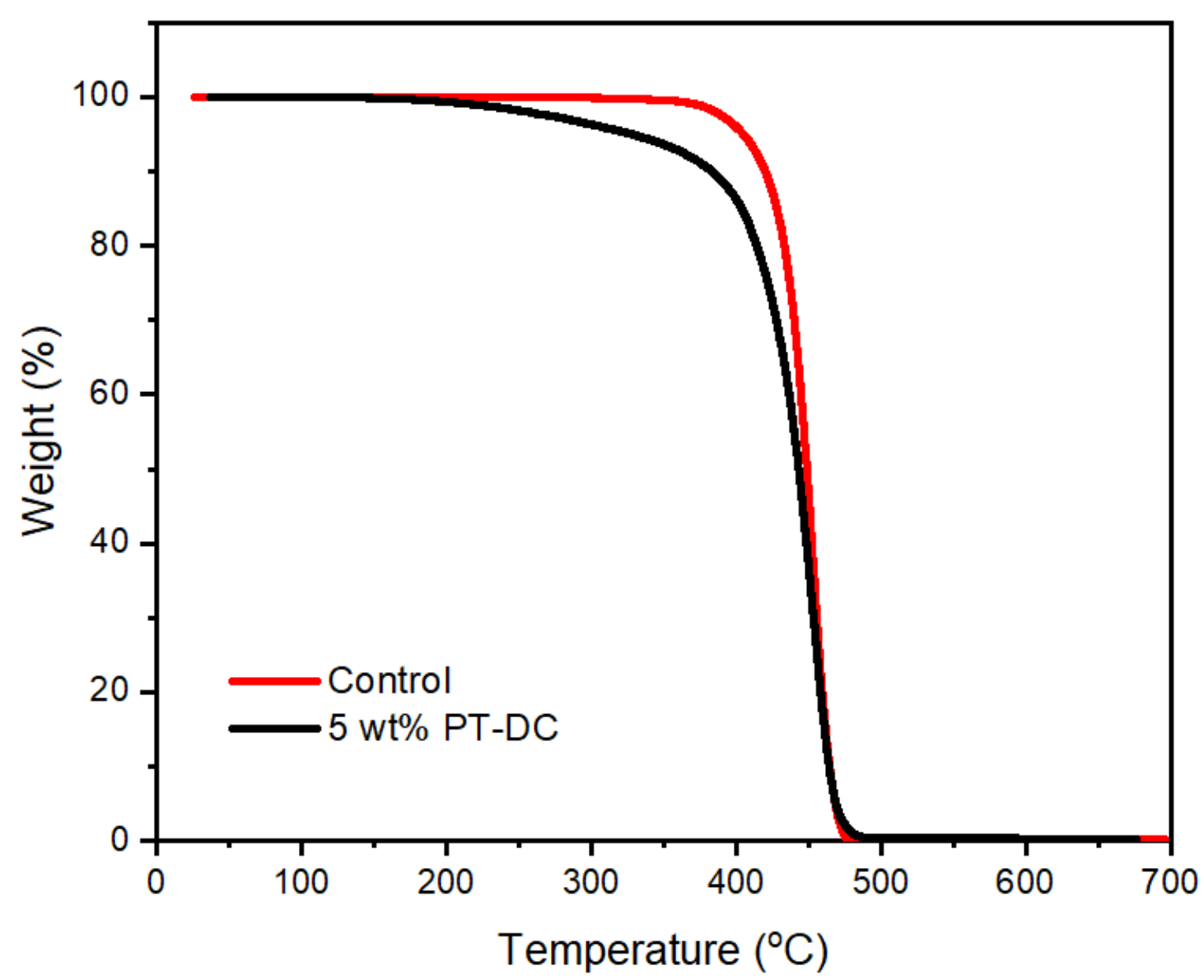

Figure S28. TGA of *i*PP control (red) and *i*PP with 5 wt% PT-DC (black).

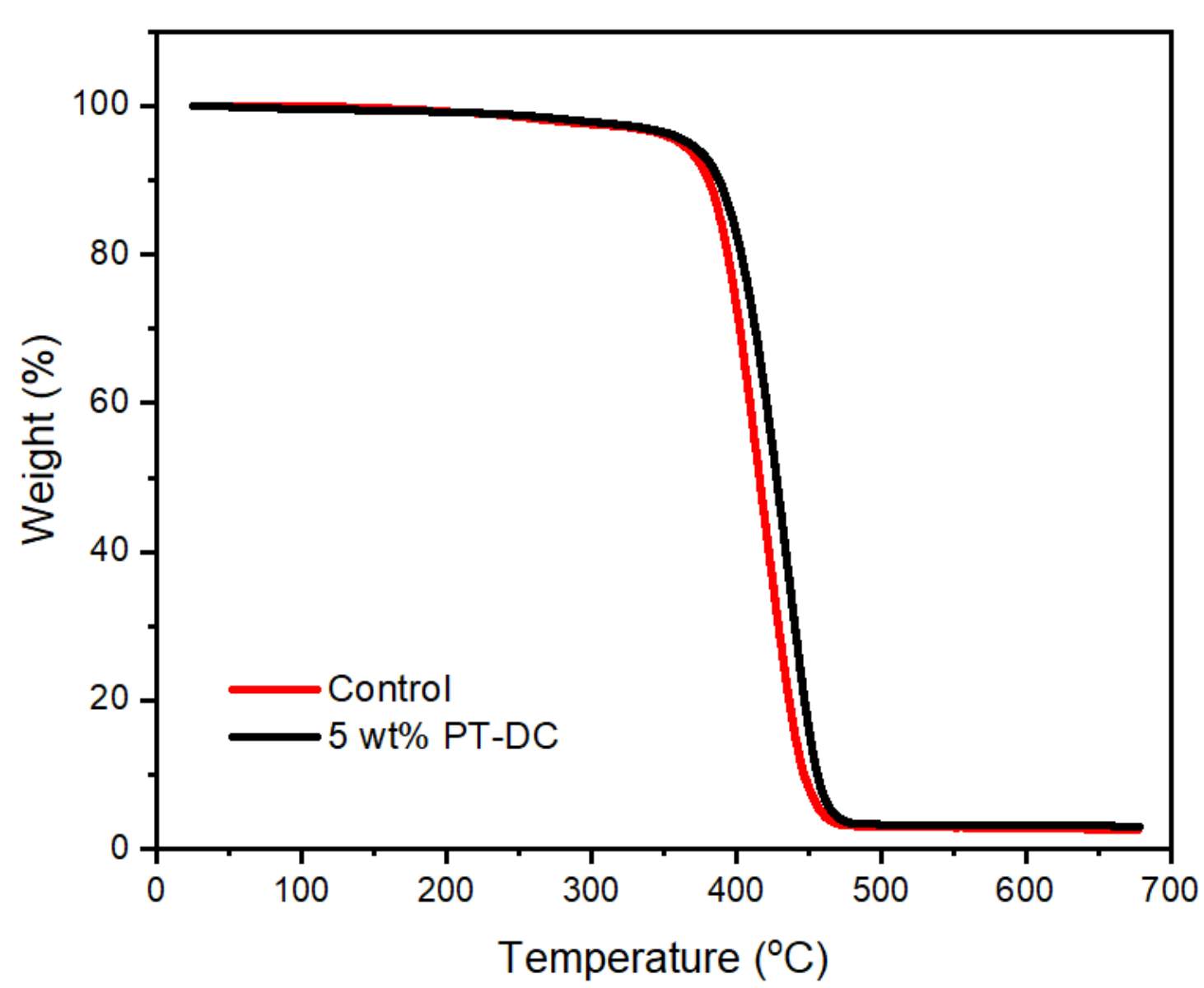

Figure S29. TGA of Nylon-6 control (red) and Nylon-6 with 5 wt% PT-DC (black).

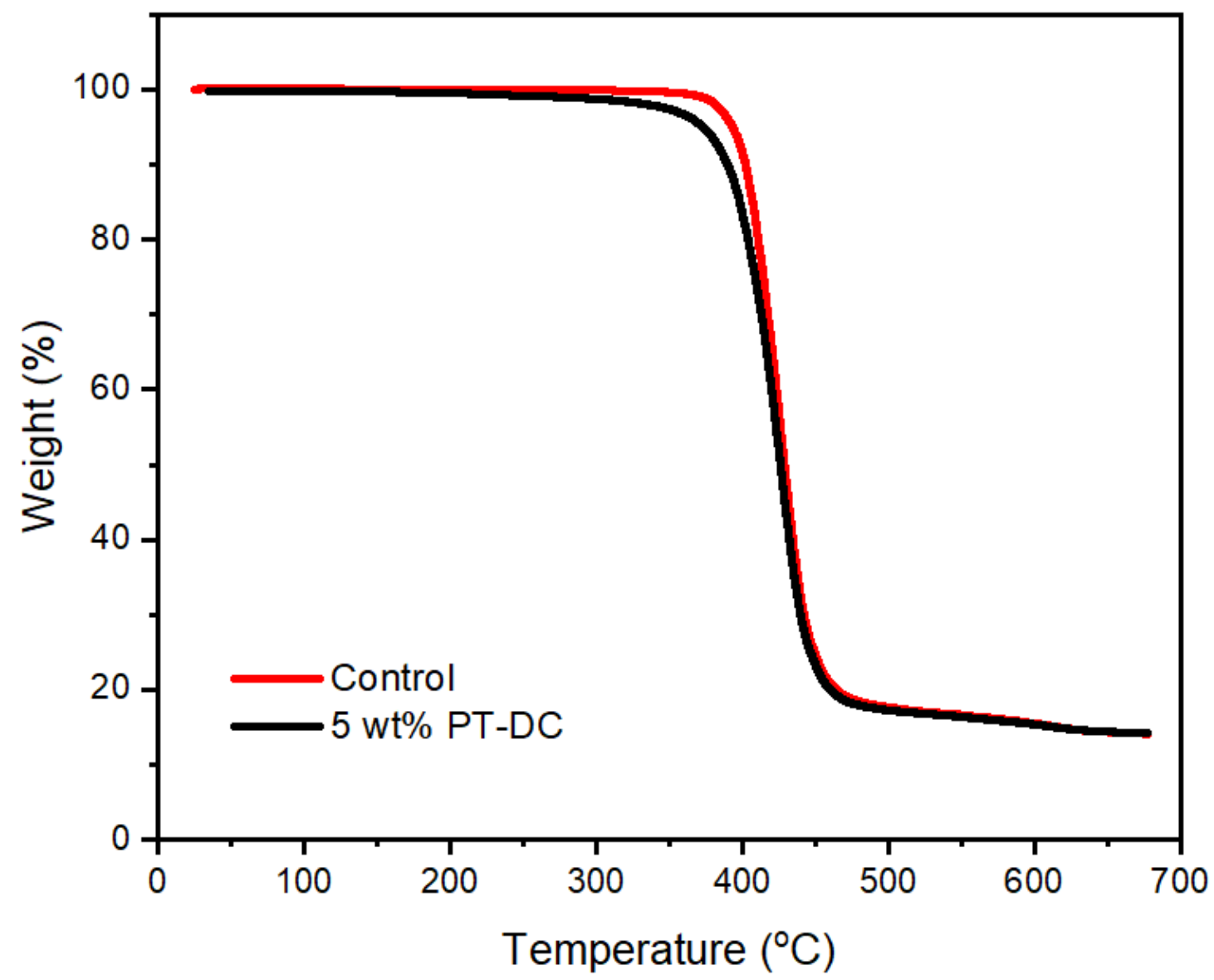

Figure S30. TGA of PET control (red) and PET with 5 wt% PT-DC (black).

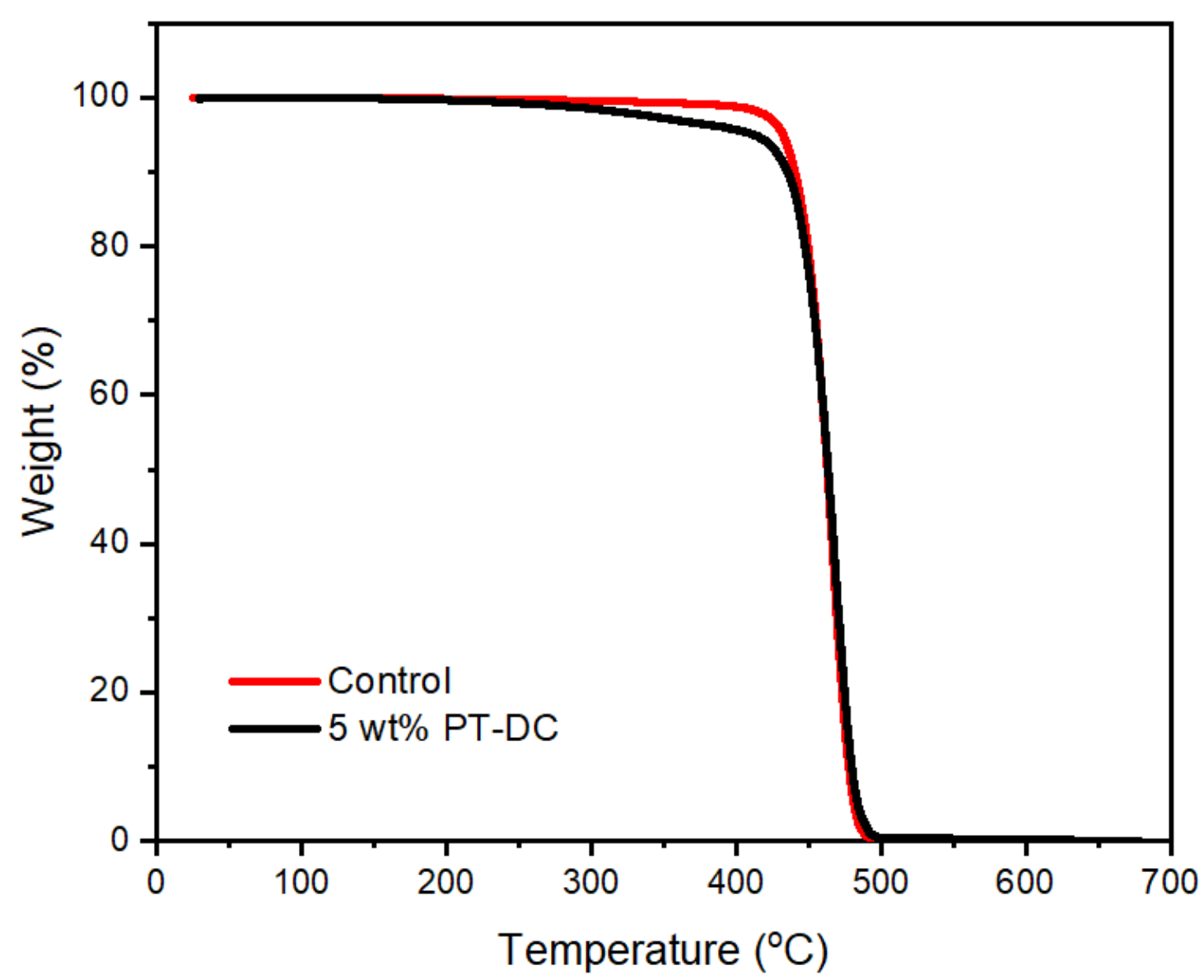

Figure S31. TGA of HDPE-*i*PP (70:30) control (red) and HDPE-*i*PP (70:30) with 5 wt% PT-DC (black).

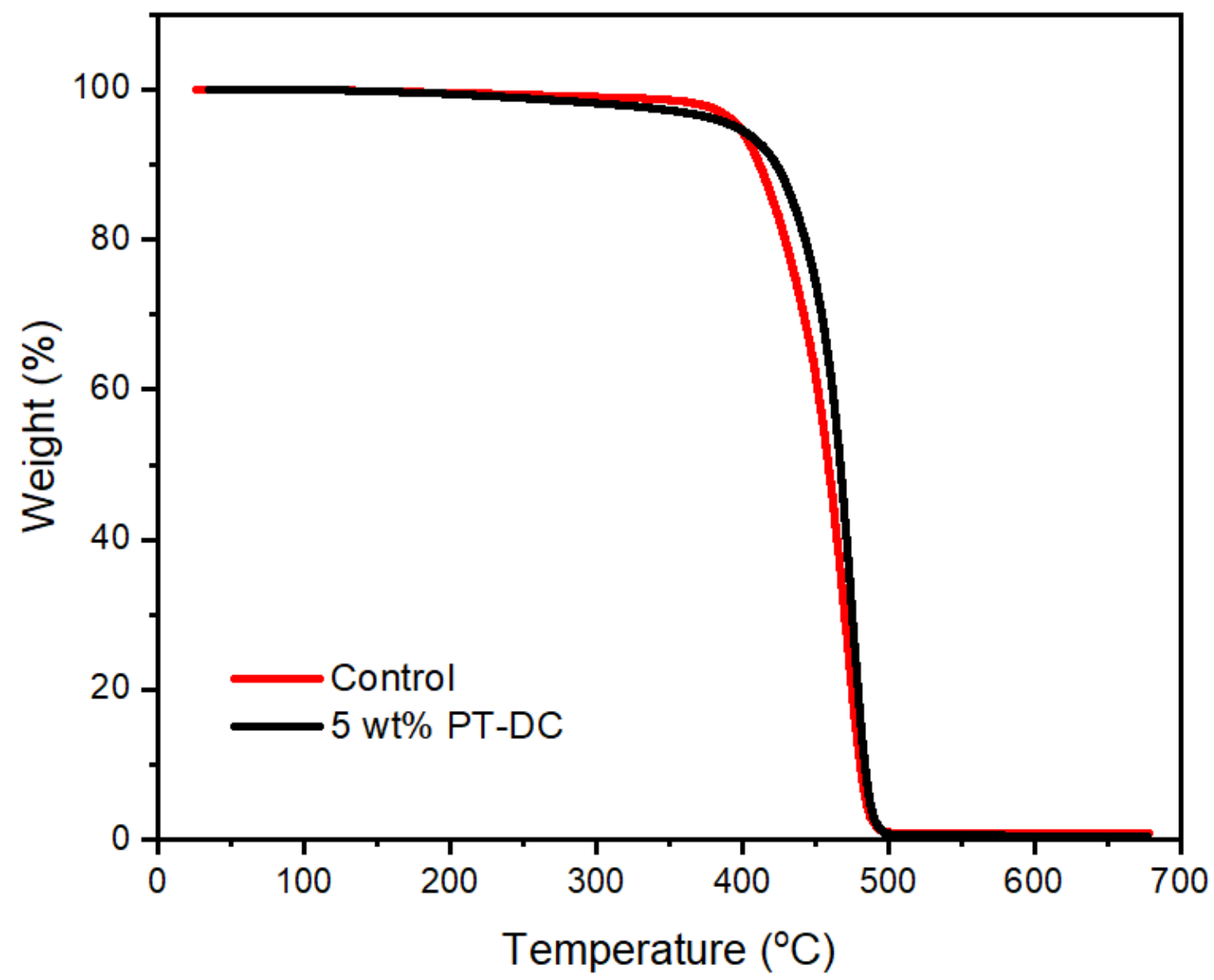

Figure S32. TGA of HDPE-Nylon 6 (80:20) control (red) and HDPE-Nylon 6 (80:20) with 5 wt% PT-DC (black).

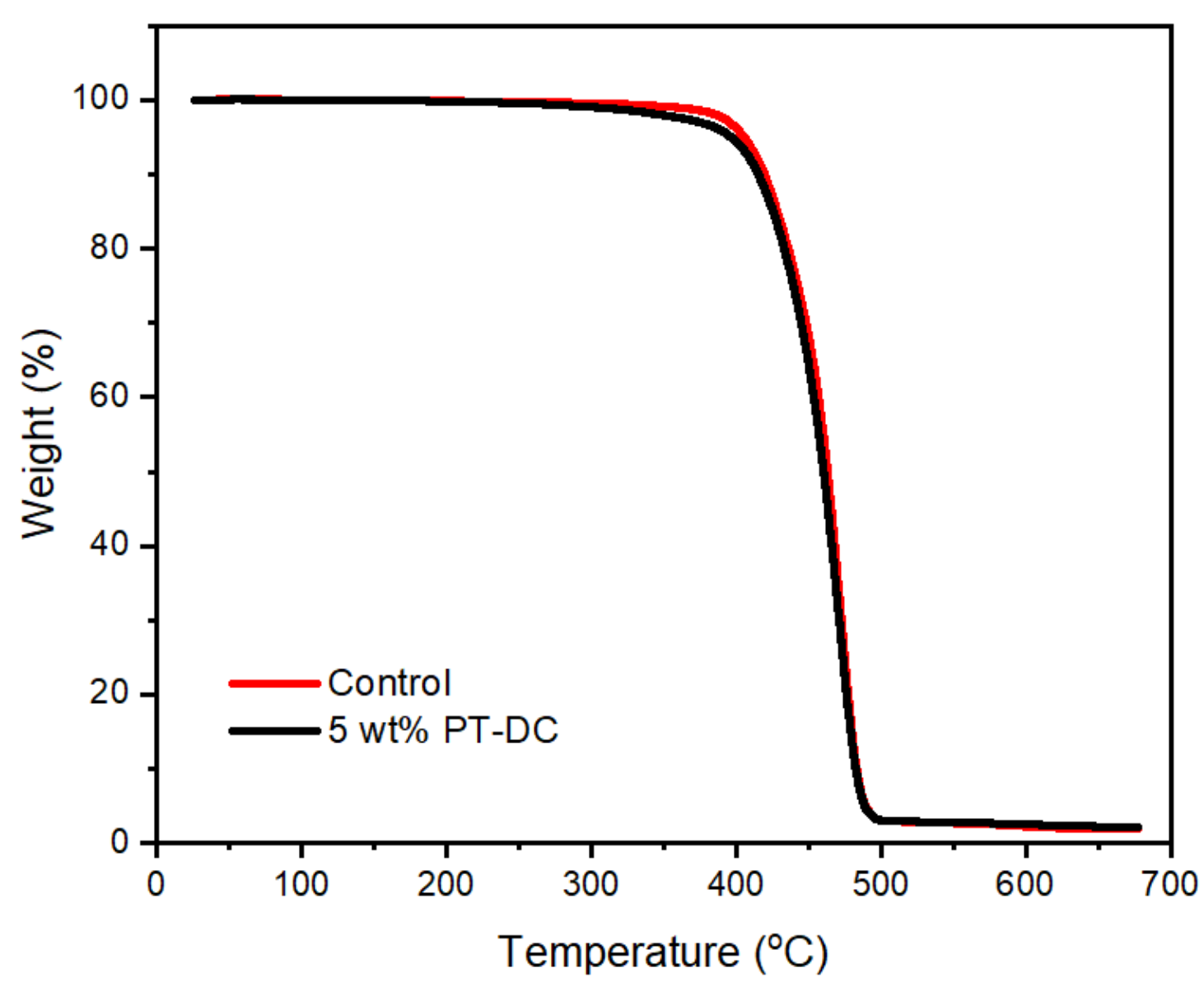

Figure S33. TGA of HDPE-PET (80:20) control (red) and HDPE-PET (80:20) with 5 wt% PT-DC (black).

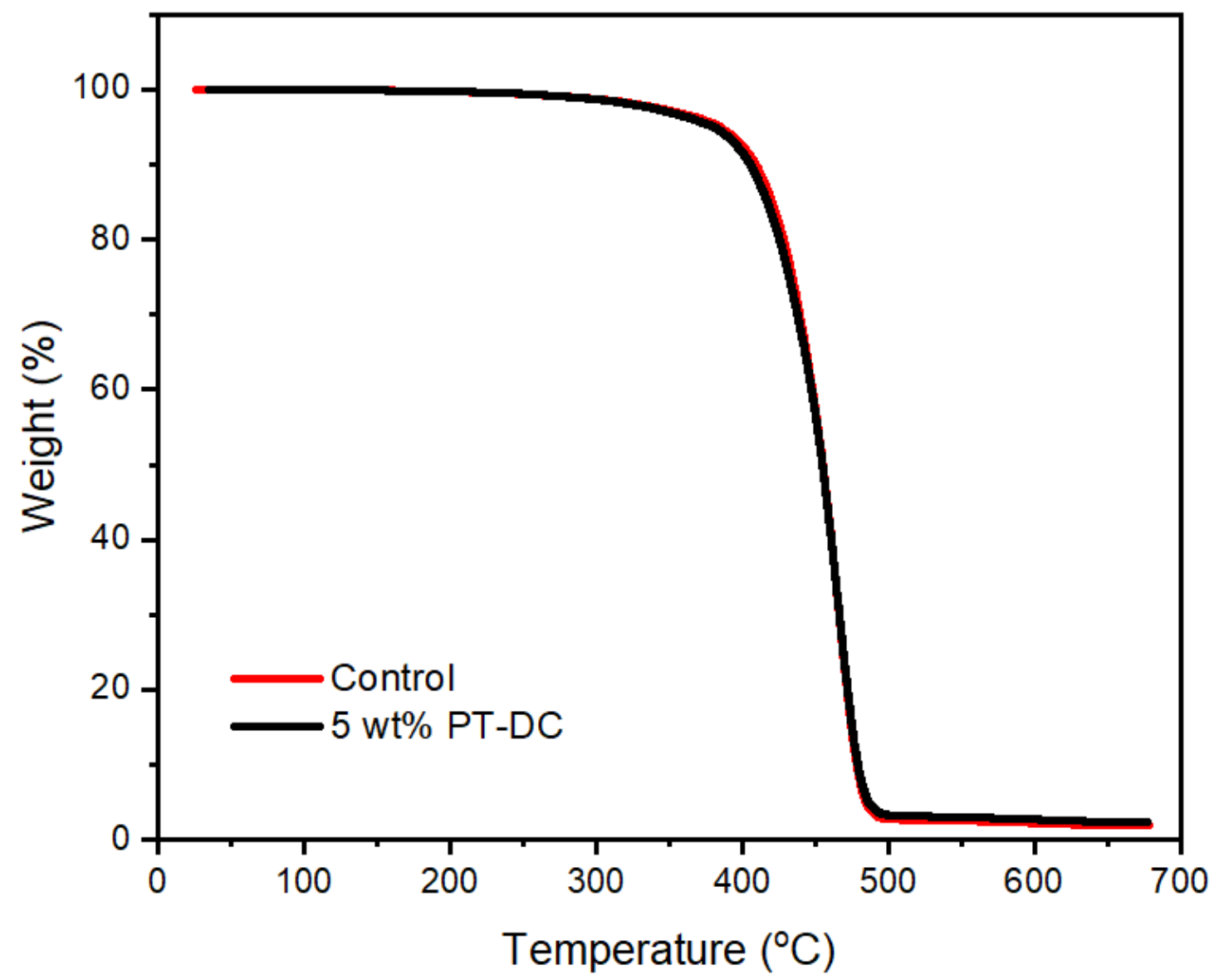

Figure S34. TGA of LDPE-PET (80:20) control (red) and LDPE-PET (80:20) with 5 wt% PT-DC (black).

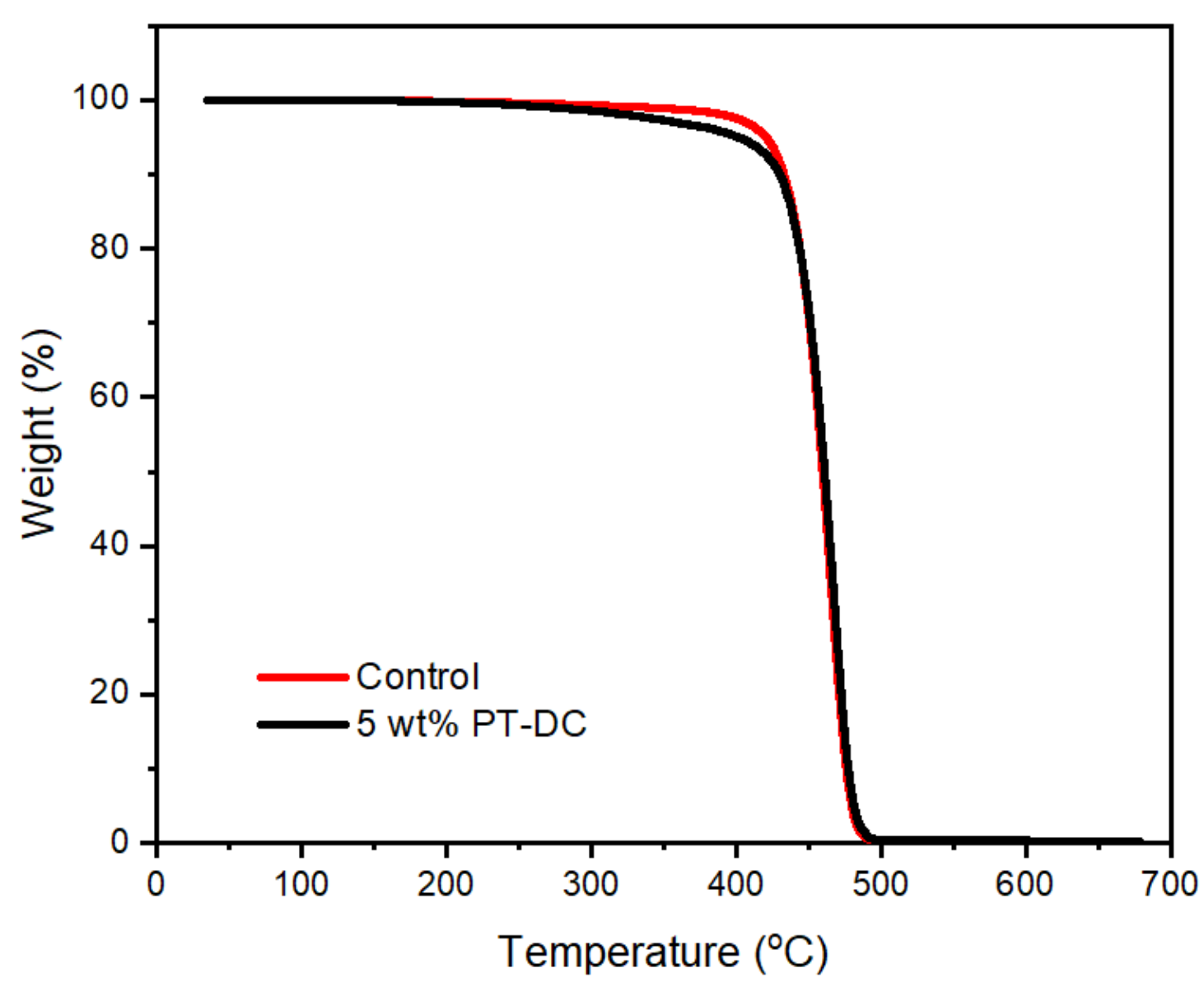

Figure S35. TGA of ternary waste (36.4% *i*PP, 28.6% HDPE, 35% LDPE) control (red) and ternary waste (36.4% *i*PP, 28.6% HDPE, 35% LDPE) with 5 wt% PT-DC (black).

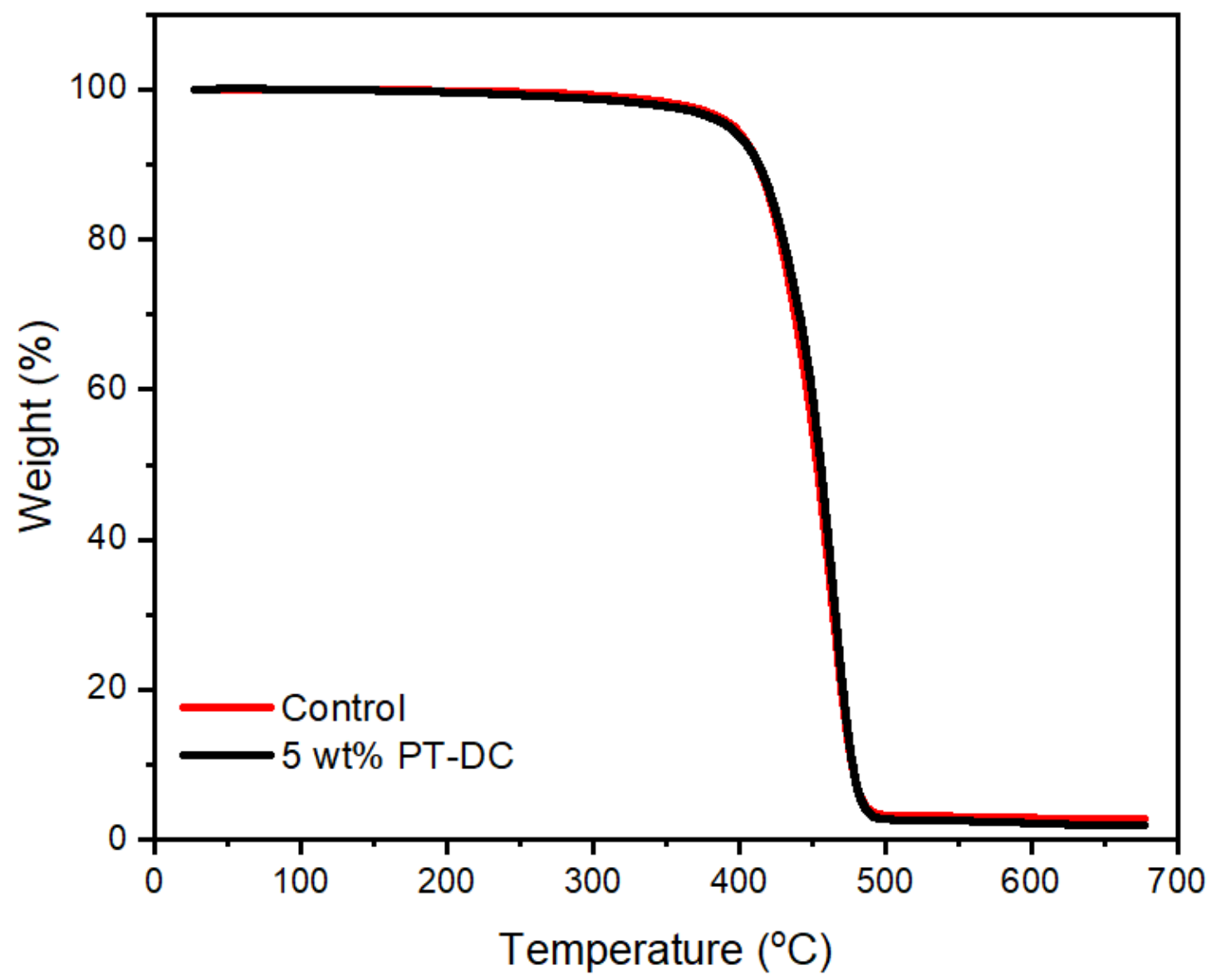

Figure S36. TGA of quaternary waste (14.4% PET, 30% LDPE, 24.4% HDPE, 31.1% *i*PP) control (red) and quaternary waste with 5 wt% PT-DC (black).

Gel Fraction

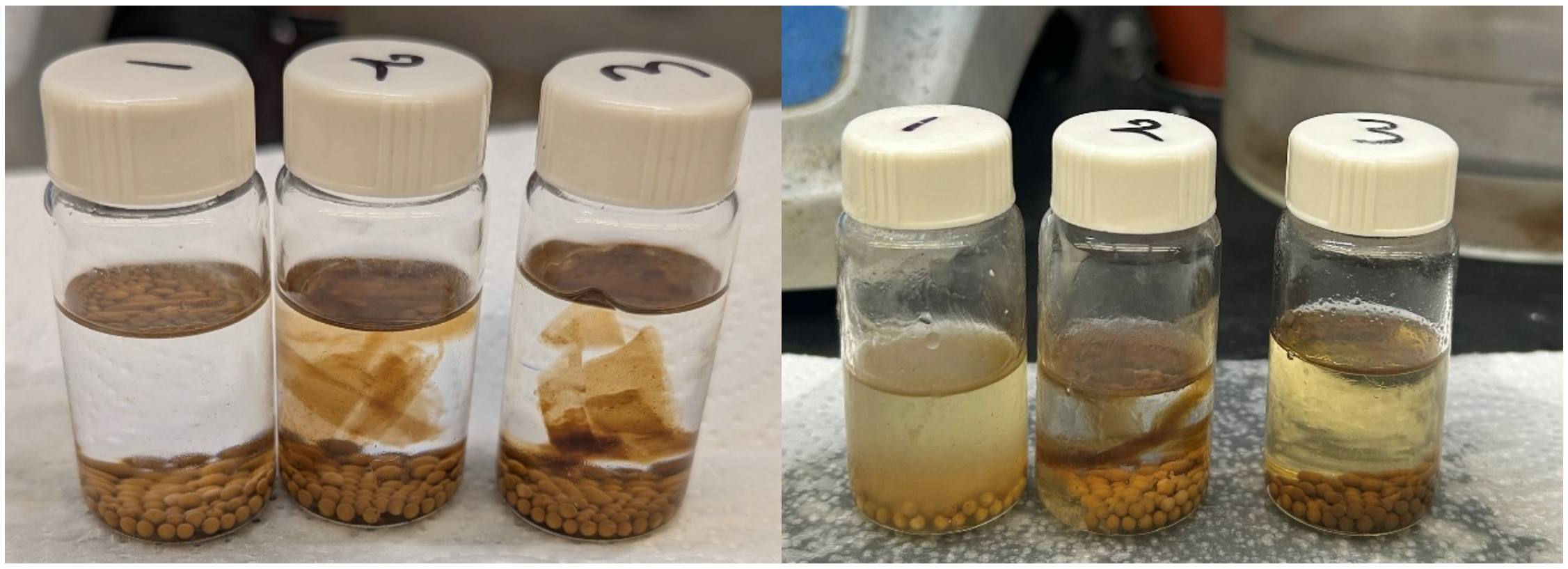

Figure S37. Gel Fraction analysis of ternary (LDPE/HDPE/*i*PP) blend for 24 h at 150 °C in xylenes (left) and after 3 h since TBAF was added only to vial 3 (right). Vial 1, control ternary blend sample; vial 2 and vial 3 (left image), ternary blend treated with 5 wt% PT-DC; vial 3 (right image), ternary blend treated with 5 wt% PT-DC and added with 1 mL of TBAF (1.0 M in THF).

Table S1. Gel fraction data of crosslinked homopolymers and binary blend

Sample	Mass (initial)(mg)	Mass (final)(mg)	% X-link	Average %
LDPE	57	36	63.158	63.541
	56.8	36.7	64.613	
	70.8	44.5	62.853	
HDPE	69.7	24.1	34.577	35.616
	73.2	27.2	37.158	
	74.5	26.16	35.114	
HDPE/*i*PP	64	38.5	60.156	58.604
	56.9	34	59.754	
	70.3	39.3	55.903	

Creep-Recovery

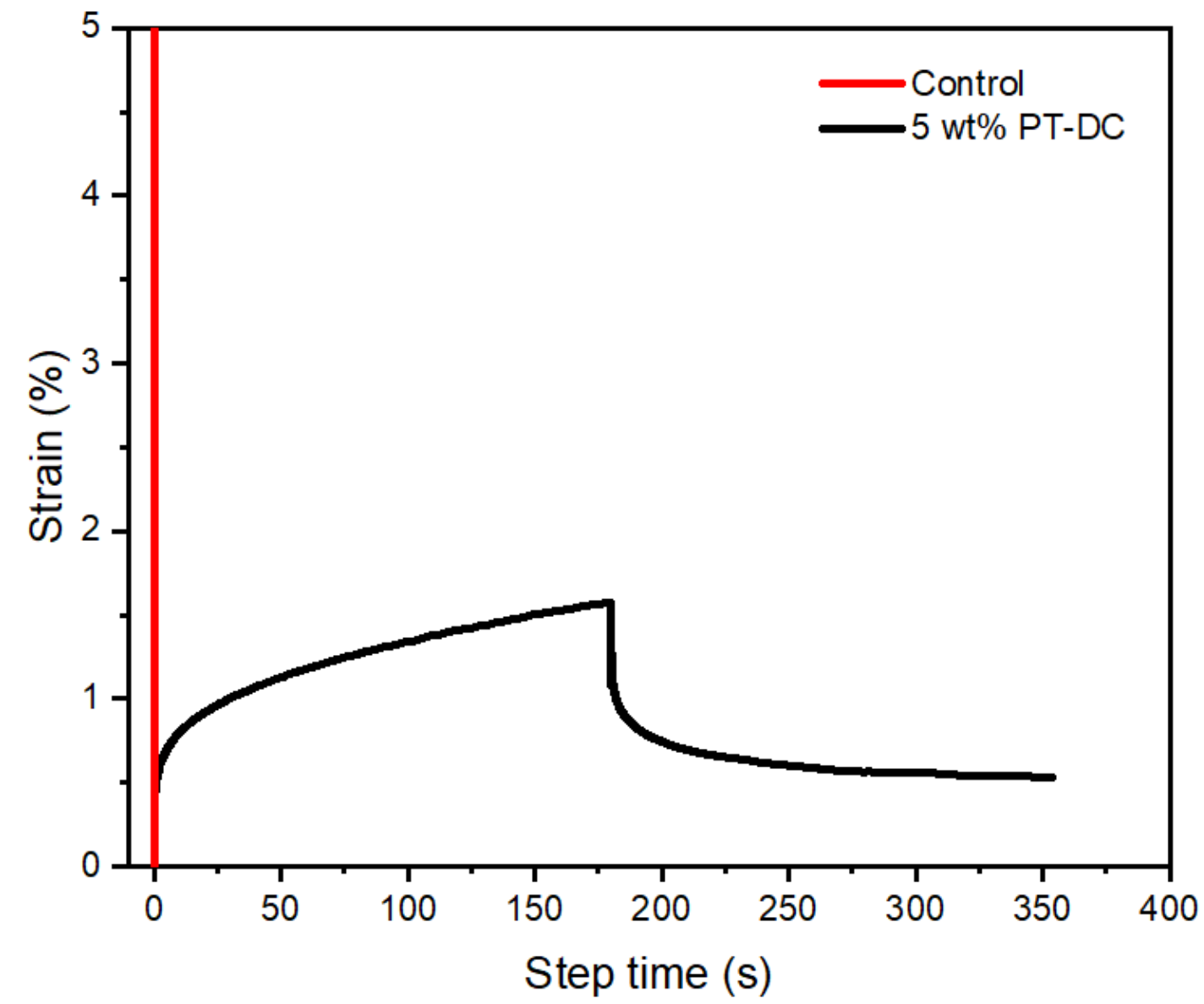

Figure S38. Creep recovery of HDPE control (red) and HDPE with 5 wt% PT-DC (black) at 180 °C.

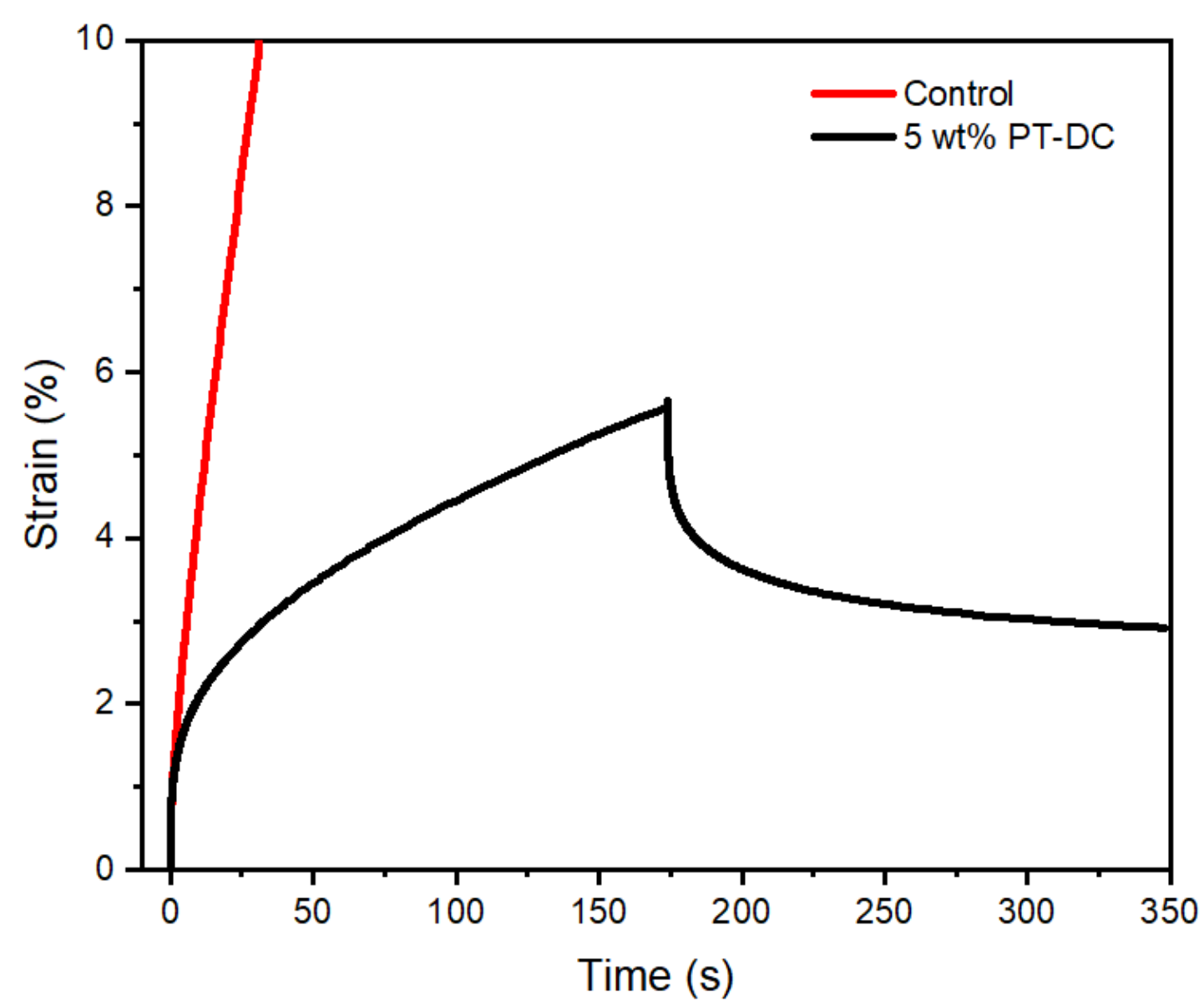

Figure S39. Creep recovery of PMMA control (red) and PMMA with 5 wt% PT-DC (black) at 240 °C.

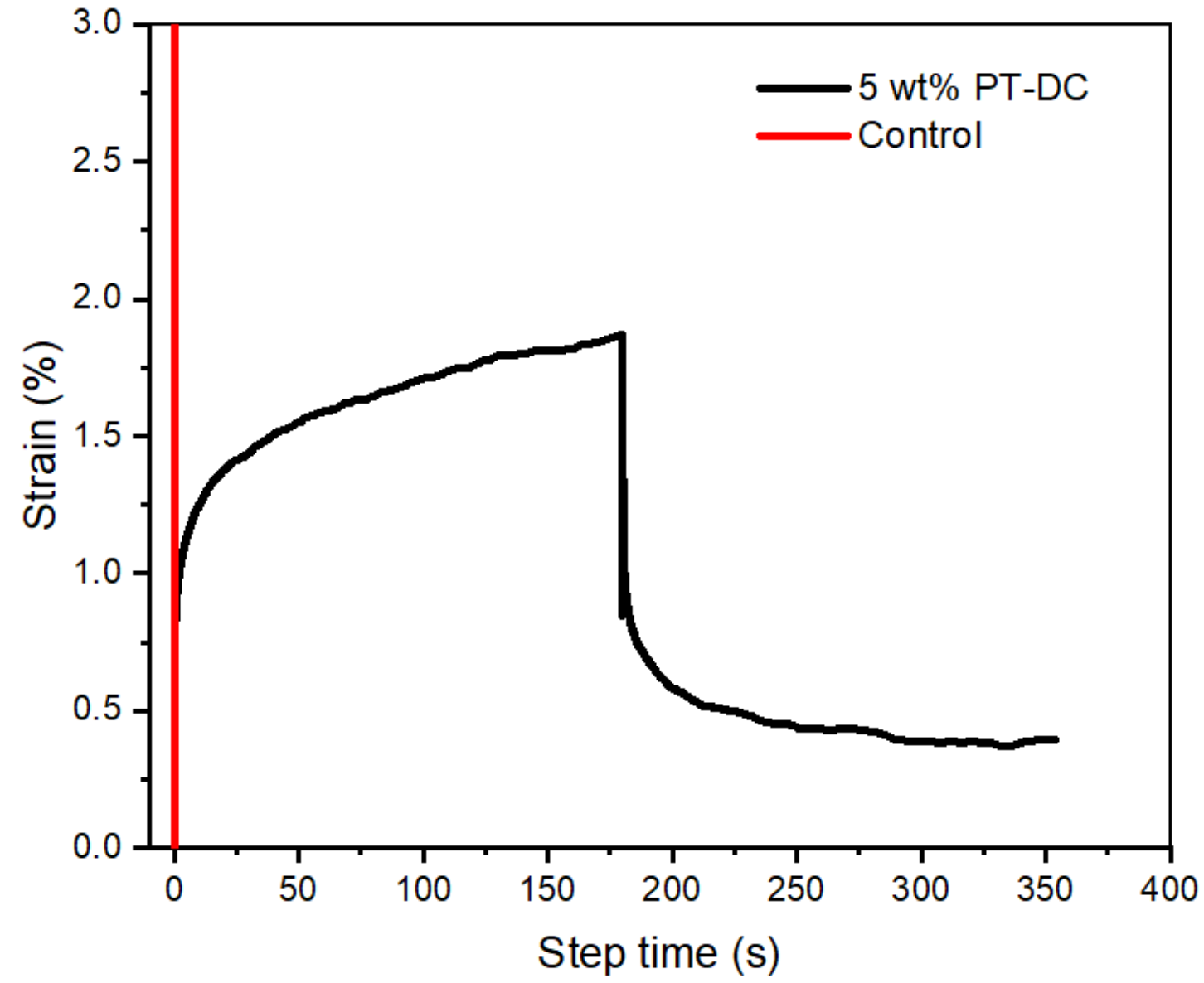

Figure S40. Creep recovery of LDPE control (red) and LDPE with 5 wt% PT-DC (black) at 180 °C.

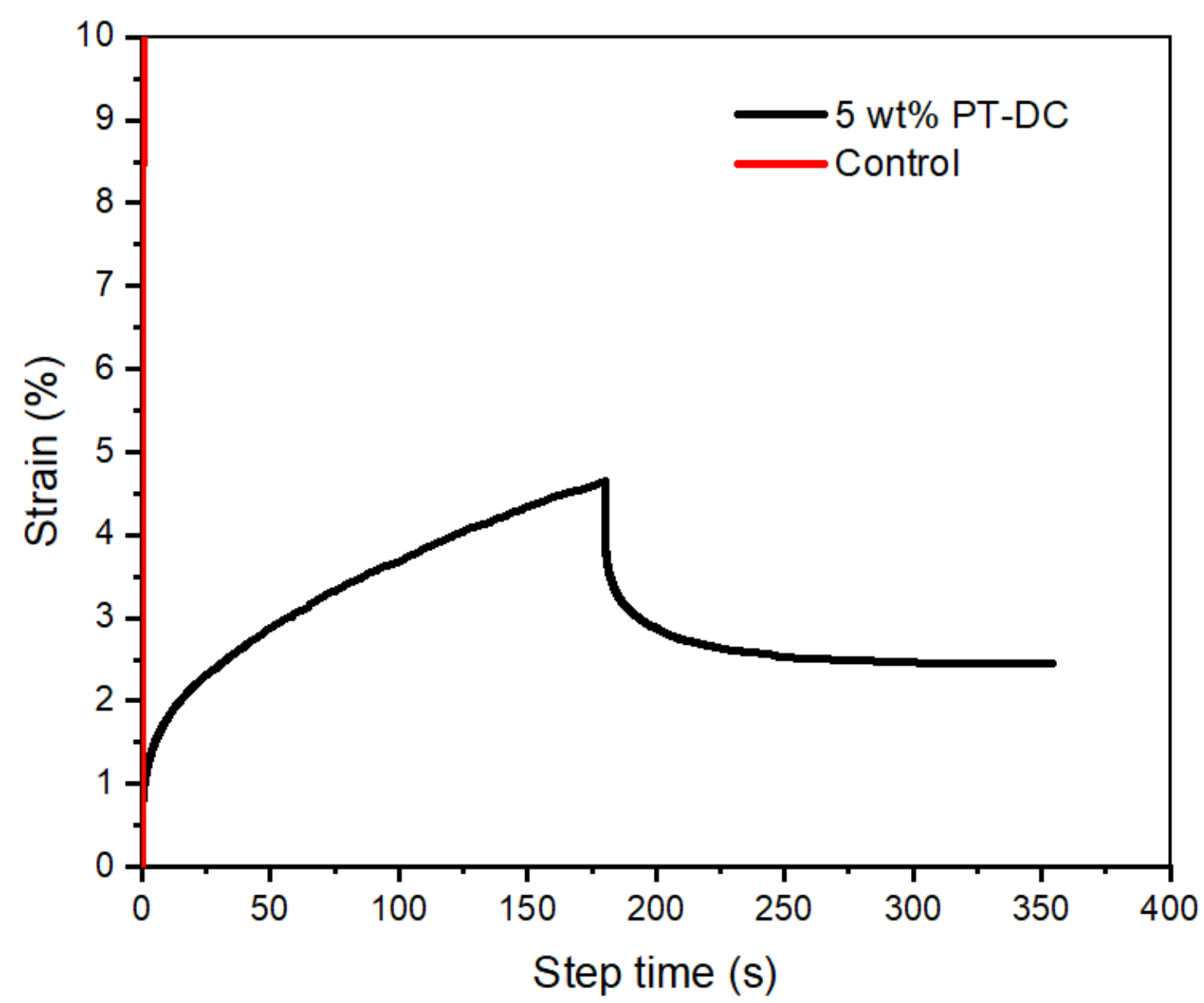

Figure S41. Creep recovery of HDPE-*i*PP (70:30) control (red) and HDPE-*i*PP (70:30) with 5 wt% PT-DC (black) at 180 °C.

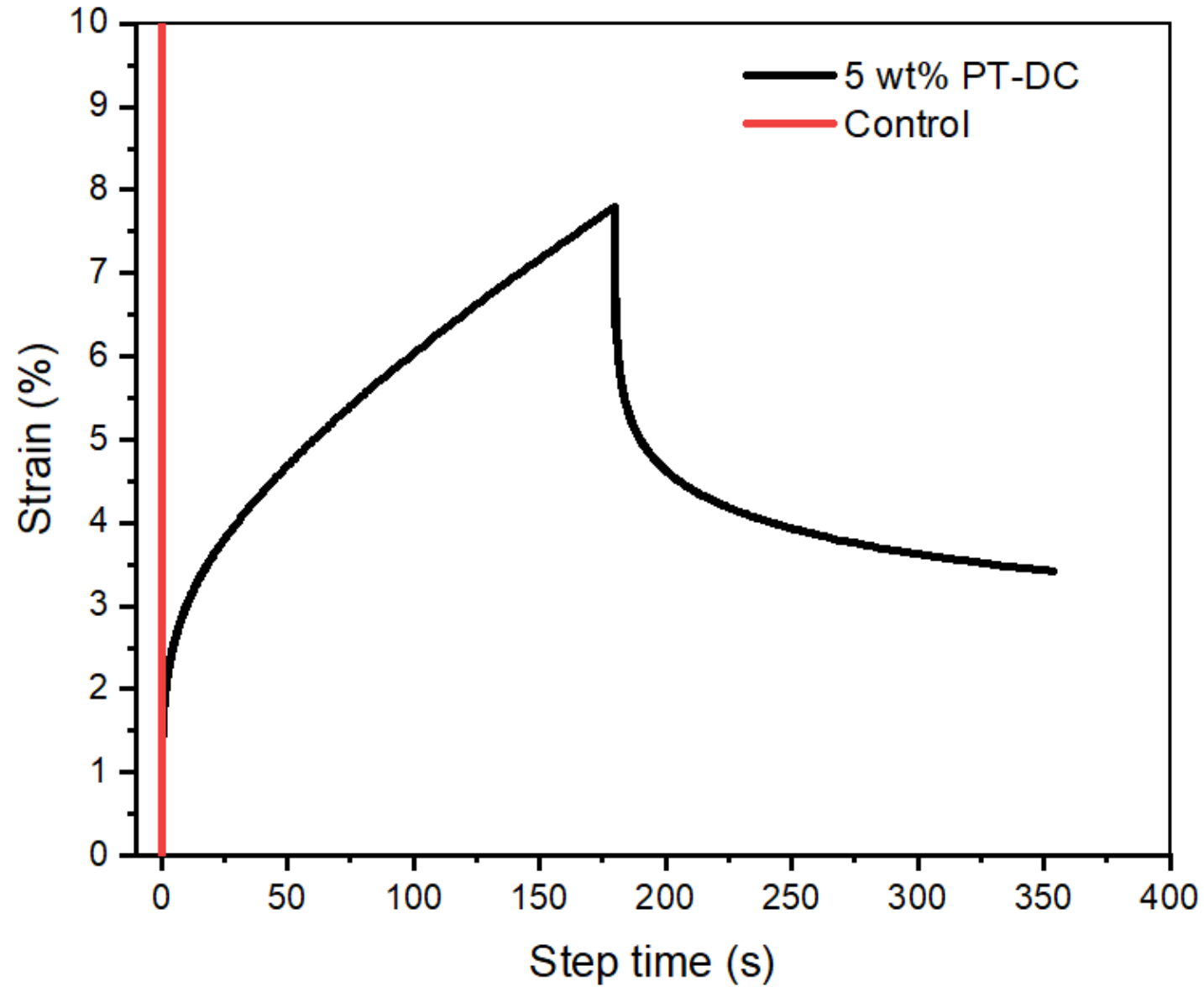

Figure S42. Creep recovery of LDPE-PET (80:20) control (red) and LDPE-PET (80:20) with 5 wt% PT-DC(black) at 270 °C.

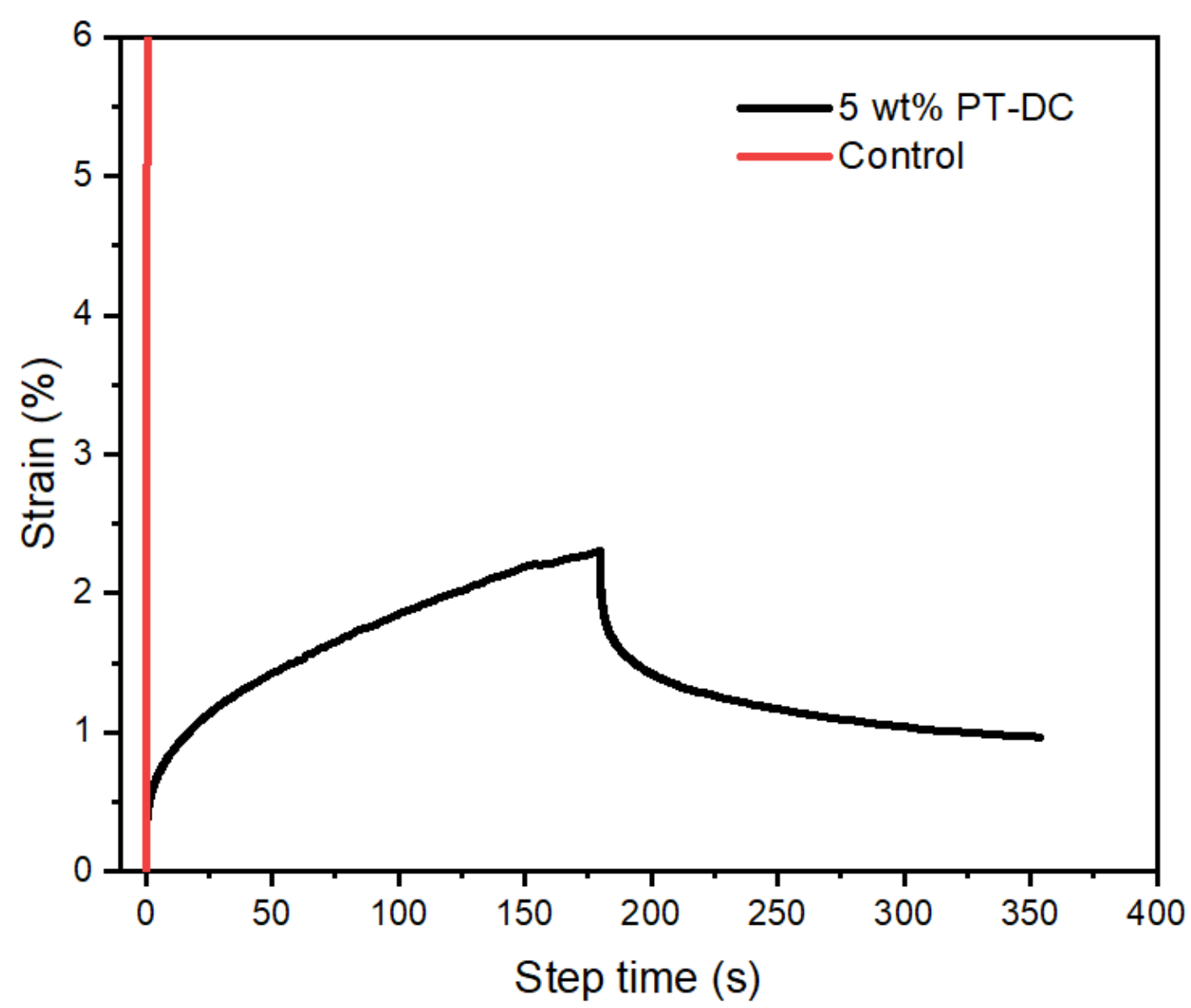

Figure S43. Creep recovery of HDPE-PET (80:20) control (red) and HDPE-PET (80:20) with 5 wt% PT-DC (black) at 270 °C.

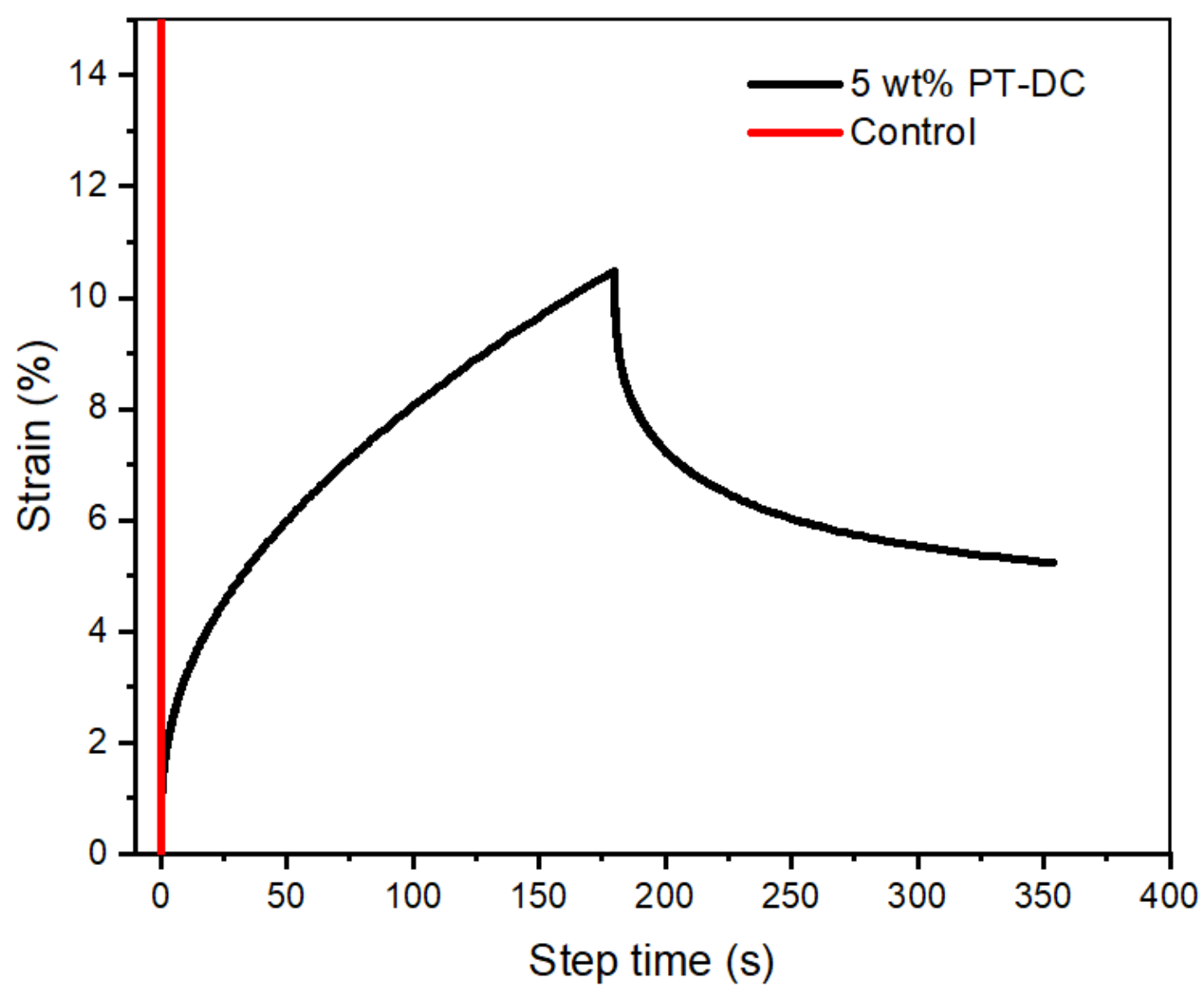

Figure S44. Creep recovery of HDPE-Nylon-6 (80:20) control (red) and HDPE-Nylon6 (80:20) with 5 wt% PT-DC (black) at 240 °C.

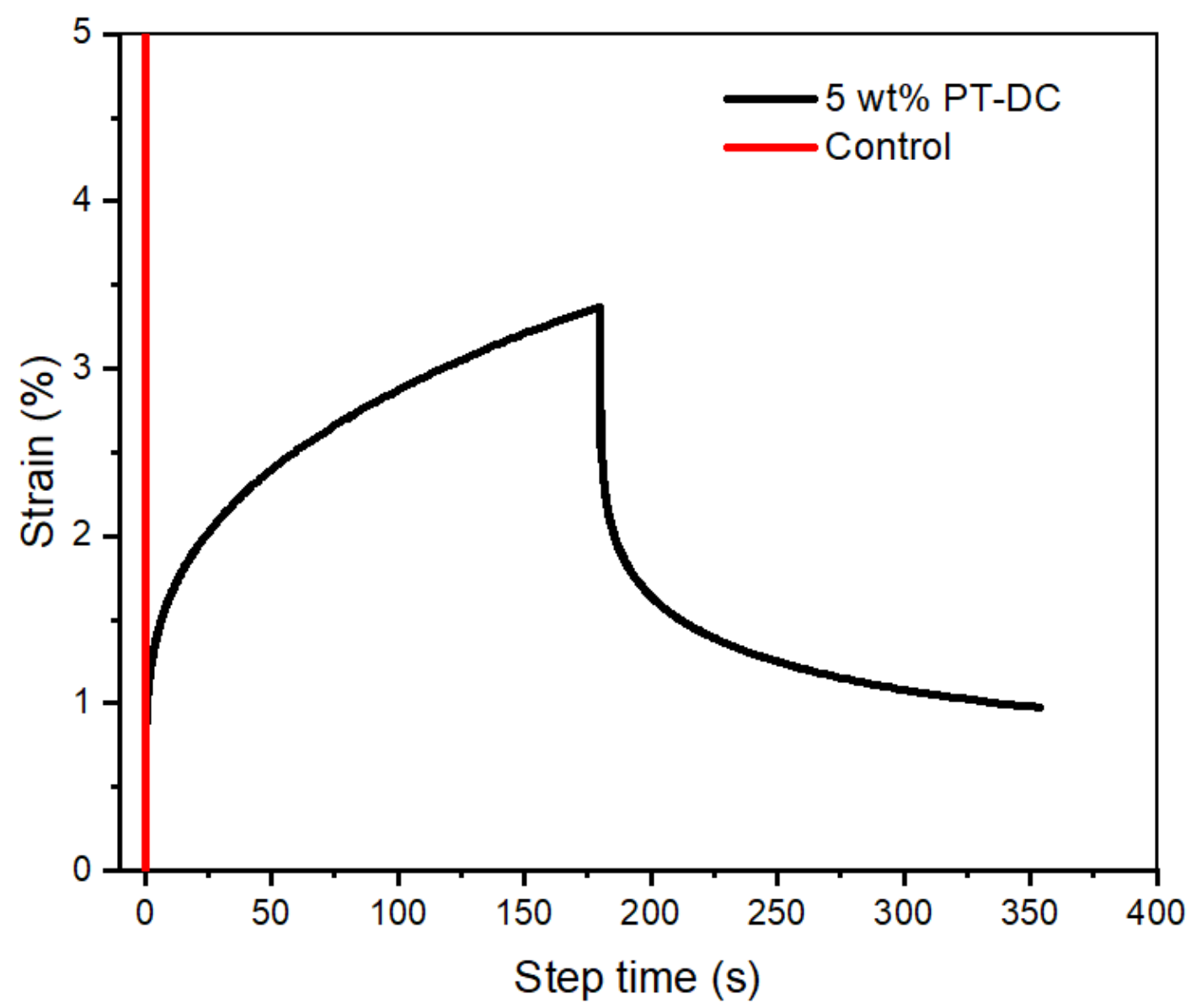

Figure S45. Creep recovery of ternary mixed plastic waste blend control (red) and ternary mixed plastic waste blend with 5 wt% PT-DC (black) at 180 °C.

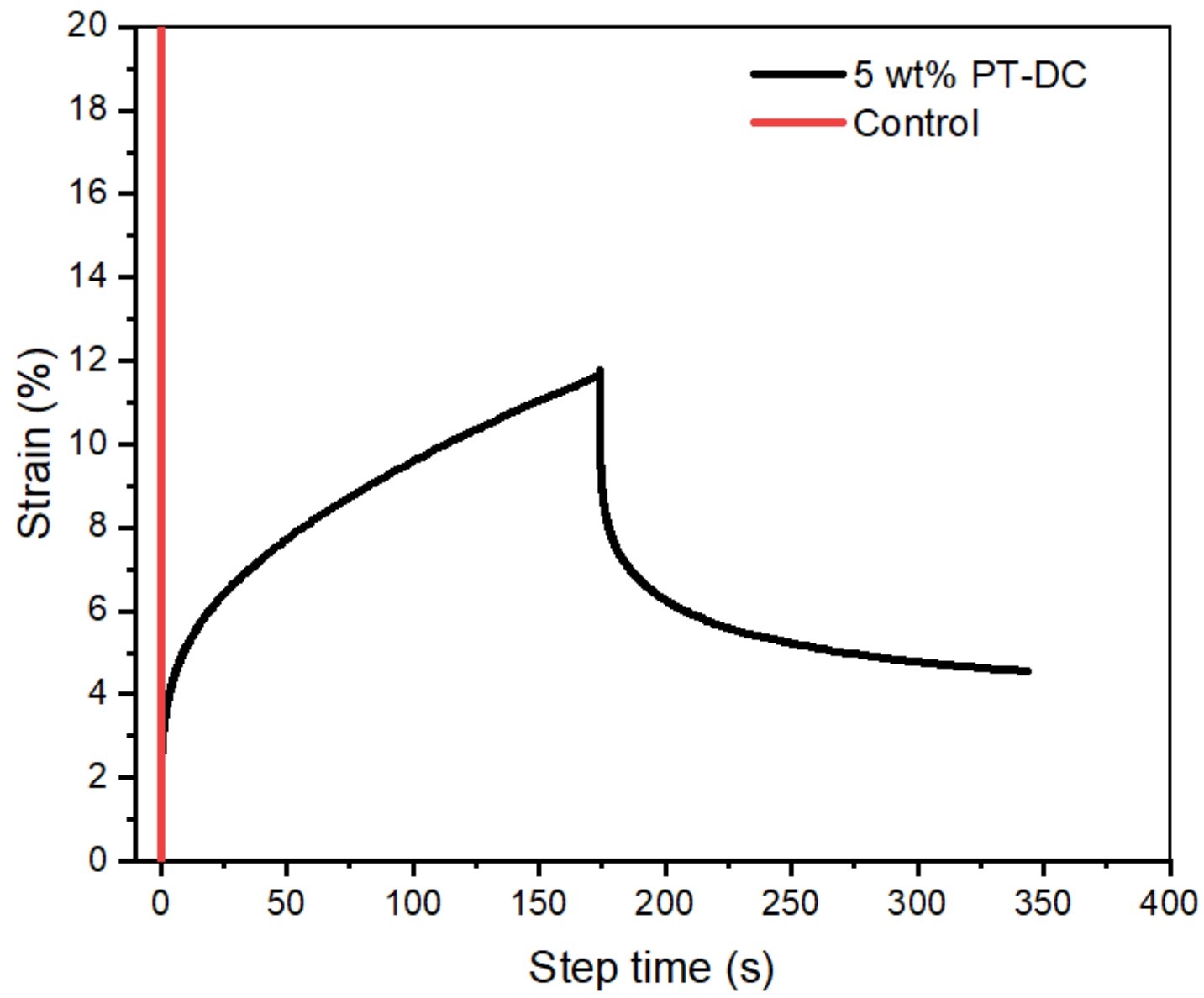

Figure S46. Creep recovery of quaternary mixed plastic waste blend control (red) and quaternary mixed plastic waste blend with 5 wt% PT-DC (black) at 270 °C.

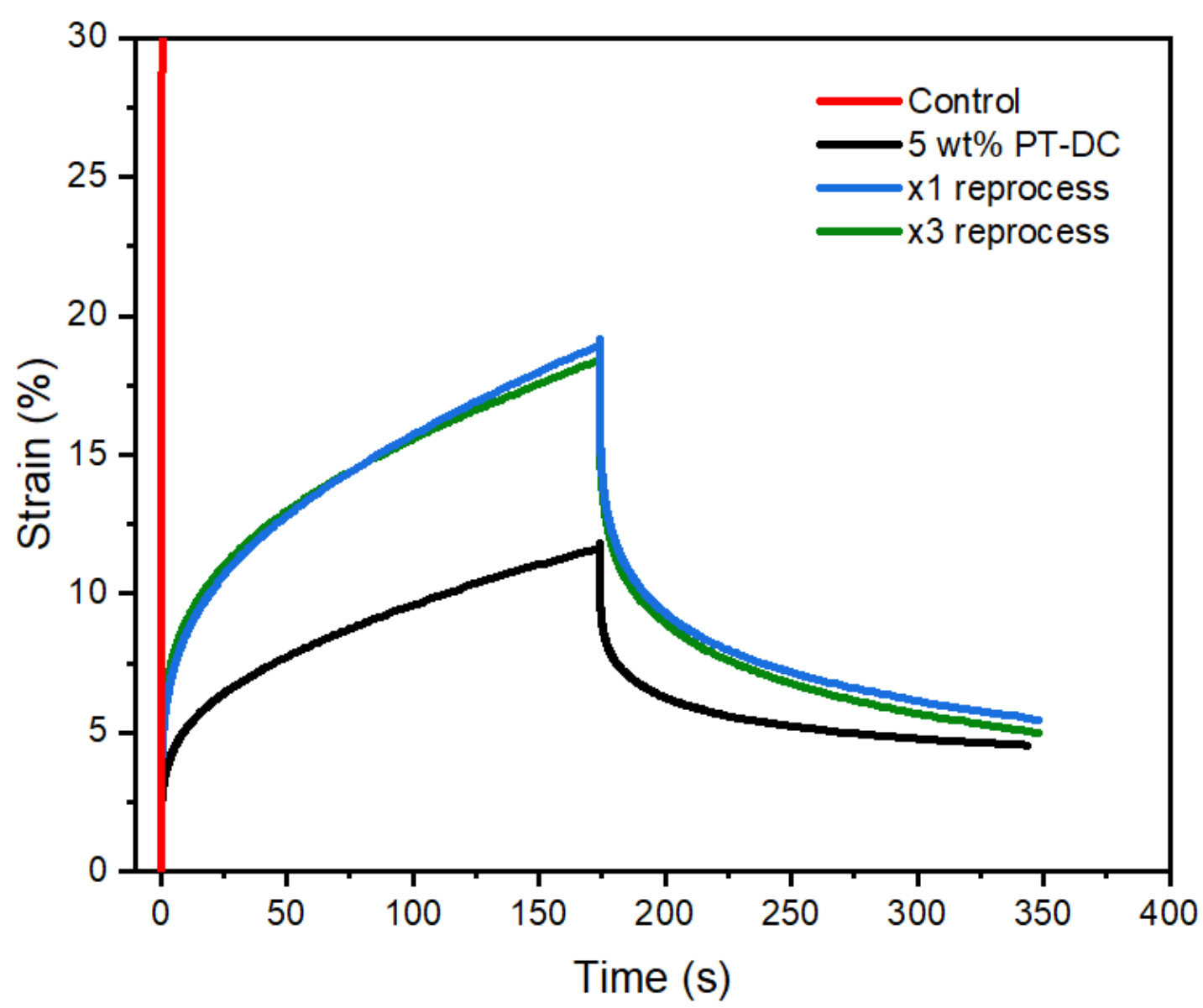

Figure S47. Creep recovery experiments of the quaternary blend and the compatibilized blend after multiple reprocessing cycles.

Frequency sweep

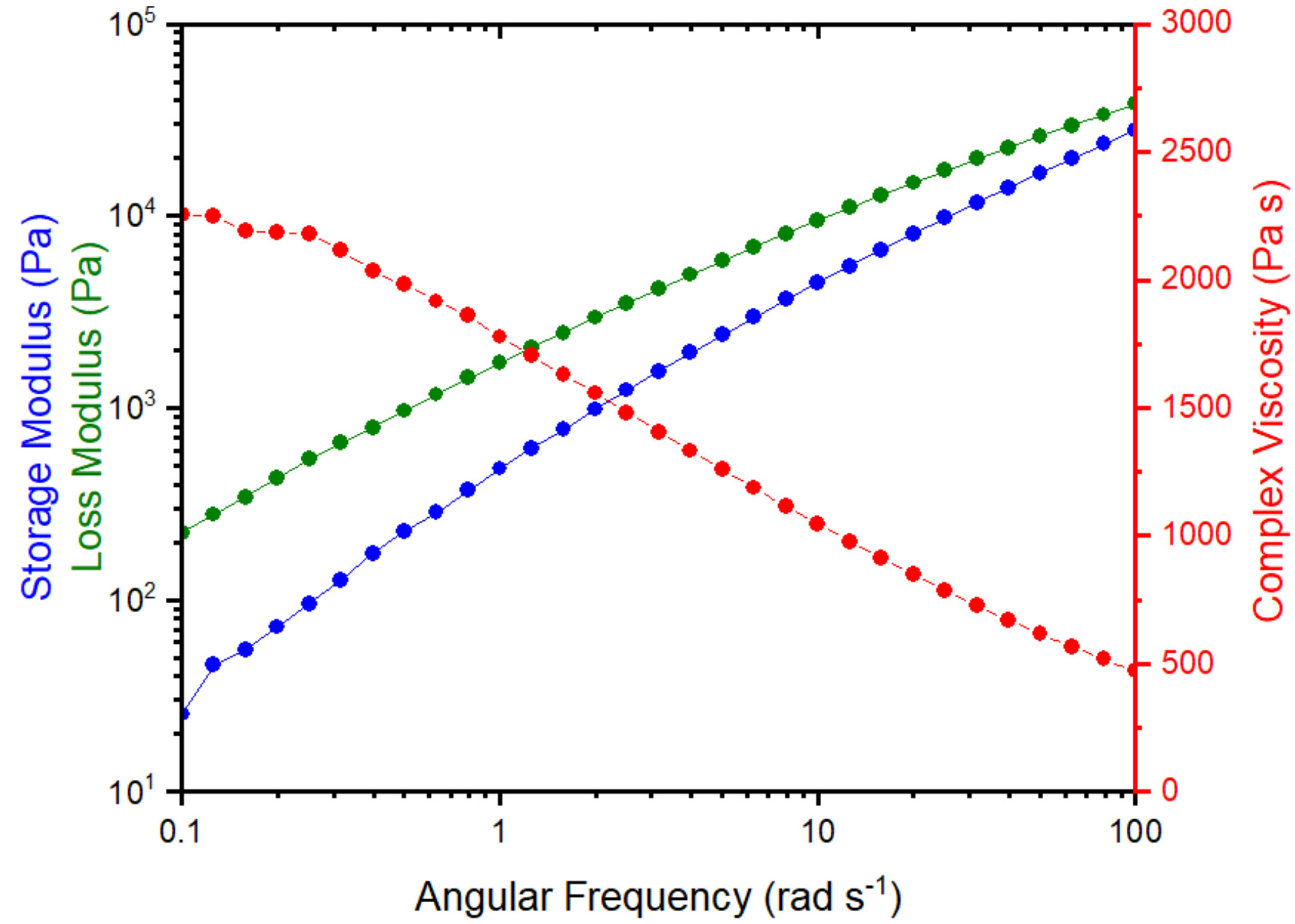

Figure S48. Frequency Sweep of HDPE control at 180 °C.

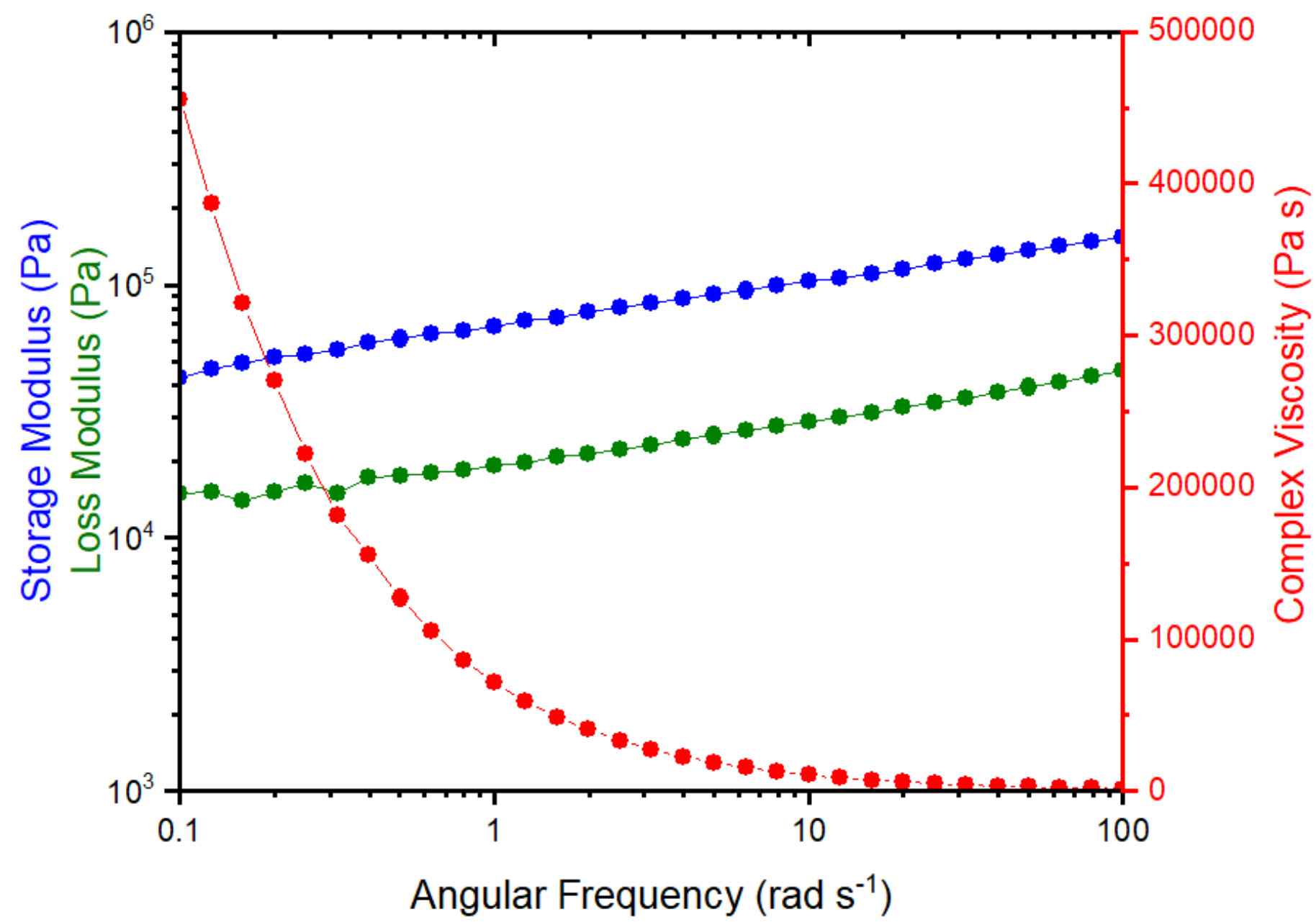

Figure S49. Frequency Sweep of HDPE with 5 wt% PT-DC at 180 °C.

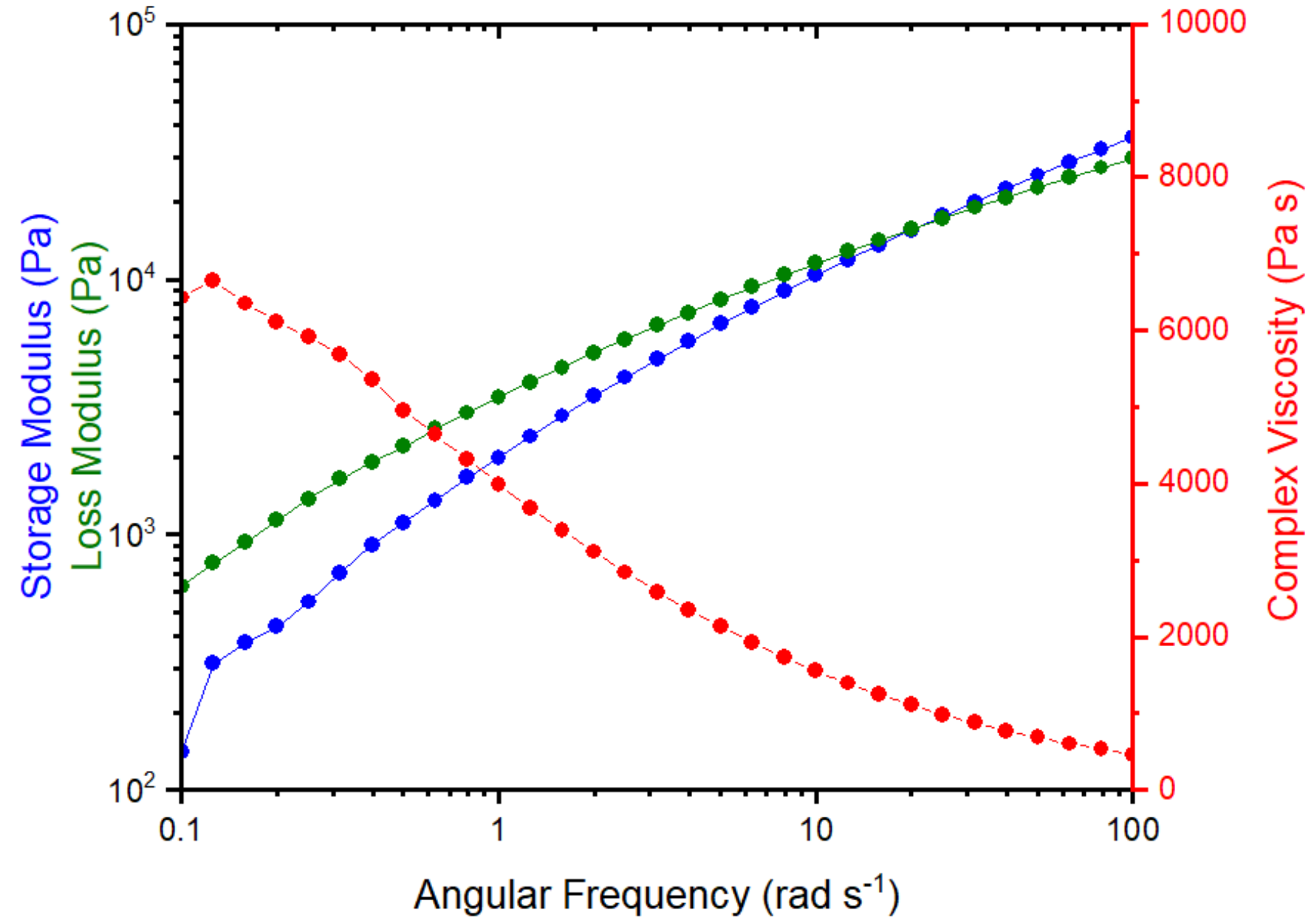

Figure S50. Frequency Sweep of LDPE control at 180 °C.

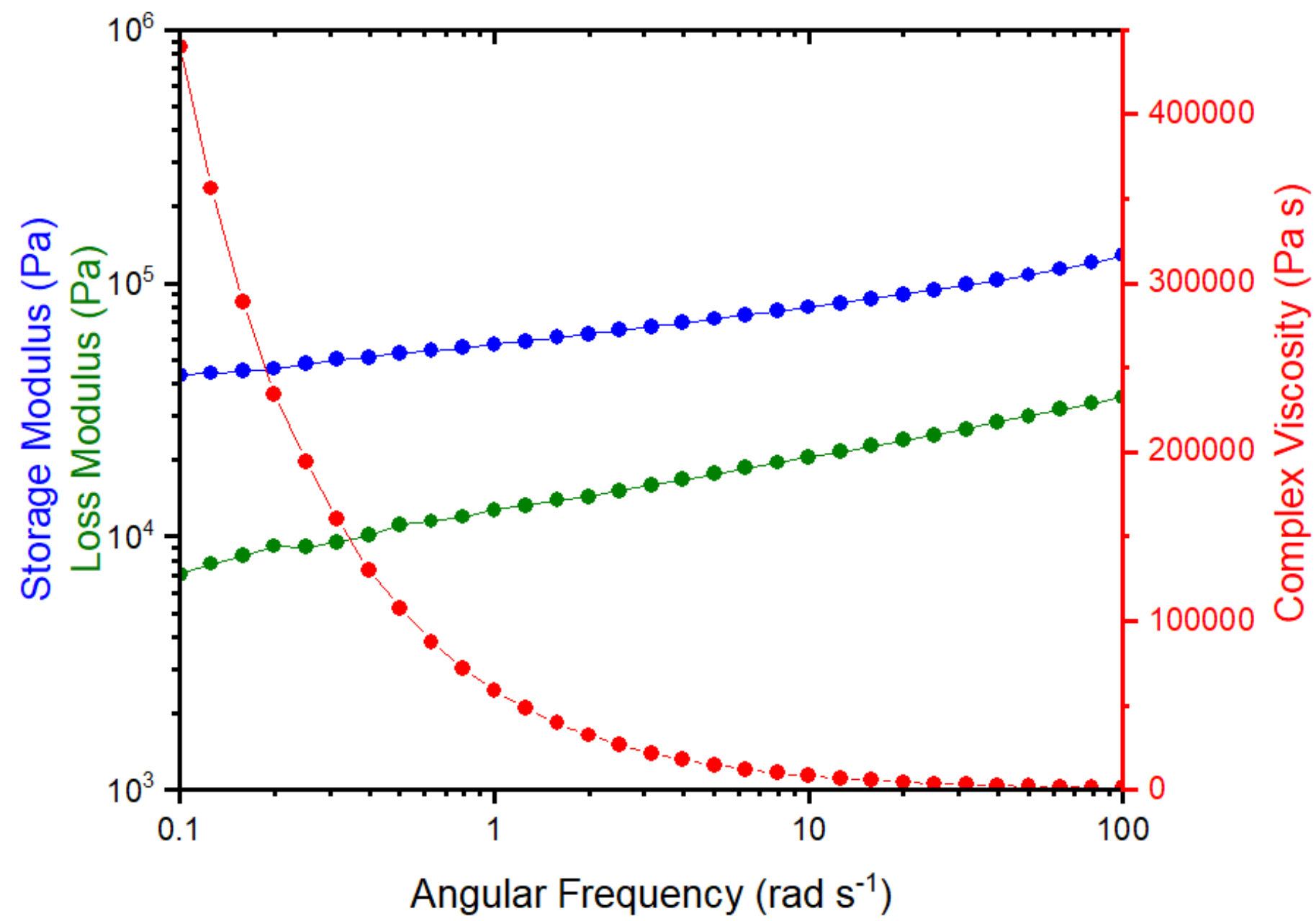

Figure S51. Frequency Sweep of LDPE with 5 wt% PT-DC at 180 °C.

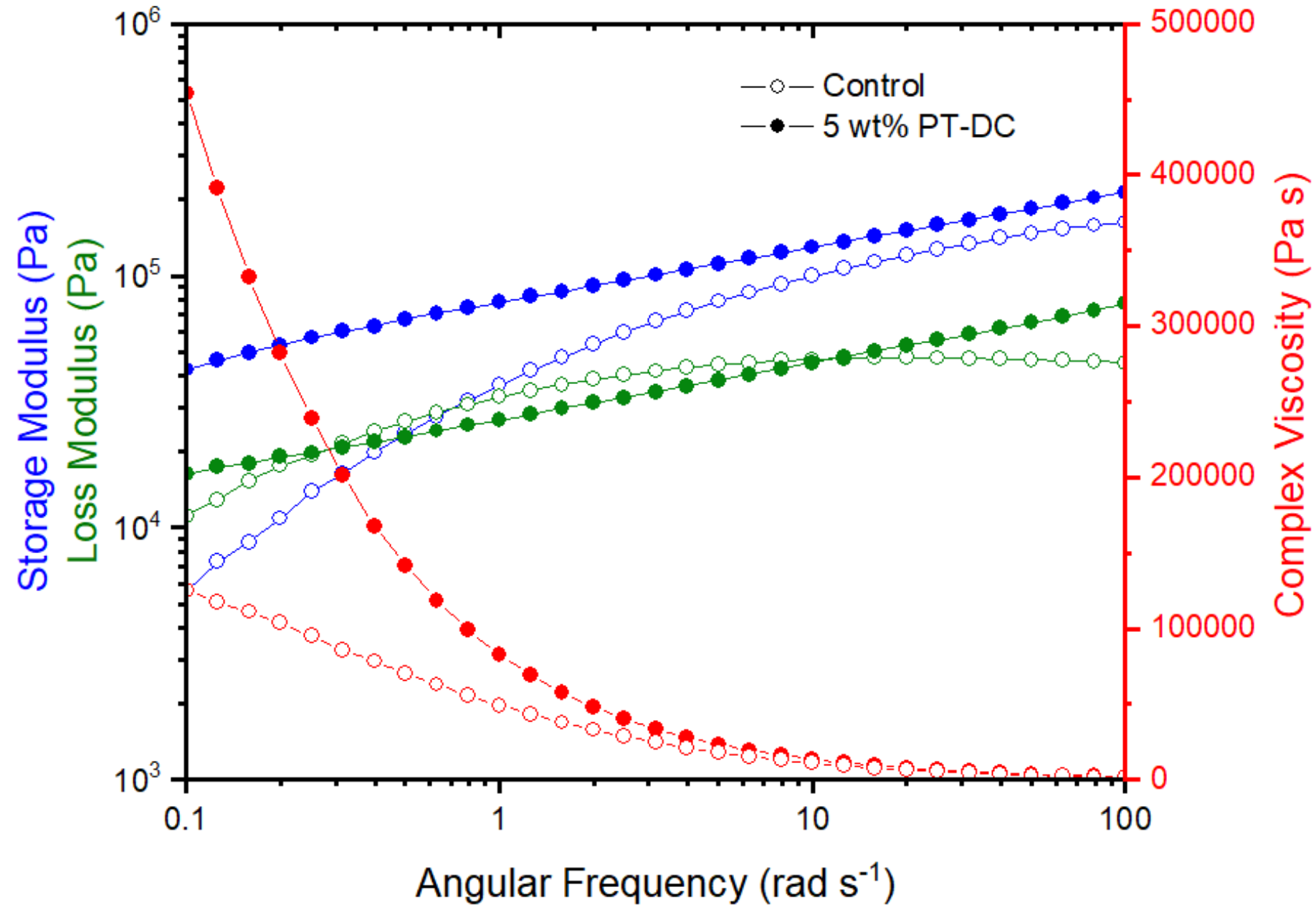

Figure S52. Frequency sweeps of PMMA control (unfilled spheres) and PMMA crosslinked with 5 wt% PT-DC (solid spheres) at 240 °C.

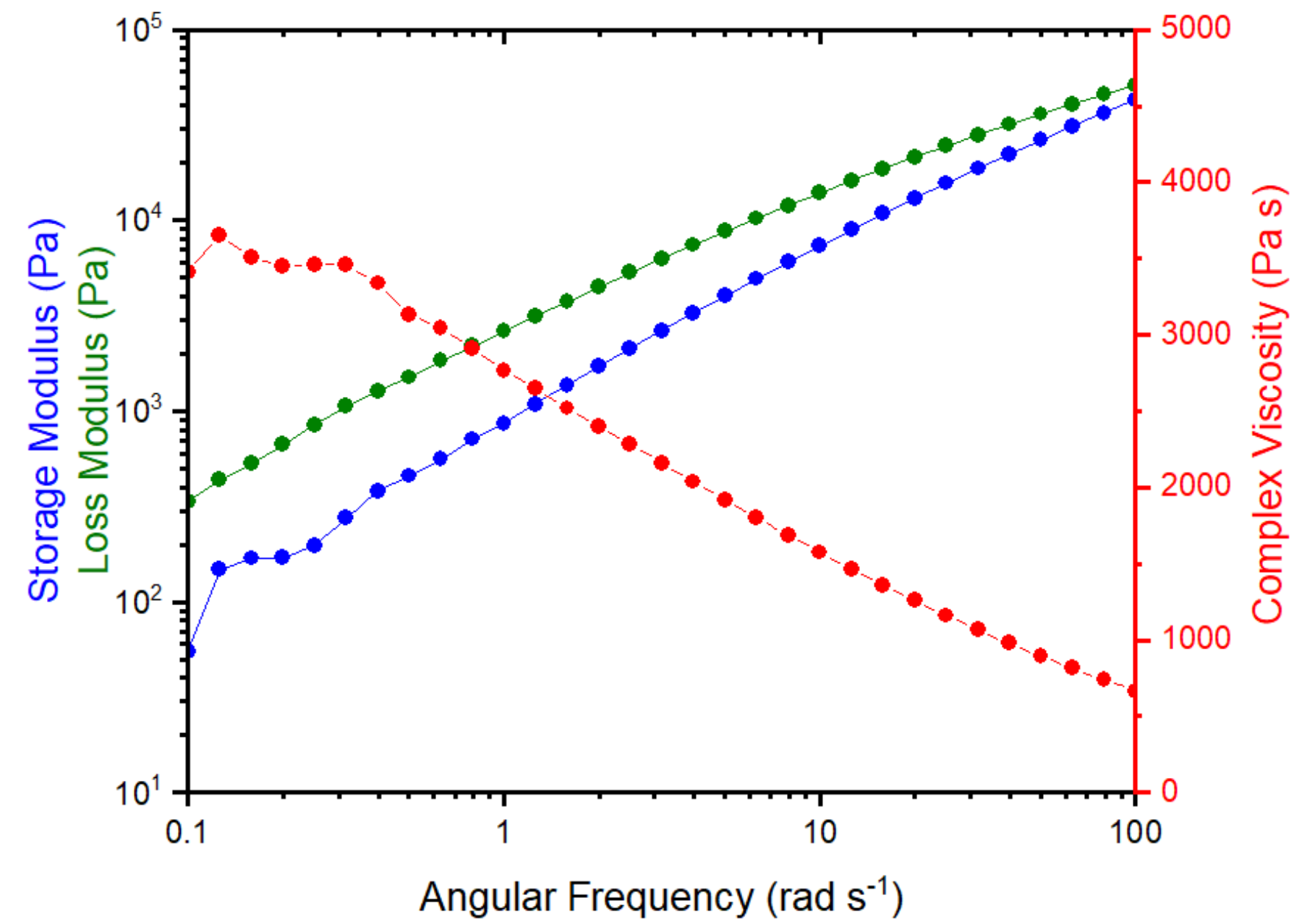

Figure S53. Frequency Sweep HDPE-*i*PP (70:30) waste control at 180 °C.

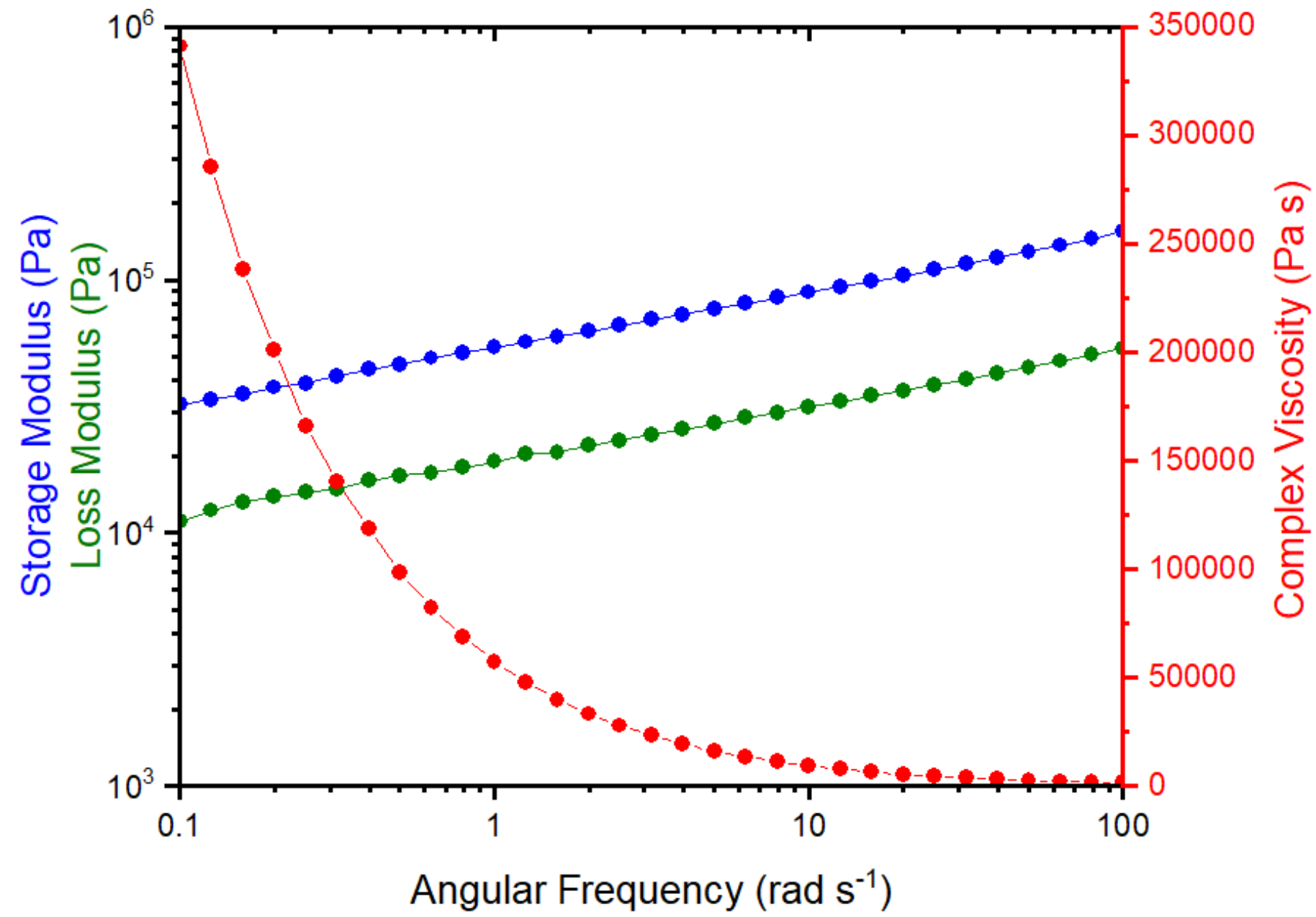

Figure S54. Frequency Sweep of HDPE-*i*PP (70:30) waste with 5 wt% PT-DC at 180 °C.

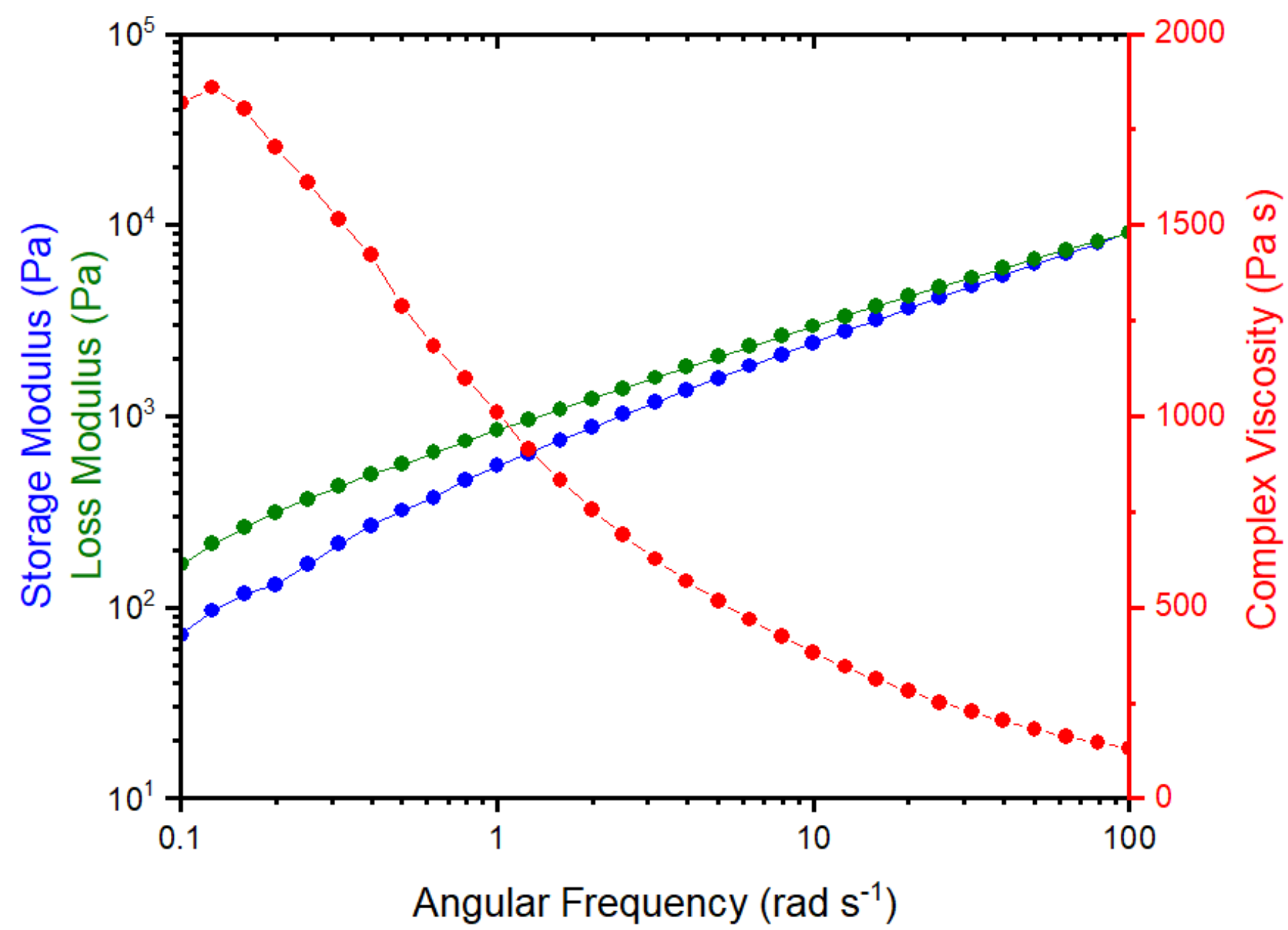

Figure S55. Frequency Sweep of LDPE-PET (80:20) blend control at 270 °C.

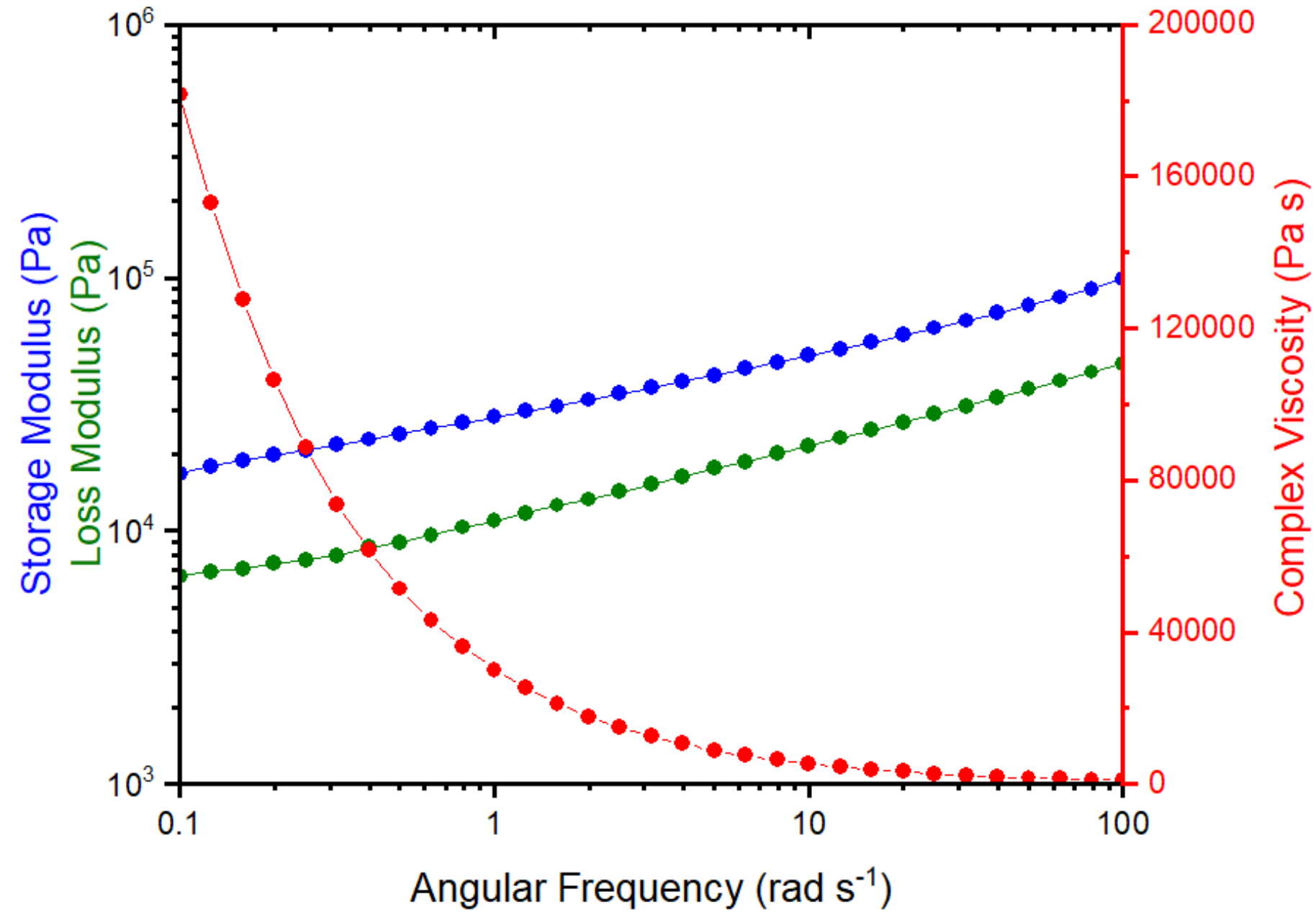

Figure S56. Frequency Sweep of LDPE-PET (80:20) with 5 wt% PT-DC at 270 °C.

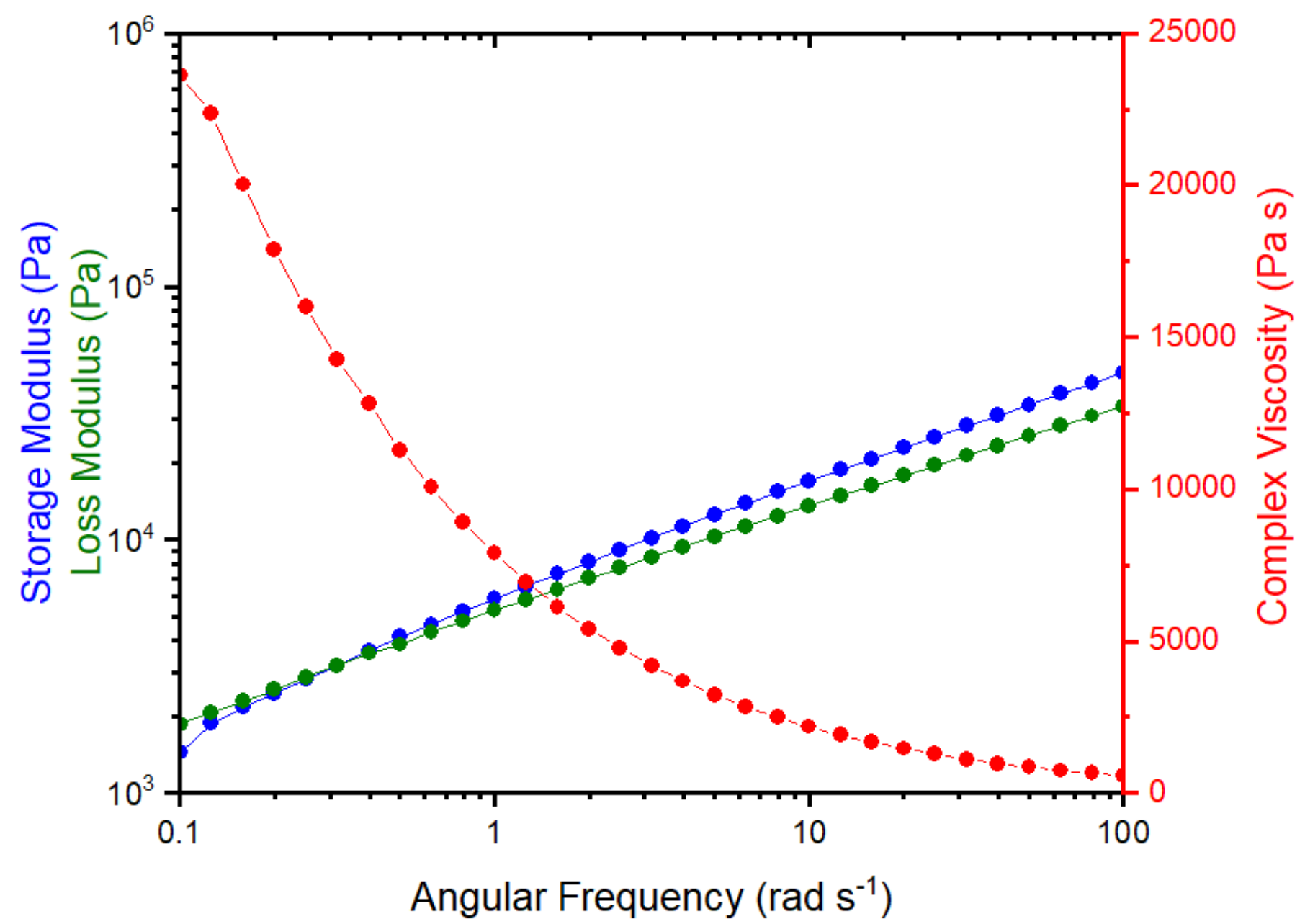

Figure S57. Frequency Sweep of HDPE-PET (80:20) blend control at 270 °C.

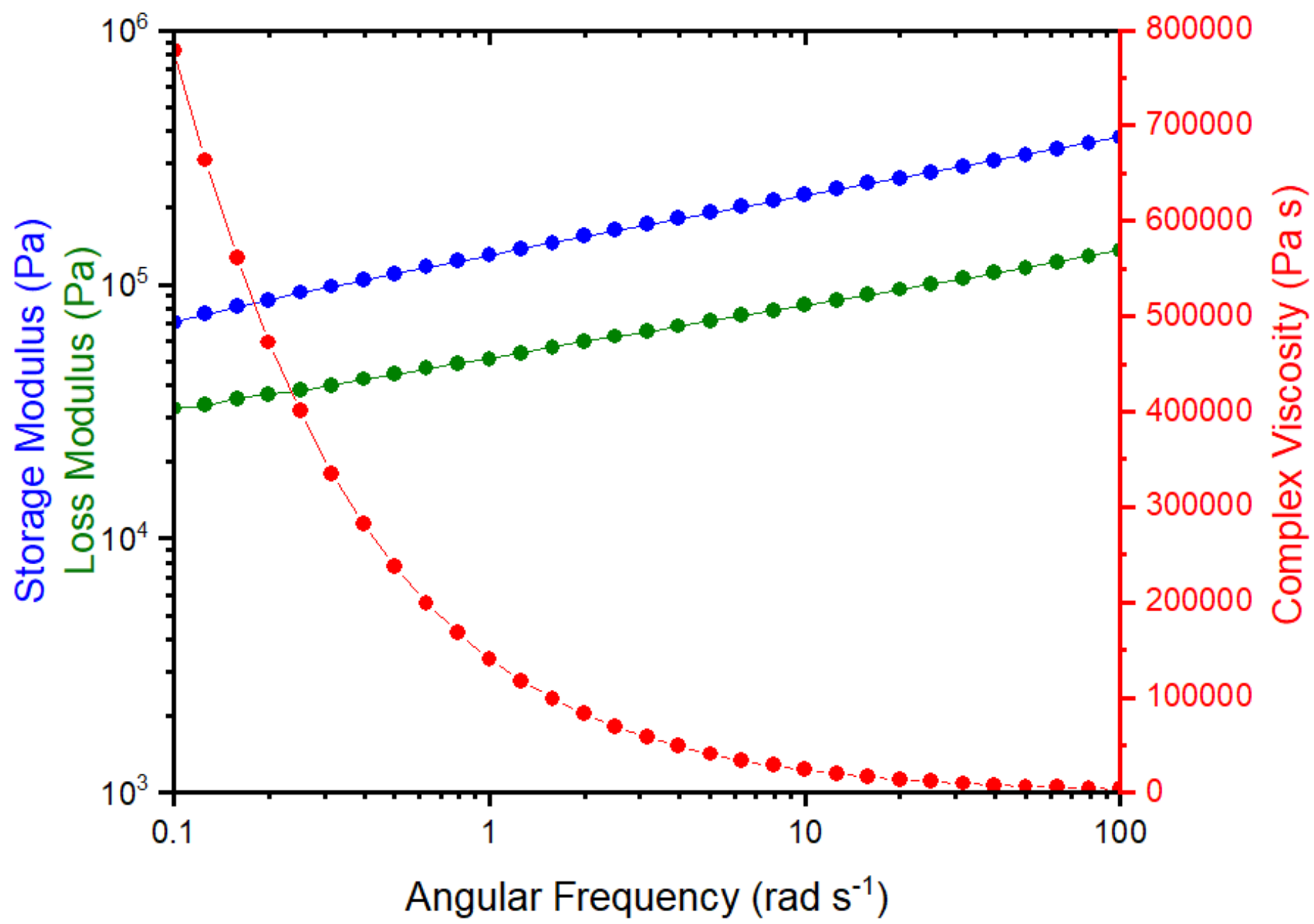

Figure S58. Frequency Sweep of HDPE-PET (80:20) with 5 wt% PT-DC at 270 °C.

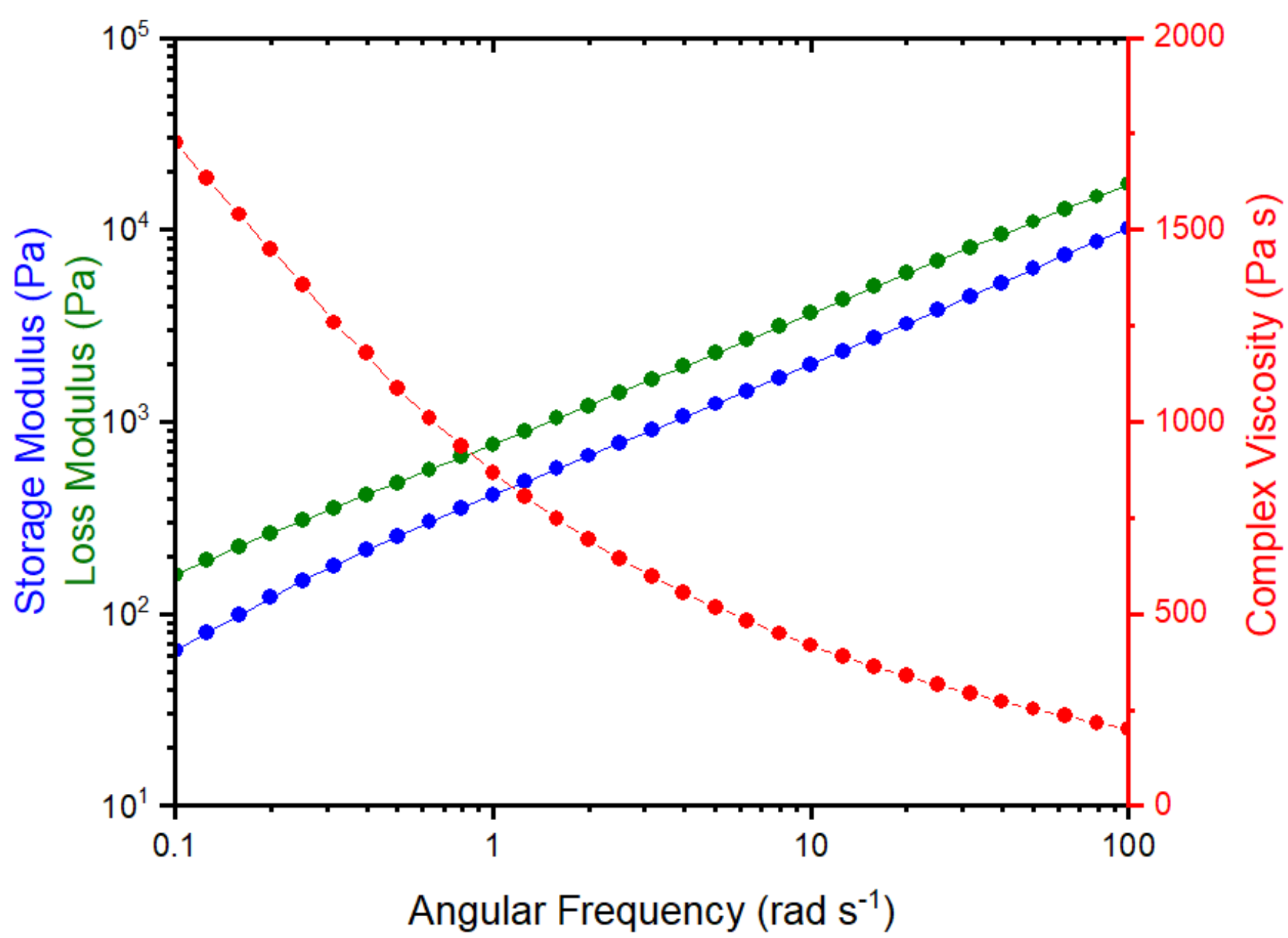

Figure S59. Frequency Sweep of HDPE-Nylon 6 (80:20) blend control at 240 °C.

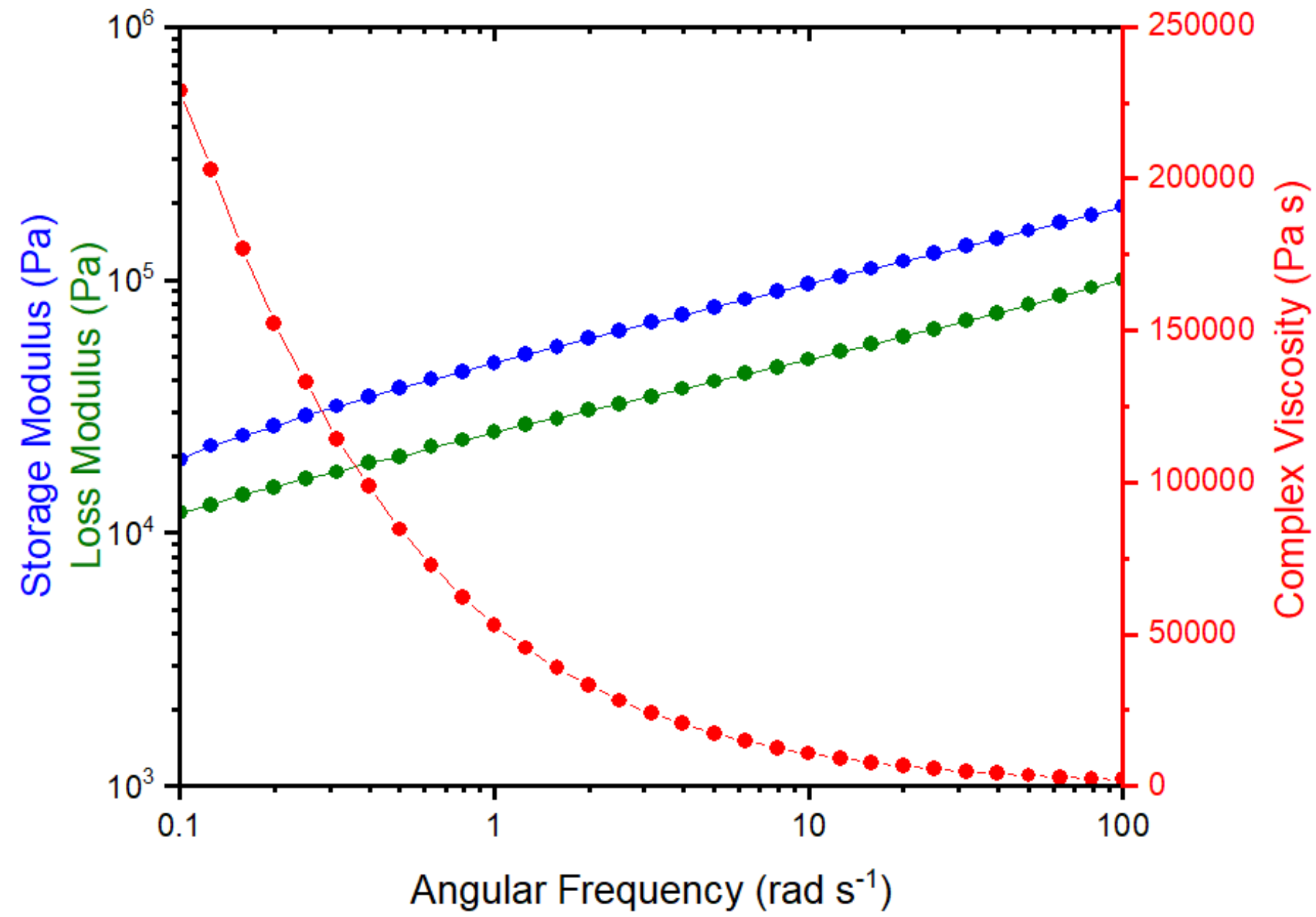

Figure S60. Frequency Sweep of HDPE-Nylon 6 (80:20) with 5 wt% PT-DC at 240 °C.

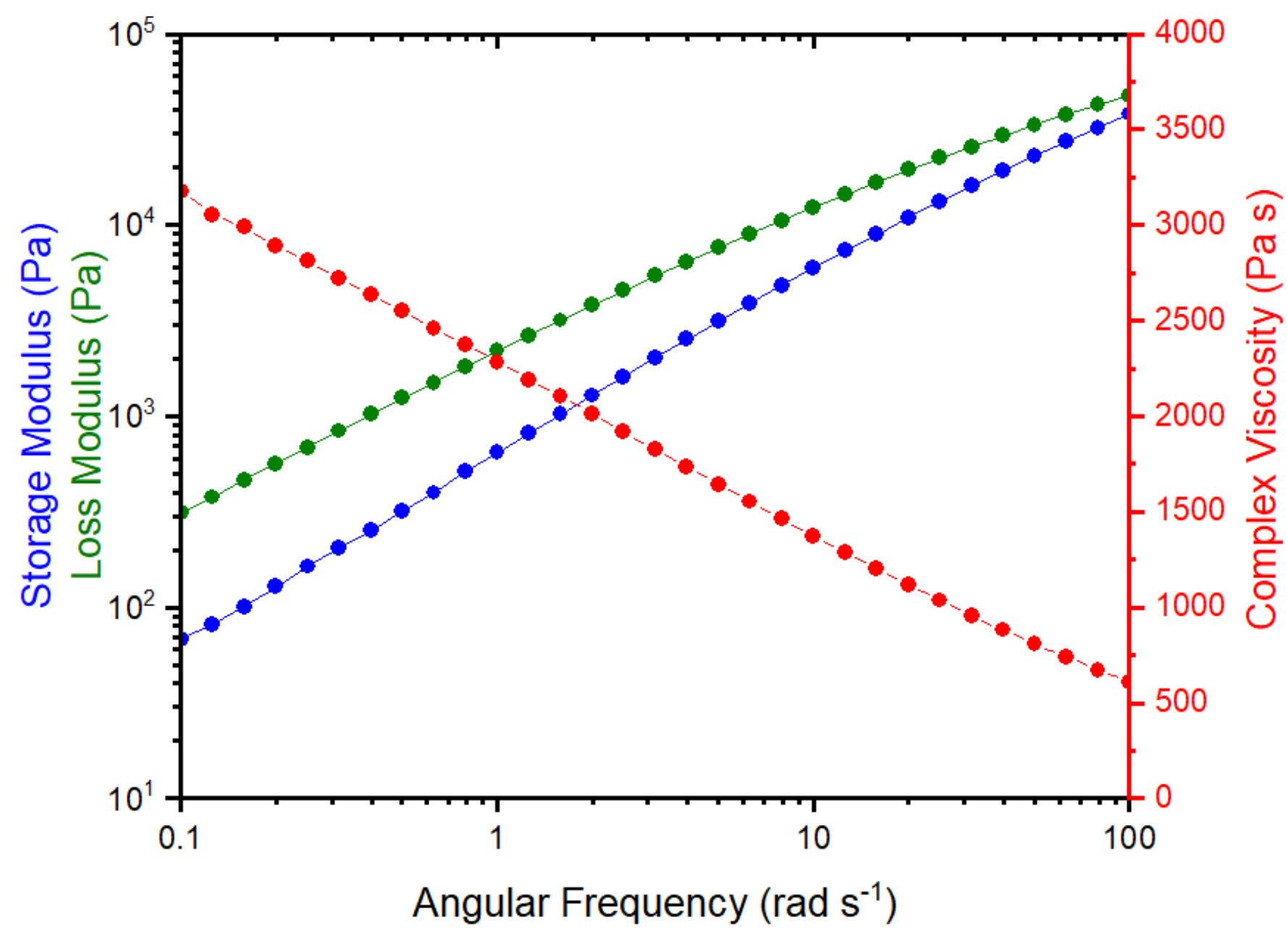

Figure S61. Frequency Sweep of ternary waste blend control at 180 °C.

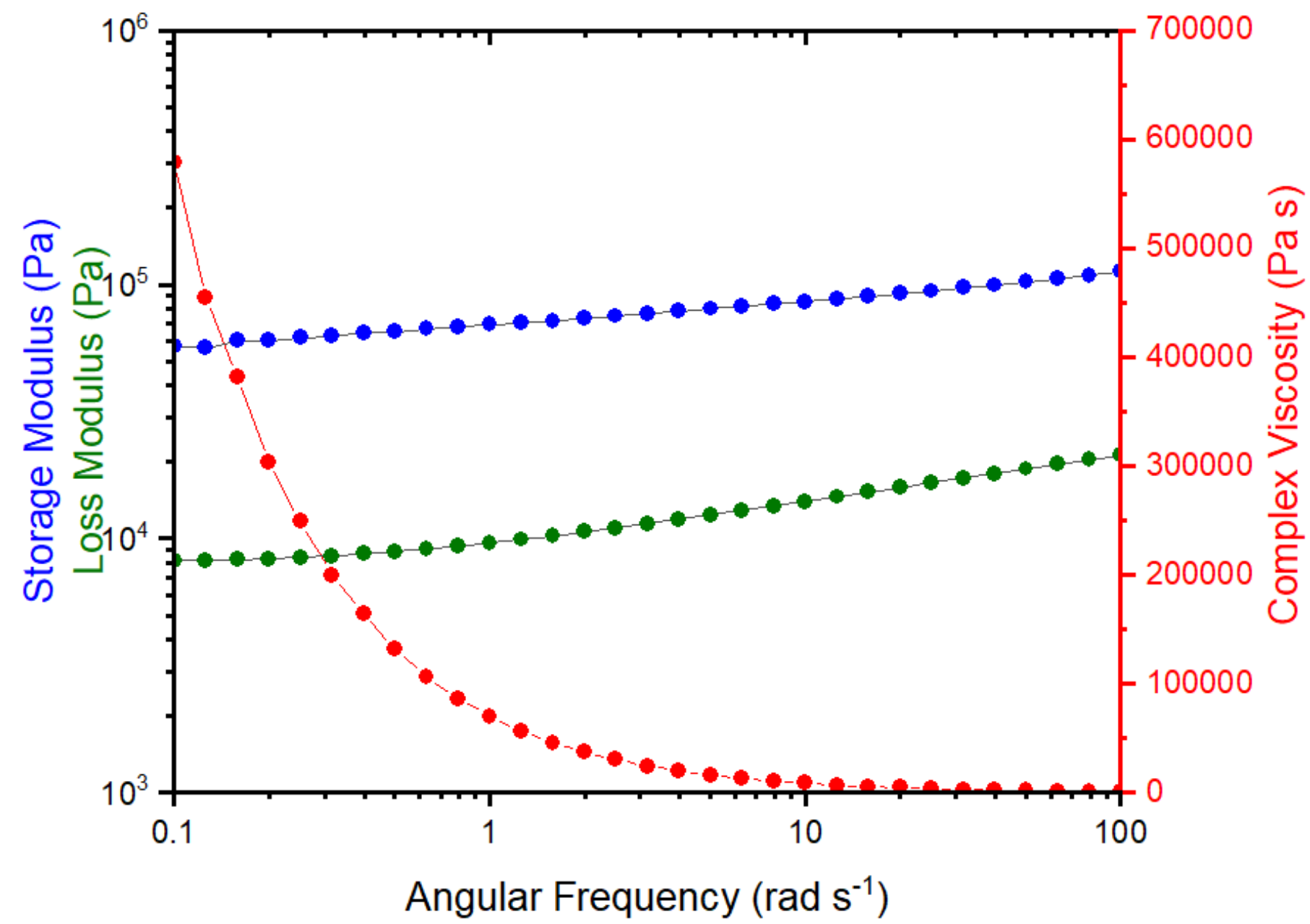

Figure S62. Frequency Sweep of ternary mixed plastic waste blend with 5 wt% PT-DC at 240 °C.

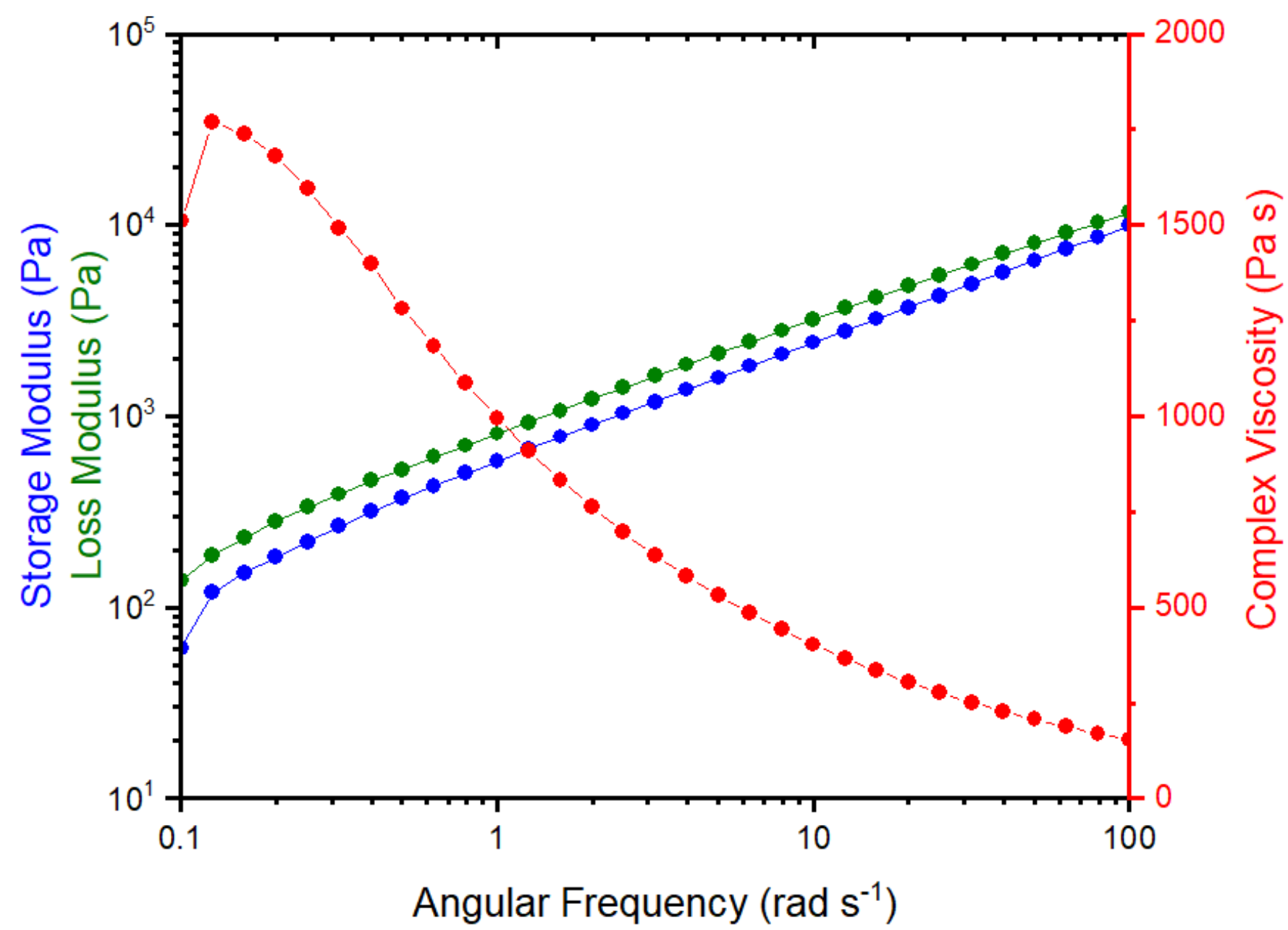

Figure S63. Frequency Sweep of quaternary waste plastic blend control at 270 °C.

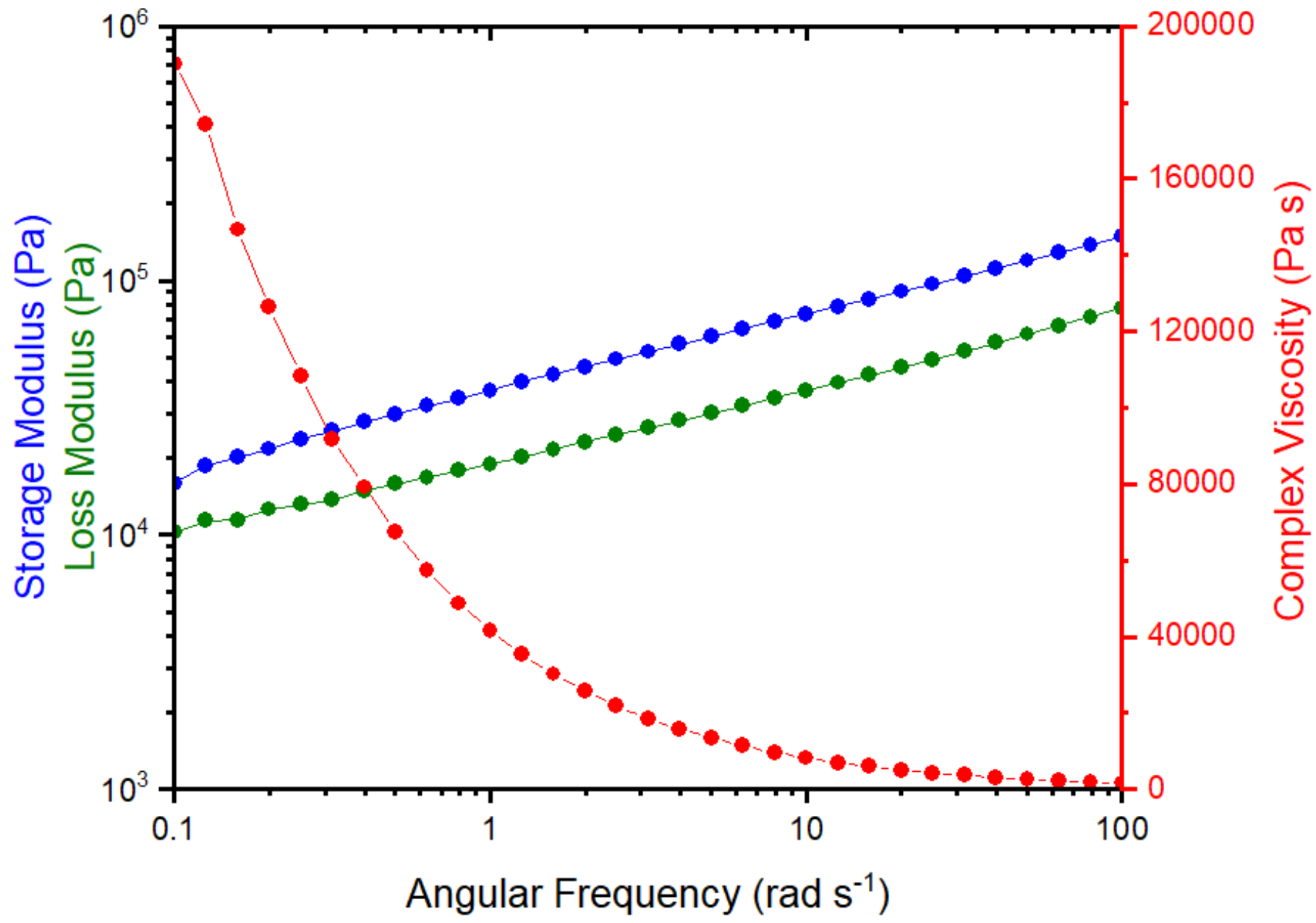

Figure S64. Frequency Sweep of quaternary mixed plastic waste blend with 5 wt% PT-DC at 270 °C.

Temperature Sweep

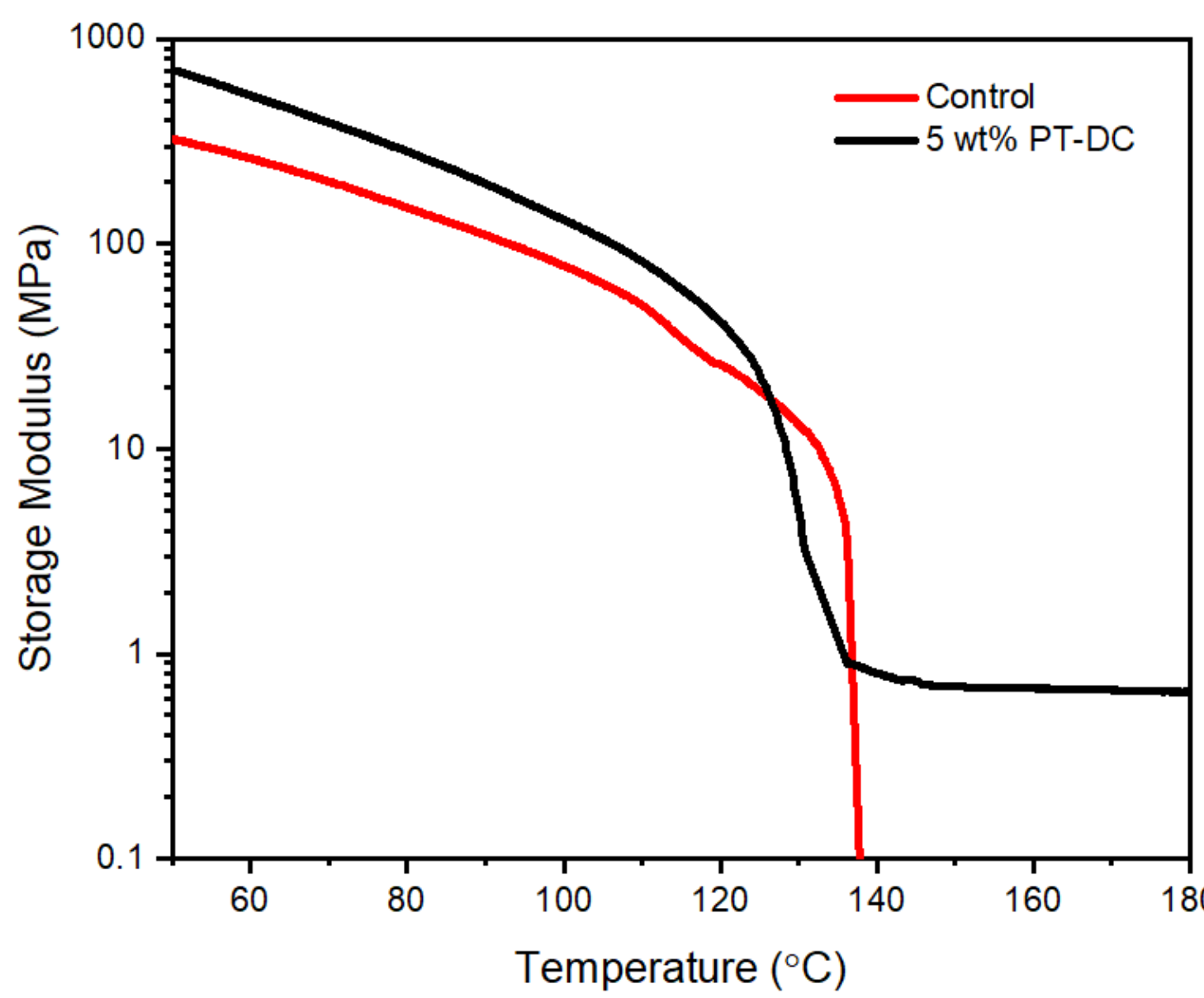

Figure S65. Temperature Sweep of HDPE control (red) and HDPE with 5 wt% of the PT-DC (black).

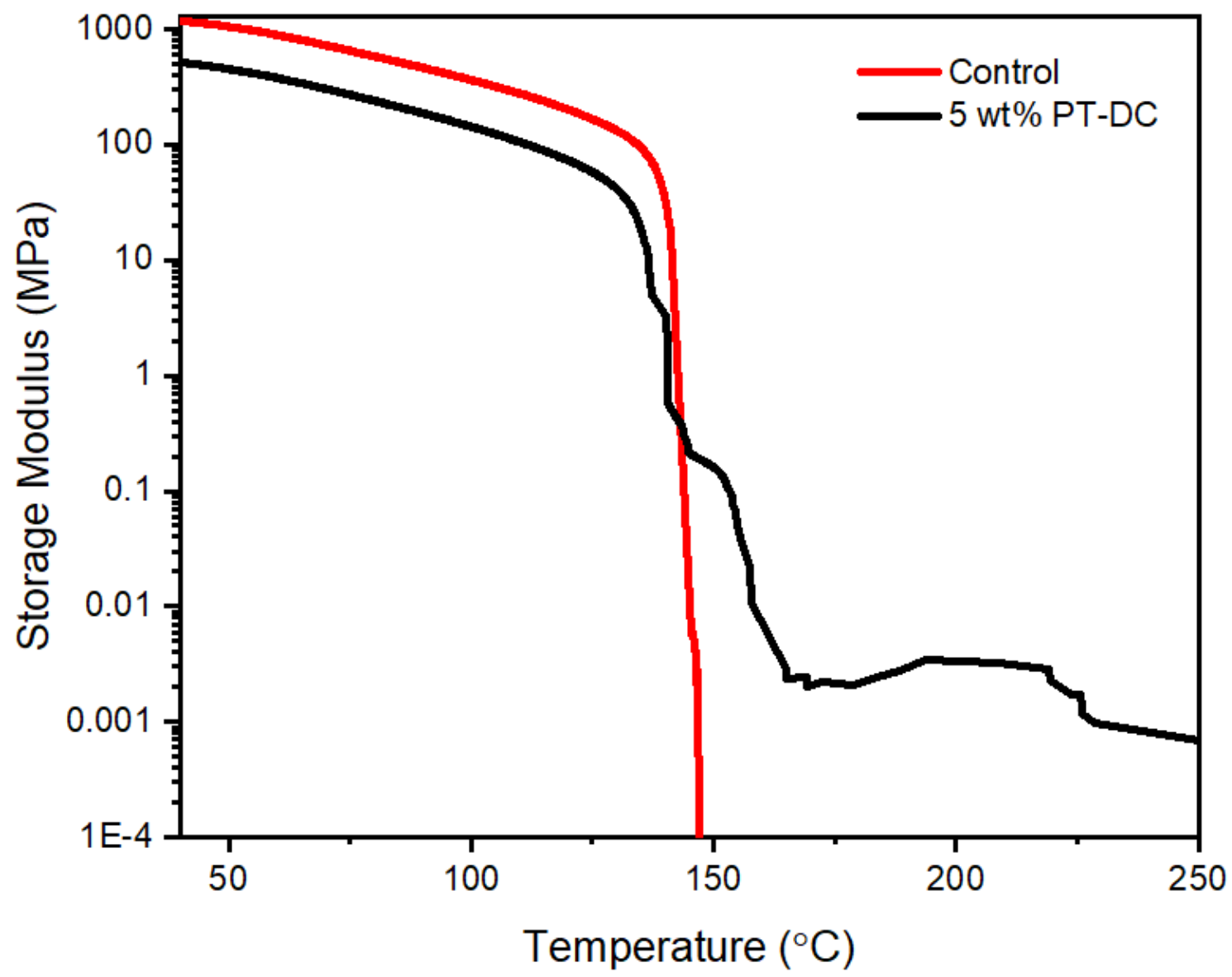

Figure S66. Temperature Sweep of HDPE-Nylon 6 (80:20) control (red) and HDPE-Nylon 6 (80:20) with 5 wt% of the PT-DC (black).

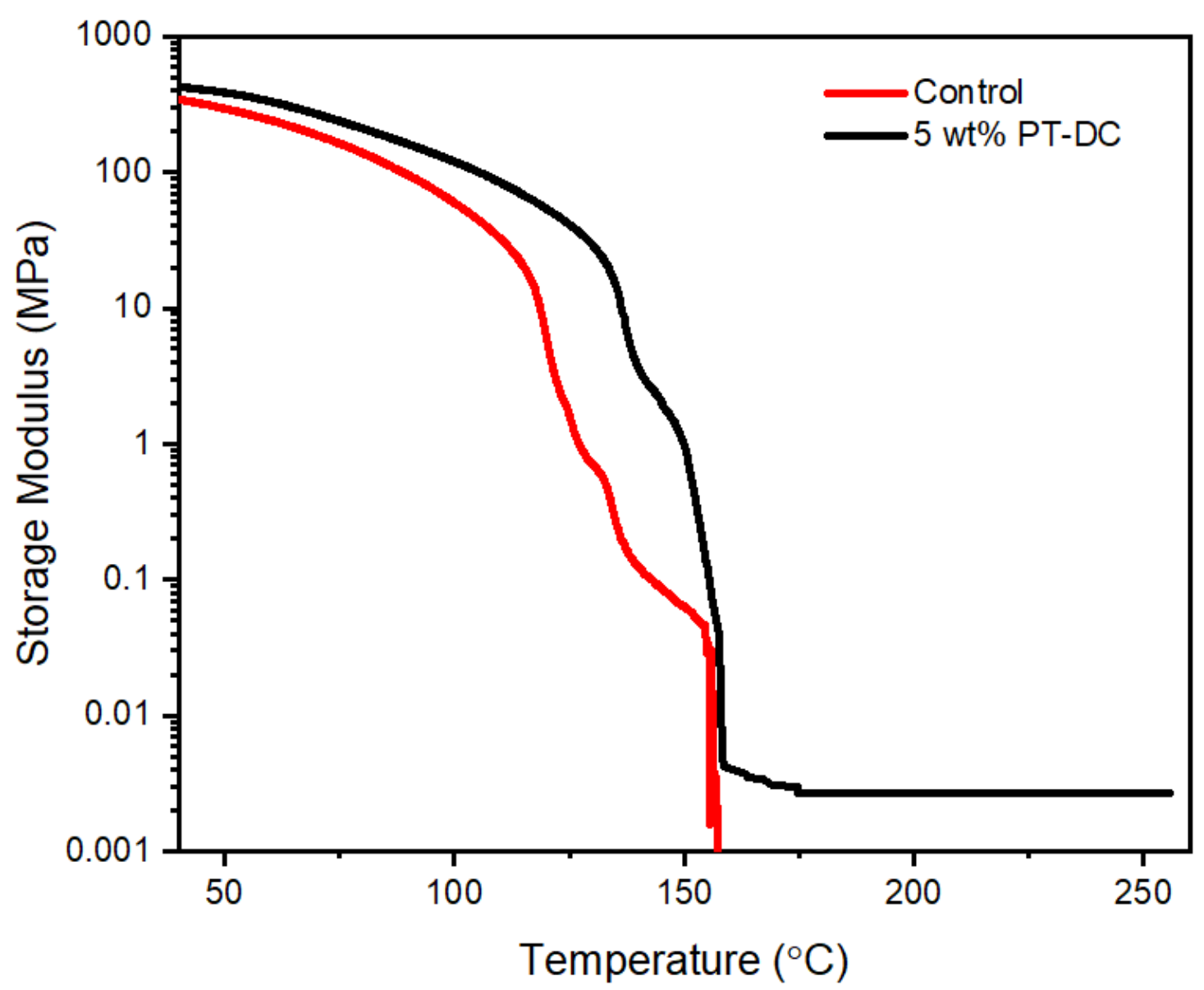

Figure S67. Temperature Sweep of HDPE-PET (80:20) control (red) and HDPE-PET (80:20) with 5 wt% of the PT-DC (black).

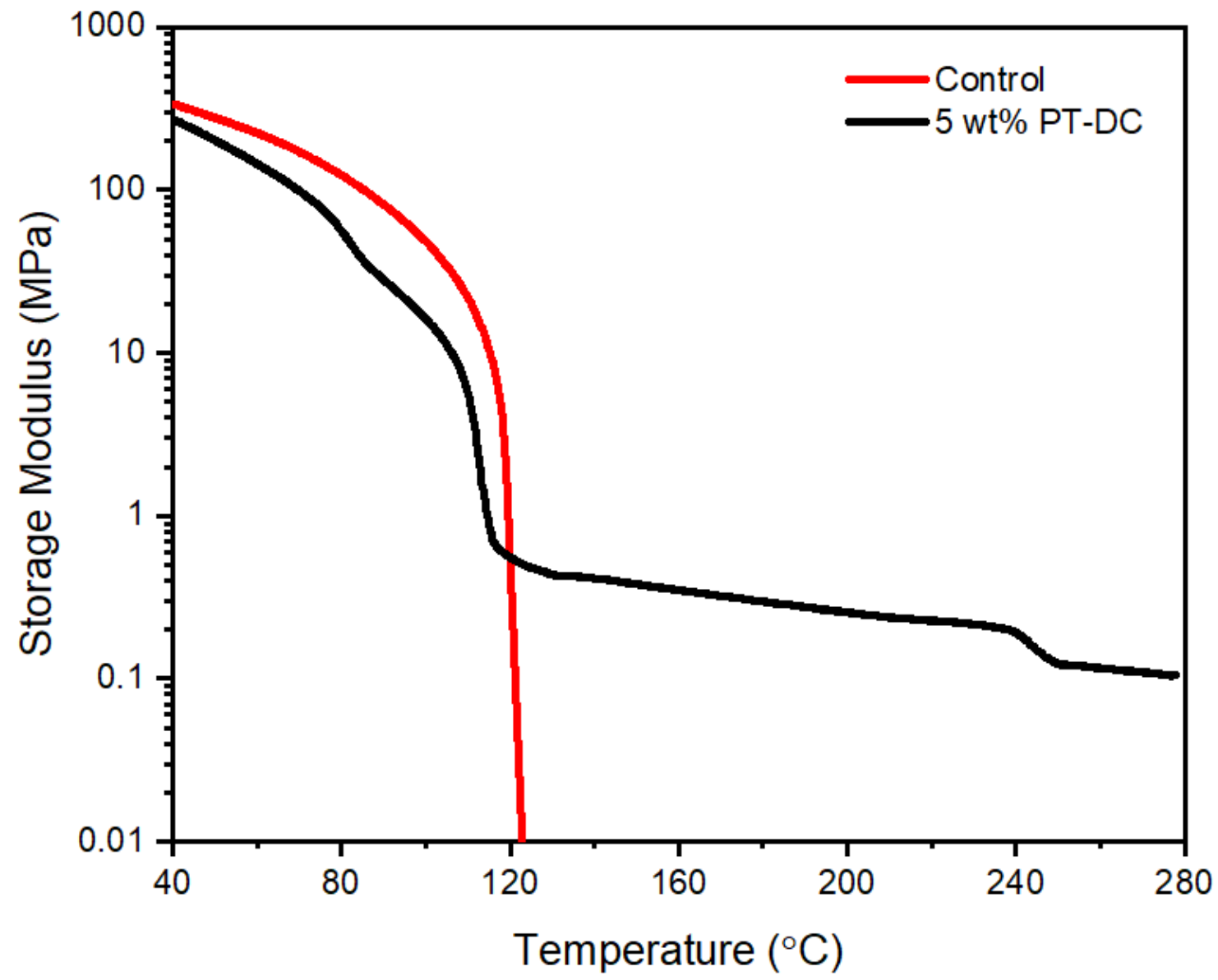

Figure S68. Temperature Sweep of LDPE-PET (80:20) control (red) and LDPE-PET (80:20) with 5 wt% of the PT-DC (black).

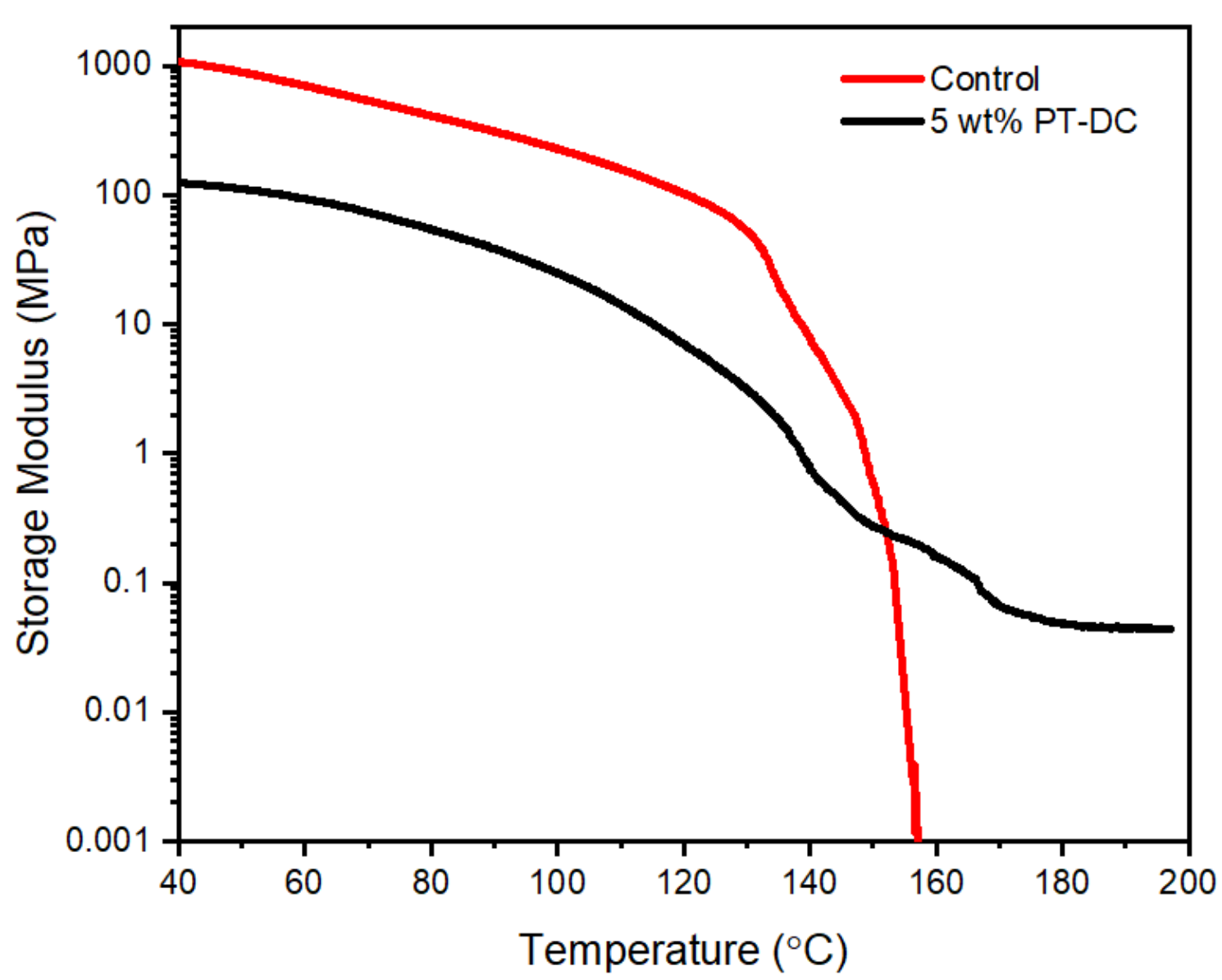

Figure S69. Temperature Sweep of ternary mixed plastic waste blend control (red) and ternary mixed plastic waste blend with 5 wt% PT-DC (black)

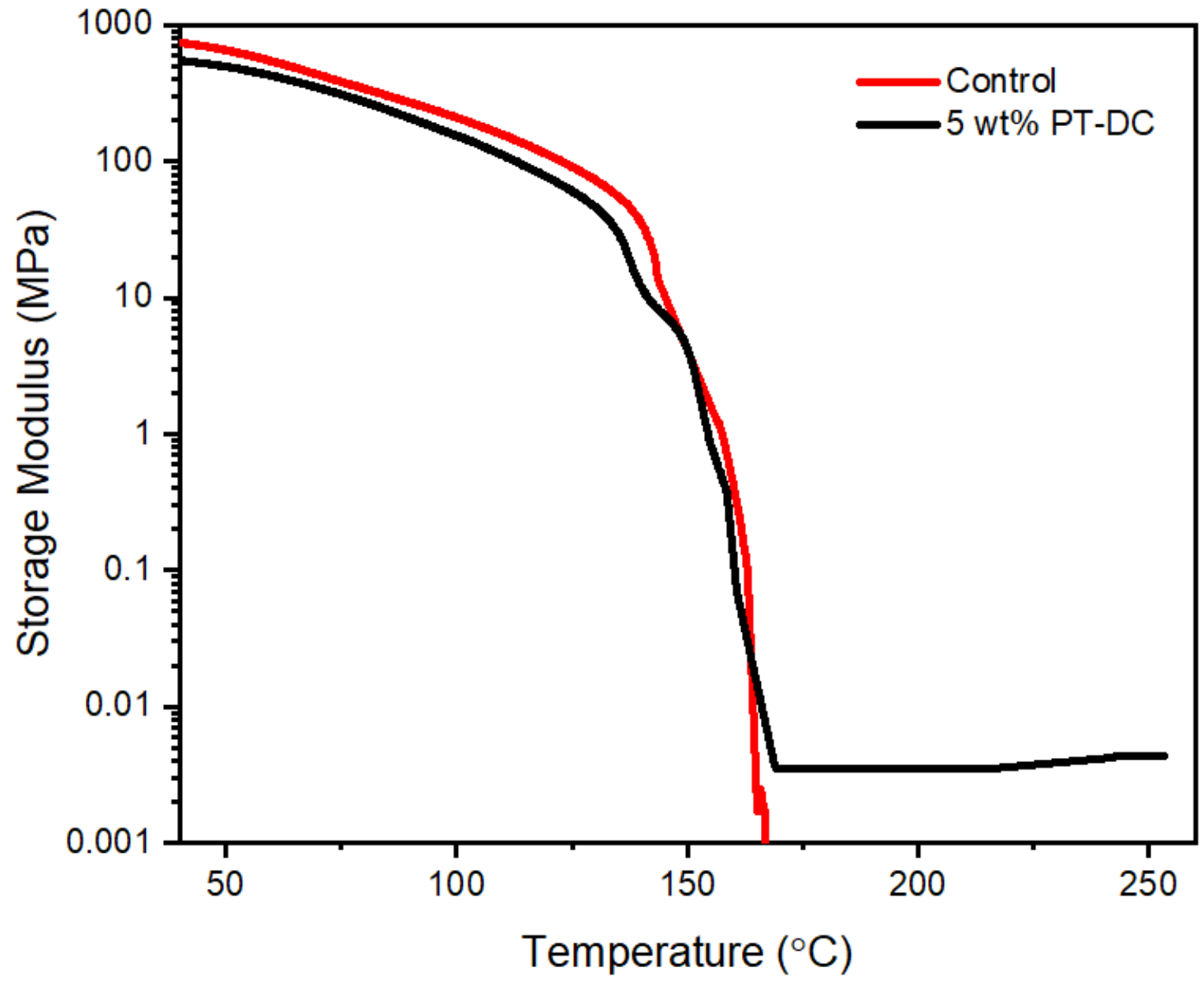

Figure S70. Temperature Sweep of quaternary mixed plastic waste blend control (red) and quaternary mixed plastic waste blend with 5 wt% PT-DC (black)

Tensile

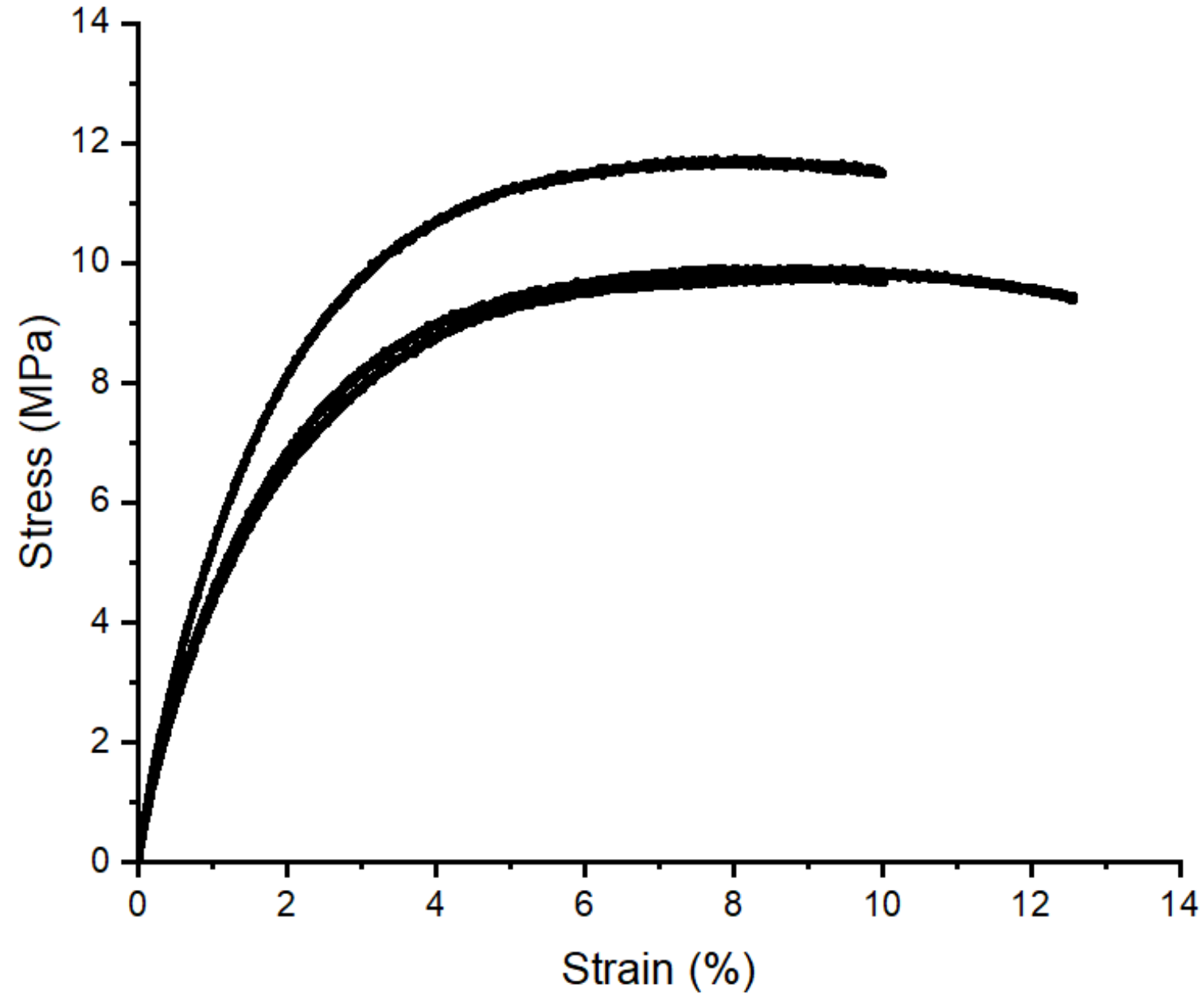

Figure S71. Uniaxial tensile testing of LDPE-PET (80:20) control blend.

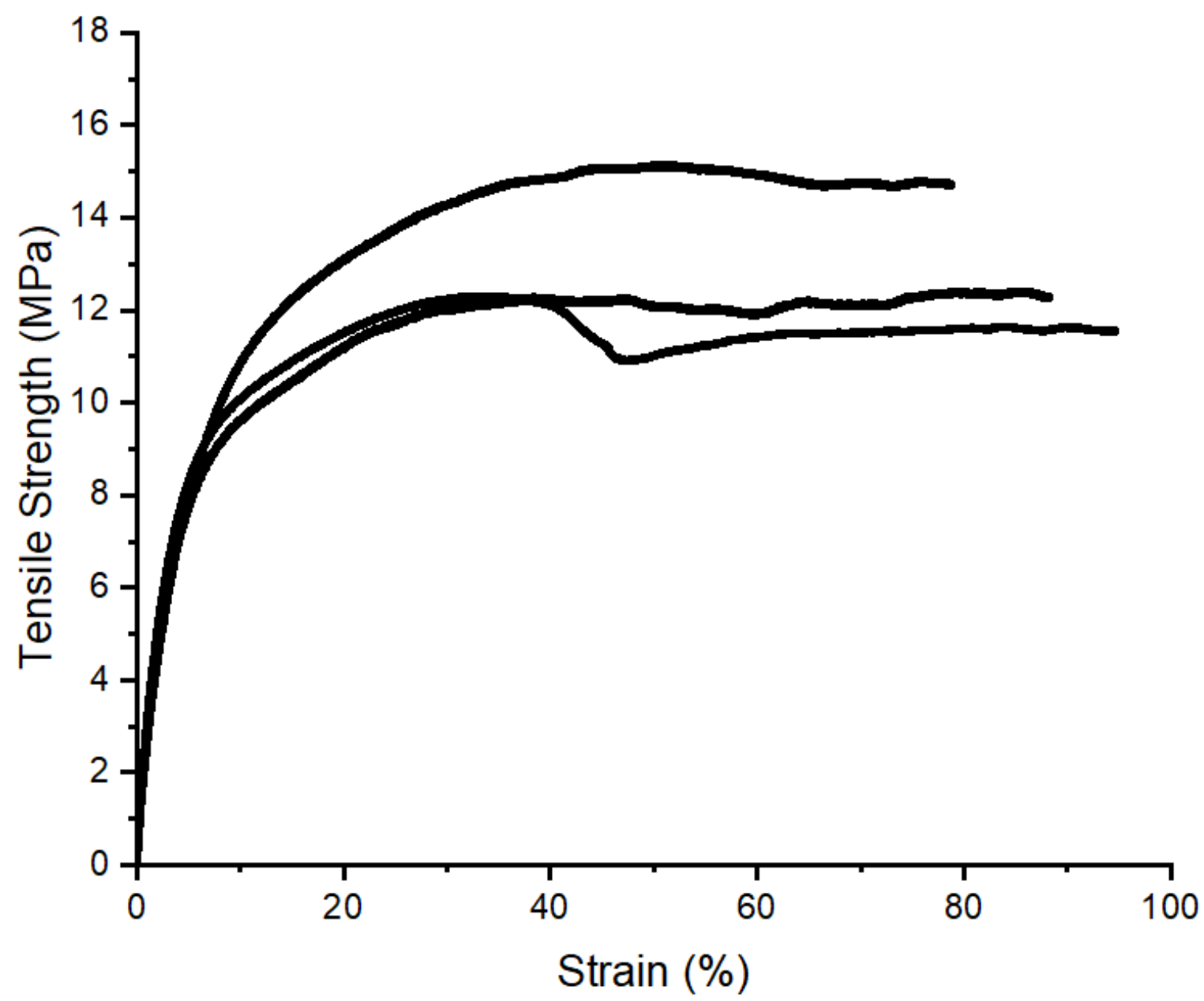

Figure S72. Uniaxial tensile testing of LDPE-PET (80:20) blend with 5 wt% PT-DC.

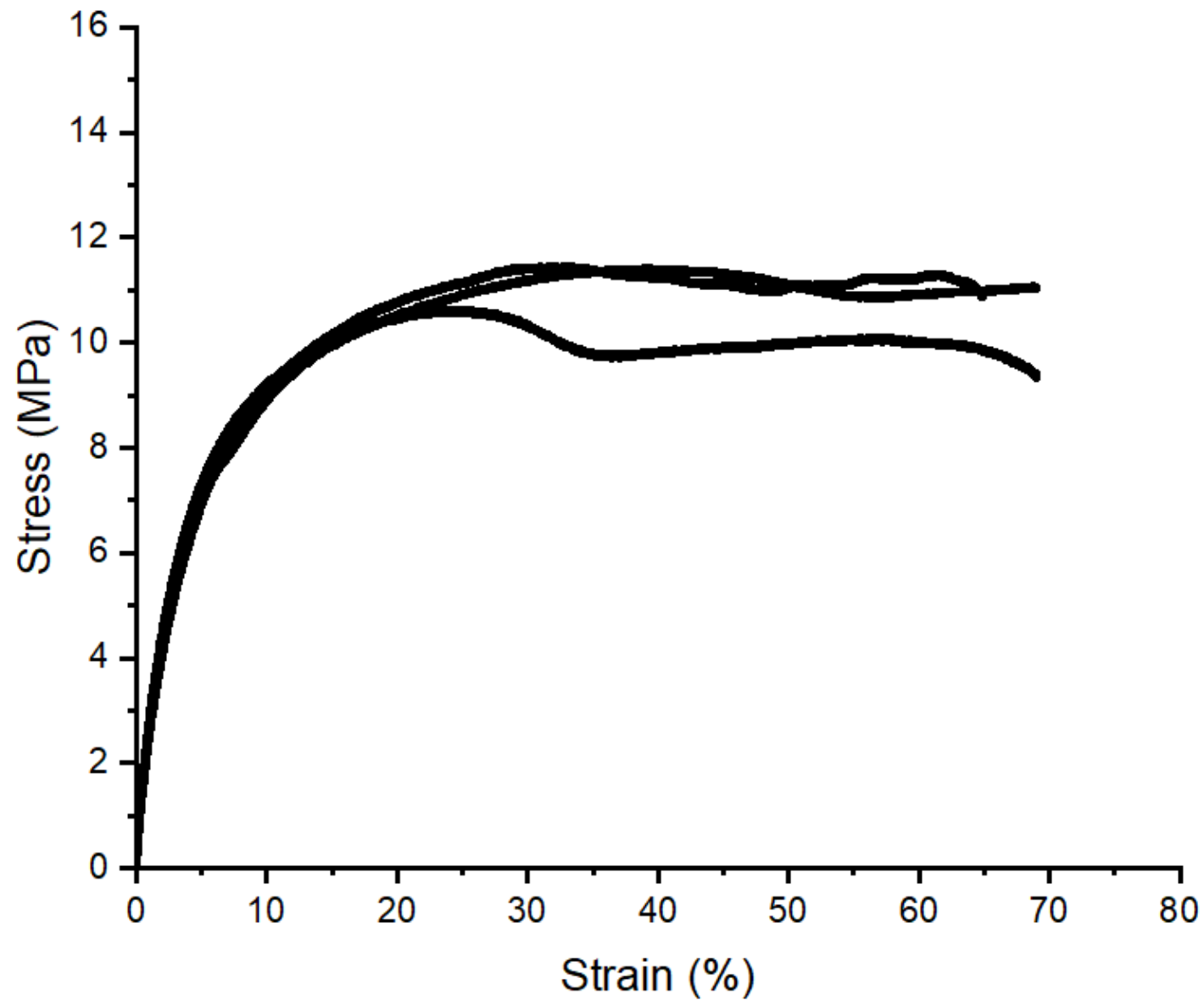

Figure S73. Uniaxial tensile stress-strain curves shown in triplicates: LDPE-PET (80:20) blend treated with 5 wt% PT-DC after 3 reprocessing cycles.

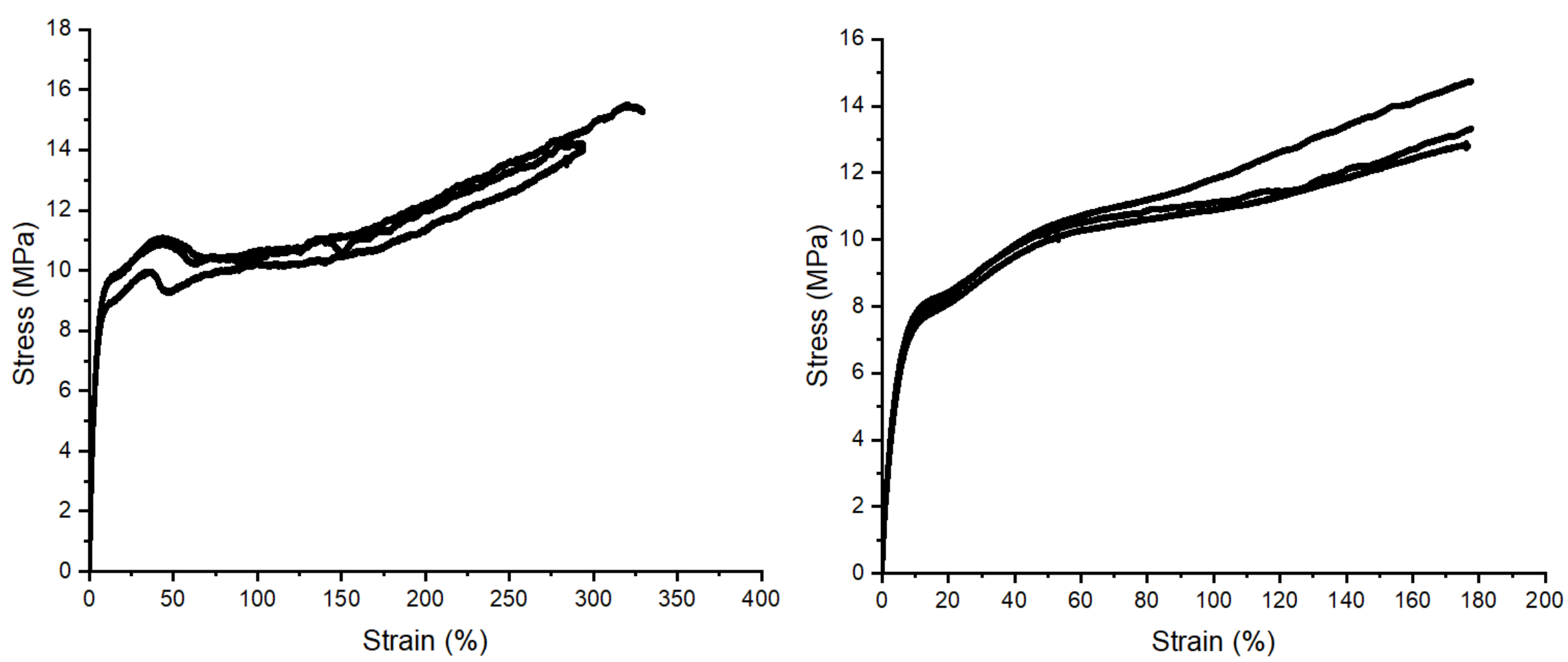

Figure S74. Uniaxial tensile stress-strain curves shown in triplicates: LDPE control (left), LDPE treated with 5 wt% PT-DC (right).

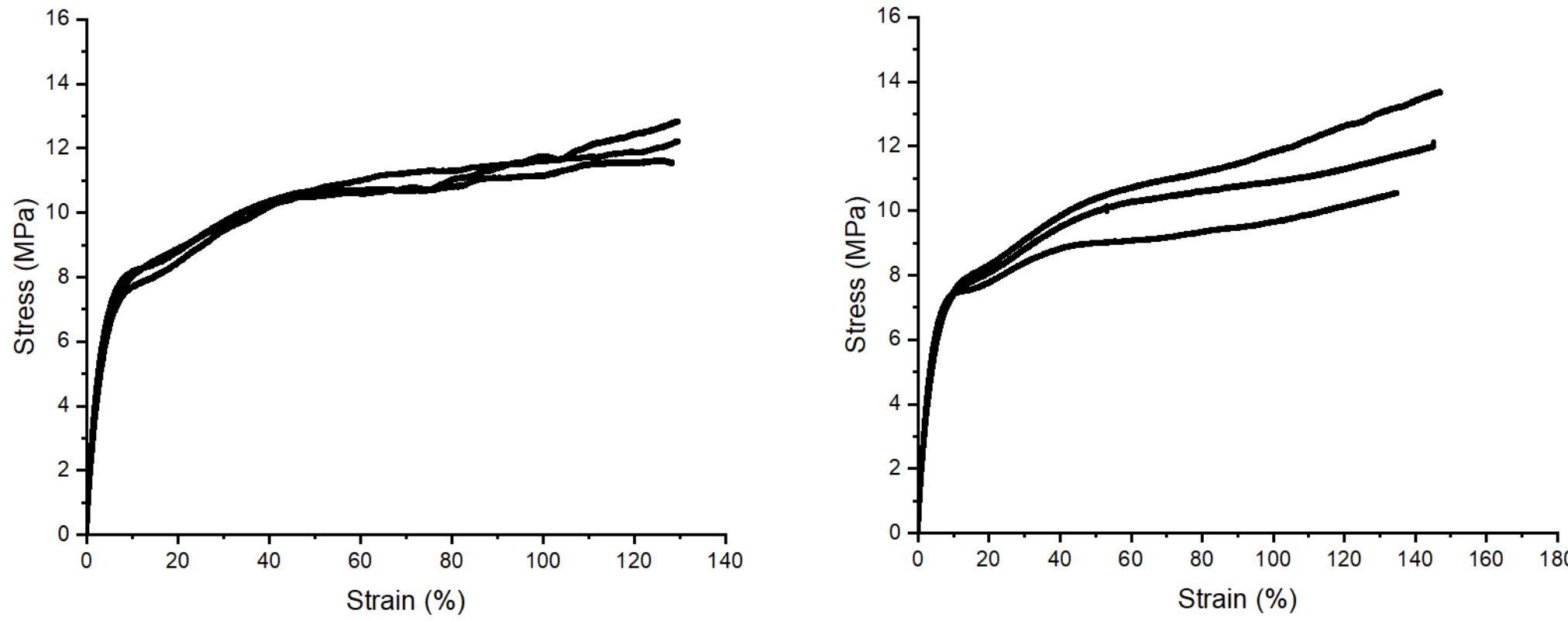

Figure S75. Uniaxial tensile stress-strain curves shown in triplicates: LDPE with 5 wt% PT-DC after 1 reprocessing (left) and after 2 reprocessing cycles (right).

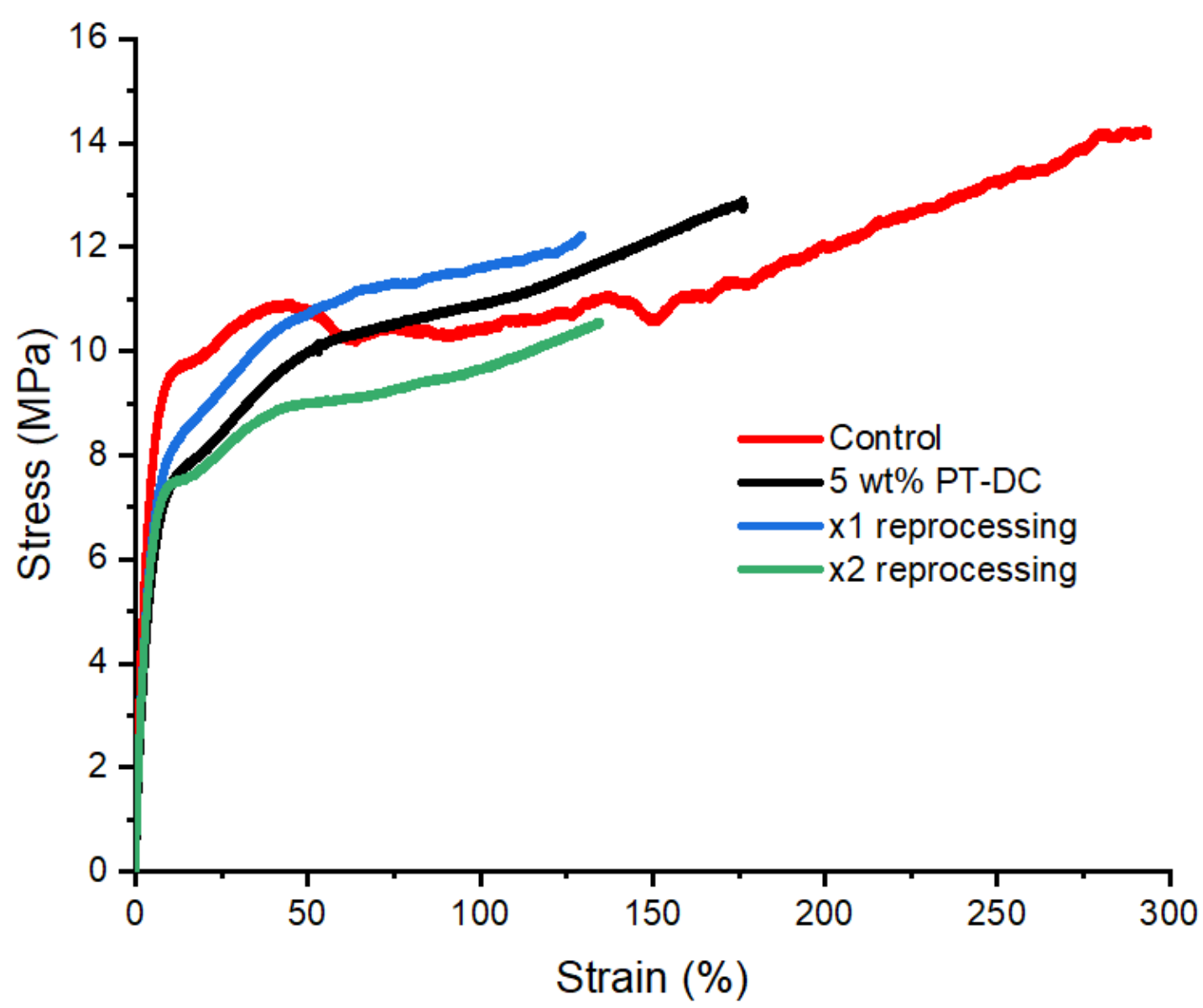

Figure S76. Comparison of uniaxial tensile testing of LDPE control (red), LDPE with 5 wt% PT-DC (black), LDPE after 1 reprocessing cycle (blue), and LDPE after 2 reprocessing cycles (green).

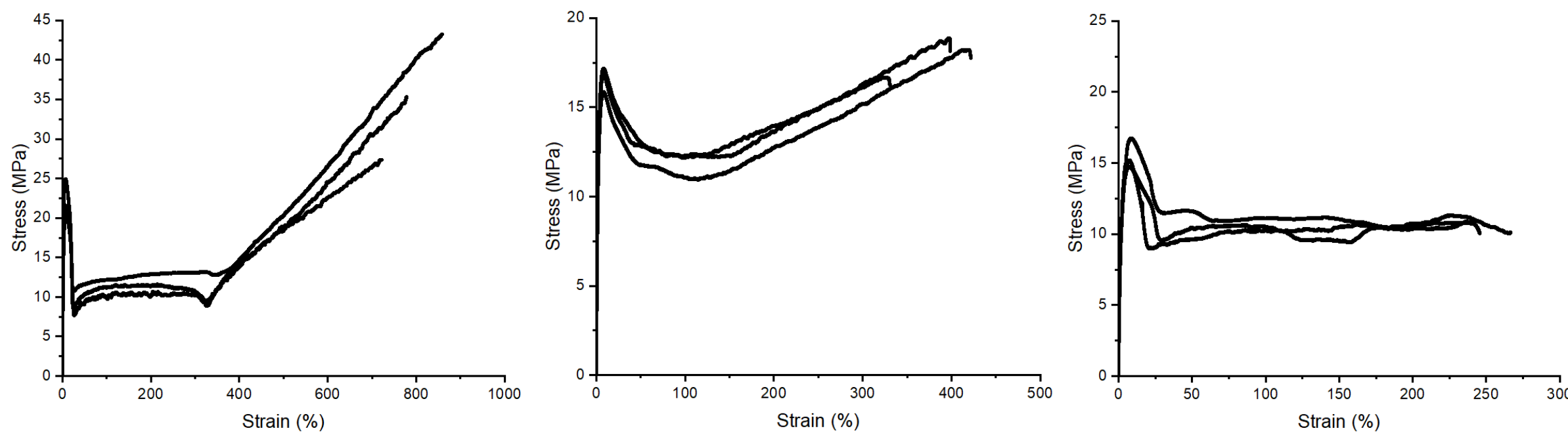

Figure S77. Uniaxial tensile stress-strain curves shown in triplicates: HDPE control (left), HDPE treated with 5 wt% PT-DC (middle), and HDPE with PT-DC reprocessed (right).

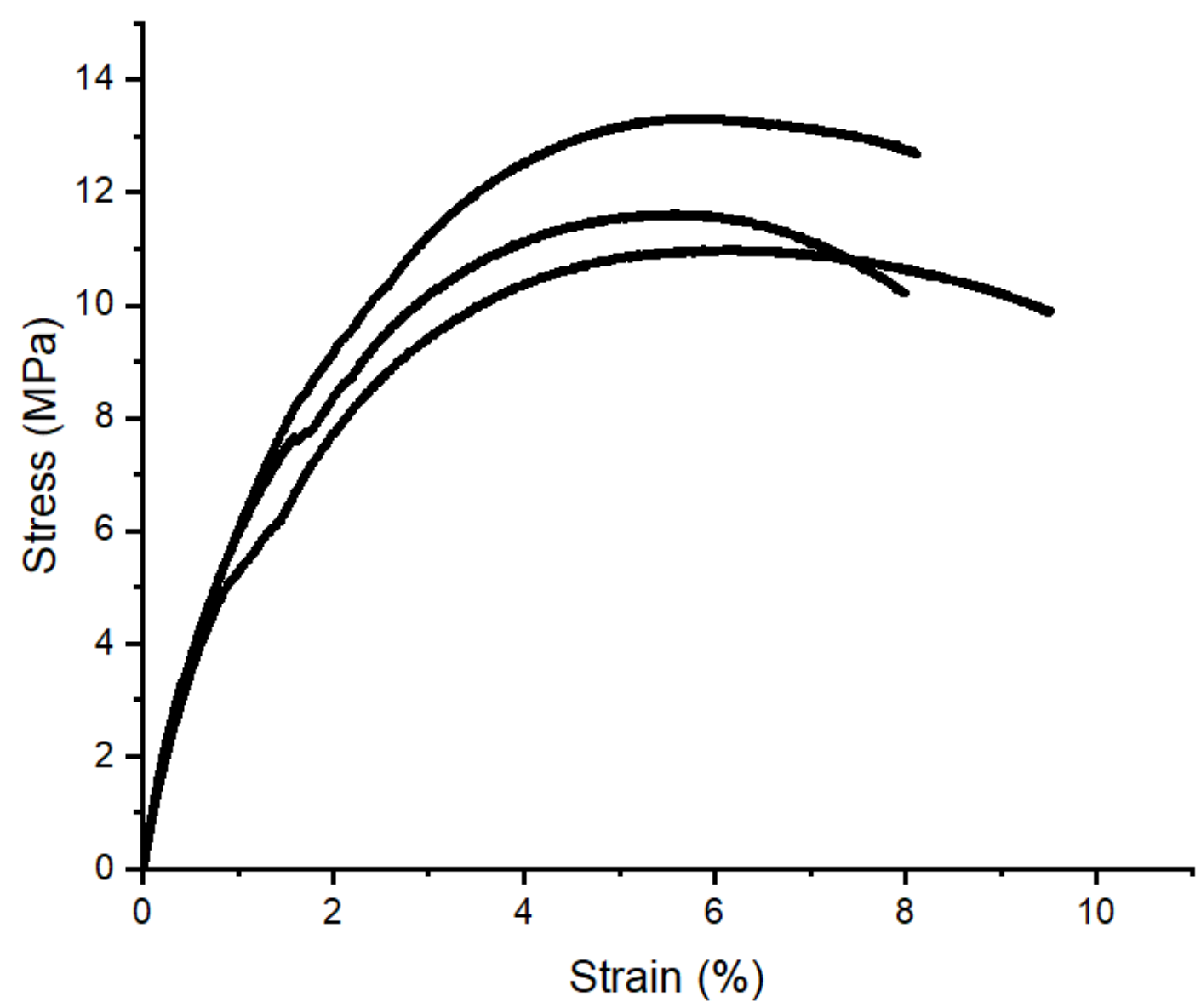

Figure S78. Uniaxial tensile stress-strain curves shown in triplicates for HDPE/Nylon-6 control blend.

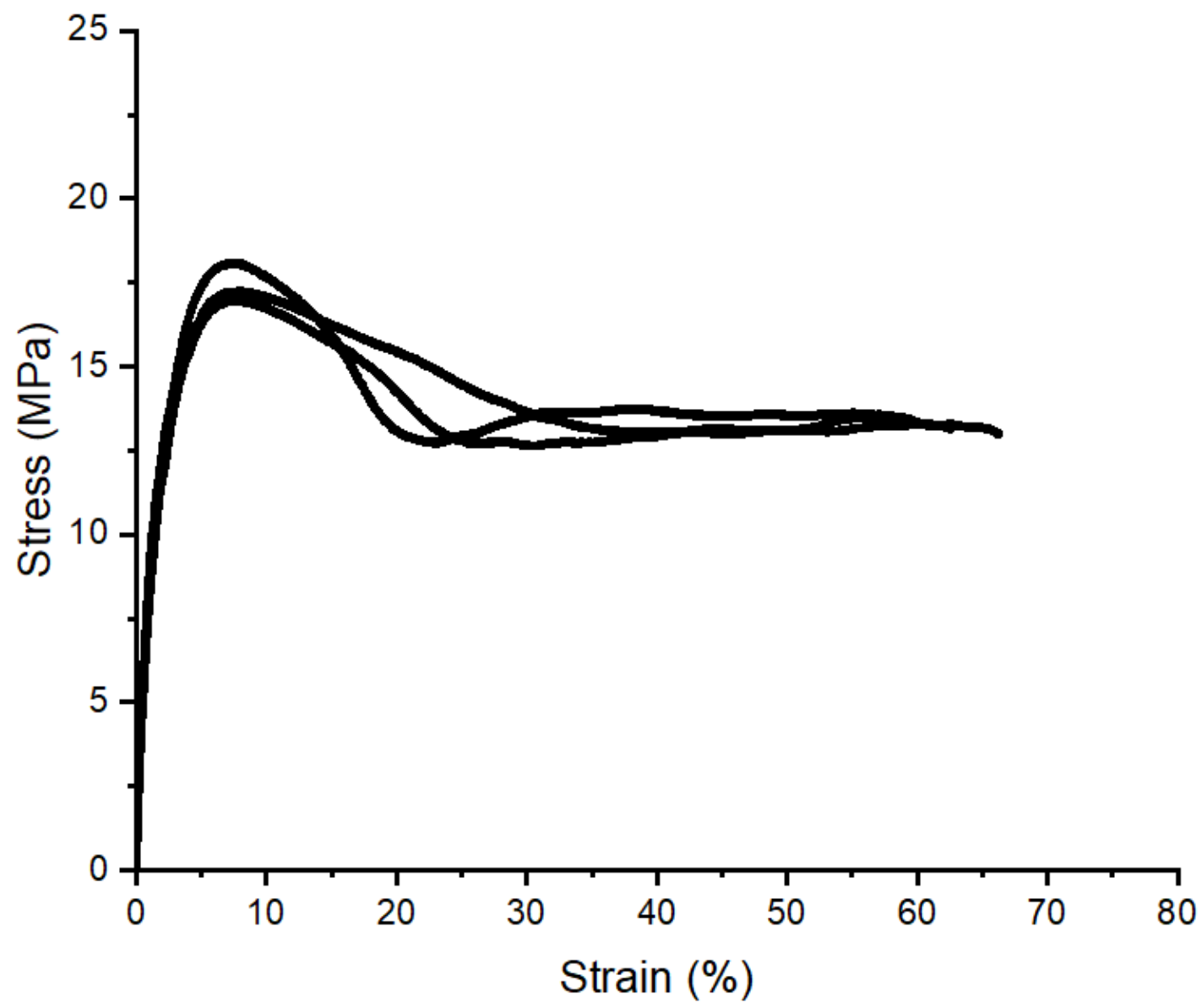

Figure S79. Uniaxial tensile stress-strain curves shown in triplicates for HDPE/Nylon-6 blend compatibilized with 5 wt% PT-DC.

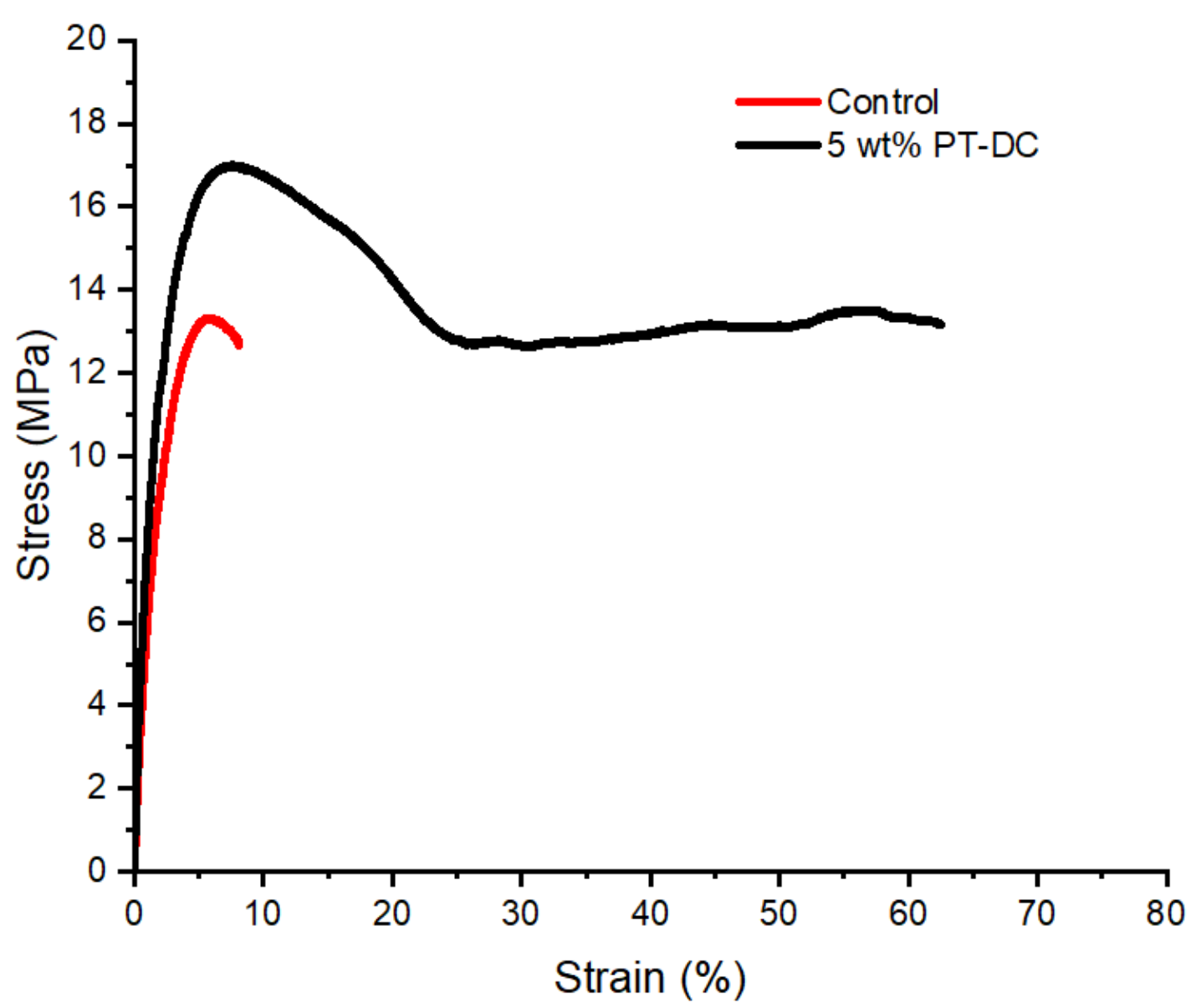

Figure S80. Uniaxial tensile stress-strain curves of HDPE/Nylon-6 control blend (red) and HDPE/Nylon-6 blend compatibilized with 5 wt% PT-DC (black).

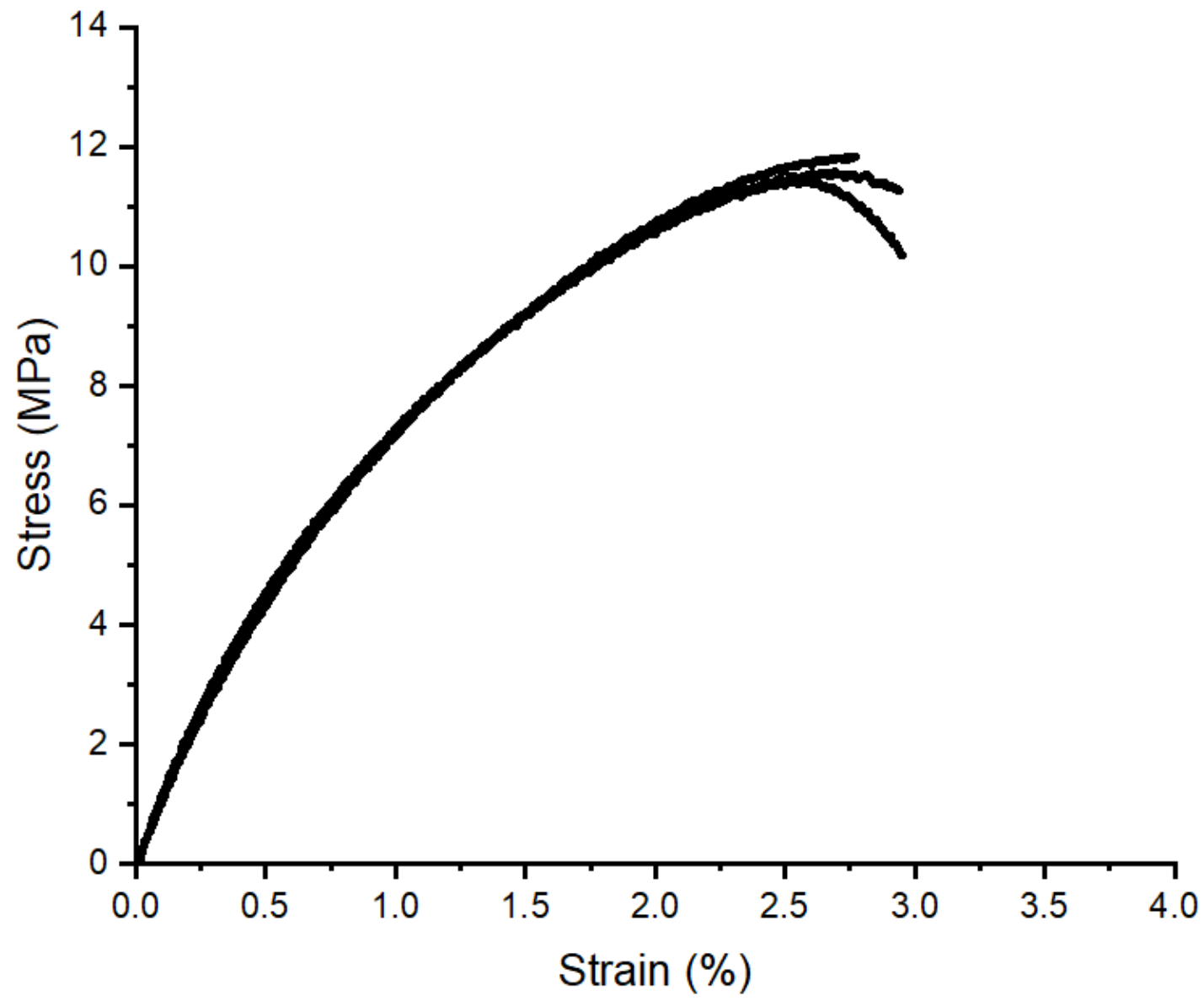

Figure S81. Uniaxial tensile testing of ternary blend control (LDPE: 35%, HDPE: 28.6%, *i*PP: 36.4%) in triplicates.

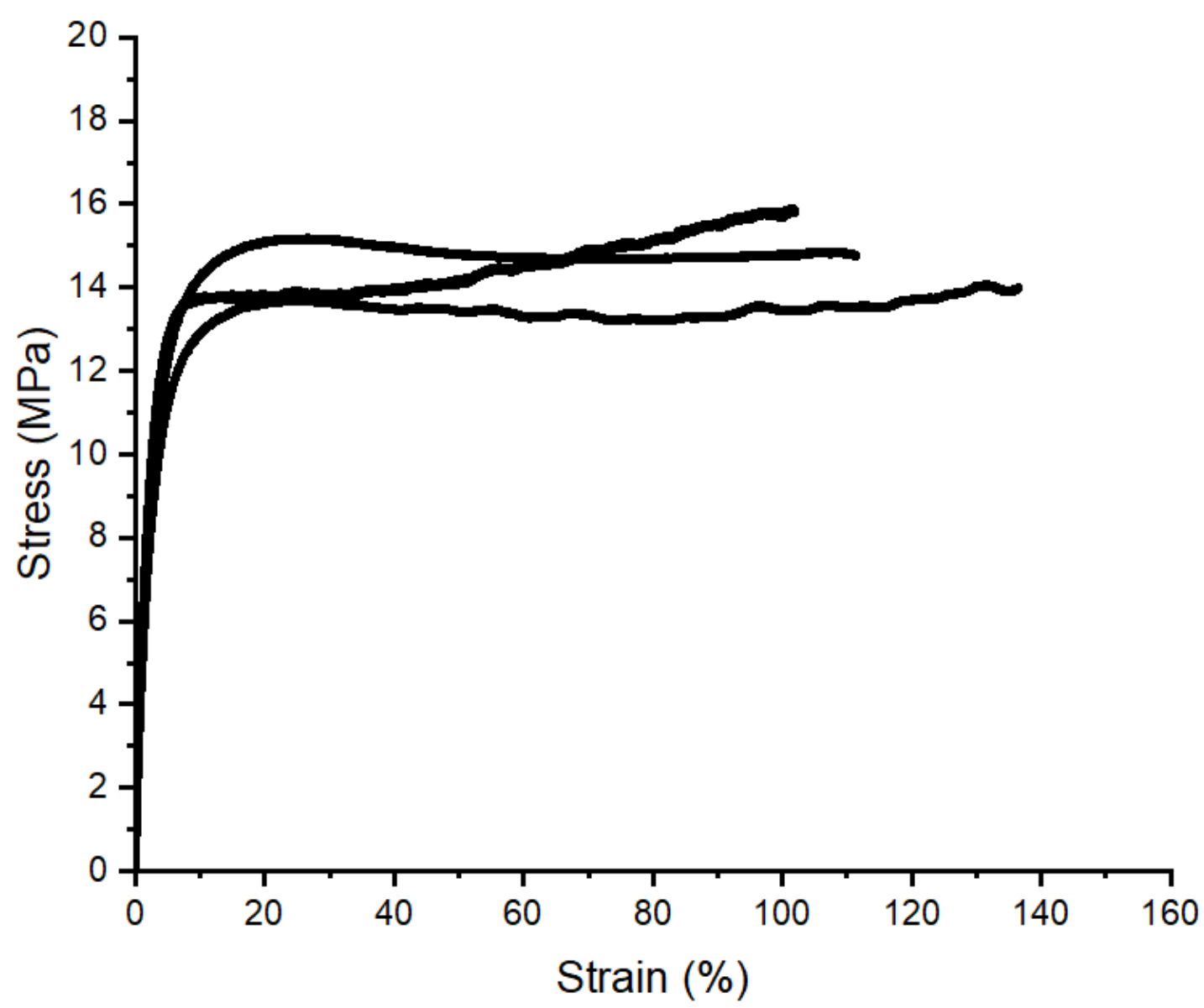

Figure S82. Uniaxial tensile testing of the ternary blend with 5 wt% PT-DC (LDPE: 35%, HDPE: 28.6%, *i*PP: 36.4%) in triplicates.

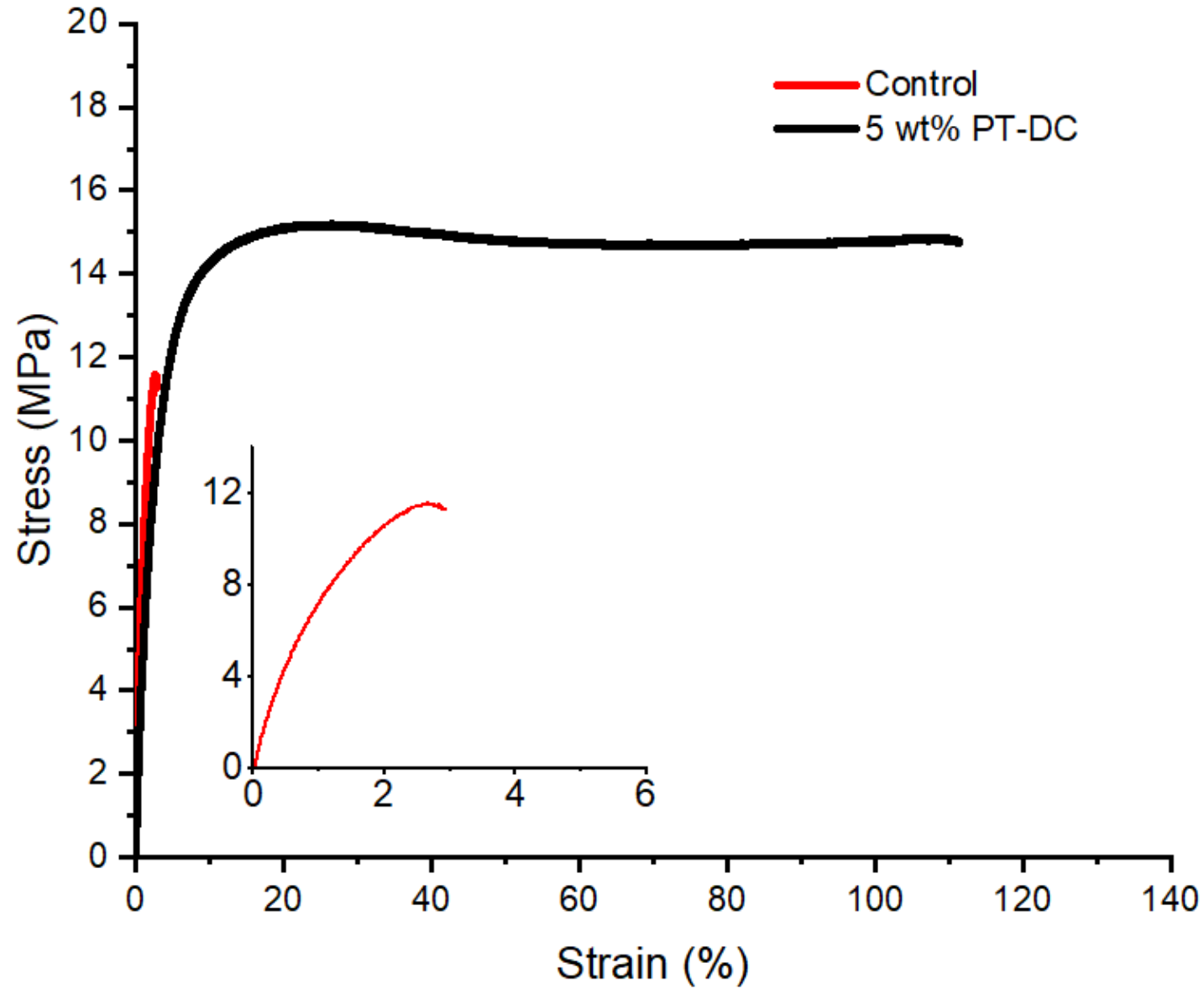

Figure S83. Uniaxial tensile profiles of ternary blend (LDPE: 35%, HDPE: 28.6%, *i*PP: 36.4%): control (red curve) and compatibilized blend with 5 wt% PT-DC (black curve).

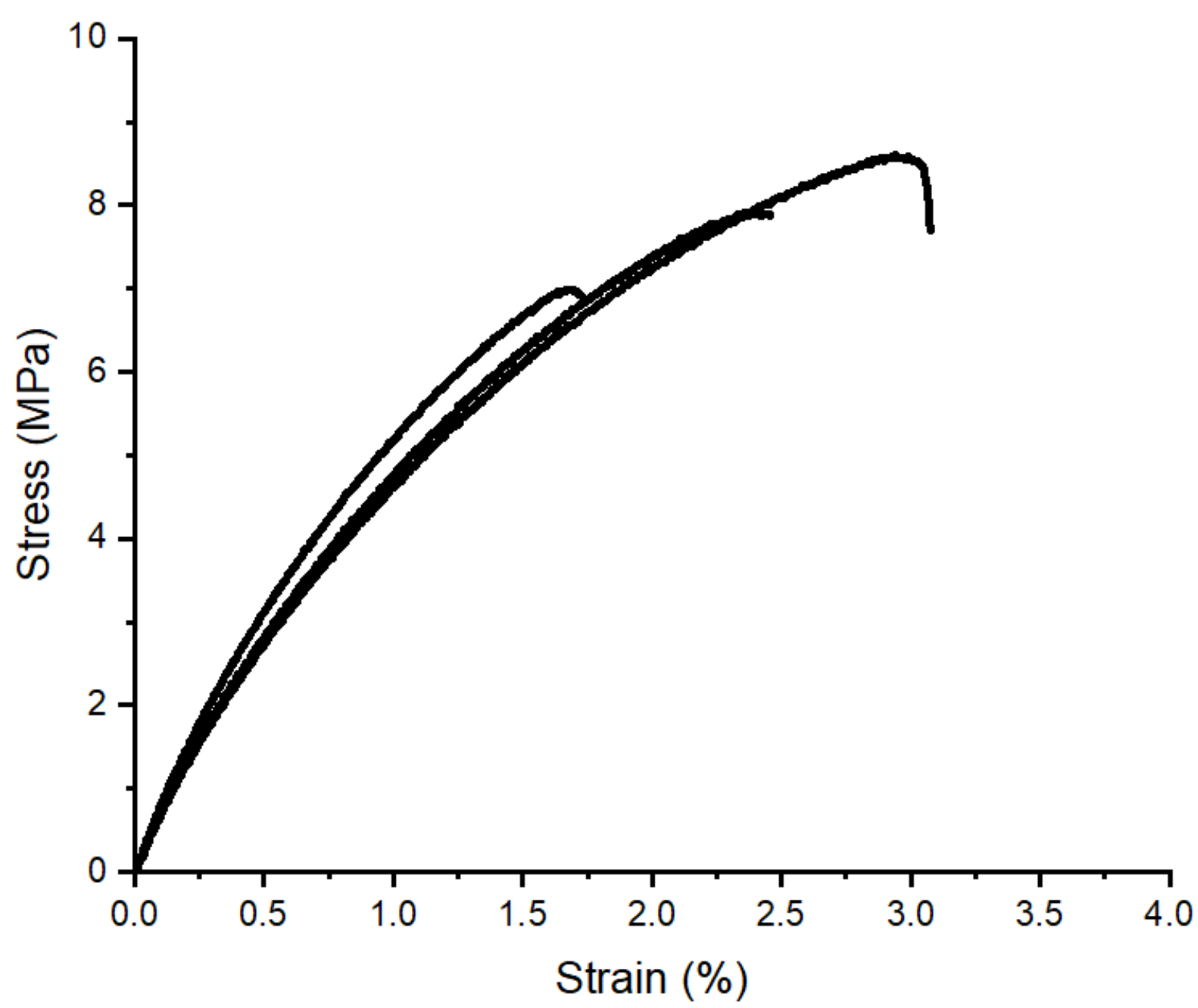

Figure S84. Uniaxial tensile testing of quaternary blend (LDPE: 30%, HDPE: 24.4%, *i*PP: 31.1%, PET: 14.4%) control in triplicates.

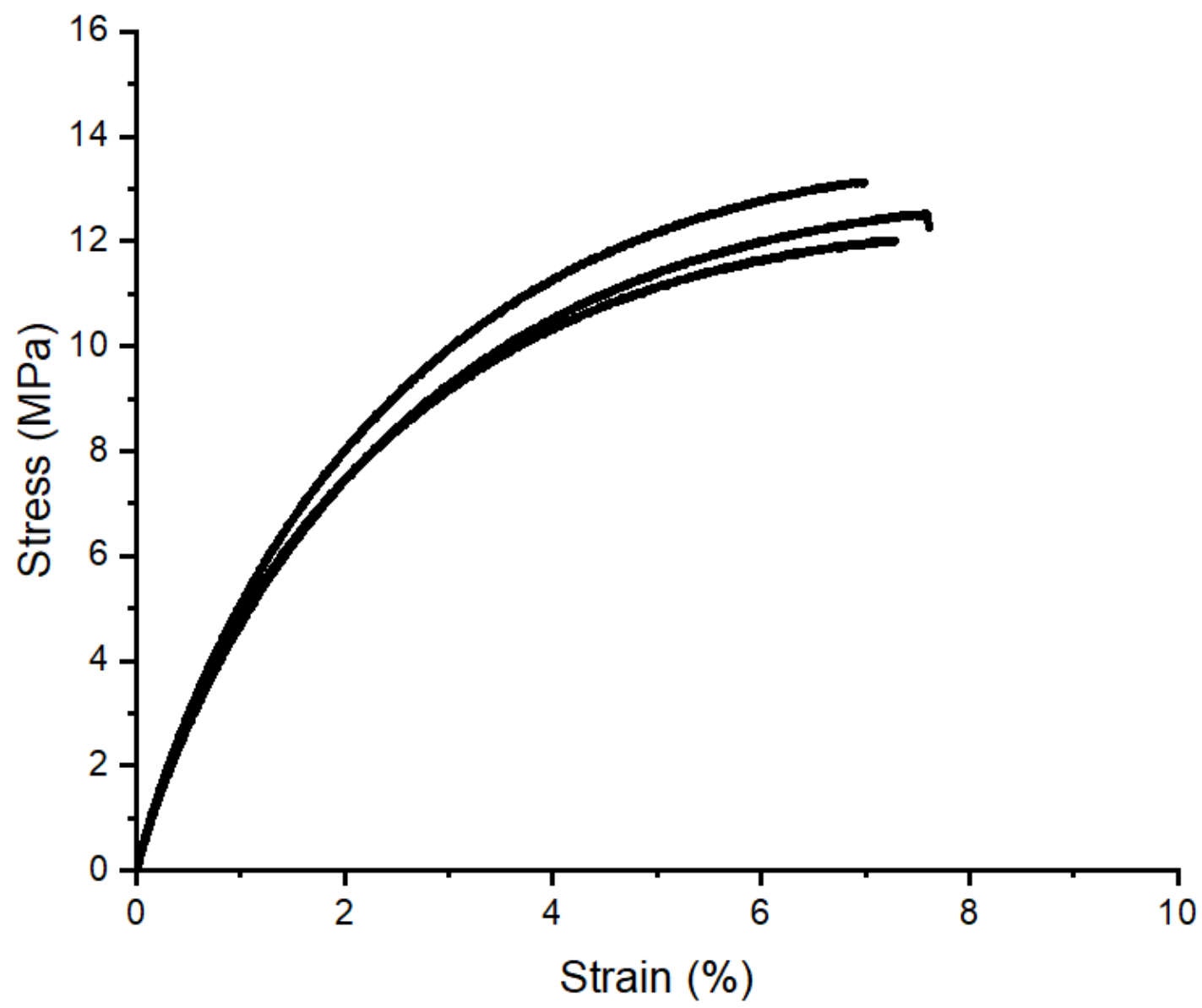

Figure S85. Uniaxial tensile testing of quaternary blend (LDPE: 30%, HDPE: 24.4%, *i*PP: 31.1%, PET: 14.4%) compatibilized with 5 wt% PT-DC in triplicates.

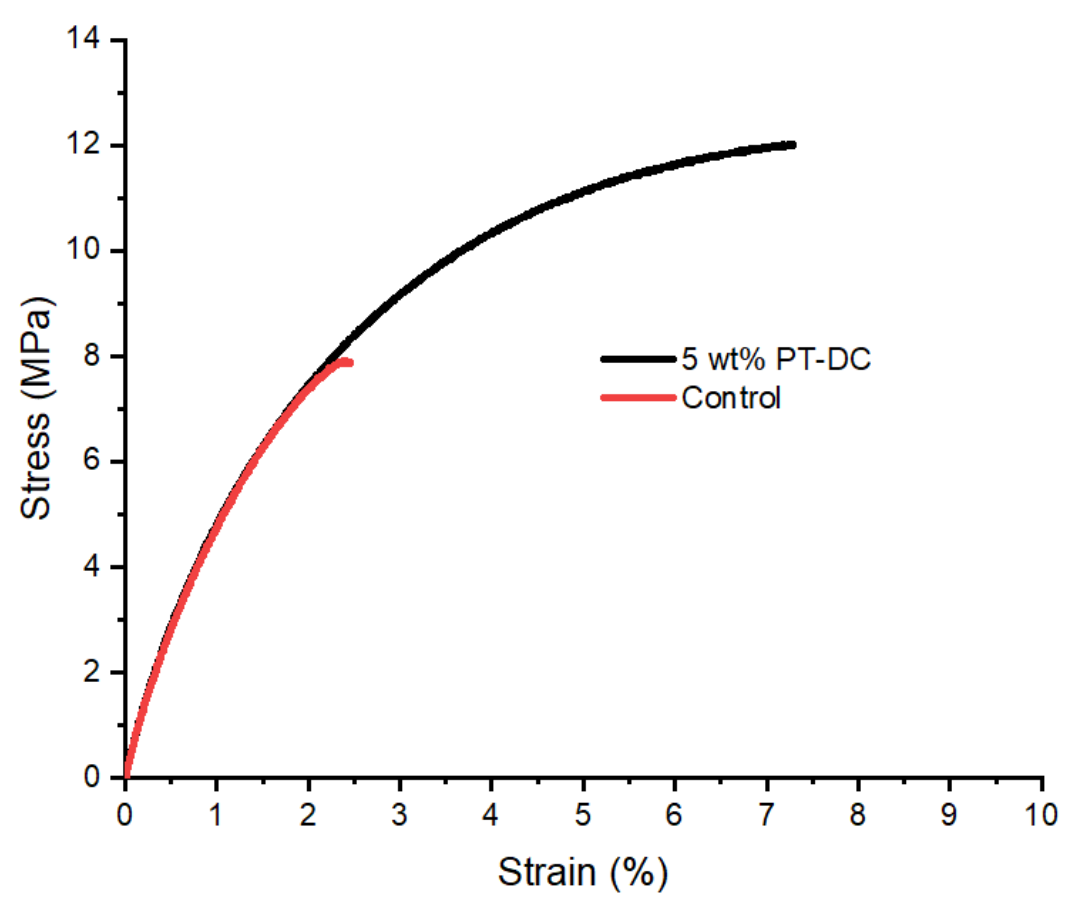

Figure S86. Uniaxial tensile profiles of quaternary blend ((LDPE: 30%, HDPE: 24.4%, *i*PP: 31.1%, PET: 14.4%) control (red curve) and compatibilized blend with 5 wt% PT-DC (black curve).

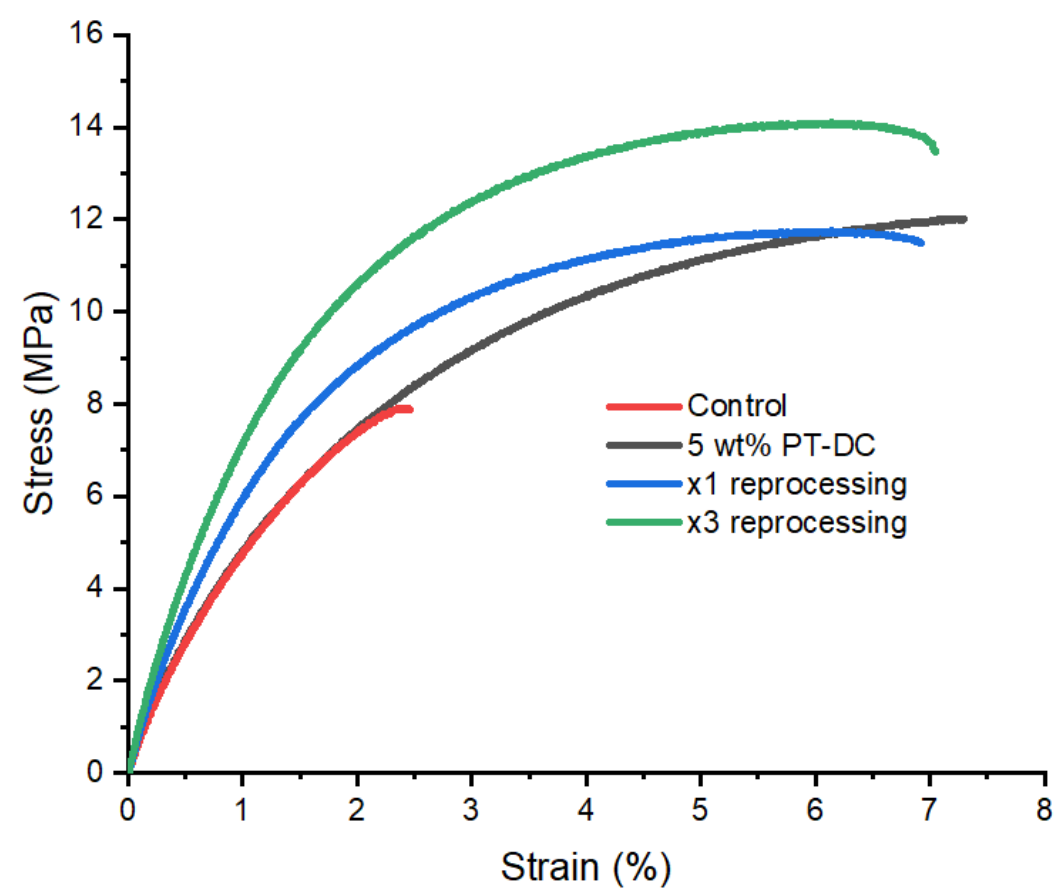

Figure S87. Uniaxial tensile testing of quaternary blending (LDPE: 30%, HDPE: 24.4%, *i*PP: 31.1%, PET: 14.4%) of control (red), with 5 wt% PT-DC (black), after one reprocessing (blue), and after three reprocessing cycles (green).

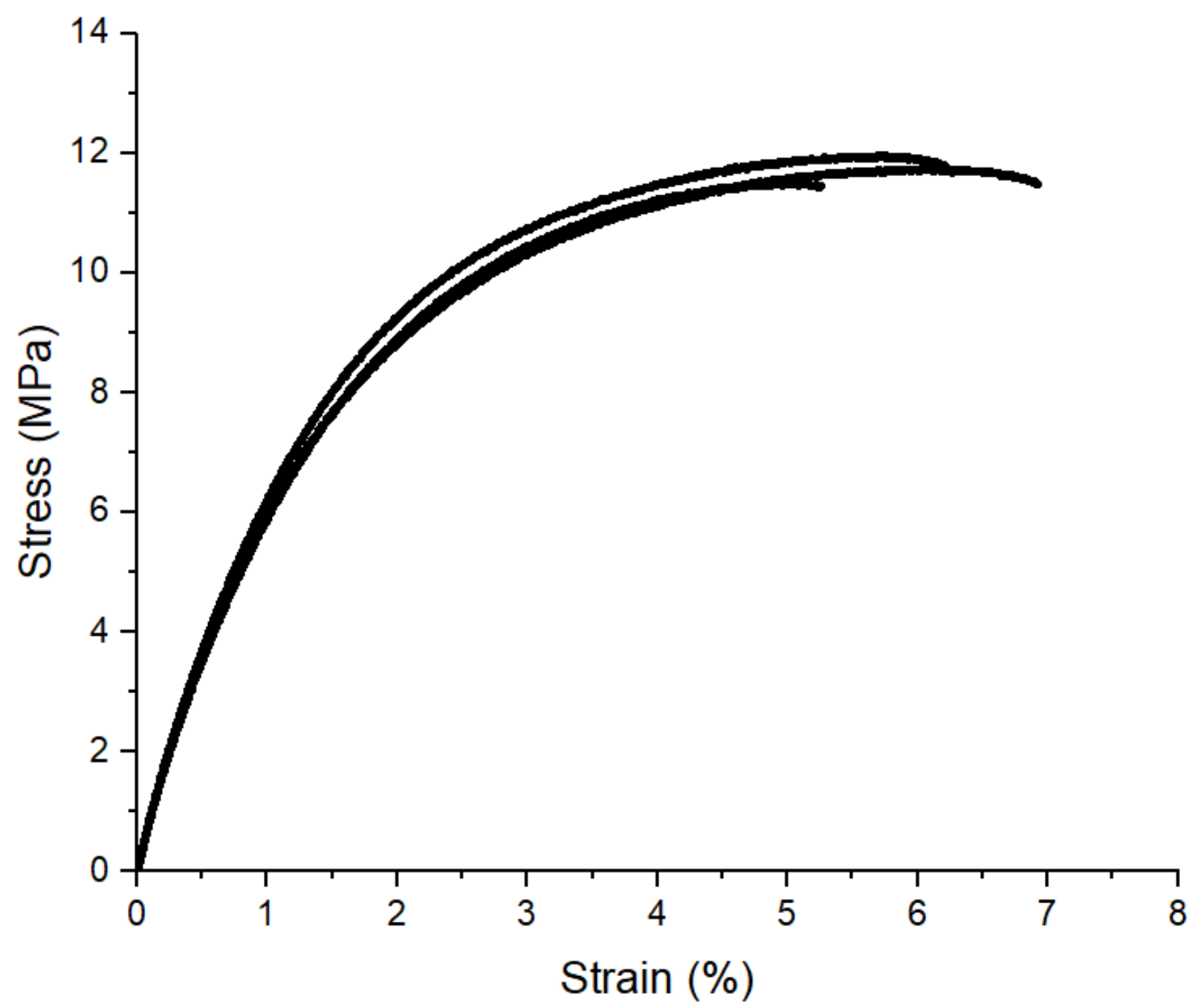

Figure S88. Uniaxial tensile testing of the compatibilized quaternary blending (LDPE: 30%, HDPE: 24.4%, *i*PP: 31.1%, PET: 14.4%) after one reprocessing cycle.

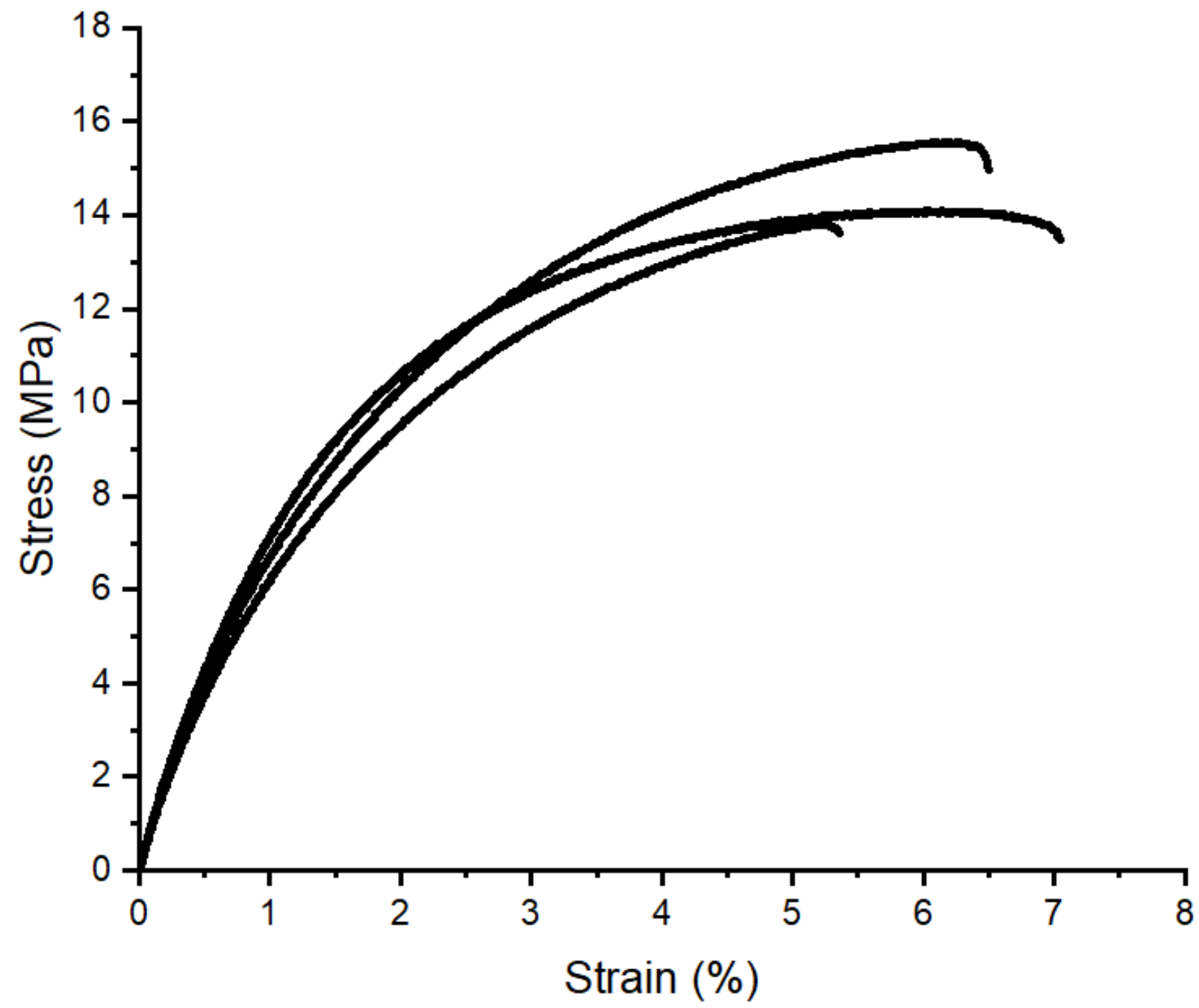

Figure S89. Uniaxial tensile testing of quaternary blending (LDPE: 30%, HDPE: 24.4%, *i*PP: 31.1%, PET: 14.4%) after three reprocessing cycles.

Scanning Electron Microscopy

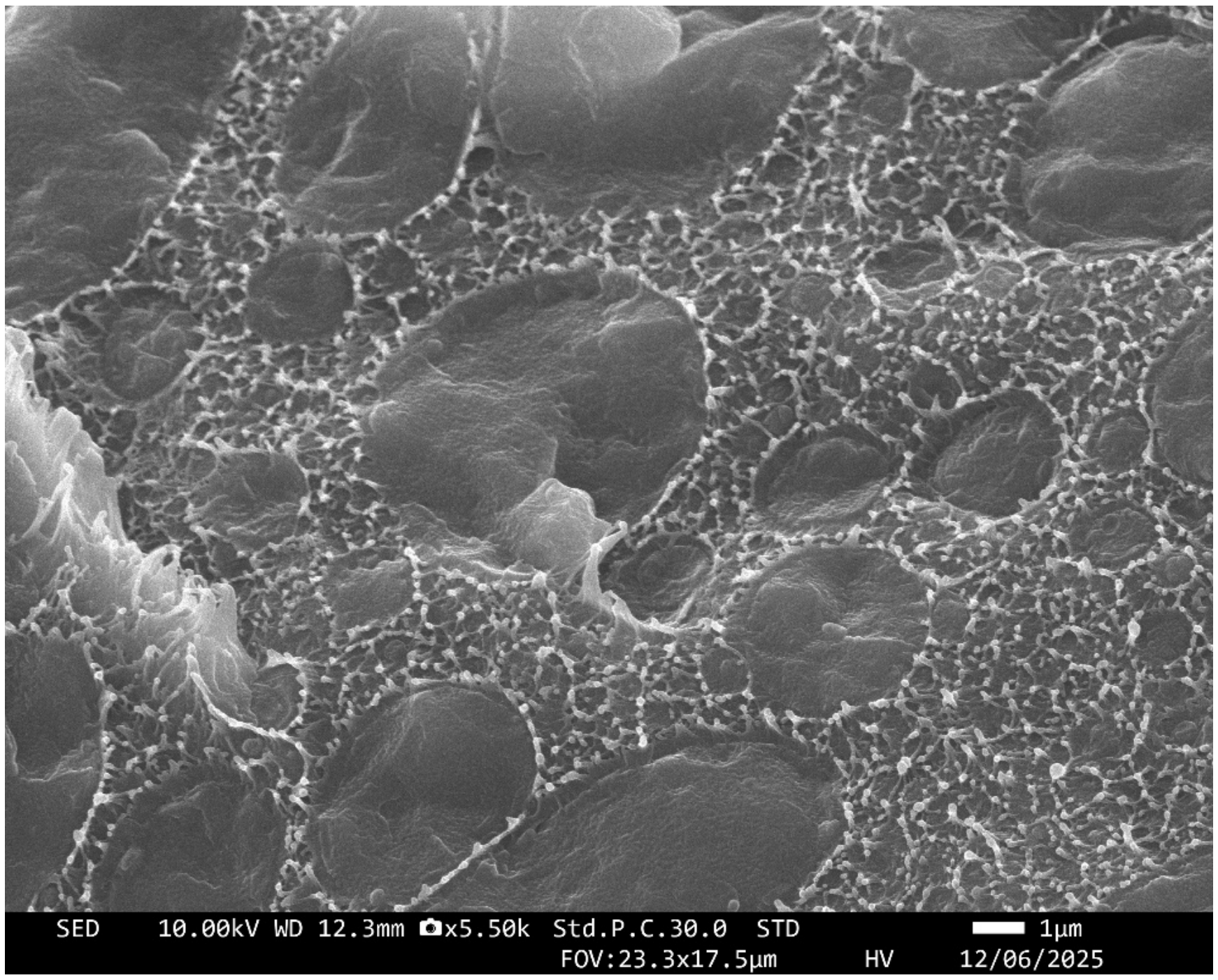

Figure S90. SEM image of ternary plastic waste blend (36.4% *i*PP, 28.6% HDPE, 35% LDPE) control.

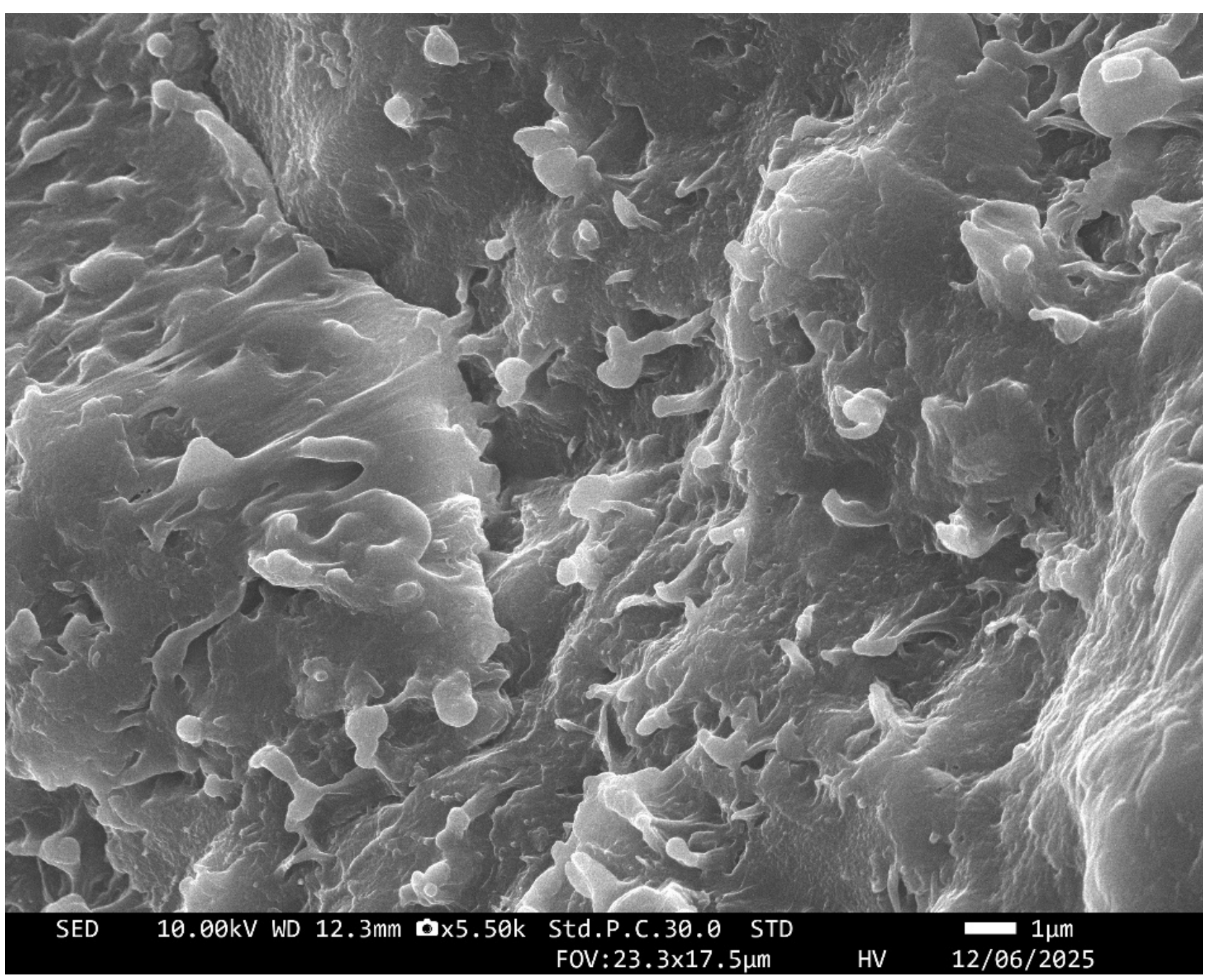

Figure S91. SEM image of ternary plastic waste blend (36.4% *i*PP, 28.6% HDPE, 35% LDPE) with 5 wt% PT-DC.

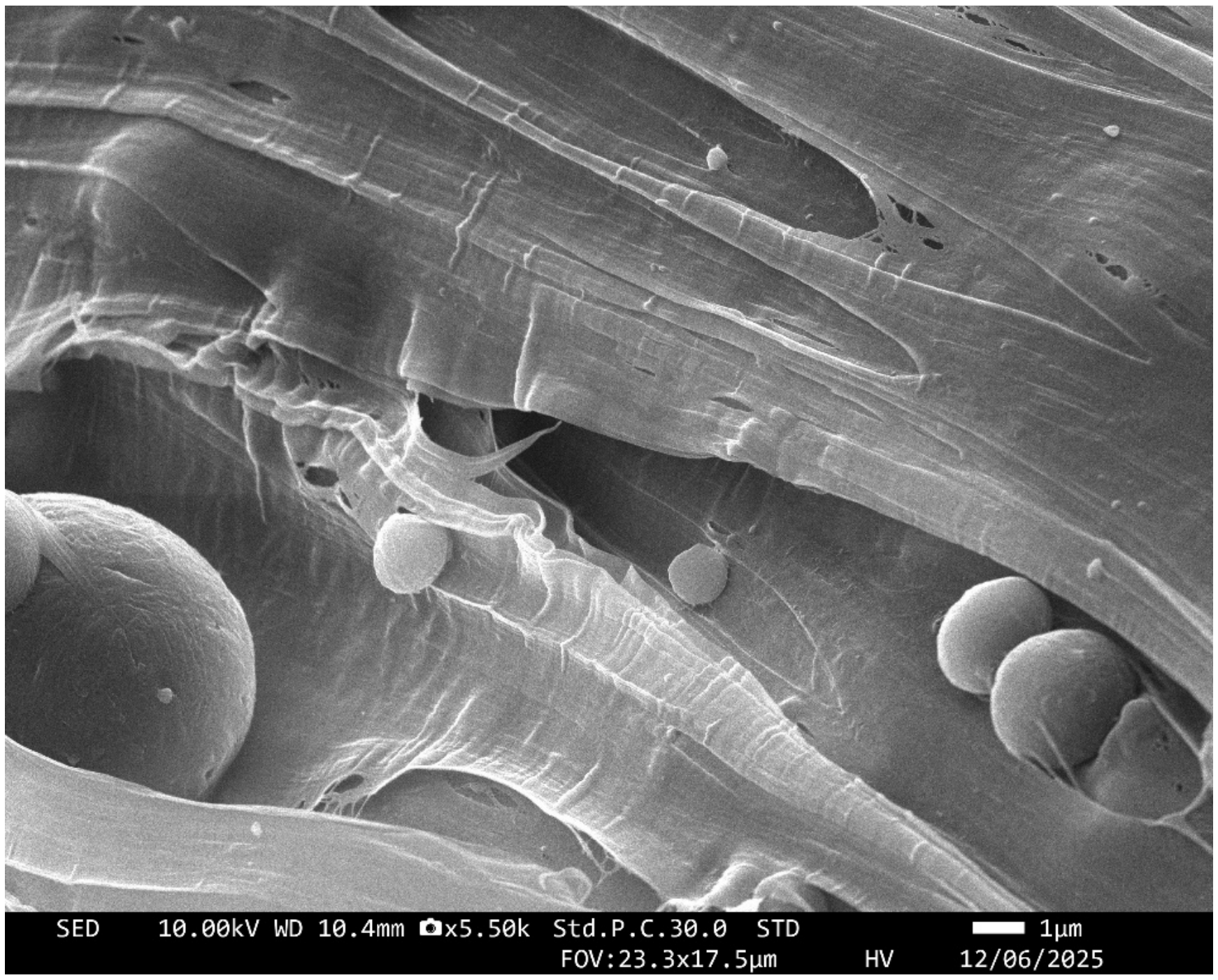

Figure S92. SEM image of quaternary plastic waste blend (14.4% PET, 30% LDPE, 24.4% HDPE, 31.1% *i*PP) control.

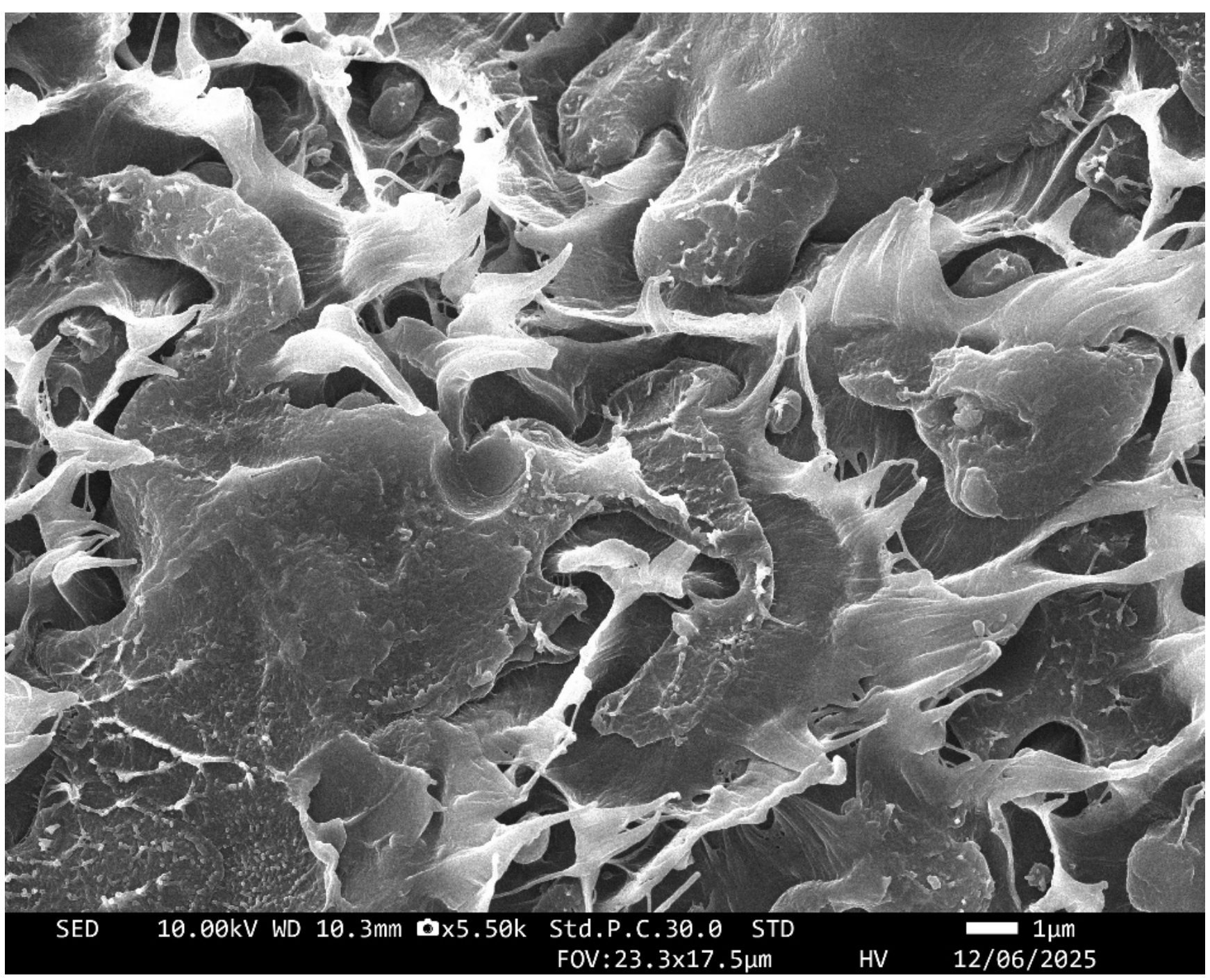

Figure S93 SEM image of quaternary plastic waste blend (14.4% PET, 30% LDPE, 24.4% HDPE, 31.1% *i*PP) with 5 wt% PT-DC.

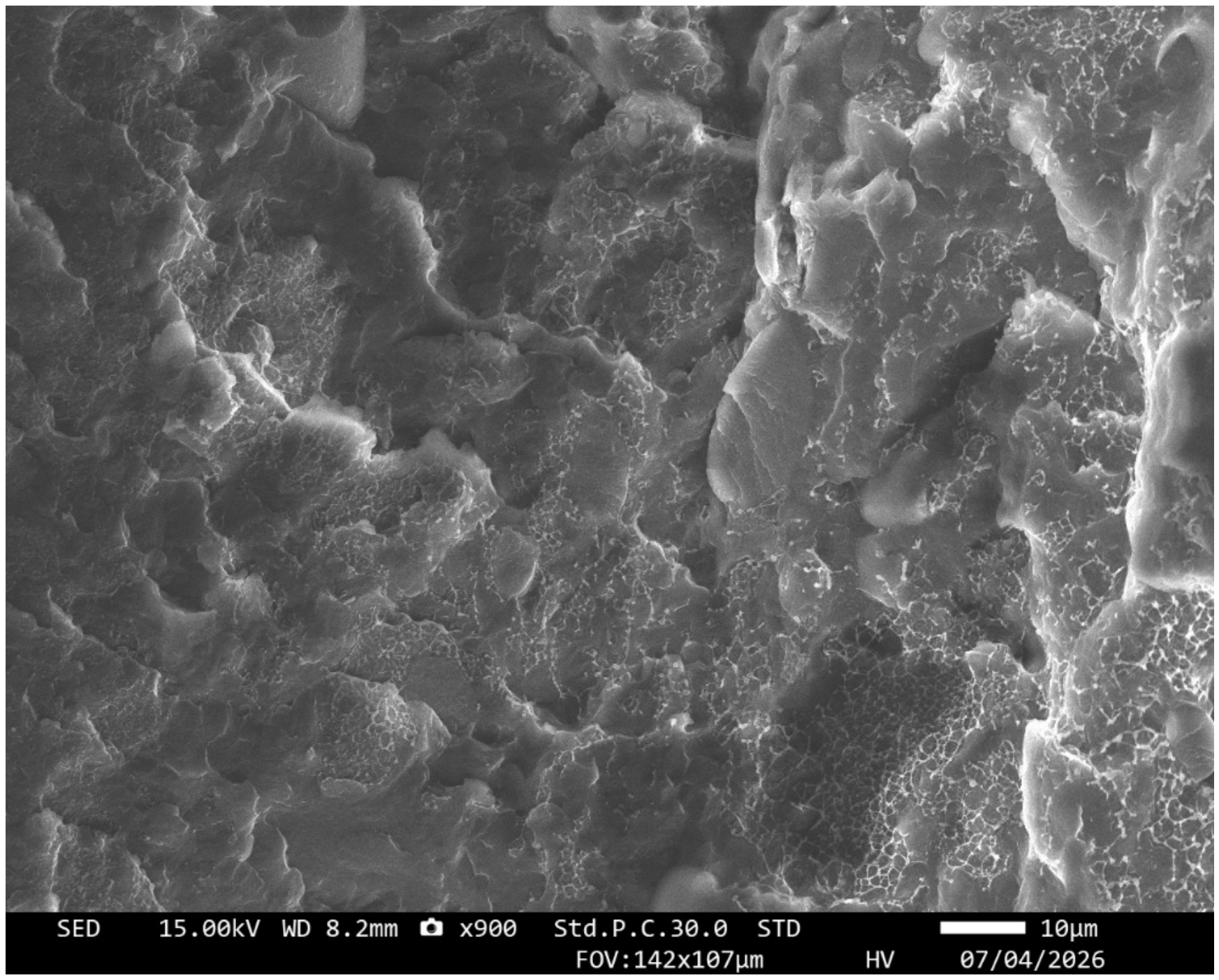

Figure S94. SEM image of quaternary plastic waste blend (14.4% PET, 30% LDPE, 24.4% HDPE, 31.1% *i*PP) with 5 wt% PT-DC after 3 reprocessing cycles.

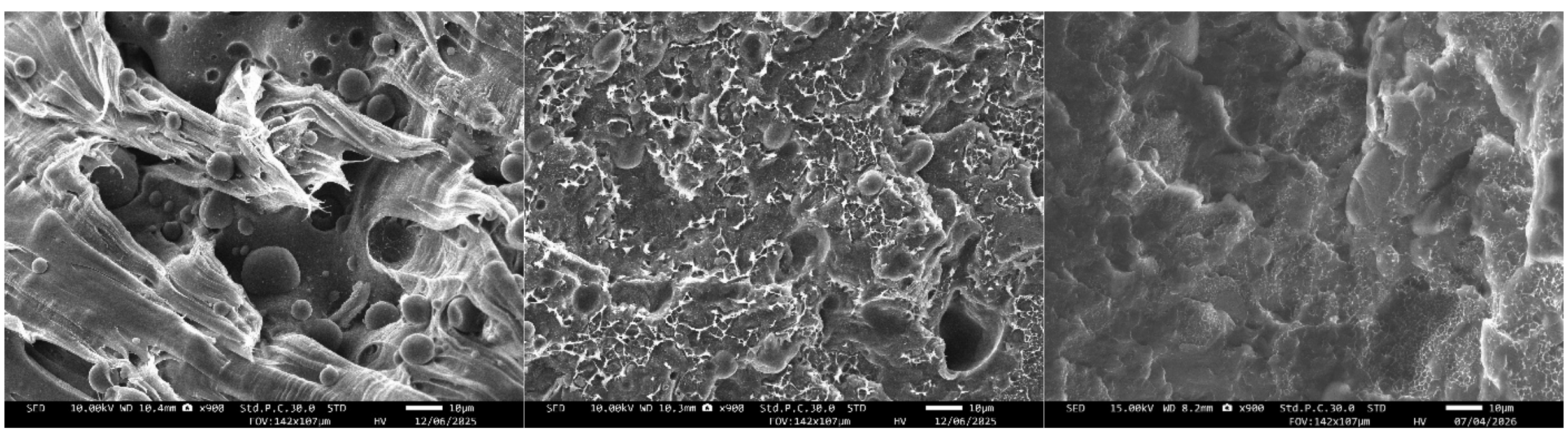

Figure S95. SEM image of quaternary plastic waste blend (14.4% PET, 30% LDPE, 24.4% HDPE, 31.1% *i*PP) control (left), with 5 wt% PT-DC (middle), and after 3 reprocessing cycles (right).

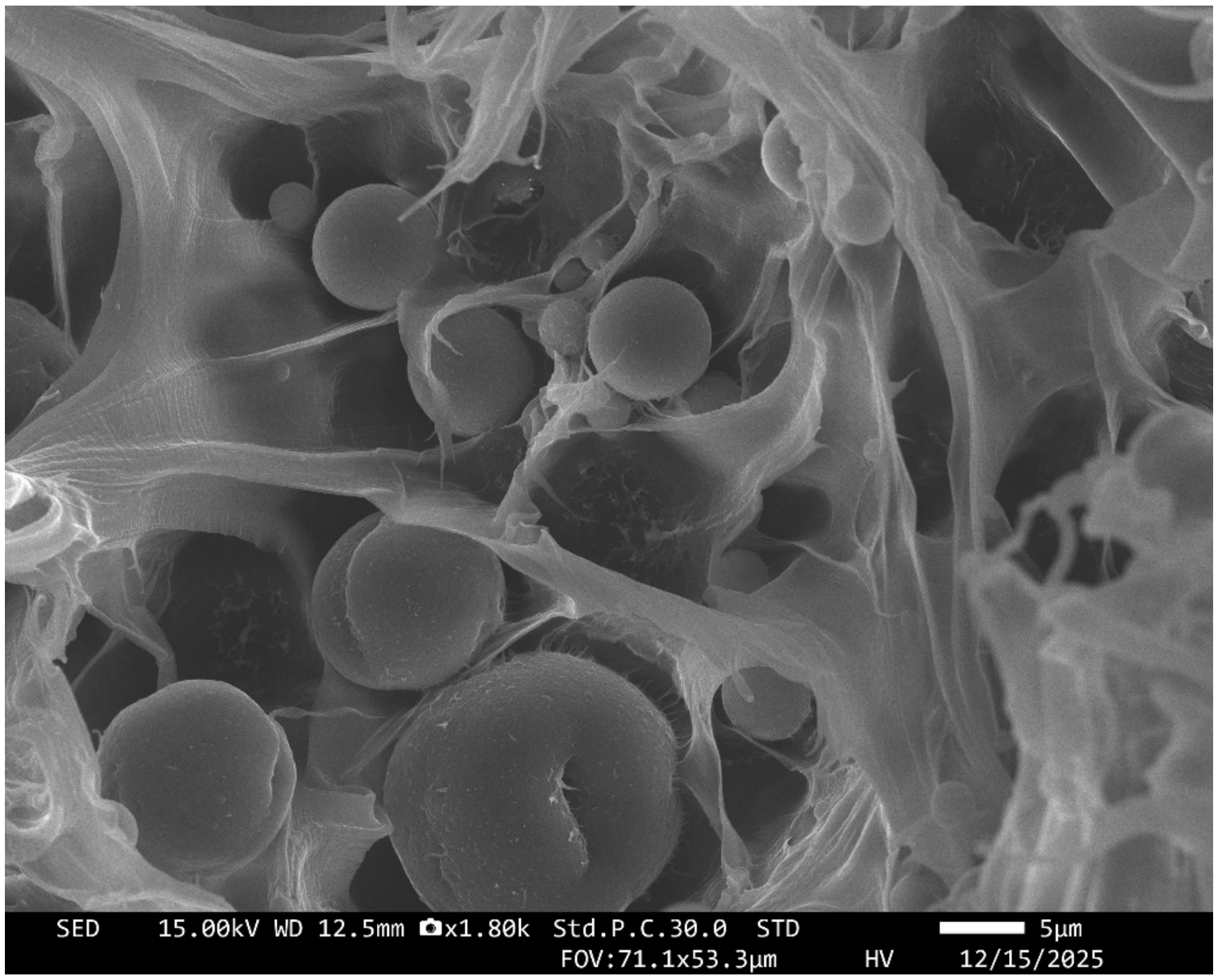

Figure S96. SEM image of LDPE-PET (80:20) waste plastic blend control.

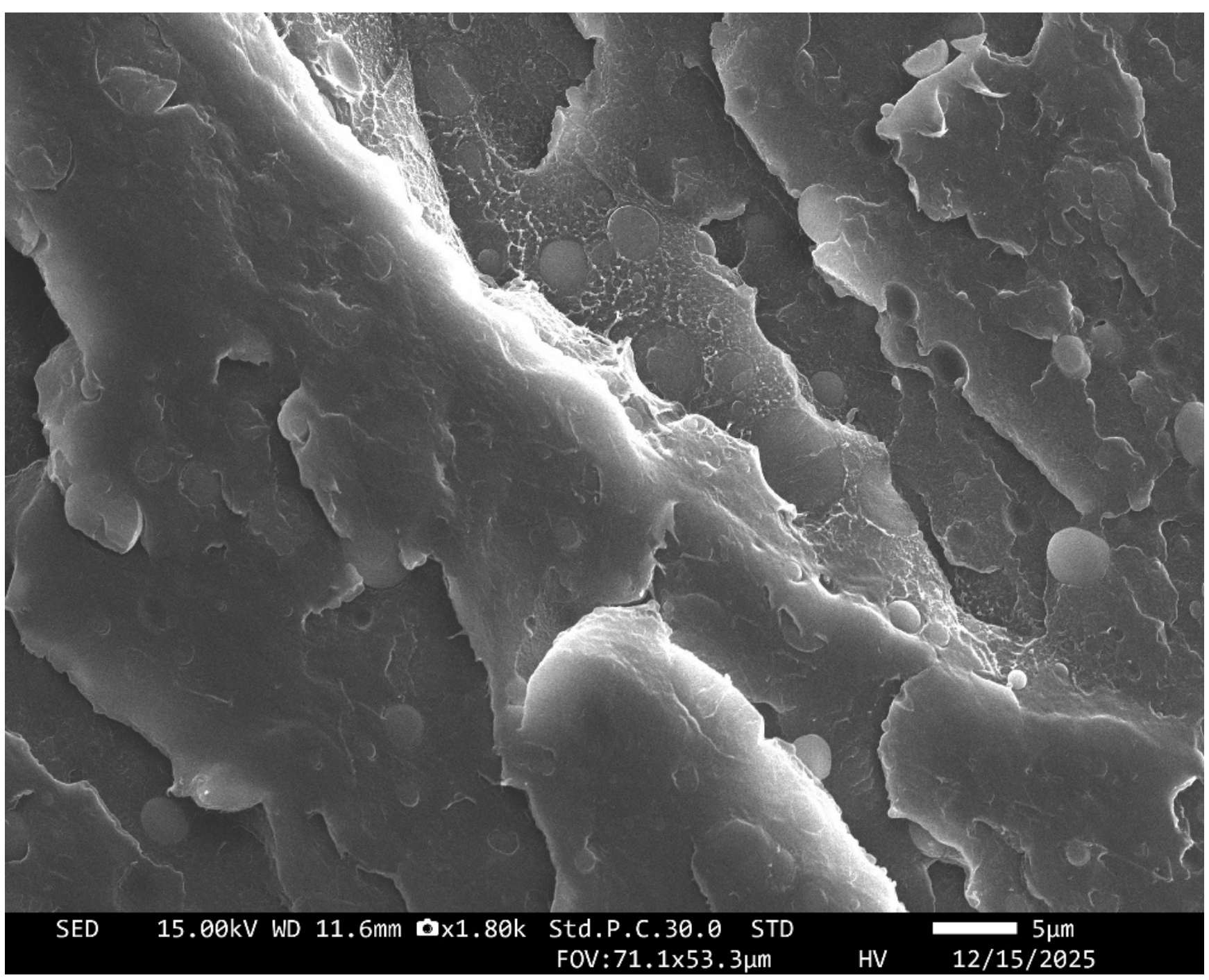

Figure S97. SEM image of LDPE-PET (80:20) waste plastic blend with 5 wt% of PT-DC.

Stress Relaxation

Samples were equilibrated at the test temperature for 5 min, strained to 3%, and monitored for 10,000 s under constant strain.

Arrhenius treatments were made for HDPE following a stretched exponential (Kohlrausch-Williams Watts) function with a beta of 0.1. Mean relaxation time (τ*) was determined for each curve.

(Equation S1) $$t^* = \frac{t}{\beta} \Gamma \frac{1}{\beta}$$

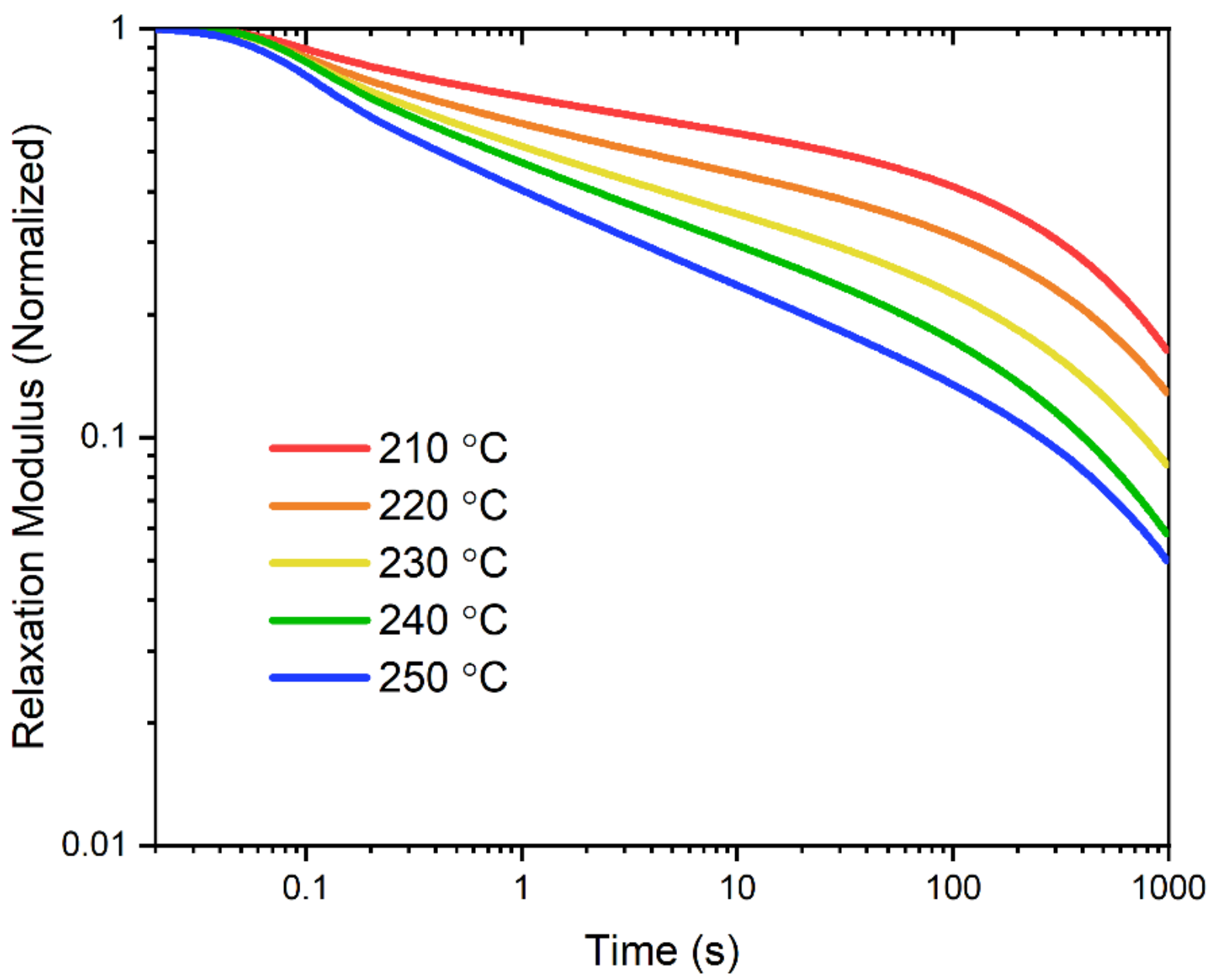

Figure S98. Stress Relaxation of HDPE with 5 wt% PT-DC.

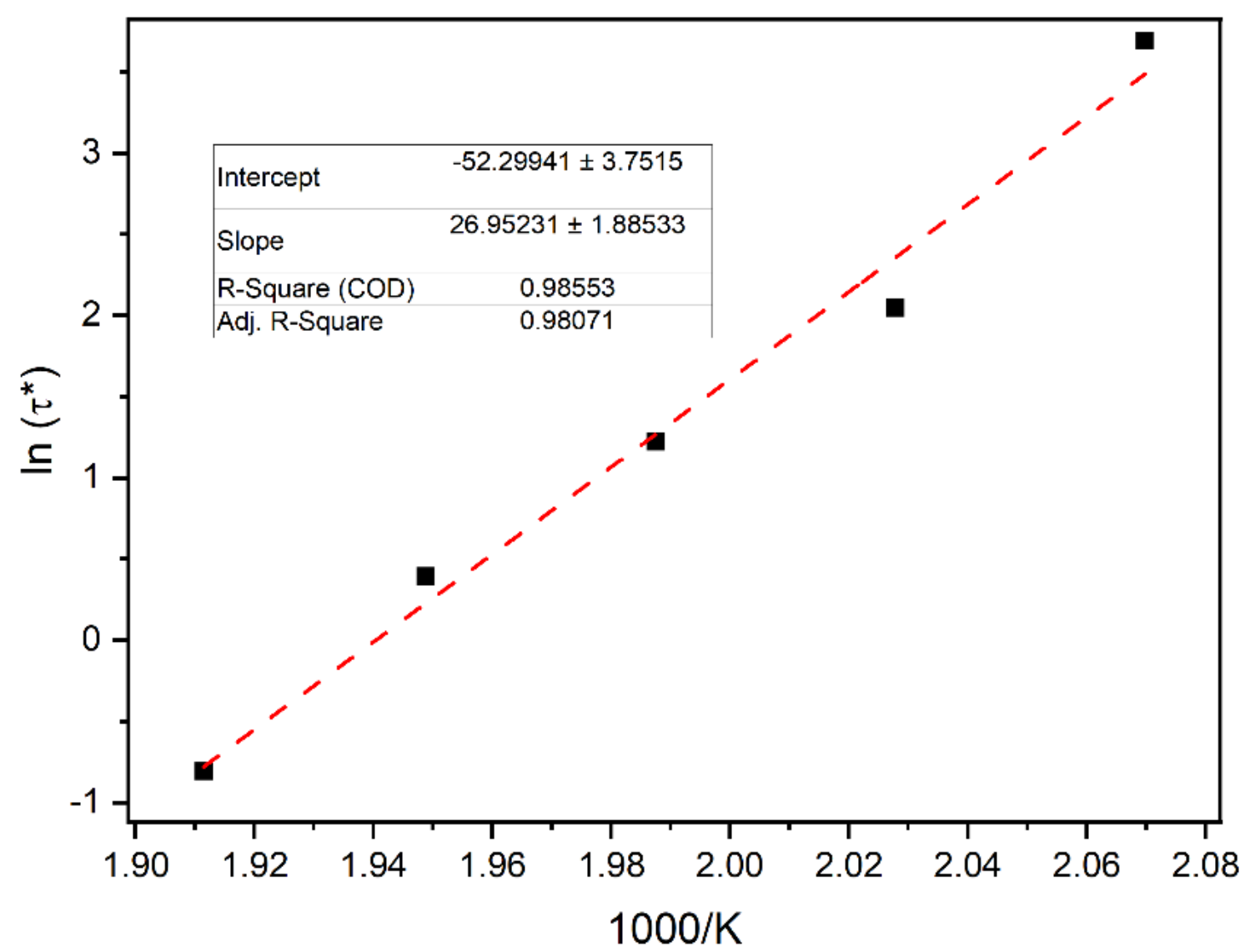

Figure S99. ln (τ*) vs 1000/K plot of HDPE with 5 wt% PT-DC.

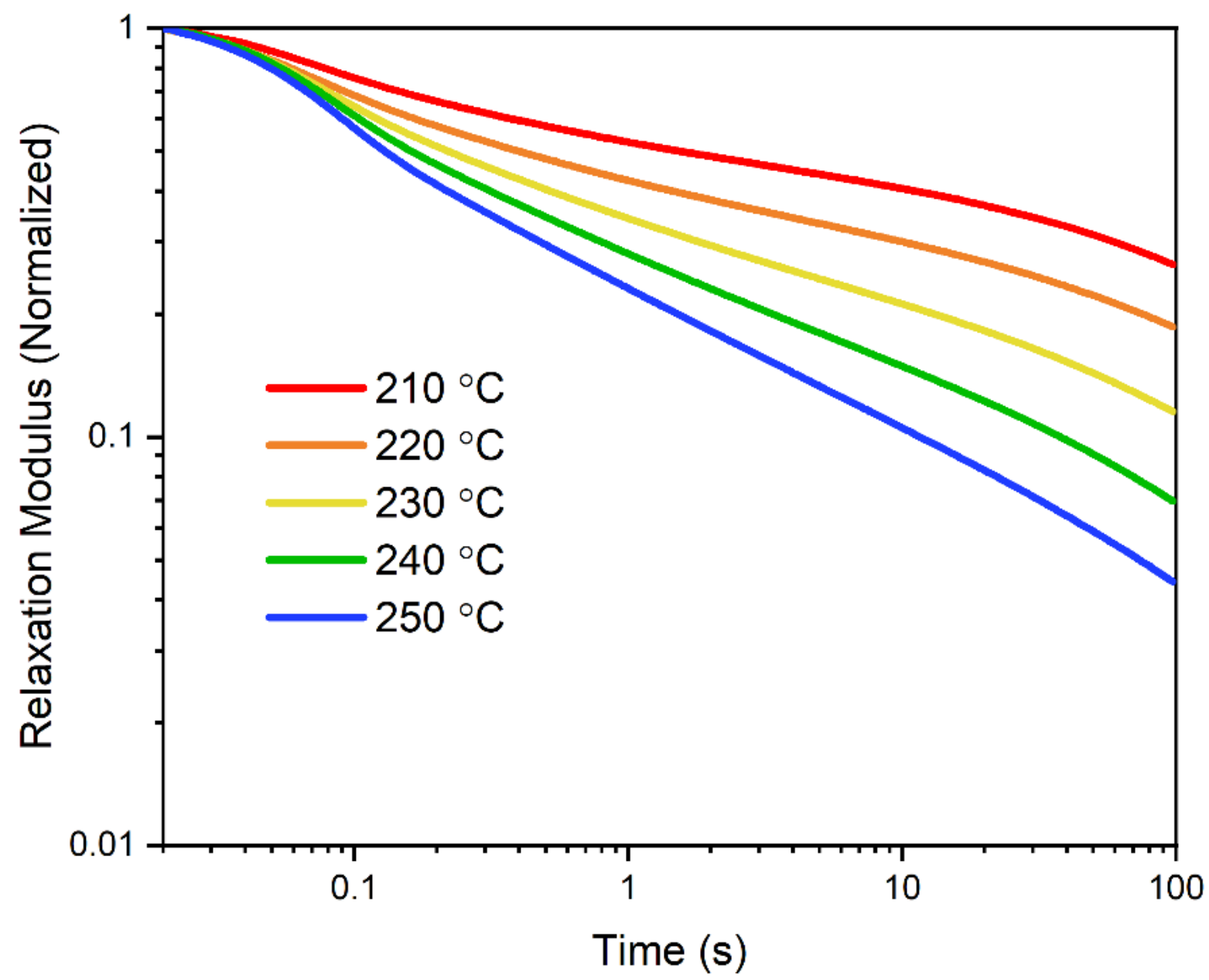

Figure S100. Stress relaxation of HDPE with 5 wt% diazomalonate UDC.

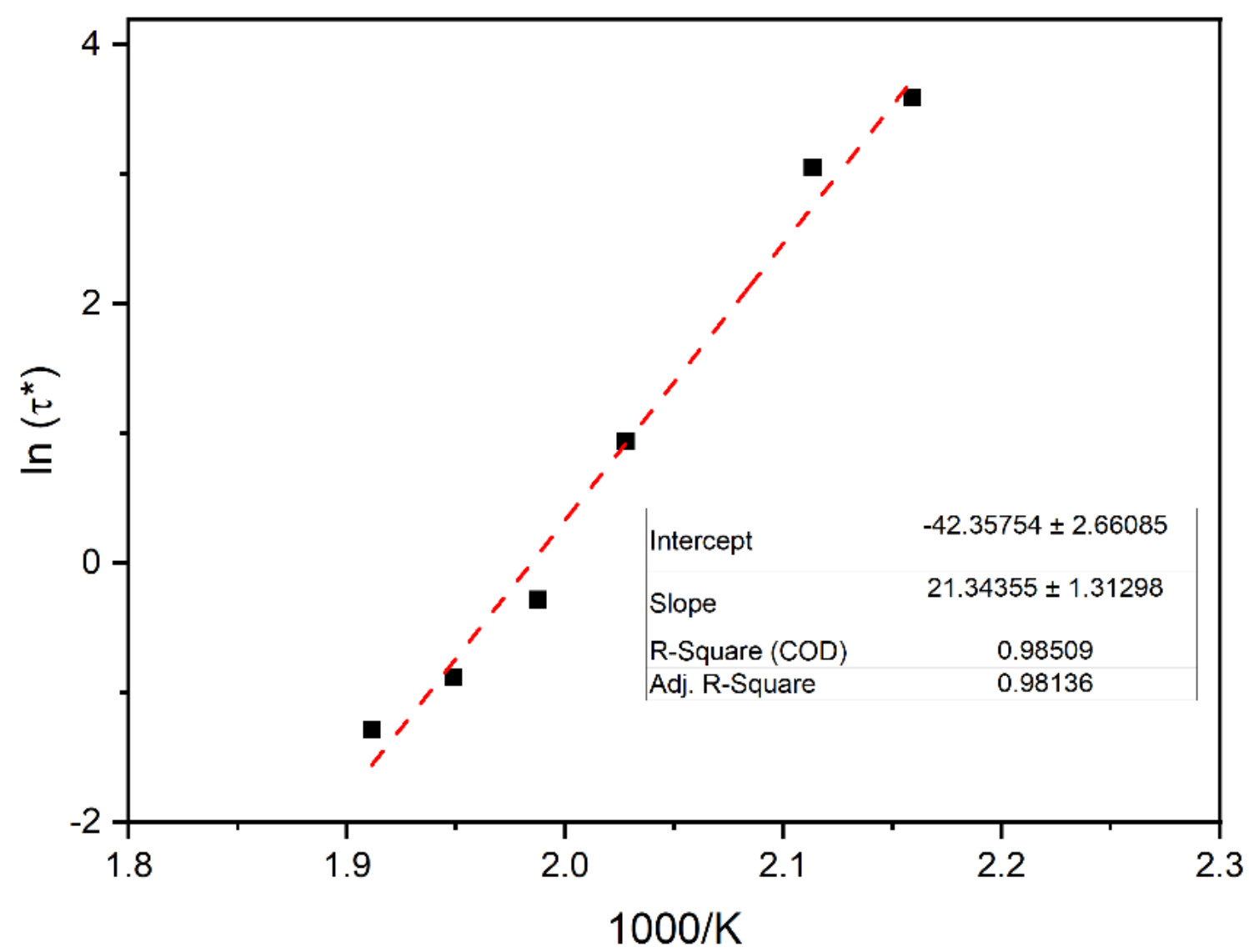

Figure S101. ln (τ*) vs 1000/K plot of HDPE with 5 wt% diazomalonate UDC.

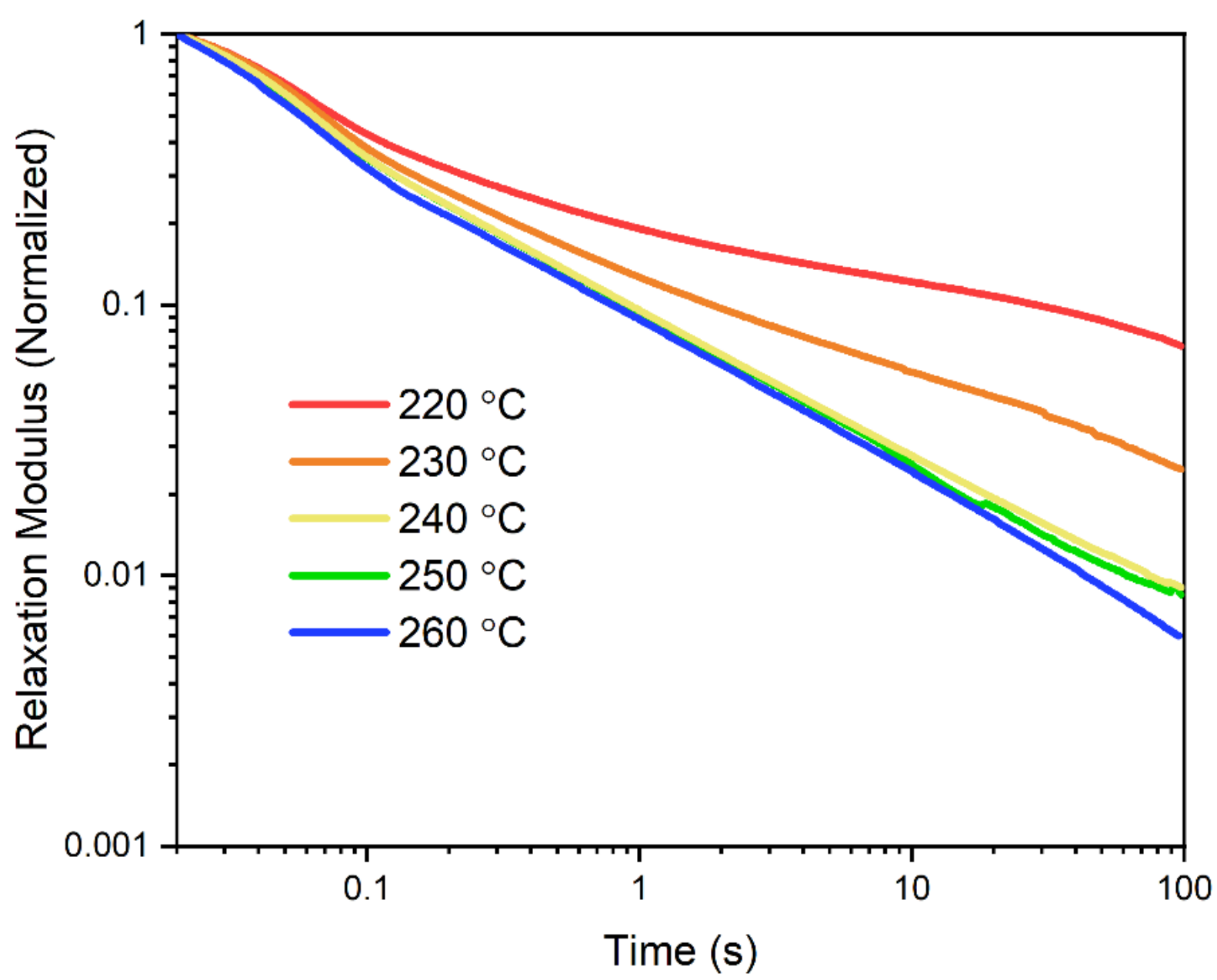

Figure S102. Stress relaxation of HDPE with 5 wt% diazomalonate UDC with 160 mg of bipyridine.

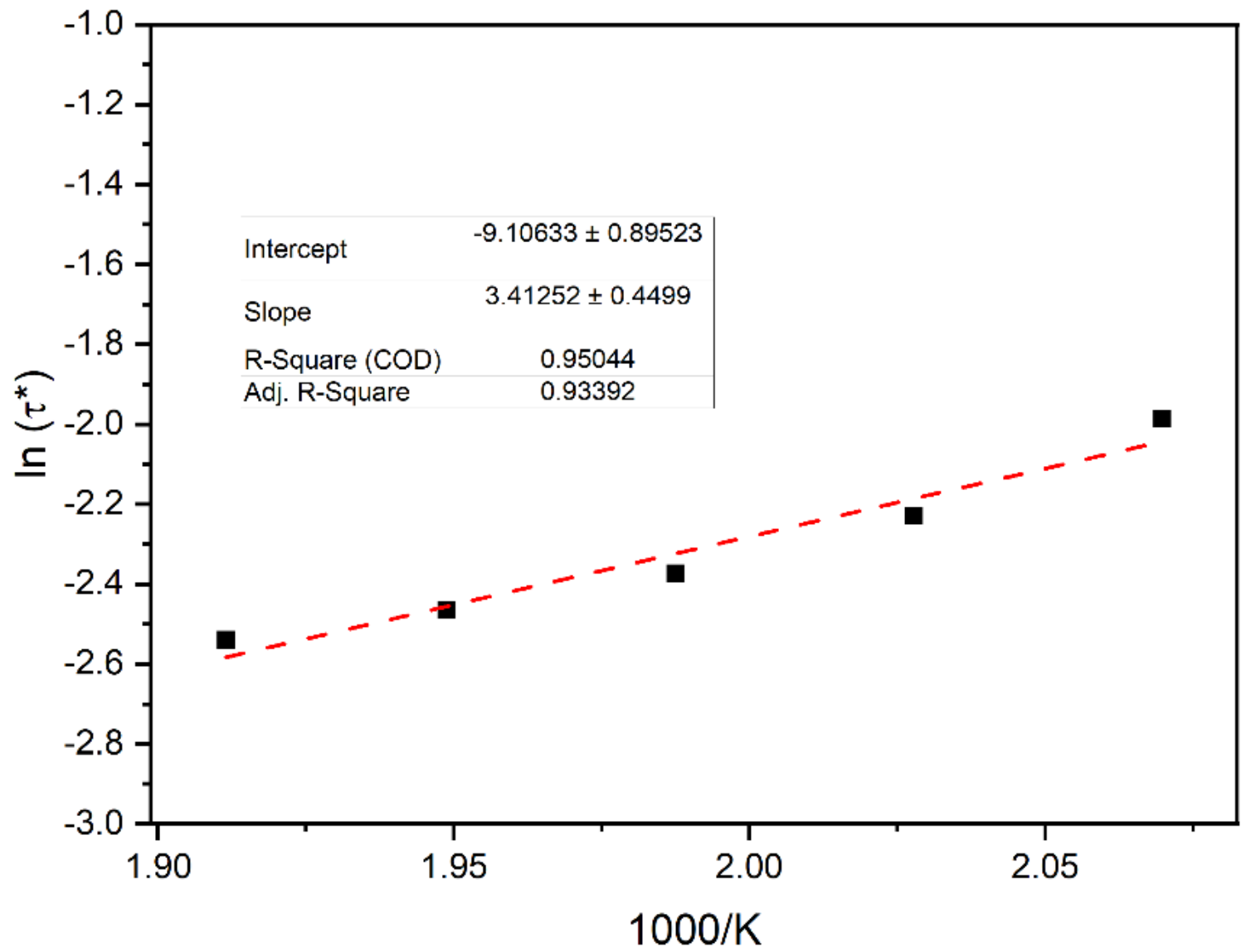

Figure S103. ln (τ*) vs 1000/K plot of HDPE with 5 wt% diazomalonate UDC and 160 mg of bipyridine.

Representative polymer samples

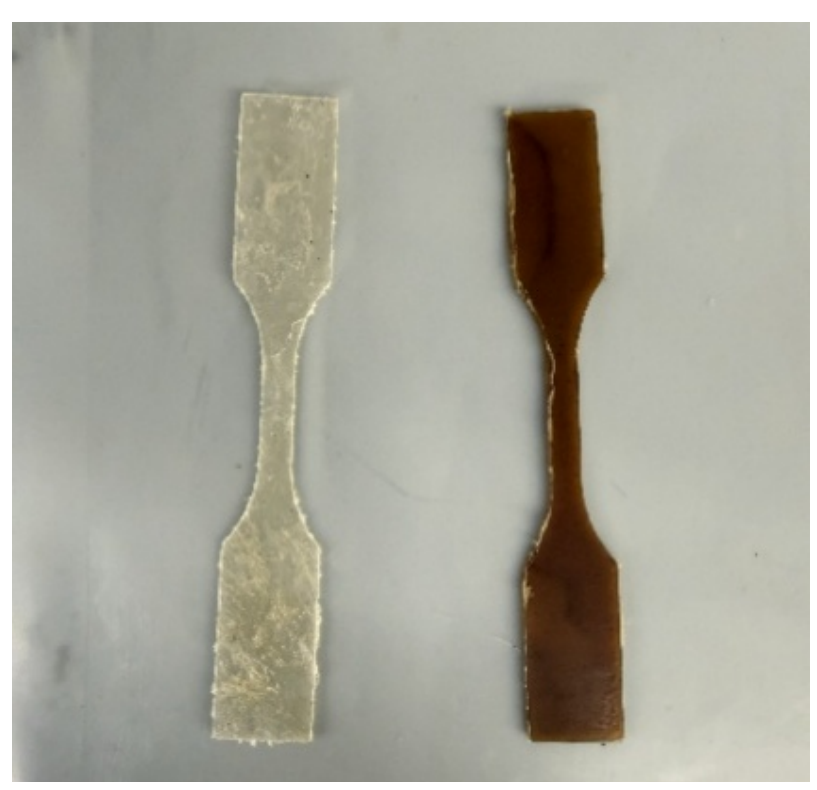

Figure S104. Photos of representative specimens of HDPE/Nylon-6 control (left) and HDPE/Nylon-6 with 5 wt% PT-DC (right) processed at 270 °C.

References

1. Yoshida, T.; Yoshizawa, F.; Itoh, M.; Matsunaga, T.; Watanabe, M. *Kogyo Kayak*. **1987**, *48*, 311–316.
2. Y. Gao, X. Westworth, E. C. Quinn, J. Nam, E. Y.-X. Chen, *Nat. Sustain.* **2026**, *9*, 260–270.
3. J. Castro, X. Westworth, R. Shrestha, K. Yokoyama, Z. Guan, *Adv. Mater.* **2024**, *36*, 2406203.
